# Merging carboxylic acids with metal-catalyzed hydrogen atom transfer (MHAT) chemistry *via* alkene-functionalized redox-active esters†

**DOI:** 10.1039/d5sc04274g

**Published:** 2025-07-23

**Authors:** Laura G. Rodríguez, Aina Serra, Josep Bonjoch, Ben Bradshaw

**Affiliations:** a Laboratori de Química Orgànica, Facultat de Farmàcia, IBUB, Universitat de Barcelona 08028 Spain benbradshaw@ub.edu

## Abstract

The development of general methods for radical bond formation remains a central goal in organic synthesis, particularly those that enable diverse transformations from simple, abundant starting materials. Here, we report a unified approach that merges carboxylic acid activation with metal-catalyzed hydrogen atom transfer (MHAT) to enable the generation and selective functionalization of open-shell intermediates under a single catalytic system. Key to this strategy is the design of a redox-active ester bearing an internal alkene “trigger” that undergoes regioselective MHAT using Fe(acac)_3_ and phenylsilane, leading to decarboxylative radical formation under mild conditions. This platform supports the synthesis of a wide array of products *via* C–C, C–heteroatom, and C–H bond-forming processes, accessed solely by varying the radical acceptor. Notably, it enables the formation of linear coupling products—previously inaccessible under conventional MHAT conditions—*via* access to primary radical intermediates. We anticipate that this conceptually distinct mode of activation will find applications in modular synthesis, late-stage functionalization, and the generation of medicinally relevant analogs.

## Abstract 2

A novel redox-active ester with an integrated alkene “trigger” unlocks carboxylic acids as alkene surrogates in MHAT couplings, expanding the substrate scope and enabling selective formation of linear coupled products for the first time.

## Graphical abstract

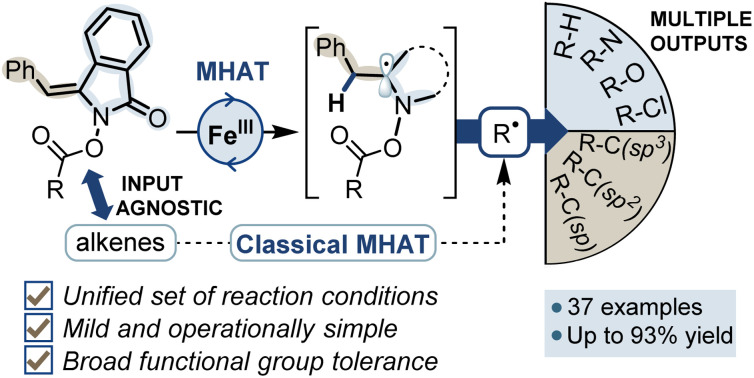

## Introduction

**Fig. 1:**
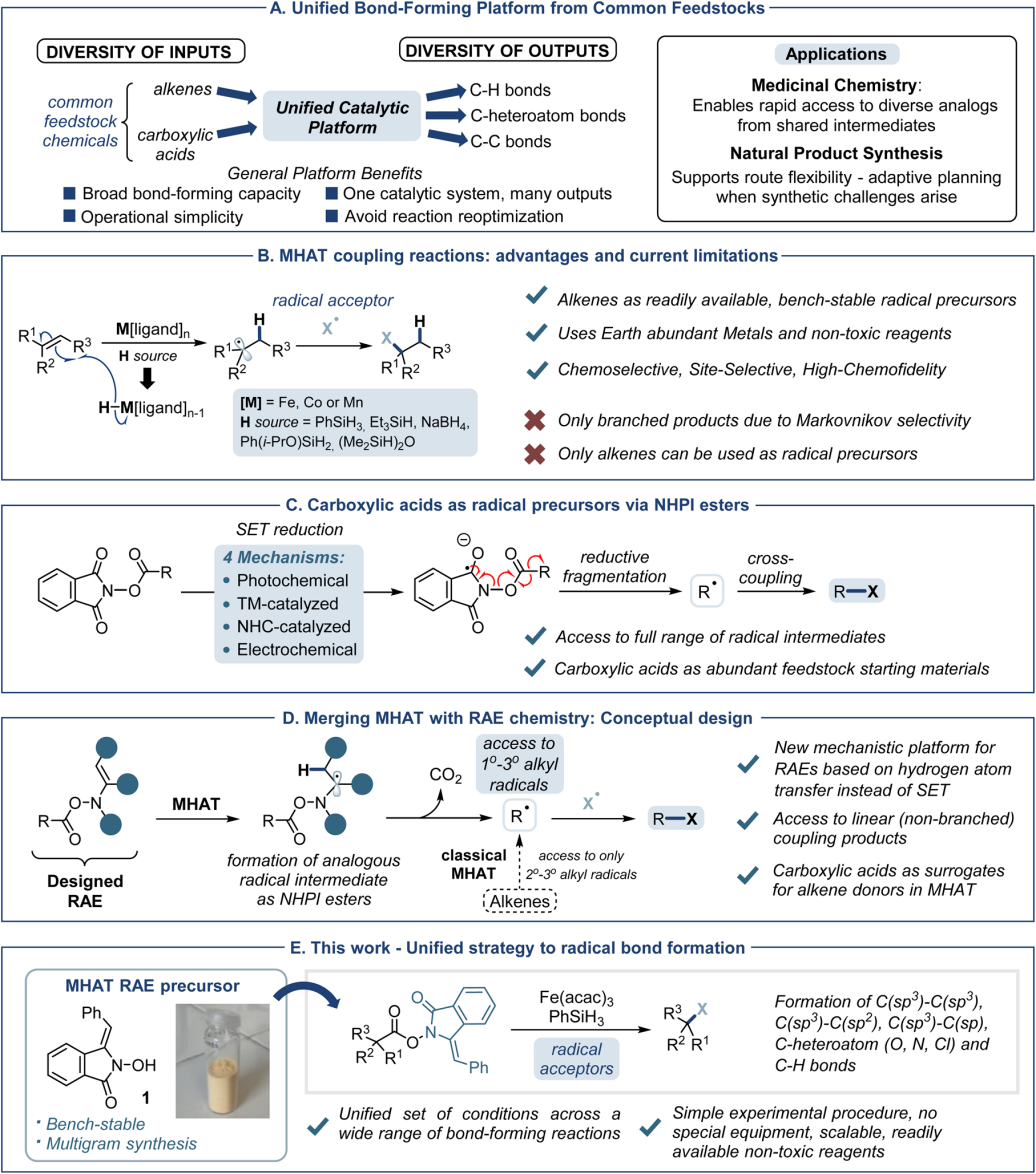
(A) Benefits of a unified bond-forming platform from abundant feedstock chemicals (B) MHAT coupling reactions overview and current limitations (C) carboxylic acids as radical precursors *via* NHPI esters (D) conceptual strategy to merge MHAT with RAE chemistry (E) this work: unified strategy to MHAT radical bond formation *via* use of an alkene-substituted RAE.

The ability to transform simple, abundant functional groups into structurally and functionally diverse products using a single, unified reaction framework represents a long-standing aspiration in synthetic chemistry.^1^ Strategies that decouple substrate identity from reaction outcome – by applying a common set of conditions across structurally distinct inputs – offer powerful platforms for modular synthesis, minimizing the need for case-by-case optimization. Such versatility is particularly valuable in complex molecule construction, where it enables route flexibility in natural product synthesis by accommodating diverse starting materials and bond-forming modes and accelerates medicinal chemistry efforts by providing rapid access to analogs from shared intermediates—ideal for structure–activity relationship studies and parallel synthesis workflows (Fig. 1A).

Efforts to achieve such unified reactivity frameworks have increasingly turned to radical chemistry – a field that, while once regarded as niche and difficult to control, has matured into a powerful and mainstream strategy in modern synthesis.^2^ The ability to harness single-electron reactivity has unlocked new frontiers in chemical space, enabling unique disconnections and novel reaction pathways that are inaccessible through traditional two-electron mechanisms.^3^ Beyond these strategic advantages, radical processes also offer significant practical benefits: broad functional group tolerance, orthogonal reactivity profiles, and a reduced reliance on protecting group strategies. These features make radical methodologies particularly well-suited for modular platforms that prioritize flexibility, diversity, and late-stage functionalization. The exploration of new chemical space has further accelerated with the emergence of metal-catalyzed hydrogen atom transfer (MHAT) as a powerful strategy for the hydrofunctionalization of alkenes through open-shell intermediates (Fig. 1B).^4^ This reaction proceeds *via* a metal hydride species, generated *in situ* from an earth-abundant first-row transition metal and a suitable hydride source. This species adds selectively to the least hindered end of the alkene, generating a radical intermediate that can be intercepted by a wide variety of acceptors.^5^ There are many inherent advantages to this methodology, including chemoselectivity,^6^ chemofidelity,^7^ site-selectivity,^8^ low toxicity,^9^ and compatibility with diverse activation modes.^10^ These benefits, together with novel disconnection possibilities, have established MHAT as a powerful platform for constructing natural products.^11^ Our group has been active in the development of MHAT chemistry over recent years, expanding its utility through a variety of new bond-forming transformations. These include the synthesis of tertiary alcohols^8*b*^ and amines,^12^ alkylated products,^13^ heterocycles,^14^ and *cis*-electron-deficient alkenes.^15^ Through these efforts, we began to recognize that two defining features of classical MHAT reactions – the reliance on alkenes as radical precursors and their intrinsic Markovnikov selectivity – can also impose important limitations. While alkenes are among the most abundant and accessible feedstocks, the chemical space accessible through MHAT cross-couplings is inherently constrained by their structural diversity. In addition, the Markovnikov selectivity that underpins traditional MHAT chemistry, although valuable for constructing branched products^16^ and sterically congested centers as demonstrated in our synthesis of the tricyclic core of (−)-4-*epi*-presilphiperfolan-8-ol,^17^ precludes the generation of primary radicals and thus limits access to linear coupling products. Overcoming these constraints would significantly broaden the scope and synthetic utility of MHAT catalysis.

In contrast, decarboxylative radical chemistry offers a mechanistically orthogonal route to a broader radical landscape. Upon conversion to redox-active esters (RAEs), these acids can serve as versatile radical precursors under a range of activation strategies, including photoredox, electrochemical, NHC-catalyzed, and metal-mediated single-electron transfer processes (Fig. 1C).^18^ These approaches offer access to primary, secondary, and tertiary radicals, and have transformed carboxylic acids into central building blocks in radical chemistry.^19^ However, they remain mechanistically distinct from MHAT catalysis, and integration of acid-derived radicals into MHAT logic has not yet been realized. Although a precedent exists from the work of Shenvi and Baran, which employed NHPI esters in an Fe-catalyzed S_H_2-type radical–radical coupling,^20^ this approach operates *via* a non-classical MHAT mechanism. Specifically, it relies on Fe(TPP)Cl – a porphyrin-based iron complex that cleaves the NHPI ester and sequesters the resulting primary radical for direct attack by another radical species. This precludes interception by external acceptors, making the system unsuitable as a general platform for MHAT hydrofunctionalization.

We therefore sought a general and operationally simple strategy that would enable carboxylic acids to serve as direct surrogates for alkenes in MHAT reactions, without modifying the existing catalytic platform (Fig. 1D). To this end, we proposed the design of a specialized RAE bearing an integrated alkene “trigger” which under standard MHAT conditions would generate a radical adjacent to the N–O bond, analogous to NHPI esters. This intermediate should spontaneously fragment to furnish the corresponding decarboxylated radical, which could be intercepted downstream by a broad range of radical acceptors. After considering various designs for the alkene-functionalized RAE in terms of stability, accessibility, and reactivity (see ESI†), we opted for using the phenylethylene-substituted phthalimide derivative 1 ^21^ which could be prepared in multigram batches as a bench-stable solid.

Herein, we report the development of a unified MHAT-based activation strategy that enables carboxylic acids to serve as direct radical precursors across a broad spectrum of C–H, C–C, and C–heteroatom bond-forming reactions (Fig. 1E). This approach proceeds *via* a redox-active ester and operates under a single, operationally simple catalytic system. By accessing primary, secondary, and tertiary radicals from structurally diverse acids – and circumventing the Markovnikov bias inherent to classical MHAT – this platform significantly expands both the scope and synthetic potential of MHAT chemistry.

## Results and discussion

To evaluate the generation of different radical species from carboxylic acids under MHAT conditions, we selected the Giese coupling reaction^22^ as a representative platform to demonstrate the breadth of accessible radical types. Since primary radicals are inaccessible under conventional MHAT conditions from alkenes, we began our investigation with compound 2a, designed to showcase the unique ability of this system to access such species. Using methyl acrylate as a model acceptor, we found that the desired product 3 could be obtained in excellent yield (93%) using 10 mol% Fe(acac)_3_ as the catalyst and 2.5 equivalents of PhSiH_3_ as the hydride source in THF, with 10 equivalents of MeOH as an additive at room temperature (entry 1). The use of heating and EtOH as the solvent led to a significant formation of the transesterification product 3′ (entries 2 and 3). To rule out that classical *N*-hydroxyphthalimide esters could be used in this reaction, the NHPI ester 2a′ was synthesized and exposed to the coupling conditions (entry 4).^23^ However, no coupled or decarboxylated products were observed, including with the addition of 1.0 equivalent of Fe(acac)_2_ under the same conditions (entry 5).^20*b*^ Replacing EtOH with DCE as the solvent (entry 6) to avoid the transesterification product yielded only traces of 3, indicating that an alcoholic solvent is essential for the reaction.^24^ Modifying the amount of acceptor (entries 7 and 8) proved detrimental to the reaction, while the amount of iron catalyst had little effect on the yield (entries 9 and 10), which improved slightly under lower loadings. Attempts to use alternative catalysts such as Mn(dpm)_3_ or Co(Sal^*t*Bu,*t*Bu^)Cl (entries 11 and 12) were unsuccessful. Finally, reducing the amount of PhSiH_3_ lowered the yield (entry 13).

**Fig. 2:**
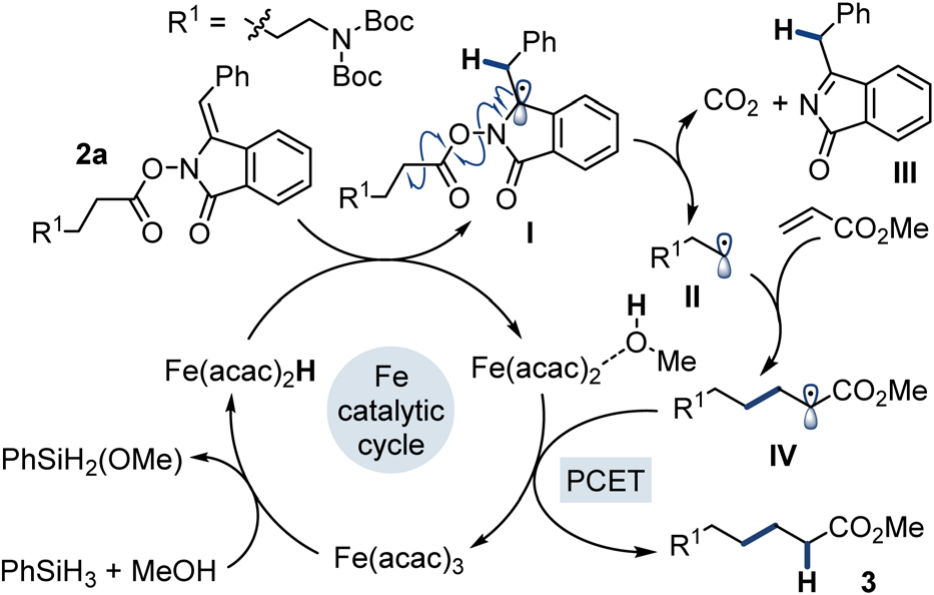
Proposed mechanism for the reaction.

Our proposed mechanism^22*c*,25^ for the reaction starts with the formation of an iron hydride species, which then adds to the alkene of the *N*-(acyloxy)phthalimide derivative 2a (Fig. 2). This results in the formation of I with a radical adjacent to the nitrogen atom, which initiates the decarboxylation sequence to give the primary radical species II along with carbon dioxide and III. Coupling of the generated radical with the acceptor gives IV, which is reduced by the Fe(ii) species and a molecule of MeOH in a proton-coupled electron transfer process (PCET)^24^ to yield 3. This step is supported by deuterium labeling studies with MeOD, which led to deuterium incorporation at the expected position in the product. In this process, Fe(ii) is also oxidized back to Fe(iii), completing the catalytic cycle. Evidence for the MHAT mechanism comes from Table 1 entries 4 and 5, which clearly demonstrate that the radical does not form either *via* SET process or direct fragmentation of the N–O bond.

**Table 1:** Optimization and control reactions

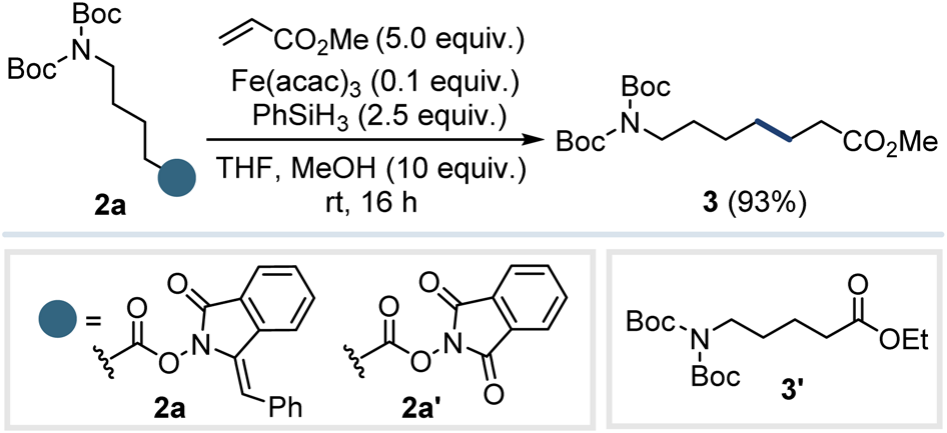		
Entry^a^	Deviation from optimum conditions	Yield^b^
1	No deviation	93
2	60 °C instead of rt, EtOH as solvent	55
3	EtOH as solvent	74
4	2a′ instead of 2a	—
5	2a′ instead of 2a and 1.0 equiv. Fe(acac)_2_	—
6	DCE instead of THF/MeOH	2
7	10 equiv. of acceptor instead of 5.0 equiv.	35
8	2.0 equiv. of acceptor	74
9	0.25 equiv. of Fe(acac)_3_	89
10	0.5 equiv. of Fe(acac)_3_	90
11	Mn(dpm)_3_ instead of Fe(acac)_3_	4
12	Co(Sal^*t*Bu,*t*Bu^)Cl instead of Fe(acac)_3_	—
13	1.0 equiv. of PhSiH_3_	64

a All reactions were carried out on a 0.20 mmol scale except the final optimized reaction which was carried out on a 1.0 mmol scale (91% on a 0.20 mmol scale).

b All yields are isolated.

**Fig. 3:**
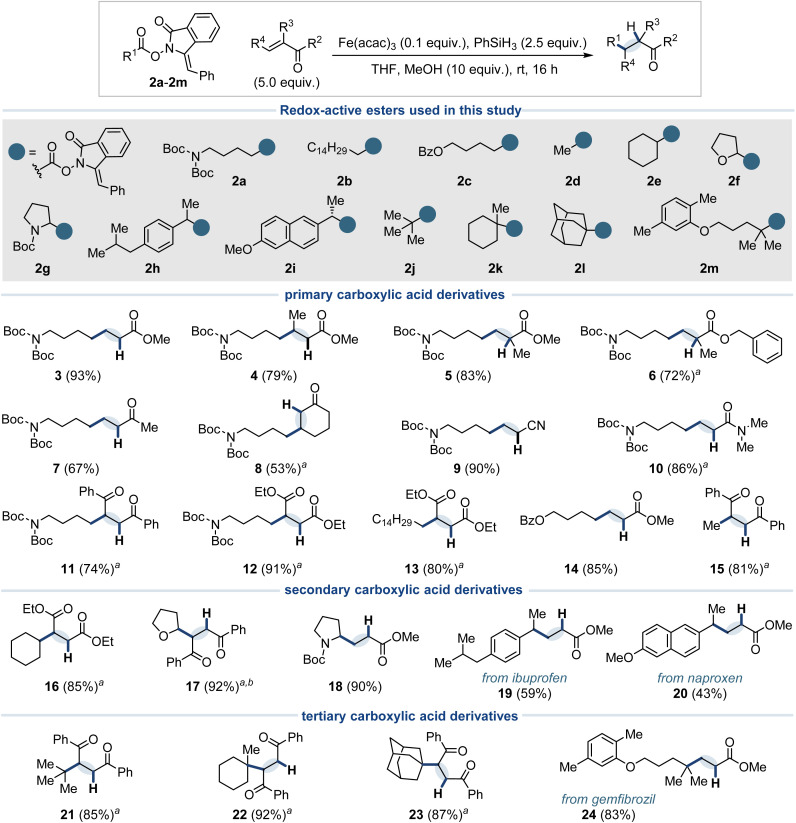
Scope of electron-deficient alkenes and novel *N*-(acyloxy)phthalimide derivatives. ^*a*^2.0 equivalents of the acceptor were used. ^*b*^Isolated as a mixture of diastereoisomers.

With the optimum conditions in hand, we started to investigate the scope of the reaction (Fig. 3). The coupling was found to be compatible with a broad range of Michael acceptors bearing assorted electron-withdrawing groups, including esters with different substitution patterns (3–6), ketones (7–8), nitriles (9), and amides (10). Acceptors bearing two electron-withdrawing groups also proved to be viable coupling partners (11–12). Finally, other primary radical donors were evaluated with good results (13–15). The methyl radical donor 2d is of particular note as the introduction of the methyl unit is highly prized in medicinal chemistry due to the profound pharmacological effects it can exert *via* the “magic methyl” effect.^26^ Next, a range of secondary radical precursors (2e–i) were assessed. In cases where the product was predicted to be volatile, methyl acrylate was replaced with an acceptor bearing two electron-withdrawing groups. Cyclohexane carboxylic acid, tetrahydro-2-furoic acid, and *N*-boc proline derivatives (2e–g) were all coupled in excellent yields, giving 16–18, respectively. The presence of a stabilizing heteroatom was found to benefit the coupling process and did not necessitate the use of alternative iron catalysts such as Fe(dibm)_3_, which are required when using alkenes as radical precursors.^18*b*^ In comparison, the yield of the benzylic radicals was somewhat lower, with the derivatives from ibuprofen (2h) and naproxen (2i) giving 19 and 20 in 59% and 43% yields, respectively. All tertiary radical precursors studied (2j–m) showed excellent results, allowing the coupling of the *tert*-butyl group (21), methylcyclohexane (22), and adamantane (23) fragments.

Finally, the pharmaceutical gemfibrozil derivative 2m could be coupled with methyl acrylate to give 24 in 83% yield.

**Fig. 4:**
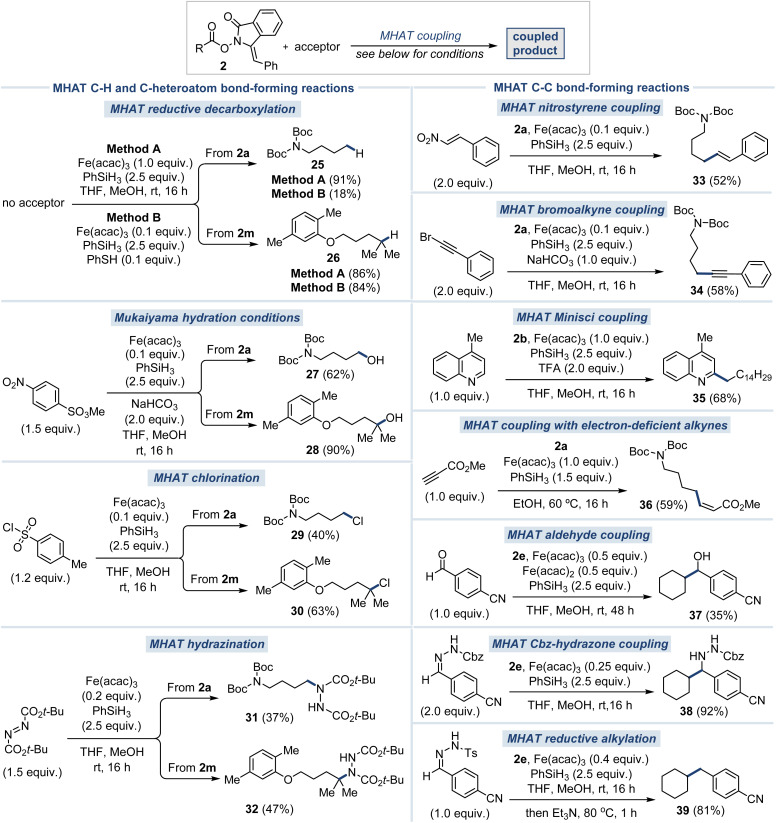
Evaluation of novel *N*-(acyloxy)phthalimide derivatives in a broad cross-section of established MHAT reactions ^*a*^all reactions are unoptimized. For detailed conditions, see ESI.† One equivalent of RAE was used in each case.

We next evaluated the generality of this radical platform across a range of previously reported MHAT-type reactions, spanning C–H, C–heteroatom and C–C bond formation, using structurally distinct RAEs without modification to the catalytic system (Fig. 4). Treatment of 2a and 2m in the absence of an acceptor and with stoichiometric Fe(acac)_3_ (Method A) gave excellent yields of the decarboxylated compounds 25 and 26, respectively, providing an operationally simple way to perform Barton-type decarboxylations under mild conditions.^27^ A catalytic version using 10% PhSH^28^ as an additive was also developed (Method B), with the yield for 2m being almost identical to that of the stoichiometric version. However, 2a gave only an 18% yield of the desired decarboxylation product 25, which was attributed to competitive quenching of the highly reactive primary radical by the metal hydride species.

Next, Studer's Mukaiyama-type hydration employing methyl 4-nitrobenzenesulfonate as the acceptor^29^ afforded good yield of the corresponding alcohol product 27, and excellent yield of 28. Notably, such direct access to alcohols is not achievable through conventional radical decarboxylation strategies, which typically require pre-functionalization to a boron species^30^ or trapping with TEMPO,^31^ followed by an additional hydrolysis step. Next, Carreira's hydrochlorination^32^ and hydrazination^33^ methods, both originally developed using cobalt catalysis with TsCl and di-*tert*-butyl azodicarboxylate as radical acceptors, respectively, were successfully adapted under our MHAT conditions. These reactions furnished chlorides 29 and 30, and nitrogen-containing derivatives 31 and 32, demonstrating the broad compatibility of our platform with such radical functionalizations without requiring catalyst modification.

To illustrate the broad versatility of our strategy in C–C bond formation, we explored coupling with a variety of acceptors, enabling access to diverse products featuring sp^3^-, sp^2^-, and sp-hybridized carbon centers under our MHAT conditions. Beginning with Cui's nitrostyrene^34^ and bromoalkyne^35^ couplings utilizing RAE 2a, this approach afforded the linearly coupled alkene 33 and alkyne 34, respectively, thereby enabling access to compounds previously unattainable under the original methodologies. The intermolecular Minisci coupling reaction of 2b with lepidine, applying conditions developed in our studies of isocyanides,^14^ gave the substituted heterocycle 35 in 68% yield, and our recently developed coupling to electron deficient alkynes gave alkene 36 in 59% yield primarily as the *cis* isomer.^15^ Finally, based on our previous work using aldehydes,^36^ Cbz-hydrazones,^12^ and Ts-hydrazones^13^ as acceptors in MHAT reactions with alkenes, the cyclohexyl radical derived from 2e was coupled to give alcohol 37, hydrazine 38, and the alkylation product 39, respectively.^37^

## Conclusions

In summary, we have developed a redox-active ester that allows carboxylic acids to act as surrogates for alkenes in a wide range of MHAT transformations, operating through a novel activation mode that is mechanistically distinct from conventional SET processes. In contrast to the intrinsic Markovnikov selectivity of traditional alkene-based MHAT, this approach allows for the formation of linear coupling products for the first time. By expanding both the scope of accessible radical precursors and the types of bond constructions enabled, this strategy significantly broadens the synthetic potential of MHAT catalysis. Moreover, by integrating carboxylic acid activation into classical MHAT logic, this work establishes a unified reaction platform capable of accommodating structurally diverse substrates under a single catalytic regime. Ongoing studies in our group are aimed at extending this platform to new classes of transformations and applications.

## Author contributions

L. G. R. contributed to the experimental work and writing of the paper; A. S. contributed to the experimental work; J. B. contributed to the writing of the paper; B. B. contributed to the ideation and writing of the paper.

## Conflicts of interest

There are no conflicts to declare.

## Supplementary Material

SC-OLF-D5SC04274G-s001

## Data Availability

The data supporting this article have been included as part of the ESI.†
