# Supplementary material for: Merging carboxylic acids with metal-catalyzed hydrogen atom transfer (MHAT) chemistry via alkene-functionalized redox-active esters
Source: Chem Sci. 2025 Jul 23;16(34):15478–85. doi: 10.1039/d5sc04274g (PMC12305676; doi:10.1039/d5sc04274g)

Supplementary Information (SI) for Chemical Science.
This journal is © The Royal Society of Chemistry 2025

Supporting Information

Merging Carboxylic Acids with Metal-Catalyzed Hydrogen Atom Transfer (MHAT) Chemistry via Alkene-Functionalized Redox-Active Esters

Laura G. Rodríguez,† Aina Serra,† Josep Bonjoch,† and Ben Bradshaw*,†

†Laboratori de Química Orgànica, Facultat de Farmàcia, IBUB, Universitat de Barcelona, Av. Joan XXIII 27-31, 08028, Barcelona, Spain.
E-mail: benbradshaw@ub.edu

Contents

General information 2
Preliminary studies for the synthesis of the ligand........ 3
Experimental section 4
Copies of NMR spectra 23

GENERAL INFORMATION

All reactions were carried out under an argon atmosphere with dry, freshly distilled solvents under anhydrous conditions. Analytical thin-layer chromatography was performed on SiO_2 (Merck silica gel 60 F_{254}), and the spots were located with 1% aqueous $KMnO_4$ or 2% ethanolic anisaldehyde. Chromatography refers to flash chromatography and was carried out on SiO_2 (SDS silica gel 60 ACC, 35-75 μm, 230-240 mesh ASTM) or aluminium oxide (neutral) pH 6.5-7.5 (63-200 μm). Drying of organic extracts during workup of reactions was performed over anhydrous Na_2SO_4. Evaporation of solvent was accomplished with a rotary evaporator. NMR spectra were recorded in $CDCl_3$ except where stated otherwise and the chemical shifts of ^1H and ^{13}C NMR spectra are reported in ppm downfield (δ) from Me_4Si or $CDCl_3$. All NMR data assignments are supported by gCOSY and gHSQC experiments. High resolution mass spectra (HMRS) were performed using an electrospray (ESI) ionization source and a TOF analyzer (Agilent Technologies).

PRELIMINARY STUDIES FOR THE SYNTHESIS OF THE LIGAND

(a) Two-step synthesis of the MHAT decarboxylation precursors

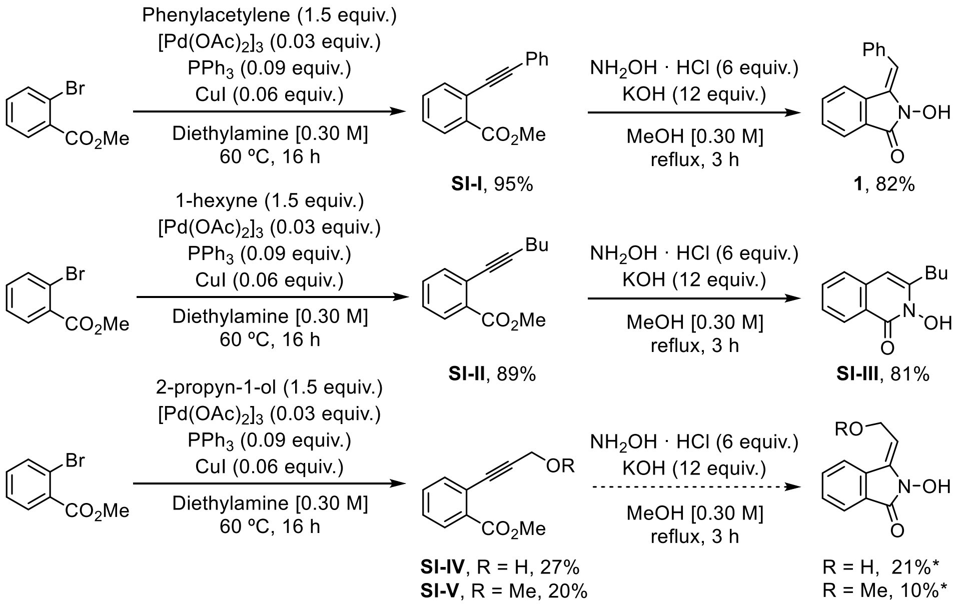

* The products could not be characterized as they were isolated alongside unidentified impurities.

(b) One-pot synthesis of the MHAT decarboxylation precursors

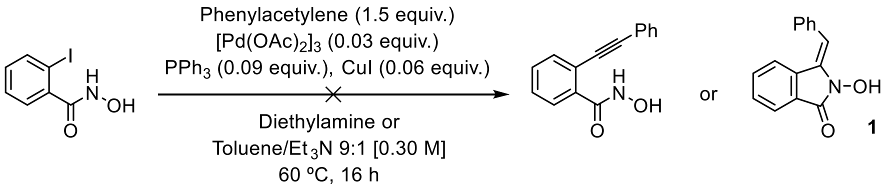

c) Study of the decarboxylative reaction under standard MHAT conditions

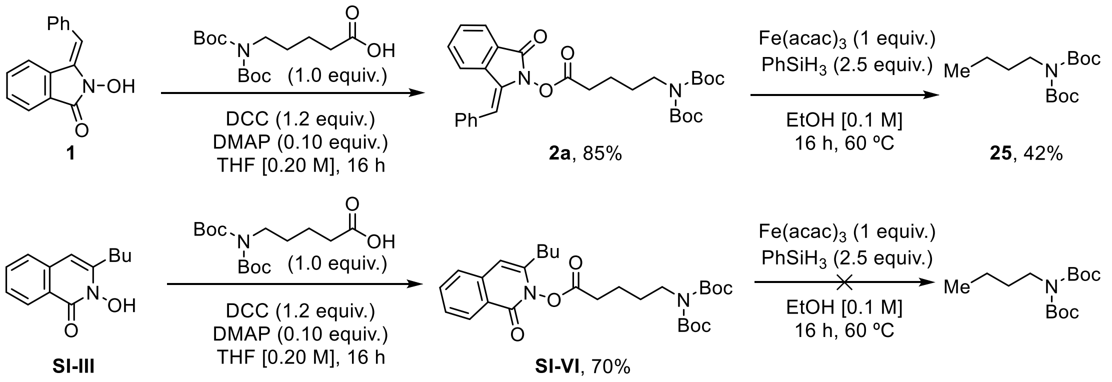

d) Synthesis of *N,N*-di-Boc aminovaleric acid

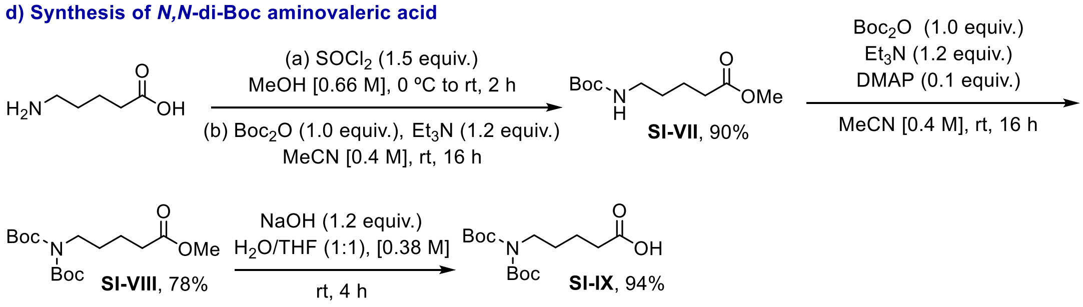

EXPERIMENTAL SECTION

SYNTHESIS OF THE REDOX-ACTIVE ESTERS

Synthesis of 3-benzylidene-2-hydroxyisoindolin-1-one (1)[1]

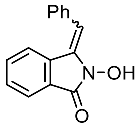

A mixture of potassium hydroxide (14.2 g, 253.5 mmol, 12.0 equiv.) and hydroxylamine hydrochloride (8.80 g, 126.7 mmol, 6.0 equiv.) was dissolved in anhydrous methanol (211 mL, 0.30 M). Afterward, methyl 2-(phenylethynyl)benzoate (4.98 g, 21.1 mmol, 1.0 equiv.) was added dropwise and the resulting mixture was heated at reflux for 3 hours. Afterward, the reaction mixture was concentrated under vacuum. The crude product was dissolved in dichloromethane and washed with 1.0 M HCl. Purification by chromatography (Hex/AcOEt 10% → AcOEt) gave **1** as a light orange solid (4.11 g, 82%). The NMR data reported correspond to the major isomer, with a 91/9 proportion of isomers. ^{1}H NMR (400 MHz, DMSO-*d*$_6$) δ 10.65 (s, 1H), 8.02 (d, *J* = 7.7 Hz, 1H), 7.74 (d, *J* = 7.5 Hz, 1H), 7.69 (td, *J* = 7.6, 1.2 Hz, 1H), 7.62 – 7.58 (m, 2H), 7.55 (td, *J* = 7.5, 0.8 Hz, 1H), 7.34 (t, *J* = 7.5 Hz, 2H), 7.29 – 7.22 (m, 1H), 6.88 (s, 1H); ^{13}C{^{1}H} NMR (101 MHz, DMSO-*d*$_6$) δ 163.2, 134.5, 133.9, 132.3, 131.6, 130.6, 129.3, 127.6, 127.1, 125.5, 122.4, 120.0, 106.8 ppm. HRMS (ESI) m/z: [M+H]$^+$: calculated for $C_{15}H_{12}NO_2^+$ 238.0863, found 238.0863.

General method for the synthesis of the redox-active esters:[2] A solution of the selected acid (1.0 equiv.), 3-benzylidene-2-hydroxyisoindolin-1-one (1.0 equiv.), dicyclohexylcarbodiimide (1.2 equiv.) and DMAP (0.10 equiv.) in THF (0.20 M) was left to stir for 16 hours at room temperature. Afterward, the reaction mixture was concentrated under vacuum. The corresponding product was purified by flash column chromatography on silica gel.

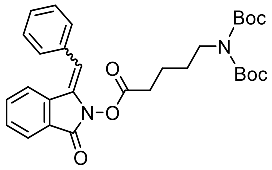

1-benzylidene-3-oxoisoindolin-2-yl 5-(bis(tert-butoxycarbonyl)amino)pentanoate (**2a**). The title compound was prepared according to the general procedure using 5-((di-*tert*-butoxycarbonyl)amino)pentanoic acid (1.71 g, 5.40 mmol, 1.0 equiv.), 3-benzylidene-2-hydroxyisoindolin-1-one (1.28 g, 5.40 mmol, 1.0 equiv.), dicyclohexylcarbodiimide (1.34 g, 6.48 mmol, 1.2 equiv.) and DMAP (66 mg, 0.54 mmol, 0.10 equiv.) in THF (27 mL, 0.20 M). Purification by chromatography (Hex → Hex/AcOEt 50%) gave **2a** (2.46 g, 85%) as a white solid in a 81/19 proportion of *E*/*Z* isomers. ^{1}H NMR (400 MHz, $CDCl_3$) δ 7.93 – 7.85 (m, 1H), 7.78 – 7.71 (m, 1H), 7.69 – 7.63 (m, 1H), 7.58 – 7.49 (m, 1H), 7.43 – 7.32 (m, 4H), 7.34 – 7.25 (m, 1H), 6.70 (s, 0.2H), 6.68 (s, 0.8H), 3.71 – 3.58 (m, 0.1H),

---

[1] S. F. Vasilevskii, T. F. Mikhailovskaya, A. A. Stepanov, V. I. Mamatyuk and D. S. Fadeev, *Russ. J. Org. Chem.*, **2014**, *50*, 506–509.
[2] A. Fawcett, J. Pradeilles, Y. Wang, T. Mutsuga, E. L. Myers and V. K. Aggarwal, *Science*, **2017**, *357*, 283–286.

3.43 (t, $J = 6.8$ Hz, 1.7H), 2.27 – 2.09 (m, 1H), 1.82 – 1.65 (m, 1H), 1.65 (s, 1H), 1.50 (s, 16H), 1.41 – 1.30 (m, 4H); $^{13}C\{^1H\}$ NMR (101 MHz, $CDCl_3$) δ 169.4, 166.8, 164.0, 152.6, 135.0, 133.2, 131.9, 130.1, 130.0, 129.6, 128.0, 127.8, 125.5, 124.1, 119.9, 106.8, 82.3, 45.7, 30.2, 28.2, 28.2, 21.3 ppm. HRMS (ESI) m/z: $[M+Na]^+$: calculated for $C_{30}H_{37}N_2NaO_7^+$ 559.2415, found 559.2418.

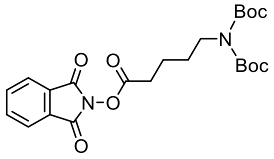

1,3-dioxoisoindolin-2-yl 5-((di-tert-butoxycarbonyl)amino)pentanoate (**2a'**). The title compound was prepared according to the general procedure using 5-((di-*tert*-butoxycarbonyl)amino)pentanoic acid (476 mg, 1.50 mmol, 1.0 equiv.), *N*-hydroxyphthalimide (245 mg, 1.50 mmol, 1.0 equiv.), dicyclohexylcarbodiimide (371 mg, 1.80 mmol, 1.2 equiv.) and DMAP (18 mg, 0.15 mmol, 0.10 equiv.) in THF (7.5 mL, 0.20 M). Purification by chromatography (Hex → Hex/AcOEt 50%) gave **2a'** (569 mg, 82%) as a white solid. ^1H NMR (400 MHz, $CDCl_3$) δ 7.90 – 7.84 (m, 2H), 7.81 – 7.74 (m, 2H), 3.62 (t, $J = 6.9$ Hz, 2H), 2.69 (t, $J = 7.2$ Hz, 2H), 1.86 – 1.65 (m, 4H), 1.50 (s, 18H); $^{13}C\{^1H\}$ NMR (101 MHz, $CDCl_3$) δ 169.4, 162.0, 152.7, 134.9, 129.1, 124.1, 82.4, 45.7, 30.8, 28.4, 28.2, 22.0 ppm. HRMS (ESI) m/z: $[M+H]^+$: calculated for $C_{23}H_{31}N_2O_8^+$ 463.2075, found 463.2071.

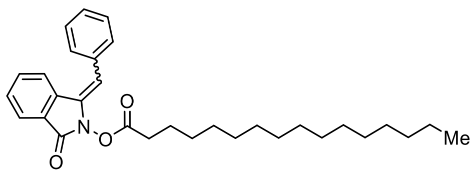

1-benzylidene-3-oxoisoindolin-2-yl palmitate (**2b**). The title compound was prepared according to the general procedure using palmitic acid (385 mg, 1.50 mmol, 1.0 equiv.), 3-benzylidene-2-hydroxyisoindolin-1-one (356 mg, 1.50 mmol, 1.0 equiv.), dicyclohexylcarbodiimide (371 mg, 1.80 mmol, 1.2 equiv.) and DMAP (18 mg, 0.54 mmol, 0.10 equiv.) in THF (7.5 mL, 0.20 M). Purification by chromatography (Hex → Hex/AcOEt 50%) gave **2b** (578 mg, 81%) as a yellow oil in a 94/6 proportion of *E*/*Z* isomers. ^1H NMR (400 MHz, $CDCl_3$) δ 7.89 (d, $J = 7.5$ Hz, 1H), 7.75 (dd, $J = 7.8$, 0.9 Hz, 1H), 7.70 – 7.61 (m, 1H), 7.53 (t, $J = 7.5$ Hz, 1H), 7.44 – 7.37 (m, 2H), 7.40 – 7.31 (m, 2H), 7.32 – 7.26 (m, 1H), 6.68 (s, 1H), 2.21 – 2.06 (m, 1H), 1.76 – 1.59 (m, 1H), 1.44 – 0.99 (m, 26H), 0.87 (t, 3H); $^{13}C\{^1H\}$ NMR (101 MHz, $CDCl_3$) δ 169.8, 164.2, 135.1, 133.2, 133.2, 132.0, 130.1, 129.6, 128.0, 127.8, 125.6, 124.2, 119.9, 106.9, 32.1, 30.6, 29.8, 29.8, 29.8, 29.8, 29.7, 29.5, 29.5, 29.2, 28.8, 24.1, 22.8, 14.3 ppm. HRMS (ESI) m/z: $[M+H]^+$: calculated for $C_{31}H_{42}NO_3^+$ 476.3159, found 476.3157.

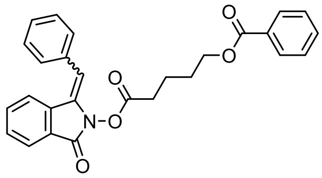

(E)-5-((1-benzylidene-3-oxoisoindolin-2-yl)oxy)-5-oxopentyl benzoate (**2c**). The title compound was prepared according to the general procedure using 5-(benzoyloxy)pentanoic acid (333 mg, 1.50 mmol, 1.0 equiv.), 3-benzylidene-2-hydroxyisoindolin-1-one (356 mg, 1.5 mmol, 1.0 equiv.), dicyclohexylcarbodiimide (371 mg, 1.8 mmol, 1.2 equiv.) and DMAP (18 mg, 0.15 mmol, 0.10 equiv.) in THF (7.5 mL, 0.20 M). Purification by chromatography (Hex → Hex/AcOEt 50%) gave **2c** (542 mg, 82%) as a yellow oil in a 94/6 proportion of *E*/*Z* isomers. ^1H NMR (500 MHz, $CDCl_3$) δ 8.02 (dd, $J = 8.4$, 1.3 Hz, 1H), 7.90 (dt, $J = 7.6$, 1.0 Hz, 1H), 7.76 (dt, $J = 7.8$, 0.9 Hz, 1H), 7.68 (td, $J = 7.6$, 1.1 Hz, 1H), 7.60 – 7.51 (m, 1H), 7.48 – 7.41 (m, 1H), 7.43 – 7.38 (m, 1H), 7.37 – 7.30 (m, 1H), 7.30 – 7.23 (m, 1H), 6.69 (s, 1H), 4.18 (t, $J = 6.2$ Hz, 1H), 2.34 – 2.19 (m, 1H), 1.87 – 1.72 (m, 1H), 1.63 – 1.40 (m, 1H); $^{13}C\{^1H\}$ NMR (126 MHz, $CDCl_3$) δ 169.4, 166.6, 164.2, 135.1, 133.3, 133.2, 133.1,

132.0, 130.4, 130.1, 129.72, 129.68, 128.5, 128.1, 127.9, 125.5, 124.2, 120.0, 106.9, 64.3, 30.1, 27.8, 20.9 ppm. HRMS (ESI) m/z: $[M+H]^+$: calculated for $C_{27}H_{24}NO_5^+$ 442.1649, found 442.1651.

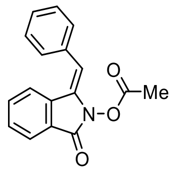

1-benzylidene-3-oxoisoindolin-2-yl acetate (**2d**). The title compound was prepared according to the general procedure using acetic acid (90 mg, 86 μL, 1.50 mmol, 1.0 equiv.), 3-benzylidene-2-hydroxyisoindolin-1-one (356 mg, 1.50 mmol, 1.0 equiv.), dicyclohexylcarbodiimide (371 mg, 1.80 mmol, 1.2 equiv.) and DMAP (18 mg, 0.15 mmol, 0.10 equiv.) in THF (7.5 mL, 0.20 M). Purification by chromatography (Hex → Hex/AcOEt 50%) gave **2d** (335 mg, 80%) as a yellow oil. ^{1}H NMR (400 MHz, $CDCl_3$) δ 7.90 (ddd, *J* = 7.6, 1.2, 0.8 Hz, 1H), 7.76 (dt, *J* = 7.8, 0.9 Hz, 1H), 7.67 (ddd, *J* = 7.8, 7.4, 1.2 Hz, 1H), 7.54 (td, *J* = 7.5, 1.0 Hz, 1H), 7.43 – 7.29 (m, 5H), 6.71 (s, 1H), 1.65 (s, 3H); $^{13}C\{^1H\}$ NMR (101 MHz, $CDCl_3$) δ 166.8, 163.8, 135.0, 133.2, 133.1, 131.8, 130.0, 129.7, 128.1, 127.8, 125.6, 124.2, 119.9, 106.9, 17.2 ppm. HRMS (ESI) m/z: $[M+H]^+$: calculated for $C_{17}H_{14}NO_3^+$ 280.0968, found 280.0968.

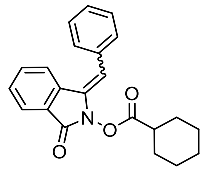

1-benzylidene-3-oxoisoindolin-2-yl cyclohexanecarboxylate (**2e**). The title compound was prepared according to the general procedure using cyclohexanecarboxylic acid (192 mg, 1.50 mmol, 1.0 equiv.), 3-benzylidene-2-hydroxyisoindolin-1-one (356 mg, 1.50 mmol, 1.0 equiv.), dicyclohexylcarbodiimide (371 mg, 1.80 mmol, 1.2 equiv.) and DMAP (18 mg, 0.15 mmol, 0.10 equiv.) in THF (7.5 mL, 0.20 M). Purification by chromatography (Hex → Hex/AcOEt 50%) gave **2e** (453 mg, 87%) as a yellow oil in a 93/7 proportion of *E*/*Z* isomers. ^{1}H NMR (400 MHz, $CDCl_3$) δ 7.92 – 7.85 (m, 1H), 7.78 – 7.71 (m, 1H), 7.66 (td, *J* = 7.6, 1.2 Hz, 1H), 7.53 (td, *J* = 7.5, 1.0 Hz, 1H), 7.46 – 7.38 (m, 2H), 7.40 – 7.32 (m, 2H), 7.32 – 7.26 (m, 1H), 6.67 (s, 1H), 2.06 – 1.92 (m, 1H), 1.89 – 1.75 (m, 1H), 1.74 – 1.61 (m, 1H), 1.59 – 1.46 (m, 2H), 1.44 – 0.94 (m, 6H); $^{13}C\{^1H\}$ NMR (101 MHz, $CDCl_3$) δ 172.0, 164.6, 135.3, 133.4, 133.2, 132.3, 130.2, 129.6, 128.0, 127.7, 125.6, 124.1, 119.9, 107.0, 40.5, 28.7, 27.8, 25.5, 25.2, 25.0 ppm. HRMS (ESI) m/z: $[M+H]^+$: calculated for $C_{22}H_{22}NO_3^+$ 348.1594, found 348.1593.

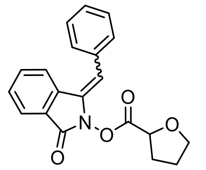

1-benzylidene-3-oxoisoindolin-2-yl tetrahydrofuran-2-carboxylate (**2f**). The title compound was prepared according to the general procedure using tetrahydro-2-furoic acid (174 mg, 1.50 mmol, 1.0 equiv.), 3-benzylidene-2-hydroxyisoindolin-1-one (356 mg, 1.50 mmol, 1.0 equiv.), dicyclohexylcarbodiimide (371 mg, 1.80 mmol, 1.2 equiv.) and DMAP (18 mg, 0.15 mmol, 0.10 equiv.) in THF (7.5 mL, 0.20 M). Purification by chromatography (Hex → Hex/AcOEt 50%) gave **2f** (453 mg, 90%) as a yellow oil. The product was isolated as a mixture of *E*/*Z* and *R*/*S* isomers and a tentative assignation is provided. ^{1}H NMR (400 MHz, $CDCl_3$) δ 7.93 – 7.84 (m, 1H), 7.79 (dt, *J* = 7.9, 0.9 Hz, 0.2H), 7.75 (d, *J* = 7.8 Hz, 0.9H), 7.71 – 7.60 (m, 1H), 7.57 – 7.50 (m, 1H), 7.47 – 7.40 (m, 2H), 7.42 – 7.33 (m, 2H), 7.36 – 7.26 (m, 1H), 6.71 (s, 0.8H), 6.56 (s, 0.2H), 4.32 (s, 0.3H), 4.00 – 3.90 (m, 0.5H), 3.89 – 3.75 (m, 2H), 2.28 – 1.98 (m, 1H), 1.96 – 1.59 (m, 2H), 1.34 – 1.10 (m, 1H); $^{13}C\{^1H\}$ NMR (101 MHz, $CDCl_3$) δ 169.9, 169.0, 138.3, 135.3, 135.1, 133.4, 133.2, 132.4, 132.3, 132.1, 130.2, 129.9, 129.7, 129.4, 128.5, 128.2,

128.0, 127.8, 125.3, 124.2, 123.7, 120.0, 119.9, 107.3, 106.0, 75.0, 74.5, 69.6, 69.5, 30.4, 29.1, 25.0 ppm. HRMS (ESI) m/z: $[M+H]^+$: calculated for $C_{20}H_{18}NO_4^+$ 336.1230, found 336.1227.

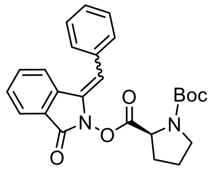

2-(1-benzylidene-3-oxoisoindolin-2-yl) 1-(tert-butyl) (S)-pyrrolidine-1,2-dicarboxylate (**2g**). The title compound was prepared according to the general procedure using *N*-Boc proline (323 mg, 1.50 mmol, 1.0 equiv.), 3-benzylidene-2-hydroxyisoindolin-1-one (356 mg, 1.50 mmol, 1.0 equiv.), dicyclohexylcarbodiimide (371 mg, 1.80 mmol, 1.2 equiv.) and DMAP (18 mg, 0.15 mmol, 0.10 equiv.) in THF (7.5 mL, 0.20 M). Purification by chromatography (Hex → Hex/AcOEt 50%) gave **2g** (476 mg, 73%) as a yellow oil in a 88/12 proportion of *E*/*Z* isomers. ^{1}H NMR (400 MHz, $CDCl_3$) δ 7.91 (d, *J* = 7.6 Hz, 0.7H), 7.88 (dt, *J* = 7.6, 1.0 Hz, 0.3H), 7.79 (dt, *J* = 7.7, 0.9 Hz, 0.2H), 7.74 (d, *J* = 7.8 Hz, 0.9H), 7.70 – 7.61 (m, 1H), 7.58 – 7.49 (m, 1H), 7.52 – 7.40 (m, 2H), 7.38 (t, *J* = 7.7 Hz, 2H), 7.33 – 7.25 (m, 1H), 6.64 (s, 0.8H), 6.55 (s, 0.1H), 4.37 – 4.11 (m, 1H), 3.48 – 3.10 (m, 2H), 1.75 – 1.23 (m, 13H); $^{13}C\{^1H\}$ NMR (101 MHz, $CDCl_3$) δ 169.6, 168.9, 165.0, 153.7, 138.3, 135.6, 135.1, 133.4, 133.2, 132.4, 130.4, 129.8, 129.4, 128.5, 128.4, 128.1, 127.8, 125.3, 124.3, 123.7, 120.0, 107.1, 106.0, 81.0, 56.7, 46.3, 46.0, 28.2 ppm. HRMS (ESI) m/z: $[M+H]^+$: calculated for $C_{25}H_{27}N_2O_5^+$ 435.1914, found 435.1918.

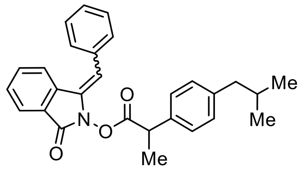

1-benzylidene-3-oxoisoindolin-2-yl 2-(4-isobutylphenyl) propanoate (**2h**). The title compound was prepared according to the general procedure using 2-(4-isobutylphenyl)propanoic acid (309 mg, 1.50 mmol, 1.0 equiv.), 3-benzylidene-2-hydroxyisoindolin-1-one (356 mg, 1.50 mmol, 1.0 equiv.), dicyclohexylcarbodiimide (371 mg, 1.80 mmol, 1.2 equiv.) and DMAP (18 mg, 0.15 mmol, 0.10 equiv.) in THF (7.5 mL, 0.20 M). Purification by chromatography (Hex → Hex/AcOEt 25%) gave **2h** (517 mg, 81%) as a yellow oil. The product was isolated as a mixture of *E*/*Z* and *R*/*S* isomers and a tentative assignation is provided. ^{1}H NMR (400 MHz, $CDCl_3$) δ 7.92 – 7.82 (m, 1H), 7.78 – 7.69 (m, 1H), 7.64 (td, *J* = 7.6, 1.2 Hz, 1H), 7.51 (td, *J* = 7.5, 1.0 Hz, 1H), 7.48 – 7.31 (m, 4H), 7.30 – 7.20 (m, 1H), 7.07 (s, 2H), 6.98 – 6.88 (m, 1H), 6.85 – 6.76 (m, 1H), 6.69 (s, 0.6H), 6.61 (s, 0.4H), 3.52 – 3.38 (m, 0.4H), 3.16 (q, *J* = 7.2 Hz, 0.6H), 2.49 – 2.32 (m, 2H), 1.82 (hept, *J* = 6.8 Hz, 1H), 1.44 (d, *J* = 6.9 Hz, 1.3H), 1.03 (d, *J* = 7.3 Hz, 1.9H), 0.88 (d, *J* = 6.6 Hz, 6H); $^{13}C\{^1H\}$ NMR (101 MHz, $CDCl_3$) δ 171.4, 171.1, 165.1, 164.3, 141.0, 140.8, 135.9, 135.6, 135.2, 133.2, 132.2, 130.2, 129.6, 129.6, 129.3, 128.1, 127.9, 127.2, 124.2, 119.9, 107.6, 106.9, 45.2, 42.4, 42.2, 30.2, 22.5, 19.0, 18.5 ppm. HRMS (ESI) m/z: $[M+H]^+$: calculated for $C_{28}H_{28}NO_3^+$ 426.2064, found 426.2069.

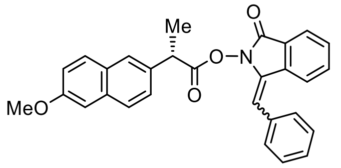

1-benzylidene-3-oxoisoindolin-2-yl (S)-2-(6-methoxynaphthalen-2-yl)propanoate (**2i**). The title compound was prepared according to the general procedure using (*S*)-2-(6-methoxynaphthalen-2-yl)propanoic acid (345 mg, 1.50 mmol, 1.0 equiv.), 3-benzylidene-2-hydroxyisoindolin-1-one (356 mg, 1.50 mmol, 1.0 equiv.), dicyclohexylcarbodiimide (371 mg, 1.80 mmol, 1.2 equiv.) and DMAP (18 mg, 0.15 mmol, 0.10 equiv.) in THF (7.5 mL, 0.20 M). Purification by chromatography (Hex → Hex/AcOEt 50%) gave **2i** (580 mg, 86%) as a yellow oil. ^{1}H NMR (400 MHz, $CDCl_3$) δ 7.95 – 7.80 (m, 1H), 7.78

– 7.60 (m, 3H), 7.59 – 7.48 (m, 2H), 7.48 – 7.30 (m, 3H), 7.30 – 7.21 (m, 2H), 7.18 – 6.95 (m, 4H), 6.86 – 6.78 (m, 0.2H), 6.70 (s, 0.4H), 6.58 (s, 0.4H), 4.28 (s, 0.2H), 3.90 (s, 2.7H), 3.75 – 3.56 (m, 0.4H), 3.40 – 3.24 (m, 0.4H), 1.83 – 1.41 (m, 2H), 1.15 (d, *J* = 7.4 Hz, 1H) ppm. HRMS (ESI) m/z: $[M+H]^+$: calculated for $C_{29}H_{24}NO_4^+$ 450.1700, found 450.1707.

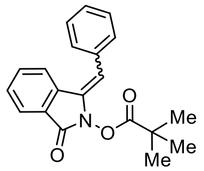

1-benzylidene-3-oxoisoindolin-2-yl pivalate (**2j**). The title compound was prepared according to the general procedure using pivalic acid (153 mg, 1.50 mmol, 1.0 equiv.), 3-benzylidene-2-hydroxyisoindolin-1-one (356 mg, 1.50 mmol, 1.0 equiv.), dicyclohexylcarbodiimide (371 mg, 1.80 mmol, 1.2 equiv.) and DMAP (18 mg, 0.15 mmol, 0.10 equiv.) in THF (7.5 mL, 0.20 M). Purification by chromatography (Hex → Hex/AcOEt 50%) gave **2j** (342 mg, 71%) as a yellow oil in a 88/12 proportion of *E*/*Z* isomers. ^{1}H NMR (400 MHz, $CDCl_3$) δ 7.88 (dt, *J* = 7.6, 1.0 Hz, 1H), 7.74 (dt, *J* = 7.9, 1.0 Hz, 1H), 7.65 (td, *J* = 7.6, 1.2 Hz, 1H), 7.52 (td, *J* = 7.5, 1.0 Hz, 1H), 7.48 – 7.40 (m, 2H), 7.40 – 7.31 (m, 2H), 7.32 – 7.23 (m, 1H), 6.67 (s, 1H), 1.47 (s, 1H), 0.95 (s, 8H); ^{13}C{^{1}H} NMR (101 MHz, $CDCl_3$) δ 174.7, 165.4, 135.6, 133.6, 133.3, 132.9, 130.2, 129.6, 128.2, 127.9, 125.5, 124.1, 120.0, 107.4, 38.2, 26.6 ppm. HRMS (ESI) m/z: $[M+H]^+$: calculated for $C_{20}H_{20}NO_3^+$ 322.1438, found 322.1433.

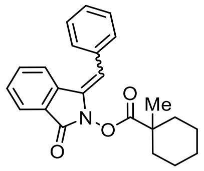

1-benzylidene-3-oxoisoindolin-2-yl 1-methylcyclohexane-1-carboxylate (**2k**). The title compound was prepared according to the general procedure using 1-methylcyclohexane-1-carboxylic acid (213 mg, 1.50 mmol, 1.0 equiv.), 3-benzylidene-2-hydroxyisoindolin-1-one (356 mg, 1.50 mmol, 1.0 equiv.), dicyclohexylcarbodiimide (371 mg, 1.80 mmol, 1.2 equiv.) and DMAP (18 mg, 0.15 mmol, 0.10 equiv.) in THF (7.5 mL, 0.20 M). Purification by chromatography (Hex → Hex/AcOEt 25%) gave **2k** (450 mg, 83%) as a yellow oil in a 84/16 proportion of *E*/*Z* isomers. ^{1}H NMR (400 MHz, $CDCl_3$) δ 7.92 – 7.83 (m, 1H), 7.75 (dt, *J* = 7.8, 0.9 Hz, 1H), 7.66 (td, *J* = 7.6, 1.2 Hz, 1H), 7.53 (td, *J* = 7.5, 1.0 Hz, 1H), 7.51 – 7.44 (m, 2H), 7.47 – 7.38 (m, 1H), 7.40 – 7.32 (m, 2H), 7.31 – 7.24 (m, 1H), 6.66 (s, 0.9H), 6.45 (s, 0.2H), 2.29 (s, 0.4H), 1.98 – 1.84 (m, 1H), 1.81 – 1.38 (m, 6H), 1.38 – 1.23 (m, 1H), 1.23 – 0.97 (m, 4H), 0.96 (s, 0.2H), 0.68 (s, 2.6H); ^{13}C{^{1}H} NMR (101 MHz, $CDCl_3$) δ 174.2, 165.8, 135.8, 133.5, 133.3, 133.1, 130.4, 129.6, 128.8, 128.3, 127.9, 125.6, 124.2, 120.0, 107.5, 42.7, 35.8, 35.2, 25.6, 22.8, 22.7 ppm. HRMS (ESI) m/z: $[M+H]^+$: calculated for $C_{23}H_{24}NO_3^+$ 362.1751, found 362.1755.

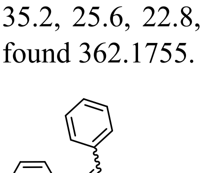

1-*benzylidene-3-oxoisoindolin-2-yl (1s,3s)-adamantane-1-carboxylate* (**2l**). The title compound was prepared according to the general procedure using adamantane-1-carboxylic acid (270 mg, 1.50 mmol, 1.0 equiv.), 3-benzylidene-2-hydroxyisoindolin-1-one (356 mg, 1.50 mmol, 1.0 equiv.), dicyclohexylcarbodiimide (371 mg, 1.80 mmol, 1.2 equiv.) and DMAP (18 mg, 0.15 mmol, 0.10 equiv.) in THF (7.5 mL, 0.20 M). Purification by chromatography (Hex → Hex/AcOEt 50%) gave **2l** (384 mg, 64%) as a yellow oil in a 87/13 proportion of *E*/*Z* isomers. ^{1}H NMR (400 MHz, $CDCl_3$) δ 7.93 – 7.83 (m, 1H), 7.78 – 7.71 (m, 1H), 7.66 (td, *J* = 7.6, 1.2 Hz, 1H), 7.53 (td, *J* = 7.5, 1.0 Hz, 1H), 7.49 – 7.40 (m, 2H), 7.43 – 7.33

(m, 2H), 7.33 – 7.26 (m, 1H), 6.66 (s, 0.9H), 6.41 (s, 0.1H), 2.22 – 2.16 (m, 0.8H), 2.16 – 2.09 (m, 0.5H), 1.92 – 1.83 (m, 3H), 1.82 – 1.76 (m, 1H), 1.69 – 1.48 (m, 11H); $^{13}C\{^1H\}$ NMR (101 MHz, $CDCl_3$) δ 173.6, 165.4, 135.6, 133.6, 133.2, 132.9, 130.3, 129.6, 128.8, 128.3, 127.7, 125.6, 124.2, 119.9, 109.3, 107.3, 40.3, 38.8, 37.9, 36.4, 36.2, 27.8, 27.7 ppm. HRMS (ESI) m/z: $[M+H]^+$: calculated for $C_{26}H_{26}NO_3^+$ 400.1907, found 400.1909.

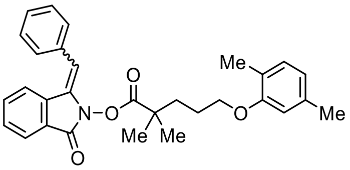

1-benzylidene-3-oxoisoindolin-2-yl 5-(2,5-dimethylphenoxy)-2,2-dimethylpentanoate (**2m**). The title compound was prepared according to the general procedure using 5-(2,5-dimethylphenoxy)-2,2-dimethylpentanoic acid (375 mg, 1.50 mmol, 1.0 equiv.), 3-benzylidene-2-hydroxyisoindolin-1-one (356 mg, 1.50 mmol, 1.0 equiv.), dicyclohexylcarbodiimide (371 mg, 1.80 mmol, 1.2 equiv.) and DMAP (18 mg, 0.15 mmol, 0.10 equiv.) in THF (7.5 mL, 0.20 M). Purification by chromatography (Hex → Hex/AcOEt 50%) gave **2m** (465 mg, 66%) as a yellow oil in a 89/11 proportion of *E*/*Z* isomers. ^1H NMR (400 MHz, $CDCl_3$) δ 7.89 (dt, *J* = 7.7, 1.0 Hz, 1H), 7.74 (dt, *J* = 7.8, 0.9 Hz, 1H), 7.66 (td, *J* = 7.6, 1.2 Hz, 1H), 7.53 (td, *J* = 7.5, 1.0 Hz, 1H), 7.49 – 7.42 (m, 2H), 7.34 (t, *J* = 7.5 Hz, 2H), 7.29 – 7.21 (m, 1H), 6.99 (d, *J* = 7.4 Hz, 1H), 6.69 – 6.61 (m, 2H), 6.58 (s, 1H), 3.81 (t, *J* = 5.8 Hz, 2H), 2.31 (s, 3H), 2.21 – 2.11 (m, 3H), 1.76 – 1.39 (m, 4H), 0.94 (s, 3H), 0.93 (s, 3H); $^{13}C\{^1H\}$ NMR (101 MHz, $CDCl_3$) δ 174.2, 165.6, 157.1, 136.6, 135.7, 133.5, 133.4, 133.0, 130.33, 130.29, 129.7, 128.3, 127.9, 125.5, 124.2, 123.6, 120.7, 120.0, 112.0, 107.5, 67.9, 41.7, 37.2, 24.68, 24.65, 24.3, 21.5, 15.9 ppm. HRMS (ESI) m/z: $[M+H]^+$: calculated for $C_{30}H_{32}NO_4^+$ 470.2326, found 470.2329.

MHAT DECARBOXYLATION OF THE REDOX-ACTIVE ESTERS

General method for the MHAT triggered decarboxylation: To a solution of the selected redox-active ester (1.0 equiv.) in THF (0.07 M) and methanol (10.0 equiv.) was added $Fe(acac)_3$ (0.10 equiv.) and the acceptor of choice (5.0 equiv.). The resulting mixture was degassed for 5 minutes with argon and, subsequently, $PhSiH_3$ (2.5 equiv.) was added dropwise. The suspension was left to stir at room temperature for 16 hours and the resulting mixture was concentrated. The corresponding product was purified by flash column chromatography on silica gel.

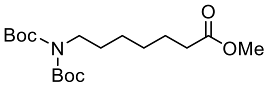

Methyl 7-((di-tert-butoxycarbonyl)amino)heptanoate (**3**). The title compound was prepared according to the general procedure using 1-benzylidene-3-oxoisoindolin-2-yl 5-(bis(*tert*-butoxycarbonyl)amino)pentanoate (107 mg, 0.20 mmol, 1.0 equiv.), $Fe(acac)_3$ (7 mg, 0.02 mmol, 0.10 equiv.), methyl acrylate (86 mg, 91 µL, 1.00 mmol, 5.0 equiv.) and $PhSiH_3$ (54 mg, 61 µL, 0.50 mmol, 2.5 equiv.) in THF (3 mL, 0.07 M) and methanol (64 mg, 81 µL, 2.00 mmol, 10.0 equiv.). Purification by chromatography (Hex → Hex/AcOEt 25%) gave **3** (67 mg, 93%) as a colorless oil; ^1H NMR (400 MHz, $CDCl_3$) δ 3.66 (s, 3H), 3.57 – 3.50 (m, 2H), 2.29 (t, *J* = 7.5 Hz, 2H), 1.68 – 1.50 (m, 4H), 1.49 (s, 18H), 1.39 – 1.21 (m, 4H).; $^{13}C\{^1H\}$ NMR (101 MHz, $CDCl_3$) δ 174.3, 152.9, 82.2, 51.6, 46.5, 34.1, 29.0, 28.2, 26.6, 25.0 ppm. HRMS (ESI) m/z: $[M+Na]^+$: calculated for $C_{18}H_{33}NNaO_6^+$ 382.2200, found 382.2191.

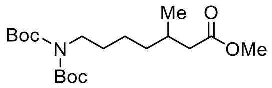

Methyl 7-((di-tert-butoxycarbonyl)amino)-3-methylheptanoate (**4**). The title compound was prepared according to the general procedure using 1-benzylidene-3-oxoisoindolin-2-yl 5-(bis(*tert*-butoxycarbonyl)amino)pentanoate (107 mg, 0.20 mmol, 1.0 equiv.), $Fe(acac)_3$ (7 mg, 0.02 mmol, 0.10 equiv.), methyl crotonate (100 mg, 106 µL, 1.00 mmol, 5.0 equiv.) and $PhSiH_3$ (54 mg, 61 µL, 0.50 mmol, 2.5 equiv.) in THF (3 mL, 0.07 M) and methanol (64 mg, 81 µL, 2.00 mmol, 10.0 equiv.). Purification by chromatography (Hex → Hex/AcOEt 25%) gave **4** (59 mg, 79%) as a colorless oil; ^{1}H NMR (400 MHz, $CDCl_3$) δ 3.65 (s, 3H), 3.53 (t, *J* = 7.5 Hz, 2H), 2.28 (dd, *J* = 14.7, 6.0 Hz, 1H), 2.10 (dd, *J* = 14.7, 8.1 Hz, 1H), 2.01 – 1.86 (m, 1H), 1.63 – 1.50 (m, 2H), 1.49 (s, 18H), 1.38 – 1.12 (m, 4H), 0.91 (d, *J* = 6.6 Hz, 3H); ^{13}C{^{1}H} NMR (101 MHz, $CDCl_3$) δ 173.8, 152.9, 82.2, 51.5, 46.5, 41.7, 36.5, 30.4, 29.3, 28.2, 24.3, 19.7 ppm. HRMS (ESI) m/z: $[M+Na]^+$: calculated for $C_{19}H_{35}NNaO_6^+$ 396.2357, found 396.2352.

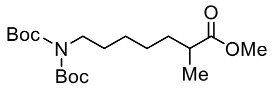

Methyl 7-((di-tert-butoxycarbonyl)amino)-2-methylheptanoate (**5**). The title compound was prepared according to the general procedure using 1-benzylidene-3-oxoisoindolin-2-yl 5-(bis(*tert*-butoxycarbonyl)amino)pentanoate (107 mg, 0.20 mmol, 1.0 equiv.), $Fe(acac)_3$ (7 mg, 0.02 mmol, 0.10 equiv.), methyl methacrylate (100 mg, 106 µL, 1.00 mmol, 5.0 equiv.) and $PhSiH_3$ (54 mg, 61 µL, 0.50 mmol, 2.5 equiv.) in THF (3 mL, 0.07 M) and methanol (64 mg, 81 µL, 2.00 mmol, 10.0 equiv.). Purification by chromatography (Hex → Hex/AcOEt 25%) gave **5** (62 mg, 83%) as a colorless oil; ^{1}H NMR (400 MHz, $CDCl_3$) δ 3.65 (s, 3H), 3.57 – 3.48 (m, 2H), 2.48 – 2.35 (m, 1H), 1.71 – 1.49 (m, 3H), 1.49 (s, 18H), 1.45 – 1.34 (m, 1H), 1.34 – 1.21 (m, 4H), 1.13 (d, *J* = 7.0 Hz, 3H); ^{13}C{^{1}H} NMR (101 MHz, $CDCl_3$) δ 177.4, 152.9, 82.2, 51.6, 46.5, 39.5, 33.9, 29.0, 28.2, 27.1, 26.9, 17.2 ppm. HRMS (ESI) m/z: $[M+Na]^+$: calculated for $C_{19}H_{35}NNaO_6^+$ 396.2357, found 396.2347.

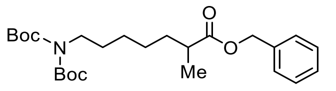

Benzyl 7-((di-tert-butoxycarbonyl)amino)-2-methylheptanoate (**6**). The title compound was prepared according to a modification of the general procedure using 1-benzylidene-3-oxoisoindolin-2-yl 5-(bis(*tert*-butoxycarbonyl)amino)pentanoate (107 mg, 0.20 mmol, 1.0 equiv.), $Fe(acac)_3$ (7 mg, 0.02 mmol, 0.10 equiv.), benzyl methacrylate (70 mg, 67 µL, 0.40 mmol, 2.0 equiv.) and $PhSiH_3$ (54 mg, 61 µL, 0.50 mmol, 2.5 equiv.) in THF (3 mL, 0.07 M) and methanol (64 mg, 81 µL, 2.00 mmol, 10.0 equiv.). Purification by chromatography (Hex → Hex/AcOEt 25%) gave **6** (65 mg, 72%) as a colorless oil; ^{1}H NMR (400 MHz, $CDCl_3$) δ 7.41 – 7.27 (m, 5H), 5.11 (s, 2H), 3.58 – 3.47 (m, 2H), 2.58 – 2.38 (m, 1H), 1.73 – 1.51 (m, 3H), 1.50 (s, 18H), 1.48 – 1.36 (m, 1H), 1.36 – 1.20 (m, 4H), 1.16 (d, *J* = 7.0 Hz, 3H); ^{13}C{^{1}H} NMR (101 MHz, $CDCl_3$) δ 176.7, 152.9, 136.4, 128.7, 128.3, 128.2, 82.2, 66.1, 46.5, 39.6, 33.8, 29.0, 28.2, 27.1, 26.9, 17.2 ppm. HRMS (ESI) m/z: $[M+Na]^+$: calculated for $C_{25}H_{39}NNaO_6^+$ 472.2670, found 472.2661.

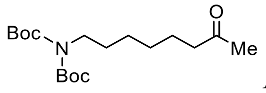

Di-tert-butyl (7-oxooctyl)carbamate (**7**). The title compound was prepared according to the general procedure using 1-benzylidene-3-oxoisoindolin-2-yl 5-(bis(*tert*-butoxycarbonyl)amino)pentanoate (107 mg, 0.20 mmol, 1.0 equiv.), $Fe(acac)_3$ (7 mg, 0.02 mmol, 0.10 equiv.), 3-buten-2-one (70 mg, 81 µL, 1.00 mmol, 5.0 equiv.) and $PhSiH_3$ (54 mg, 61 µL, 0.50 mmol, 2.5 equiv.) in THF (3 mL, 0.07 M) and methanol (64 mg, 81 µL, 2.00 mmol, 10.0 equiv.). Purification by chromatography (Hex → Hex/AcOEt 25%) gave **7** (46 mg, 67%) as a colorless oil; ^{1}H NMR (400 MHz, $CDCl_3$) δ 3.60 – 3.47 (m, 2H), 2.41 (t, *J* = 7.4 Hz, 2H), 2.12 (s,

3H), 1.66 – 1.51 (m, 4H), 1.50 (s, 18H), 1.36 – 1.22 (m, 4H); $^{13}C\{^1H\}$ NMR (101 MHz, $CDCl_3$) δ 209.3, 152.9, 82.2, 46.5, 43.8, 30.0, 29.0, 28.2, 26.8, 23.9 ppm. HRMS (ESI) m/z: $[M+Na]^+$: calculated for $C_{18}H_{33}NNaO_5^+$ 366.2251, found 366.2259.

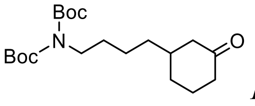

Di-tert-butyl (4-(3-oxocyclohexyl)butyl)carbamate (**8**). The title compound was prepared according to a modification of the general procedure using 1-benzylidene-3-oxoisoindolin-2-yl 5-(bis(*tert*-butoxycarbonyl)amino)pentanoate (107 mg, 0.20 mmol, 1.0 equiv.), $Fe(acac)_3$ (7 mg, 0.02 mmol, 0.10 equiv.), 2-cyclohexen-1-one (38 mg, 38 µL, 0.40 mmol, 2.0 equiv.) and $PhSiH_3$ (54 mg, 61 µL, 0.50 mmol, 2.5 equiv.) in THF (3 mL, 0.07 M) and methanol (64 mg, 81 µL, 2.00 mmol, 10.0 equiv.). Purification by chromatography (Hex → Hex/AcOEt 25%) gave **8** (39 mg, 53%) as a colorless oil; ^1H NMR (400 MHz, $CDCl_3$) δ 3.60 – 3.49 (m, 2H), 2.46 – 2.30 (m, 2H), 2.30 – 2.19 (m, 1H), 2.11 – 1.94 (m, 2H), 1.95 – 1.83 (m, 1H), 1.83 – 1.68 (m, 1H), 1.72 – 1.59 (m, 1H), 1.62 – 1.51 (m, 2H), 1.50 (s, 18H), 1.43 – 1.23 (m, 5H); $^{13}C\{^1H\}$ NMR (101 MHz, $CDCl_3$) δ 212.0, 152.9, 82.3, 48.3, 46.4, 41.6, 39.2, 36.4, 31.3, 29.2, 28.2, 25.4, 24.1 ppm. HRMS (ESI) m/z: $[M+Na]^+$: calculated for $C_{20}H_{35}NNaO_5^+$ 392.2407, found 392.2403.

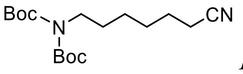

Di-tert-butyl (6-cyanohexyl)carbamate (**9**). The title compound was prepared according to the general procedure using 1-benzylidene-3-oxoisoindolin-2-yl 5-(bis(*tert*-butoxycarbonyl)amino)pentanoate (107 mg, 0.20 mmol, 1.0 equiv.), $Fe(acac)_3$ (7 mg, 0.02 mmol, 0.10 equiv.), acrylonitrile (53 mg, 65 µL, 1.00 mmol, 5.0 equiv.) and $PhSiH_3$ (54 mg, 61 µL, 0.50 mmol, 2.5 equiv.) in THF (3 mL, 0.07 M) and methanol (64 mg, 81 µL, 2.00 mmol, 10.0 equiv.). Purification by chromatography (Hex → Hex/AcOEt 25%) gave **9** (59 mg, 90%) as a colorless oil; ^1H NMR (400 MHz, $CDCl_3$) δ 3.60 – 3.48 (m, 2H), 2.32 (t, J = 7.1 Hz, 2H), 1.71 – 1.59 (m, 2H), 1.62 – 1.51 (m, 2H), 1.49 (s, 18H), 1.49 – 1.40 (m, 2H), 1.38 – 1.25 (m, 2H); $^{13}C\{^1H\}$ NMR (101 MHz, $CDCl_3$) δ 152.9, 119.8, 82.3, 46.3, 28.8, 28.5, 28.2, 26.1, 25.4, 17.2 ppm. HRMS (ESI) m/z: $[M+Na]^+$: calculated for $C_{17}H_{30}N_2NaO_4^+$ 349.2098, found 349.2103.

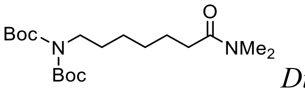

Di-tert-butyl (7-(dimethylamino)-7-oxoheptyl)carbamate (**10**). The title compound was prepared according to a modification of the general procedure using 1-benzylidene-3-oxoisoindolin-2-yl 5-(bis(*tert*-butoxycarbonyl)amino)pentanoate (107 mg, 0.20 mmol, 1.0 equiv.), $Fe(acac)_3$ (7 mg, 0.02 mmol, 0.10 equiv.), *N,N*-dimethylacrylamide (40 mg, 42 µL, 0.40 mmol, 2.0 equiv.) and $PhSiH_3$ (54 mg, 61 µL, 0.50 mmol, 2.5 equiv.) in THF (3 mL, 0.07 M) and methanol (64 mg, 81 µL, 2.00 mmol, 10.0 equiv.). Purification by chromatography (Hex → AcOEt) gave **10** (64 mg, 86%) as a colorless oil; ^1H NMR (400 MHz, $CDCl_3$) δ 3.59 – 3.47 (m, 2H), 2.99 (s, 3H), 2.93 (s, 3H), 2.34 – 2.24 (m, 2H), 1.68 – 1.50 (m, 4H), 1.49 (s, 18H), 1.41 – 1.22 (m, 4H); $^{13}C\{^1H\}$ NMR (101 MHz, $CDCl_3$) δ 173.2, 152.9, 82.2, 46.5, 37.4, 35.5, 33.4, 29.4, 29.1, 28.2, 26.8, 25.3 ppm. HRMS (ESI) m/z: $[M+Na]^+$: calculated for $C_{19}H_{36}N_2NaO_5^+$ 395.2516, found 395.2523.

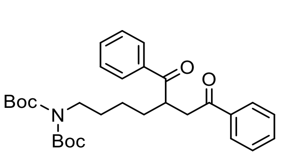

Di-tert-butyl (5-benzoyl-7-oxo-7-phenylheptyl)carbamate (**11**). The title compound was prepared according to a modification of the general procedure using 1-benzylidene-3-oxoisoindolin-2-yl 5-(bis(*tert*-butoxycarbonyl)amino)pentanoate (107 mg, 0.20 mmol, 1.0 equiv.), $Fe(acac)_3$ (7 mg, 0.02 mmol, 0.10 equiv.), *trans*-1,4-diphenyl-2-butene-1,4-dione (95 mg, 0.40 mmol, 2.0 equiv.) and $PhSiH_3$ (54 mg, 61 µL, 0.50 mmol, 2.5 equiv.) in THF

(3 mL, 0.07 M) and methanol (64 mg, 81 µL, 2.00 mmol, 10.0 equiv.). Purification by chromatography (Hex → Hex/AcOEt 25%) gave **11** (75 mg, 74%) as a white solid; ^{1}H NMR (400 MHz, $CDCl_3$) δ 8.09 – 8.02 (m, 2H), 8.00 – 7.93 (m, 2H), 7.61 – 7.51 (m, 2H), 7.53 – 7.40 (m, 4H), 4.21 – 4.08 (m, 1H), 3.72 (dd, *J* = 18.0, 9.2 Hz, 1H), 3.58 – 3.45 (m, 2H), 3.16 (dd, *J* = 18.0, 4.1 Hz, 1H), 1.86 – 1.70 (m, 1H), 1.65 – 1.48 (m, 3H), 1.47 (s, 18H), 1.40 – 1.23 (m, 2H); $^{13}C\{^1H\}$ NMR (101 MHz, $CDCl_3$) δ 203.3, 198.7, 152.9, 136.9, 136.7, 133.3, 133.1, 128.8, 128.7, 128.6, 128.3, 82.3, 46.2, 41.3, 40.8, 32.3, 29.2, 28.2, 24.7 ppm. HRMS (ESI) m/z: $[M+Na]^+$: calculated for $C_{20}H_{35}NNaO_5^+$ 532.2670, found 532.2673.

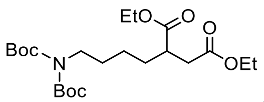

Diethyl 2-(4-((di-tert-butoxycarbonyl)amino)butyl)succinate (**12**). The title compound was prepared according to a modification of the general procedure using 1-benzylidene-3-oxoisoindolin-2-yl 5-(bis(*tert*-butoxycarbonyl)amino)pentanoate (107 mg, 0.20 mmol, 1.0 equiv.), $Fe(acac)_3$ (7 mg, 0.02 mmol, 0.10 equiv.), diethyl maleate (69 mg, 65 µL, 0.40 mmol, 2.0 equiv.) and $PhSiH_3$ (54 mg, 61 µL, 0.50 mmol, 2.5 equiv.) in THF (3 mL, 0.07 M) and methanol (64 mg, 81 µL, 2.00 mmol, 10.0 equiv.). Purification by chromatography (Hex → Hex/AcOEt 25%) gave **12** (81 mg, 91%) as a colorless oil; ^{1}H NMR (400 MHz, $CDCl_3$) δ 4.19 – 4.07 (m, 4H), 3.57 – 3.49 (m, 2H), 2.86 – 2.73 (m, 1H), 2.68 (dd, *J* = 16.4, 9.3 Hz, 1H), 2.40 (dd, *J* = 16.3, 5.1 Hz, 1H), 1.72 – 1.57 (m, 2H), 1.61 – 1.49 (m, 2H), 1.49 (s, 18H), 1.36 – 1.25 (m, 2H), 1.27 – 1.19 (m, 6H); $^{13}C\{^1H\}$ NMR (101 MHz, $CDCl_3$) δ 174.9, 172.0, 152.8, 82.3, 60.7, 46.2, 41.4, 36.2, 31.8, 29.0, 28.2, 24.3, 14.3, 14.3 ppm. HRMS (ESI) m/z: $[M+Na]^+$: calculated for $C_{22}H_{39}NNaO_8^+$ 468.2568, found 468.2573.

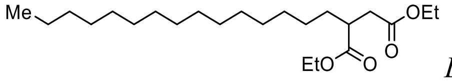

Diethyl 2-pentadecylsuccinate (**13**). The title compound was prepared according to a modification of the general procedure using 1-benzylidene-3-oxoisoindolin-2-yl palmitate (95 mg, 0.20 mmol, 1.0 equiv.), $Fe(acac)_3$ (7 mg, 0.02 mmol, 0.10 equiv.), diethyl maleate (69 mg, 65 µL, 0.40 mmol, 2.0 equiv.) and $PhSiH_3$ (54 mg, 61 µL, 0.50 mmol, 2.5 equiv.) in THF (3 mL, 0.07 M) and methanol (64 mg, 81 µL, 2.00 mmol, 10.0 equiv.). Purification by chromatography (Hex → Hex/AcOEt 25%) gave **13** (62 mg, 80%) as a colorless oil; ^{1}H NMR (400 MHz, $CDCl_3$) δ 4.21 – 4.07 (m, 4H), 2.86 – 2.75 (m, 1H), 2.68 (dd, *J* = 16.3, 9.4 Hz, 1H), 2.40 (dd, *J* = 16.3, 5.1 Hz, 1H), 1.69 – 1.56 (m, 1H), 1.55 – 1.42 (m, 1H), 1.33 – 1.18 (m, 32H), 0.92 – 0.82 (m, 3H); $^{13}C\{^1H\}$ NMR (101 MHz, $CDCl_3$) δ 175.2, 172.2, 60.7, 60.6, 41.4, 36.3, 32.1, 32.1, 29.82, 29.79, 29.76, 29.7, 29.6, 29.53, 29.50, 27.0, 22.8, 14.4, 14.3, 14.3 ppm. HRMS (ESI) m/z: $[M+H]^+$: calculated for $C_{23}H_{45}O_4^+$ 385.3312, found 385.3320.

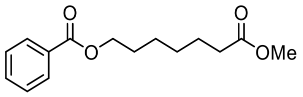

7-methoxy-7-oxoheptyl benzoate (**14**). The title compound was prepared according to the general procedure using 5-((1-benzylidene-3-oxoisoindolin-2-yl)oxy)-5-oxopentyl benzoate (88 mg, 0.20 mmol, 1.0 equiv.), $Fe(acac)_3$ (7 mg, 0.02 mmol, 0.10 equiv.), methyl acrylate (86 mg, 91 µL, 1.00 mmol, 5.0 equiv.) and $PhSiH_3$ (54 mg, 61 µL, 0.50 mmol, 2.5 equiv.) in THF (3 mL, 0.07 M) and methanol (64 mg, 81 µL, 2.00 mmol, 10.0 equiv.). Purification by chromatography (Hex → Hex/AcOEt 50%) gave **14** (45 mg, 85%) as a colorless oil. ^{1}H NMR (500 MHz, $CDCl_3$) δ 8.07 – 8.01 (m, 2H), 7.59 – 7.52 (m, 1H), 7.47 – 7.40 (m, 2H), 4.31 (t, *J* = 6.6 Hz, 2H), 3.66 (s, 3H), 2.32 (t, *J* = 7.5 Hz, 2H), 1.83 – 1.73 (m, 2H), 1.72 – 1.61 (m, 2H), 1.52 – 1.42 (m, 2H), 1.44 – 1.34 (m, 2H); $^{13}C\{^1H\}$ NMR (126 MHz, $CDCl_3$) δ 174.3, 166.8, 133.0, 130.6, 129.7, 128.5, 65.0, 51.6, 34.1, 28.9, 28.7, 25.9, 24.9 ppm. HRMS (ESI) m/z: $[M+H]^+$: calculated for $C_{15}H_{21}O_4^+$ 265.1434, found 265.1440.

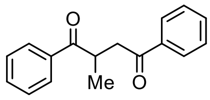
2-methyl-1,4-diphenylbutane-1,4-dione (**15**). The title compound was prepared according to a modification of the general procedure using 1-benzylidene-3-oxoisoindolin-2-yl acetate (56 mg, 0.20 mmol, 1.0 equiv.), $Fe(acac)_3$ (7 mg, 0.02 mmol, 0.10 equiv.), (*E*)-1,4-diphenylbut-2-ene-1,4-dione (95 mg, 0.40 mmol, 2.0 equiv.) and $PhSiH_3$ (54 mg, 61 µL, 0.50 mmol, 2.5 equiv.) in THF (3 mL, 0.07 M) and methanol (64 mg, 81 µL, 2.00 mmol, 10.0 equiv.). Purification by chromatography (Hex → Hex/AcOEt 25%) gave **15** (41 mg, 81%) as a colorless oil. Spectral data were identical to those previously reported.[3] ^{1}H NMR (400 MHz, $CDCl_3$) δ 8.10 – 8.03 (m, 2H), 8.03 – 7.96 (m, 2H), 7.62 – 7.52 (m, 2H), 7.54 – 7.41 (m, 4H), 4.26 – 4.12 (m, 1H), 3.73 (dd, *J* = 18.0, 8.4 Hz, 1H), 3.12 (dd, *J* = 18.0, 4.9 Hz, 1H), 1.29 (d, *J* = 7.2 Hz, 3H); ^{13}C{^{1}H} NMR (101 MHz, $CDCl_3$) δ 203.6, 198.6, 136.8, 136.2, 133.3, 133.1, 128.8, 128.7, 128.7, 128.2, 42.5, 36.4, 18.1 ppm. HRMS (ESI) m/z: [M+H]$^+$: calculated for $C_{17}H_{17}O_2^+$ 253.1223, found 253.1227.

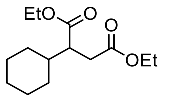
Diethyl 2-cyclohexylsuccinate (**16**). The title compound was prepared according to a modification of the general procedure using 1-benzylidene-3-oxoisoindolin-2-yl cyclohexanecarboxylate (69 mg, 0.20 mmol, 1.0 equiv.), $Fe(acac)_3$ (7 mg, 0.02 mmol, 0.10 equiv.), diethyl maleate (69 mg, 65 µL, 0.40 mmol, 2.0 equiv.) and $PhSiH_3$ (54 mg, 61 µL, 0.50 mmol, 2.5 equiv.) in THF (3 mL, 0.07 M) and methanol (64 mg, 81 µL, 2.00 mmol, 10.0 equiv.). Purification by chromatography (Hex → Hex/AcOEt 25%) gave **16** (44 mg, 85%) as a colorless oil. Spectral data were identical to those previously reported.[4] ^{1}H NMR (400 MHz, $CDCl_3$) δ 4.23 – 4.02 (m, 4H), 2.76 – 2.62 (m, 2H), 2.51 – 2.35 (m, 1H), 1.79 – 1.68 (m, 2H), 1.70 – 1.53 (m, 4H), 1.29 – 1.20 (m, 7H), 1.21 – 0.94 (m, 4H); ^{13}C{^{1}H} NMR (101 MHz, $CDCl_3$) δ 174.6, 172.7, 60.7, 60.5, 47.2, 40.1, 33.6, 30.7, 30.2, 26.5, 26.3, 14.4, 14.3 ppm. HRMS (ESI) m/z: [M+H]$^+$: calculated for $C_{14}H_{25}O_4^+$ 257.1747, found 257.1747.

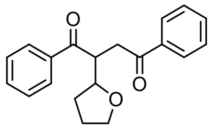
1,4-diphenyl-2-(tetrahydrofuran-2-yl)butane-1,4-dione (**17**). The title compound was prepared according to a modification of the general procedure using 1-benzylidene-3-oxoisoindolin-2-yl tetrahydrofuran-2-carboxylate (67 mg, 0.20 mmol, 1.0 equiv.), $Fe(acac)_3$ (7 mg, 0.02 mmol, 0.10 equiv.), (*E*)-1,4-diphenylbut-2-ene-1,4-dione (95 mg, 0.40 mmol, 2.0 equiv.) and $PhSiH_3$ (54 mg, 61 µL, 0.50 mmol, 2.5 equiv.) in THF (3 mL, 0.07 M) and methanol (64 mg, 81 µL, 2.00 mmol, 10.0 equiv.). Purification by chromatography (Hex → Hex/AcOEt 25%) gave **17** (57 mg, 92%) as a colorless oil. A 60/40 mixture of stereoisomers was obtained, spectroscopic data is reported for the major isomer. ^{1}H NMR (400 MHz, $CDCl_3$) δ 8.13 – 8.08 (m, 2H), 7.99 – 7.91 (m, 2H), 7.61 – 7.51 (m, 2H), 7.53 – 7.44 (m, 2H), 7.48 – 7.40 (m, 2H), 4.40 (ddd, *J* = 10.2, 7.1, 3.2 Hz, 1H), 4.22 – 4.12 (m, 1H), 3.91 – 3.83 (m, 1H), 3.80 (dd, *J* = 18.0, 10.1 Hz, 1H), 3.74 – 3.64 (m, 1H), 3.21 (dd, *J* = 18.0, 3.2 Hz, 1H), 2.01 – 1.79 (m, 3H), 1.78 – 1.64 (m, 1H); ^{13}C{^{1}H} NMR (101 MHz, $CDCl_3$) δ 202.0, 198.3, 137.9, 136.6, 133.4, 133.0, 128.8, 128.7, 128.7, 128.2, 79.9, 68.4, 46.1, 37.8, 29.0, 25.8 ppm. HRMS (ESI) m/z: [M+H]$^+$: calculated for $C_{20}H_{21}O_3^+$ 309.1485, found 309.1487.

[3] A. Voituriez, L. E. Zimmer and A. B. Charrette, *J. Org. Chem.*, **2010**, *75*, 1244–1250.
[4] S. Rohe, A. O. Morris, T. McCallum and L. Barriault, *Angew. Chem., Int. Ed.*, **2018**, *57*, 15664–15669.

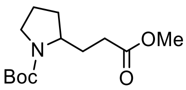

tert-butyl 2-(3-methoxy-3-oxopropyl)pyrrolidine-1-carboxylate (**18**). The title compound was prepared according to the general procedure using 2-(1-benzylidene-3-oxoisoindolin-2-yl) 1-(*tert*-butyl) pyrrolidine-1,2-dicarboxylate (87 mg, 0.20 mmol, 1.0 equiv.), $Fe(acac)_3$ (7 mg, 0.02 mmol, 0.10 equiv.), methyl acrylate (86 mg, 91 µL, 1.00 mmol, 5.0 equiv.) and $PhSiH_3$ (54 mg, 61 µL, 0.50 mmol, 2.5 equiv.) in THF (3 mL, 0.07 M) and methanol (64 mg, 81 µL, 2.00 mmol, 10.0 equiv.). Purification by chromatography (Hex → Hex/AcOEt 50%) gave **18** (46 mg, 90%) as a colorless oil. Spectral data were identical to those previously reported.[5] ^{1}H NMR (400 MHz, $CDCl_3$) δ 3.86 – 3.75 (m, 1H), 3.66 (s, 3H), 3.46 – 3.33 (m, 1H), 3.32 – 3.21 (m, 1H), 2.32 (t, *J* = 7.8 Hz, 2H), 2.05 – 1.75 (m, 4H), 1.74 – 1.56 (m, 2H), 1.45 (s, 9H); $^{13}C\{^1H\}$ NMR (101 MHz, $CDCl_3$) δ 174.0, 154.9, 79.4, 56.7, 51.7, 46.4, 31.3, 30.6, 29.9, 28.7, 23.5 ppm. HRMS (ESI) m/z: $[M+H]^+$: calculated for $C_{13}H_{24}NO_4^+$ 258.1700, found 258.1704.

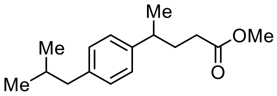

Methyl 4-(4-isobutylphenyl)pentanoate (**19**). The title compound was prepared according to the general procedure using 1-benzylidene-3-oxoisoindolin-2-yl 2-(4-isobutylphenyl)propanoate (85 mg, 0.20 mmol, 1.0 equiv.), $Fe(acac)_3$ (7 mg, 0.02 mmol, 0.10 equiv.), methyl acrylate (86 mg, 91 µL, 1.00 mmol, 5.0 equiv.) and $PhSiH_3$ (54 mg, 61 µL, 0.50 mmol, 2.5 equiv.) in THF (3 mL, 0.07 M) and methanol (64 mg, 81 µL, 2.00 mmol, 10.0 equiv.). Purification by chromatography (Hex → Hex/AcOEt 25%) gave **19** (29 mg, 59%) as a colorless oil; ^{1}H NMR (400 MHz, $CDCl_3$) δ 7.07 (s, 4H), 3.62 (s, 3H), 2.77 – 2.59 (m, 1H), 2.43 (d, *J* = 7.1 Hz, 2H), 2.30 – 2.08 (m, 2H), 2.02 – 1.73 (m, 3H), 1.26 (d, *J* = 7.0 Hz, 3H), 0.90 (d, *J* = 6.6 Hz, 6H); $^{13}C\{^1H\}$ NMR (101 MHz, $CDCl_3$) δ 174.4, 143.5, 139.6, 129.3, 126.8, 51.6, 45.2, 39.2, 33.4, 32.5, 30.4, 22.6, 22.3 ppm. HRMS (ESI) m/z: $[M+Na]^+$: calculated for $C_{16}H_{24}NaO_2^+$ 271.1669, found 271.1669.

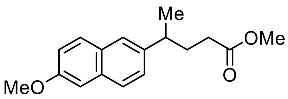

Methyl (R)-4-(6-methoxynaphthalen-2-yl)pentanoate (**20**). The title compound was prepared according to the general procedure using 1-benzylidene-3-oxoisoindolin-2-yl (*S*)-2-(6-methoxynaphthalen-2-yl)propanoate (90 mg, 0.20 mmol, 1.0 equiv.), $Fe(acac)_3$ (7 mg, 0.02 mmol, 0.10 equiv.), methyl acrylate (86 mg, 91 µL, 1.00 mmol, 5.0 equiv.) and $PhSiH_3$ (54 mg, 61 µL, 0.50 mmol, 2.5 equiv.) in THF (3 mL, 0.07 M) and methanol (64 mg, 81 µL, 2.00 mmol, 10.0 equiv.). Purification by chromatography (Hex → Hex/AcOEt 25%) gave **20** (23 mg, 43%) as a colorless oil; ^{1}H NMR (400 MHz, $CDCl_3$) δ 7.69 (dd, *J* = 8.5, 3.0 Hz, 2H), 7.53 (d, *J* = 1.7 Hz, 1H), 7.30 (dd, *J* = 8.5, 1.8 Hz, 1H), 7.17 – 7.09 (m, 2H), 3.91 (s, 3H), 3.61 (s, 3H), 2.95 – 2.76 (m, 1H), 2.32 – 2.13 (m, 2H), 2.08 – 1.89 (m, 2H), 1.34 (d, *J* = 6.9 Hz, 3H); $^{13}C\{^1H\}$ NMR (101 MHz, $CDCl_3$) δ 174.3, 157.4, 141.4, 133.4, 129.2, 127.2, 126.2, 125.4, 118.9, 105.7, 55.4, 51.6, 39.5, 33.3, 32.5, 22.4 ppm. HRMS (ESI) m/z: $[M+H]^+$: calculated for $C_{17}H_{21}O_3^+$ 273.1485, found 273.1485.

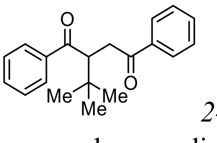

2-(tert-butyl)-1,4-diphenylbutane-1,4-dione (**21**). The title compound was prepared according to a modification of the general procedure using 1-benzylidene-3-oxoisoindolin-2-yl pivalate (64 mg, 0.20 mmol, 1.0 equiv.), $Fe(acac)_3$ (7 mg, 0.02 mmol, 0.10 equiv.), (*E*)-1,4-diphenylbut-2-ene-1,4-dione (95 mg, 0.40 mmol, 2.0 equiv.) and $PhSiH_3$ (54 mg, 61 µL, 0.50 mmol, 2.5 equiv.) in THF (3 mL, 0.07 M) and methanol (64 mg, 81 µL, 2.00 mmol,

---

[5] R. K. Dieter, K. Lu and S. E. Velu, *J. Org. Chem.*, **2000**, *65*, 8715–8724.

10.0 equiv.). Purification by chromatography (Hex → Hex/AcOEt 25%) gave **21** (50 mg, 85%) as a white solid; ^{1}H NMR (400 MHz, $CDCl_3$) δ 8.14 – 8.09 (m, 2H), 7.99 – 7.92 (m, 2H), 7.58 – 7.49 (m, 2H), 7.52 – 7.43 (m, 2H), 7.47 – 7.38 (m, 2H), 4.05 (dd, *J* = 11.0, 2.4 Hz, 1H), 3.88 (dd, *J* = 17.9, 11.0 Hz, 1H), 3.23 (dd, *J* = 17.9, 2.4 Hz, 1H), 0.98 (s, 9H); ^{13}C{^{1}H} NMR (101 MHz, $CDCl_3$) δ 204.4, 199.5, 140.0, 136.8, 133.3, 132.5, 128.7, 128.6, 128.6, 128.2, 49.4, 39.0, 33.6, 28.8 ppm. HRMS (ESI) m/z: [M+H]$^+$: calculated for $C_{20}H_{23}O_2^+$ 295.1693, found 295.1686.

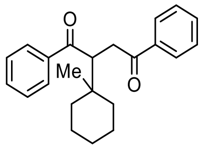

2-(1-methylcyclohexyl)-1,4-diphenylbutane-1,4-dione (**22**). The title compound was prepared according to a modification of the general procedure using 1-benzylidene-3-oxoisoindolin-2-yl 1-methylcyclohexane-1-carboxylate (72 mg, 0.20 mmol, 1.0 equiv.), $Fe(acac)_3$ (7 mg, 0.02 mmol, 0.10 equiv.), (*E*)-1,4-diphenylbut-2-ene-1,4-dione (95 mg, 0.40 mmol, 2.0 equiv.) and $PhSiH_3$ (54 mg, 61 µL, 0.50 mmol, 2.5 equiv.) in THF (3 mL, 0.07 M) and methanol (64 mg, 81 µL, 2.00 mmol, 10.0 equiv.). Purification by chromatography (Hex → Hex/AcOEt 25%) gave **22** (62 mg, 92%) as a white solid. ^{1}H NMR (400 MHz, $CDCl_3$) δ 8.16 – 8.09 (m, 2H), 8.00 – 7.92 (m, 2H), 7.58 – 7.49 (m, 2H), 7.52 – 7.43 (m, 2H), 7.47 – 7.38 (m, 2H), 4.11 (dd, *J* = 10.9, 2.4 Hz, 1H), 3.87 (dd, *J* = 17.9, 10.9 Hz, 1H), 3.24 (dd, *J* = 18.0, 2.5 Hz, 1H), 1.59 – 1.45 (m, 3H), 1.49 – 1.35 (m, 2H), 1.38 – 1.24 (m, 3H), 1.26 – 1.08 (m, 2H), 1.04 (s, 3H); ^{13}C{^{1}H} NMR (101 MHz, $CDCl_3$) δ 204.9, 199.7, 140.5, 136.8, 133.3, 132.4, 128.6, 128.5, 128.2, 50.2, 38.0, 37.2, 36.7, 26.1, 22.0, 21.8, 20.3 ppm. HRMS (ESI) m/z: [M+H]$^+$: calculated for $C_{23}H_{27}O_2^+$ 335.2006, found 335.2001.

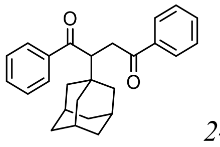

2-((3R,5R,7R)-adamantan-1-yl)-1,4-diphenylbutane-1,4-dione (**23**). The title compound was prepared according to a modification of the general procedure using 1-benzylidene-3-oxoisoindolin-2-yl (1*S*,3*S*)-adamantane-1-carboxylate (80 mg, 0.20 mmol, 1.0 equiv.), $Fe(acac)_3$ (7 mg, 0.02 mmol, 0.10 equiv.), (*E*)-1,4-diphenylbut-2-ene-1,4-dione (95 mg, 0.40 mmol, 2.0 equiv.) and $PhSiH_3$ (54 mg, 61 µL, 0.50 mmol, 2.5 equiv.) in THF (3 mL, 0.07 M) and methanol (64 mg, 81 µL, 2.00 mmol, 10.0 equiv.). Purification by chromatography (Hex → Hex/AcOEt 25%) gave **23** (65 mg, 87%) as a colorless oil; ^{1}H NMR (400 MHz, $CDCl_3$) δ 8.18 – 8.10 (m, 2H), 8.00 – 7.92 (m, 2H), 7.58 – 7.51 (m, 2H), 7.53 – 7.44 (m, 2H), 7.47 – 7.38 (m, 2H), 3.94 (dd, *J* = 11.0, 2.0 Hz, 1H), 3.84 (dd, *J* = 17.5, 11.0 Hz, 1H), 3.27 (dd, *J* = 17.6, 2.1 Hz, 1H), 1.99 – 1.89 (m, 3H), 1.78 – 1.56 (m, 9H), 1.56 – 1.46 (m, 3H); ^{13}C{^{1}H} NMR (101 MHz, $CDCl_3$) δ 204.1, 199.7, 140.1, 136.8, 133.2, 132.5, 128.7, 128.6, 128.5, 128.2, 50.5, 40.9, 37.3, 36.9, 35.8, 28.7 ppm. HRMS (ESI) m/z: [M+H]$^+$: calculated for $C_{26}H_{29}O_2^+$ 373.2162, found 373.2178.

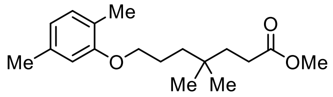

Methyl 7-(2,5-dimethylphenoxy)-4,4-dimethylheptanoate (**24**). The title compound was prepared according to the general procedure using 1-benzylidene-3-oxoisoindolin-2-yl 5-(2,5-dimethylphenoxy)-2,2-dimethylpentanoate (94 mg, 0.20 mmol, 1.0 equiv.), $Fe(acac)_3$ (7 mg, 0.02 mmol, 0.10 equiv.), methyl acrylate (86 mg, 91 µL, 1.00 mmol, 5.0 equiv.) and $PhSiH_3$ (54 mg, 61 µL, 0.50 mmol, 2.5 equiv.) in THF (3 mL, 0.07 M) and methanol (64 mg, 81 µL, 2.00 mmol, 10.0 equiv.). Purification by chromatography (Hex → Hex/AcOEt 25%) gave **24** (49 mg, 83%) as a colorless oil; ^{1}H NMR (400 MHz, $CDCl_3$) δ 7.01 (d, *J* = 7.5 Hz, 1H), 6.69 – 6.63 (m, 1H), 6.62 (s, 1H), 3.91 (t, *J* = 6.4 Hz, 2H), 3.67 (s, 3H), 2.34 – 2.25 (m, 5H), 2.18 (s, 3H), 1.82 – 1.69 (m, 2H), 1.65 – 1.55 (m, 2H), 1.43 – 1.32 (m, 2H), 0.91 (s, 6H); ^{13}C{^{1}H}

NMR (101 MHz, $CDCl_3$) δ 175.0, 157.2, 136.6, 130.4, 123.7, 120.8, 112.1, 68.5, 51.7, 38.0, 36.5, 32.4, 29.5, 26.9, 24.4, 21.6, 15.9 ppm. HRMS (ESI) m/z: $[M+H]^+$: calculated for $C_{18}H_{29}O_3^+$ 293.2111, found 293.2110.

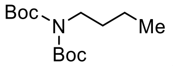

Di-tert-butyl butylcarbamate (**25**). The title compound was prepared according to a modification of the general procedure using 1-benzylidene-3-oxoisoindolin-2-yl 5-(bis(*tert*-butoxycarbonyl)amino)pentanoate (215 mg, 0.40 mmol, 1.0 equiv.), $Fe(acac)_3$ (141 mg, 0.40 mmol, 1.0 equiv.), and $PhSiH_3$ (108 mg, 123 µL, 1.00 mmol, 2.5 equiv.) in THF (6 mL, 0.07 M) and methanol (128 mg, 162 µL, 4.00 mmol, 10.0 equiv.). Purification by chromatography (Hex → Hex/AcOEt 25%) gave **25** (100 mg, 91%) as a colorless oil; ^{1}H NMR (400 MHz, $CDCl_3$) δ 3.59 – 3.48 (m, 2H), 1.58 – 1.50 (m, 2H), 1.49 (s, 18H), 1.36 – 1.23 (m, 2H), 0.90 (t, J = 7.4 Hz, 3H); $^{13}C\{^1H\}$ NMR (101 MHz, $CDCl_3$) δ 152.9, 82.1, 46.4, 31.3, 28.2, 20.1, 13.9 ppm. HRMS (ESI) m/z: $[M+H]^+$: calculated for $C_{14}H_{27}NNaO_4^+$ 296.1832, found 296.1839.

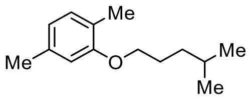

1,4-dimethyl-2-((4-methylpentyl)oxy)benzene (**26**).

Method A: The title compound was prepared according to a modification of the general procedure using 1-benzylidene-3-oxoisoindolin-2-yl 5-(2,5-dimethylphenoxy)-2,2-dimethylpentanoate (188 mg, 0.40 mmol, 1.0 equiv.), $Fe(acac)_3$ (141 mg, 0.40 mmol, 1.0 equiv.), and $PhSiH_3$ (108 mg, 123 µL, 1.00 mmol, 2.5 equiv.) in THF (6 mL, 0.07 M) and methanol (128 mg, 162 µL, 4.00 mmol, 10.0 equiv.). Purification by chromatography (Hex → Hex/AcOEt 25%) gave **26** (71 mg, 86%) as a colorless oil.

Method B: The title compound was prepared according to a modification of the general procedure using 1-benzylidene-3-oxoisoindolin-2-yl 5-(2,5-dimethylphenoxy)-2,2-dimethylpentanoate (188 mg, 0.40 mmol, 1.0 equiv.), $Fe(acac)_3$ (14 mg, 0.04 mmol, 0.1 equiv.), thiophenol (4 mg, 4 µL, 0.04 mmol, 0.1 equiv.) and $PhSiH_3$ (108 mg, 123 µL, 1.00 mmol, 2.5 equiv.) in THF (6 mL, 0.07 M) and methanol (128 mg, 162 µL, 4.00 mmol, 10.0 equiv.). Purification by chromatography (Hex → Hex/AcOEt 25%) gave **26** (69 mg, 84%) as a colorless oil.

^{1}H NMR (400 MHz, $CDCl_3$) δ 7.03 (d, J = 7.5 Hz, 1H), 6.68 (d, J = 7.8 Hz, 1H), 6.66 (s, 1H), 3.95 (t, J = 6.5 Hz, 2H), 2.34 (s, 3H), 2.21 (s, 3H), 1.89 – 1.77 (m, 2H), 1.72 – 1.59 (m, 1H), 1.44 – 1.34 (m, 2H), 0.96 (d, J = 6.6 Hz, 6H); $^{13}C\{^1H\}$ NMR (101 MHz, $CDCl_3$) δ 157.3, 136.6, 130.4, 123.8, 120.7, 112.1, 68.3, 35.5, 27.9, 27.4, 22.7, 21.6, 15.9 ppm. HRMS (ESI) m/z: $[M+H]^+$: calculated for $C_{14}H_{23}O^+$ 207.1743, found 207.1749.

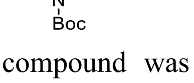

tert-butyl (tert-butoxycarbonyl)(4-hydroxybutyl)carbamate (**27**).[6] The title compound was prepared according to a modification of the general procedure using 1-benzylidene-3-oxoisoindolin-2-yl 5-(bis(*tert*-butoxycarbonyl)amino)pentanoate (150 mg, 0.28 mmol, 1.0 equiv.), $Fe(acac)_3$ (11 mg, 0.03 mmol, 0.10 equiv.), $NaHCO_3$ (47 mg, 0.56 mmol, 2.0 equiv.), methyl 4-nitrobenzenesulfonate (91 mg, 0.42 mmol, 1.5 equiv.), and $PhSiH_3$ (76 mg, 86 µL, 0.70 mmol, 2.5 equiv.) in THF (4 mL, 0.07 M) and methanol (90 mg, 114 µL, 2.80 mmol, 10.0 equiv.). Purification by chromatography (Hex → Hex/AcOEt 50%) gave **27** (50 mg, 62%) as a colorless oil. Spectral data were identical to those previously reported.[7] ^{1}H NMR (400 MHz, $CDCl_3$) δ 3.64 (t, J = 6.3 Hz, 2H), 3.60 – 3.55 (m, 2H), 1.68 – 1.59 (m, 2H), 1.59 – 1.52 (m, 2H),

[6] A. Bhunia, K. Bergander, C. G. Daniliuc and A. Studer, *Angew. Chem., Int. Ed.*, **2021**, *60*, 8313–8320.
[7] N. P. McLaughlin and P. Evans, *J. Org. Chem.*, **2010**, *75*, 518–521.

1.48 (s, 18H); $^{13}C\{^1H\}$ NMR (101 MHz, $CDCl_3$) δ 152.9, 82.4, 62.6, 46.1, 29.8, 28.2, 25.4 ppm. HRMS (ESI) m/z: $[M+H]^+$: calculated for $C_{14}H_{28}NO_5^+$ 290.1962, found 290.1962.

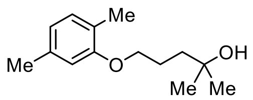

5-(2,5-dimethylphenoxy)-2-methylpentan-2-ol (**28**).[11] The title compound was prepared according to a modification of the general procedure using 1-benzylidene-3-oxoisoindolin-2-yl 5-(2,5-dimethylphenoxy)-2,2-dimethylpentanoate (188 mg, 0.40 mmol, 1.0 equiv.), methyl 4-nitrobenzenesulfonate (130 mg, 0.60 mmol, 1.5 equiv.), $Fe(acac)_3$ (14 mg, 0.04 mmol, 0.1 equiv.), $NaHCO_3$ (67 mg, 0.80 mmol, 2.0 equiv.), and $PhSiH_3$ (108 mg, 123 μL, 1.00 mmol, 2.5 equiv.) in THF (6 mL, 0.07 M) and methanol (128 mg, 162 μL, 4.00 mmol, 10.0 equiv.). Purification by chromatography (Hex → Hex/AcOEt 50%) gave **28** (80 mg, 90%) as a colorless oil. Spectral data were identical to those previously reported.[8] ^1H NMR (400 MHz, $CDCl_3$) δ 7.01 (d, J = 7.4 Hz, 1H), 6.67 (d, J = 7.1 Hz, 1H), 6.64 (s, 1H), 3.97 (t, J = 6.3 Hz, 2H), 2.31 (s, 3H), 2.18 (s, 3H), 1.95 – 1.84 (m, 2H), 1.72 – 1.62 (m, 2H), 1.27 (s, 6H); $^{13}C\{^1H\}$ NMR (101 MHz, $CDCl_3$) δ 157.1, 136.6, 130.5, 123.7, 120.9, 112.2, 70.9, 68.4, 40.5, 29.5, 24.7, 21.5, 16.0 ppm. HRMS (ESI) m/z: $[M+H]^+$: calculated for $C_{14}H_{23}O_2^+$ 223.1693, found 223.1695.

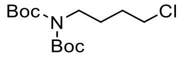

tert-butyl (tert-butoxycarbonyl)(4-chlorobutyl)carbamate (**29**).[9] The title compound was prepared according to a modification of the general procedure using 1-benzylidene-3-oxoisoindolin-2-yl 5-(bis(*tert*-butoxycarbonyl)amino)pentanoate (214 mg, 0.40 mmol, 1.0 equiv.), tosyl chloride (92 mg, 0.48 mmol, 1.2 equiv.), $Fe(acac)_3$ (14 mg, 0.04 mmol, 0.1 equiv.), and $PhSiH_3$ (108 mg, 123 μL, 1.00 mmol, 2.5 equiv.) in THF (6 mL, 0.07 M) and methanol (128 mg, 162 μL, 4.00 mmol, 10.0 equiv.). Purification by chromatography (Hex → Hex/AcOEt 25%) gave **29** (49 mg, 40%) as a colorless oil; ^1H NMR (400 MHz, $CDCl_3$) δ 3.60 (t, J = 7.0 Hz, 2H), 3.55 (t, J = 6.4 Hz, 2H), 1.85 – 1.67 (m, 4H), 1.50 (s, 18H); $^{13}C\{^1H\}$ NMR (101 MHz, $CDCl_3$) δ 152.8, 82.5, 45.7, 44.8, 30.0, 28.2, 26.5. ppm. HRMS (ESI) m/z: $[M+H]^+$: calculated for $C_{14}H_{27}ClNO_4^+$ 308.1623, found 308.1625.

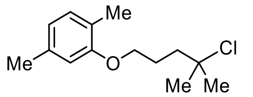

2-((4-chloro-4-methylpentyl)oxy)-1,4-dimethylbenzene (**30**).[14] The title compound was prepared according to a modification of the general procedure using 1-benzylidene-3-oxoisoindolin-2-yl 5-(2,5-dimethylphenoxy)-2,2-dimethylpentanoate (188 mg, 0.40 mmol, 1.0 equiv.), tosyl chloride (92 mg, 0.48 mmol, 1.2 equiv.), $Fe(acac)_3$ (14 mg, 0.04 mmol, 0.1 equiv.), and $PhSiH_3$ (108 mg, 123 μL, 1.00 mmol, 2.5 equiv.) in THF (6 mL, 0.07 M) and methanol (128 mg, 162 μL, 4.00 mmol, 10.0 equiv.). Purification by chromatography (Hex → Hex/AcOEt 50%) gave **30** (61 mg, 63%) as a colorless oil; ^1H NMR (400 MHz, $CDCl_3$) δ 7.02 (d, J = 7.4 Hz, 1H), 6.68 (d, J = 7.5 Hz, 1H), 6.64 (s, 1H), 3.99 (t, J = 5.9 Hz, 2H), 2.32 (s, 3H), 2.19 (s, 3H), 2.08 – 1.96 (m, 2H), 2.00 – 1.91 (m, 2H), 1.63 (s, 6H); $^{13}C\{^1H\}$ NMR (101 MHz, $CDCl_3$) δ 157.1, 136.6, 130.5, 123.7, 120.9, 112.1, 70.9, 67.8, 42.8, 32.6, 25.6, 21.6, 15.9 ppm. HRMS (ESI) m/z: $[M+H]^+$: calculated for $C_{14}H_{22}ClO^+$ 241.1354, found 241.1356.

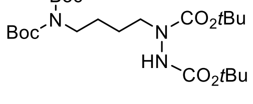

Di-tert-butyl 1-(4-(bis(tert-butoxycarbonyl)amino)butyl) hydrazine-1,2-dicarboxylate (**31**).[10] The title compound was prepared according to a modification of the general procedure using 1-benzylidene-3-oxoisoindolin-2-yl 5-(bis(*tert*-butoxycarbonyl)amino)

[8] C. Zheng, Y. Wang, Y. Xu, Z. Chen, G. Chen and S. H. Liang, *Org. Lett.*, **2018**, *20*, 4824–4827.
[9] B. Gaspar and E. M. Carreira, *Angew. Chem., Int. Ed.*, **2008**, *47*, 5758–5760.
[10] J. Waser, B. Gaspar, H. Nambu and E. M. Carreira, *J. Am. Chem. Soc.*, **2006**, *128*, 11693–11712.

pentanoate (214 mg, 0.40 mmol, 1.0 equiv.), di-*tert*-butyl diazene-1,2-dicarboxylate (138 mg, 0.60 mmol, 1.5 equiv.), $Fe(acac)_3$ (28 mg, 0.08 mmol, 0.2 equiv.), and $PhSiH_3$ (108 mg, 123 μL, 1.00 mmol, 2.5 equiv.) in THF (6 mL, 0.07 M) and methanol (128 mg, 162 μL, 4.00 mmol, 10.0 equiv.). Purification by chromatography (Hex → Hex/AcOEt 50%) gave **31** (75 mg, 37%) as a colorless oil; ^{1}H NMR (400 MHz, $CDCl_3$) δ 6.40 (s, 0.7H), 6.12 (s, 0.3H), 3.60 – 3.50 (m, 2H), 3.48 – 3.36 (m, 2H), 1.57 – 1.52 (m, 4H), 1.50 – 1.47 (m, 20H), 1.45 (s, 9H), 1.44 (s, 7H); $^{13}C\{^1H\}$ NMR (101 MHz, $CDCl_3$) δ 155.5, 155.2, 152.9, 152.8, 82.3, 81.1, 50.6, 49.2, 46.2, 46.1, 28.3, 28.2, 26.3, 25.5, 25.1, 24.8 ppm. HRMS (ESI) m/z: $[M+H]^+$: calculated for $C_{24}H_{46}N_3O_8^+$ 504.3279, found 504.3277.

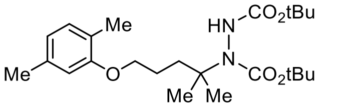

Di-tert-butyl 1-(5-(2,5-dimethylphenoxy)-2-methylpentan-2-yl)hydrazine-1,2-dicarboxylate (**32**).[15] The title compound was prepared according to a modification of the general procedure using 1-benzylidene-3-oxoisoindolin-2-yl 5-(2,5-dimethylphenoxy)-2,2-dimethylpentanoate (188 mg, 0.40 mmol, 1.0 equiv.), di-*tert*-butyl diazene-1,2-dicarboxylate (138 mg, 0.60 mmol, 1.5 equiv.), $Fe(acac)_3$ (28 mg, 0.08 mmol, 0.2 equiv.), and $PhSiH_3$ (108 mg, 123 μL, 1.00 mmol, 2.5 equiv.) in THF (6 mL, 0.07 M) and methanol (128 mg, 162 μL, 4.00 mmol, 10.0 equiv.). Purification by chromatography (Hex → Hex/AcOEt 50%) gave **32** (82 mg, 47%) as a colorless oil; ^{1}H NMR (400 MHz, $CDCl_3$) δ 7.00 (d, J = 8.2 Hz, 1H), 6.65 (d, J = 7.4 Hz, 1H), 6.62 (s, 1H), 6.18 (s, 0.6H), 5.89 (s, 0.3H), 3.93 (t, J = 6.3 Hz, 2H), 2.30 (s, 3H), 2.17 (s, 3H), 2.13 – 1.99 (m, 1H), 1.91 – 1.69 (m, 3H), 1.54 – 1.41 (m, 21H), 1.31 (s, 3H); $^{13}C\{^1H\}$ NMR (101 MHz, $CDCl_3$) δ 157.1, 156.6, 156.2, 154.9, 154.4, 136.6, 130.4, 123.7, 120.8, 112.1, 81.5, 81.0, 80.8, 68.3, 62.0, 36.9, 28.4, 28.4, 27.1, 26.5, 24.9, 21.5, 16.0 ppm. HRMS (ESI) m/z: $[M+H]^+$: calculated for $C_{24}H_{41}N_2O_5^+$ 437.3010, found 437.3012.

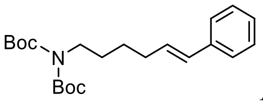

Di-tert-butyl (E)-(6-phenylhex-5-en-1-yl)carbamate (**33**).[11] The title compound was prepared according to a modification of the general procedure using 1-benzylidene-3-oxoisoindolin-2-yl 5-(bis(*tert*-butoxycarbonyl)amino)pentanoate (107 mg, 0.20 mmol, 1.0 equiv.), $Fe(acac)_3$ (7 mg, 0.02 mmol, 0.10 equiv.), (*E*)-(2-nitrovinyl)benzene (60 mg, 0.40 mmol, 2.0 equiv.), and $PhSiH_3$ (54 mg, 61 μL, 0.50 mmol, 2.5 equiv.) in THF (3 mL, 0.07 M) and methanol (64 mg, 81 μL, 2.00 mmol, 10.0 equiv.). Purification by chromatography (Hex → Hex/AcOEt 25%) gave **33** (39 mg, 52%) as a colorless oil; ^{1}H NMR (400 MHz, $CDCl_3$) δ 7.36 – 7.29 (m, 2H), 7.32 – 7.24 (m, 2H), 7.23 – 7.14 (m, 1H), 6.38 (d, J = 15.9 Hz, 1H), 6.20 (dt, J = 15.8, 6.8 Hz, 1H), 3.63 – 3.54 (m, 2H), 2.23 (qd, J = 7.1, 1.4 Hz, 2H), 1.69 – 1.57 (m, 2H), 1.50 (s, 18H), 1.50 – 1.41 (m, 2H); $^{13}C\{^1H\}$ NMR (101 MHz, $CDCl_3$) δ 152.9, 137.9, 130.7, 130.3, 128.6, 127.0, 126.1, 82.2, 46.4, 32.8, 28.8, 28.2, 26.7 ppm. HRMS (ESI) m/z: $[M+Na]^+$: calculated for $C_{22}H_{33}NNaO_4^+$ 398.2302, found 398.2310.

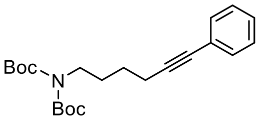

Di-tert-butyl (6-phenylhex-5-yn-1-yl)carbamate (**34**).[12] The title compound was prepared according to a modification of the general procedure using 1-benzylidene-3-oxoisoindolin-2-yl 5-(bis(*tert*-butoxycarbonyl)amino)pentanoate (107 mg, 0.20 mmol, 1.0 equiv.), $Fe(acac)_3$ (7 mg, 0.02 mmol, 0.10 equiv.), (bromoethynyl)benzene (72 mg, 0.40 mmol,

[11] J. Zheng, D. Wang and S. Cui, *Org. Lett.*, **2015**, *17*, 4572–4575.
[12] Y. Shen, B. Huang, J. Zheng, C. Lin, Y. Liu and S. Cui, *Org. Lett.*, **2017**, *19*, 1744–1747.

2.0 equiv.), $NaHCO_3$ (17 mg, 0.20 mmol, 1.0 equiv.) and $PhSiH_3$ (54 mg, 61 μL, 0.50 mmol, 2.5 equiv.) in THF (3 mL, 0.07 M) and methanol (64 mg, 81 μL, 2.00 mmol, 10.0 equiv.). Purification by chromatography (Hex → Hex/AcOEt 25%) gave **34** (43 mg, 58%) as a colorless oil; ^{1}H NMR (400 MHz, $CDCl_3$) δ 7.41 – 7.33 (m, 2H), 7.30 – 7.22 (m, 3H), 3.68 – 3.56 (m, 2H), 2.44 (t, *J* = 7.0 Hz, 2H), 1.83 – 1.69 (m, 2H), 1.68 – 1.55 (m, 2H), 1.50 (s, 18H); ^{13}C{^{1}H} NMR (101 MHz, $CDCl_3$) δ 152.8, 131.7, 128.3, 127.7, 124.1, 90.0, 82.3, 81.1, 46.1, 28.5, 28.2, 26.2, 19.4 ppm. HRMS (ESI) m/z: $[M+Na]^+$: calculated for $C_{22}H_{31}NNaO_4^+$ 396.2145, found 396.2154.

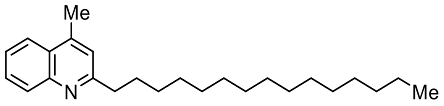

4-methyl-2-pentadecylquinoline (**35**).[13] The title compound was prepared according to a modification of the general procedure using 1-benzylidene-3-oxoisoindolin-2-yl palmitate (142 mg, 0.30 mmol, 1.0 equiv.), $Fe(acac)_3$ (106 mg, 0.30 mmol, 1.0 equiv.), lepidine (43 mg, 40 μL, 0.30 mmol, 1.0 equiv.), TFA (68 mg, 46 μL, 0.60 mmol, 2.0 equiv.), and $PhSiH_3$ (81 mg, 92 μL, 0.75 mmol, 2.5 equiv.) in THF (4.5 mL, 0.07 M) and methanol (96 mg, 122 μL, 3.00 mmol, 10.0 equiv.). Purification by chromatography (Hex → Hex/AcOEt 25%) gave **35** (72 mg, 68%) as a colorless oil; ^{1}H NMR (400 MHz, $CDCl_3$) δ 8.08 (d, *J* = 8.4 Hz, 1H), 7.95 (dd, *J* = 8.3, 1.4 Hz, 1H), 7.68 (ddd, *J* = 8.4, 6.9, 1.4 Hz, 1H), 7.51 (ddd, *J* = 8.2, 6.9, 1.3 Hz, 1H), 7.15 (d, *J* = 1.1 Hz, 1H), 3.00 – 2.84 (m, 2H), 2.69 (d, *J* = 1.0 Hz, 3H), 1.86 – 1.73 (m, 2H), 1.48 – 1.18 (m, 24H), 0.92 – 0.82 (m, 3H); ^{13}C{^{1}H} NMR (101 MHz, $CDCl_3$) δ 162.8, 147.4, 144.8, 129.37, 129.2, 126.9, 125.7, 123.7, 122.2, 39.2, 32.1, 30.3, 29.8, 29.8, 29.8, 29.8, 29.7, 29.7, 29.5, 22.8, 18.9, 14.3 ppm. HRMS (ESI) m/z: $[M+H]^+$: calculated for $C_{25}H_{40}N^+$ 354.3155, found 354.3151.

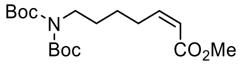

Methyl (Z)-7-(bis(tert-butoxycarbonyl)amino)hept-2-enoate (**36**).[14] The title compound was prepared according to a modification of the general procedure using 1-benzylidene-3-oxoisoindolin-2-yl 5-(bis(*tert*-butoxycarbonyl)amino)pentanoate (215 mg, 0.40 mmol, 2.0 equiv.), $Fe(acac)_3$ (72 mg, 0.20 mmol, 1.0 equiv.), methyl propiolate (17 mg, 18 μL, 0.20 mmol, 1.0 equiv.), and $PhSiH_3$ (32 mg, 37 μL, 0.30 mmol, 1.5 equiv.) in THF (3 mL, 0.07 M) and methanol (64 mg, 81 μL, 2.00 mmol, 10.0 equiv.). Purification by chromatography (Hex → Hex/AcOEt 50%) gave **36** (42 mg, 59%, 70:30 mixture of *cis* and *trans* isomers) as a colorless oil; ^{1}H NMR (400 MHz, $CDCl_3$) δ 6.20 (dt, *J* = 11.5, 7.5 Hz, 1H), 5.78 (dt, *J* = 11.5, 1.7 Hz, 1H), 3.70 (s, 3H), 3.61 – 3.53 (m, 2H), 2.67 (qd, *J* = 7.5, 1.8 Hz, 2H), 1.68 – 1.55 (m, 4H), 1.50 (s, 18H), 1.50 – 1.39 (m, 2H); ^{13}C{^{1}H} NMR (101 MHz, $CDCl_3$) δ 166.9, 152.8, 150.4, 119.7, 82.2, 51.2, 46.3, 28.8, 28.8, 28.2, 26.4 ppm. HRMS (ESI) m/z: $[M+Na]^+$: calculated for $C_{18}H_{31}NNaO_6^+$ 380.2044, found 380.2057

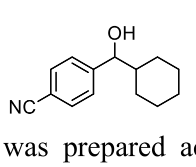

4-(cyclohexyl(hydroxy)methyl)benzonitrile (**37**).[15] The title compound was prepared according to a modification of the general procedure using 1-benzylidene-3-oxoisoindolin-2-yl cyclohexanecarboxylate (69 mg, 0.20 mmol, 1.0 equiv.), $Fe(acac)_3$ (35 mg, 0.10 mmol, 0.5 equiv.), $Fe(acac)_2$ (25 mg, 0.10 mmol, 0.5 equiv.), 4-formylbenzonitrile (26 mg, 0.20 mmol, 1.0 equiv.), and $PhSiH_3$ (54 mg, 61 μL, 0.50 mmol, 2.5 equiv.) in THF (3 mL, 0.07 M) and methanol (64 mg, 81 μL, 2.00 mmol, 10.0 equiv.) for 48 hours. Purification by chromatography (Hex → Hex/AcOEt 25%) gave **37** (15 mg, 35 %) as a colorless oil. Spectral

[13] J. Puig, J. Bonjoch and B. Bradshaw, *Org. Lett.*, **2023**, *25*, 6539–6543.
[14] L. G. Rodríguez, J. Bonjoch and B. Bradshaw, *Org. Lett.*, **2024**, *26*, 10553–10558.
[15] M. Saladrigas, J. Puig, J. Bonjoch and B. Bradshaw, *Org. Lett.*, **2020**, *22*, 8111–8115.

data were identical to those previously reported.[16] ^{1}H NMR (400 MHz, $CDCl_3$) δ 7.63 (d, *J* = 7.9 Hz, 2H), 7.41 (d, *J* = 8.1 Hz, 2H), 4.48 (d, *J* = 6.4 Hz, 1H), 1.92 – 1.51 (m, 5H), 1.47 – 1.36 (m, 1H), 1.31 – 0.86 (m, 5H); $^{13}C\{^1H\}$ NMR (101 MHz, $CDCl_3$) δ 149.0, 132.1, 127.4, 119.1, 111.2, 78.6, 45.2, 29.4, 28.2, 26.4, 26.2, 26.1 ppm. HRMS (ESI) m/z: $[M+H]^+$: calculated for $C_{14}H_{18}NO^+$ 216.1383, found 216.1377.

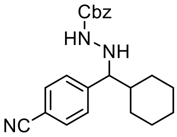

Benzyl-2-((4-cyanophenyl)(cyclohexyl)methyl)hydrazine-1-carboxylate (**38**).[17] The title compound was prepared according to a modification of the general procedure using 1-benzylidene-3-oxoisoindolin-2-yl cyclohexanecarboxylate (69 mg, 0.20 mmol, 1.0 equiv.), $Fe(acac)_3$ (18 mg, 0.05 mmol, 0.25 equiv.), benzyl-2-(4-cyanobenzylidene)hydrazine-1-carboxylate (112 mg, 0.40 mmol, 2.0 equiv.), and $PhSiH_3$ (54 mg, 61 µL, 0.50 mmol, 2.5 equiv.) in THF (3 mL, 0.07 M) and methanol (64 mg, 81 µL, 2.00 mmol, 10.0 equiv.). Purification by chromatography (Hex → Hex/AcOEt 25%) gave **38** (67 mg, 92%) as a colorless oil; ^{1}H NMR (400 MHz, $CDCl_3$) δ 7.58 (d, *J* = 7.8 Hz, 2H), 7.38 (d, *J* = 7.8 Hz, 2H), 7.36 – 7.23 (m, 5H), 6.07 (s, 1H), 5.17 – 4.96 (m, 2H), 3.96 (d, *J* = 6.7 Hz, 1H), 1.88 (d, *J* = 12.6 Hz, 1H), 1.75 (d, *J* = 13.1 Hz, 1H), 1.69 – 1.51 (m, 3H), 1.40 (d, *J* = 13.1 Hz, 1H), 1.31 – 0.95 (m, 4H), 0.90 – 0.71 (m, 1H); $^{13}C\{^1H\}$ NMR (101 MHz, $CDCl_3$) δ 157.2, 146.6, 135.9, 132.0, 129.4, 128.7, 128.5, 128.2, 119.0, 111.2, 69.8, 67.3, 42.1, 30.0, 29.2, 26.3, 26.1, 26.1 ppm. HRMS (ESI) m/z: $[M+H]^+$: calculated for $C_{22}H_{26}N_3O_2^+$ 364.2020, found 364.2023.

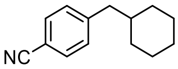

4-(cyclohexylmethyl)benzonitrile (**39**).[18] The title compound was prepared according to a modification of the general procedure using 1-benzylidene-3-oxoisoindolin-2-yl cyclohexanecarboxylate (104 mg, 0.30 mmol, 1.0 equiv.), $Fe(acac)_3$ (42 mg, 0.12 mmol, 0.4 equiv.), *N'*-(4-cyanobenzylidene)-4-methylbenzenesulfonohydrazide (90 mg, 0.30 mmol, 1.0 equiv.), and $PhSiH_3$ (81 mg, 92 µL, 0.75 mmol, 2.5 equiv.) in THF (4.5 mL, 0.07 M) and methanol (96 mg, 122 µL, 3.00 mmol, 10.0 equiv.). After the MHAT coupling, triethylamine (121 mg, 166 µL, 1.20 mmol, 4.0 equiv.) was added and the mixture was heated at reflux for 1 hour. Purification by chromatography (Hex → Hex/AcOEt 25%) gave **39** (48 mg, 81%) as a colorless oil. Spectral data were identical to those previously reported.[23] ^{1}H NMR (400 MHz, $CDCl_3$) δ 7.55 (d, *J* = 8.1 Hz, 2H), 7.23 (d, *J* = 8.1 Hz, 2H), 2.53 (d, *J* = 7.1 Hz, 2H), 1.76 – 1.59 (m, 5H), 1.59 – 1.45 (m, 1H), 1.30 – 1.06 (m, 3H), 1.03 – 0.85 (m, 2H); $^{13}C\{^1H\}$ NMR (101 MHz, $CDCl_3$) δ 147.3, 132.0, 130.0, 119.4, 109.6, 44.3, 39.7, 33.1, 26.5, 26.3 ppm. HRMS (ESI) m/z: $[M+H]^+$: calculated for $C_{14}H_{18}N^+$ 200.1434, found 200.1432.

Synthesis of methyl 2-(phenylethynyl)benzoate (SI-I)[19]

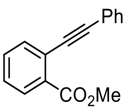

A mixture of methyl 2-bromobenzoate (5.16 g, 3.4 mL, 24.0 mmol, 1.0 equiv.), CuI (274 mg, 1.44 mmol, 0.06 equiv.), PPh_3 (567 mg, 2.16 mmol, 0.09 equiv.) and $[Pd(OAc)_2]_3$ (162 mg, 0.72 mmol, 0.03 equiv.) was dissolved in degassed diethylamine (80 mL, 0.30 M).

[16] C. C. K. Keh, C. Wei and C. J. Li, *J. Am. Chem. Soc.*, **2003**, *125*, 4062–4063.
[17] M. Saladrigas, G. Loren, J. Bonjoch and B. Bradshaw, *ACS Catal.*, **2018**, *8*, 11699–11703.
[18] B. Bradshaw, J. Bonjoch and M. Saladrigas, *Org. Lett.*, **2020**, *22*, 684–688.
[19] K. Y. Yoon and G. Dong, *Angew. Chem., Int. Ed.*, **2018**, *57*, 8592–8596.

Afterward, phenylacetylene (3.68 g, 4.0 mL, 36.0 mmol, 1.5 equiv.) was added dropwise and the resulting mixture was heated at 60 °C for 16 hours. Then, the crude mixture was concentrated under vacuum. Purification by chromatography (Hex → Hex/AcOEt 25%) gave **SI-I** (5.38 g, 95%) as a yellow oil. Spectral data were identical to those previously reported.[20] ^{1}H NMR (400 MHz, $CDCl_3$) δ 7.98 (dd, *J* = 7.8, 1.4 Hz, 1H), 7.68 – 7.62 (m, 1H), 7.61 – 7.55 (m, 2H), 7.50 (td, *J* = 7.6, 1.4 Hz, 1H), 7.43 – 7.32 (m, 4H), 3.97 (s, 3H); ^{13}C{^{1}H} NMR (101 MHz, $CDCl_3$) δ 166.9, 134.1, 132.0, 131.9, 131.8, 130.6, 128.7, 128.5, 128.0, 123.8, 123.5, 94.5, 88.4, 52.3 ppm.

Synthesis of methyl 5-((*tert*-butoxycarbonyl)amino)pentanoate (SI-VII)[21,22]

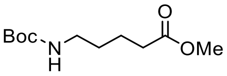

To a solution of 5-aminovaleric acid (2.93 g, 25.0 mmol, 1.0 equiv.) in methanol (38 mL, 0.66 M) at 0 °C was added thionyl chloride (4.46 g, 2.7 mL, 37.5 mmol, 1.5 equiv.) dropwise and the resulting mixture was left to stir at room temperature for 2 hours. Afterward, the solvent and the excess of thionyl chloride were evaporated and the residue was dissolved in acetonitrile (63 mL, 0.40 M). Triethylamine (3.04 g, 4.2 mL, 30.0 mmol, 1.2 equiv.) and Boc_2O (5.46 g, 25.0 mmol, 1.0 equiv.) were slowly added and the solution was left to stir at room temperature for 16 hours. Then, the solvent was evaporated, and ethyl acetate (40 mL) and 1 M hydrochloric acid (40 mL) were added. The two phases were separated, and the aqueous layer was extracted with ethyl acetate (2 × 40 mL). The combined organic layers were dried with Na_2SO_4 and concentrated. Purification by chromatography (Hex → Hex/AcOEt 25%) gave **SI-VII** (5.20 g, 90%) as a yellow oil. ^{1}H NMR (400 MHz, $CDCl_3$) δ 3.67 (s, 3H), 3.12 (t, *J* = 6.9 Hz, 2H), 2.34 (t, *J* = 7.4 Hz, 2H), 1.72 – 1.60 (m, 2H), 1.57 – 1.48 (m, 2H), 1.44 (s, 9H); ^{13}C{^{1}H} NMR (101 MHz, $CDCl_3$) δ 174.0, 156.1 79.3, 51.7, 40.3, 33.7, 29.6, 28.5, 22.2 ppm. HRMS (ESI) m/z: $[M+H]^+$: calculated for $C_{11}H_{22}NO_4^+$ 232.1543, found 232.1547.

Synthesis of methyl 5-((di-*tert*-butoxycarbonyl)amino)pentanoate (SI-VIII)[23]

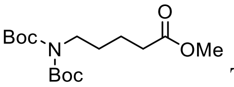

To a solution of methyl 5-((*tert*-butoxycarbonyl)amino)pentanoate (3.47 g, 15.0 mmol, 1.0 equiv.), DMAP (183 mg, 1.50 mmol, 0.10 equiv.) and triethylamine (1.82 g, 2.5 mL, 18.0 mmol, 1.2 equiv.) in acetonitrile (38 mL, 0.40 M) was added Boc_2O (3.27 g, 15.0 mmol, 1.0 equiv.) and the resulting mixture was left to stir at room temperature for 16 hours. Then, the solvent was evaporated, and ethyl acetate (30 mL) and 1 M hydrochloric acid (30 mL) were added. The two phases were separated, and the aqueous layer was extracted with ethyl acetate (2 × 30 mL). The combined organic layers were dried with Na_2SO_4 and concentrated. Purification by chromatography (Hex → Hex/AcOEt 25%) gave **SI-VIII** (3.88 g, 78%) as a yellow oil. ^{1}H NMR (400 MHz, $CDCl_3$) δ 3.66 (s, 3H), 3.57 (t, *J* = 6.9 Hz, 2H), 2.34 (t, *J* = 7.2 Hz, 2H), 1.67 – 1.57 (m, 4H), 1.50 (s, 18H); ^{13}C{^{1}H} NMR (101 MHz, $CDCl_3$) δ 173.9, 152.7, 82.3, 51.6, 46.0, 33.8, 28.6, 28.2, 22.3 ppm. HRMS (ESI) m/z: $[M+H]^+$: calculated for $C_{16}H_{30}NO_6^+$ 332.2068, found 332.2070.

[20] J. H. Park, S. V. Bhilare and S. W. Youn, *Org. Lett.*, **2011**, *13*, 2228–2231.

[21] F. F. Diaz-Rullo, F. Zamberlan, R. E. Mewis, M. Fekete, L. Broche, L. A. Cheyne, S. Dall'Angelo, S. B. Duckett, D. Dawson and M. Zanda, *Bioorg. Med. Chem.*, **2017**, *25*, 2730–2742.

[22] G. Bort, S. Catoen, H. Borderies, A. Kebsi, S. Ballet, G. Louin, M. Port and C. Ferroud, *Eur. J. Med. Chem.*, **2014**, *87*, 843–861.

[23] E. A. Englund, H. N. Gopi and D. M. Appella, *Org. Lett.*, **2004**, *6*, 213–215.

Synthesis of 5-((di-*tert*-butoxycarbonyl)amino)pentanoic acid (SI-IX)

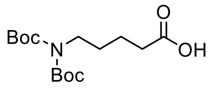

To a solution of methyl 5-((di-*tert*-butoxycarbonyl)amino)pentanoate (4.97 g, 15.0 mmol, 1.0 equiv.) in a 1:1 THF/H_2O mixture (40 mL, 0.38 M) was added sodium hydroxide (720 mg, 18.0 mmol, 1.2 equiv.) and the resulting mixture was stirred at room temperature for 4 hours. Then, the mixture was neutralized with a 10% solution of citric acid (50 mL) and subsequently extracted with ethyl acetate (3 × 40 mL). The combined organic layers were dried with Na_2SO_4 and concentrated. Purification by chromatography (Hex → Hex/AcOEt 25%) gave **SI-IX** (4.48 g, 94%) as a yellow oil. ^{1}H NMR (400 MHz, $CDCl_3$) δ 3.62 – 3.51 (m, 2H), 2.42 – 2.32 (m, 2H), 1.68 – 1.56 (m, 4H), 1.49 (s, 18H); $^{13}C\{^1H\}$ NMR (101 MHz, $CDCl_3$) δ 179.4, 152.8, 82.4, 46.0, 33.7, 28.5, 28.2, 22.0 ppm. HRMS (ESI) m/z: $[M+H]^+$: calculated for $C_{15}H_{28}NO_6^+$ 318.1911, found 318.1911.

COPIES OF NMR SPECTRA

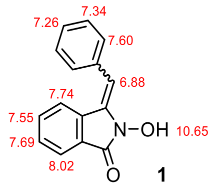

1

auto-16072024-112402.1.fid 1H 400 MHz
Equip: B400Q / N.Inv: 1035091
N.Reg: 24070675
Usuari: san / Mostra: LRG613CH
Nom: LAURA RODRIGUEZ GONZALEZ
Data: 16/07/2024 12:44:09 h./ Ope.: AUTOSERVEI
Experiment: A_1H-zg30 Solvent: DMSO Operator:

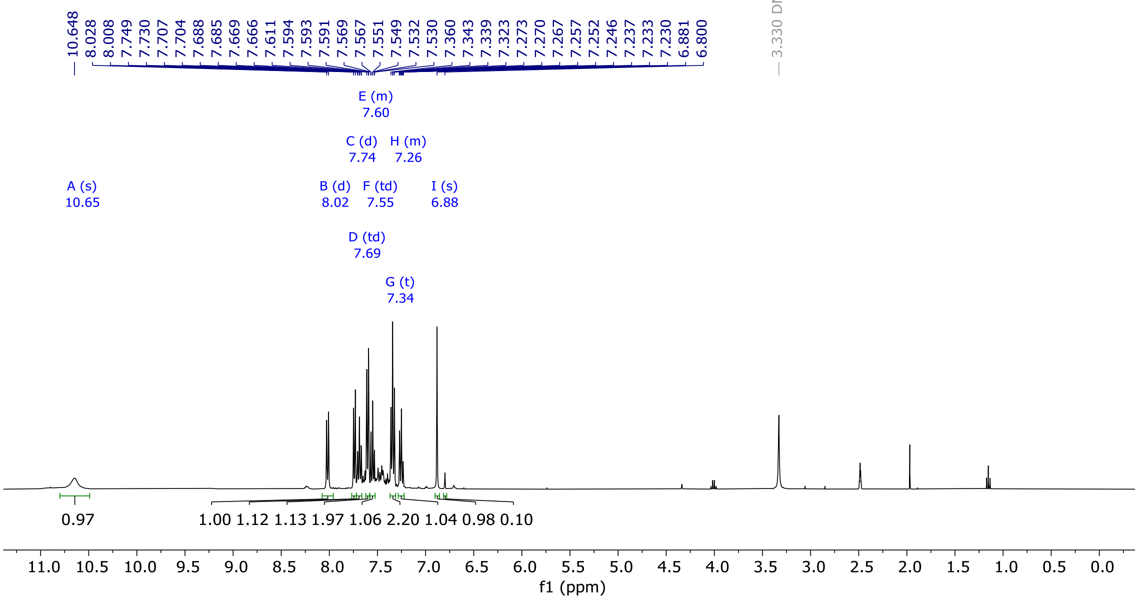

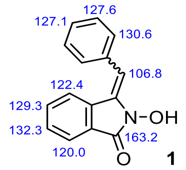

1

auto-16072024-112402.2.fid 13C{1H} 101 MHz
Equip: B400Q / N.Inv: 1035091
N.Reg: 24070675
Usuari: san / Mostra: LRG613CH
Nom: LAURA RODRIGUEZ GONZALEZ
Data: 16/07/2024 22:48:13 h./ Ope.: AUTOSERVEI
Experiment: A_13C-zgpg30 Solvent: DMSO Operator:

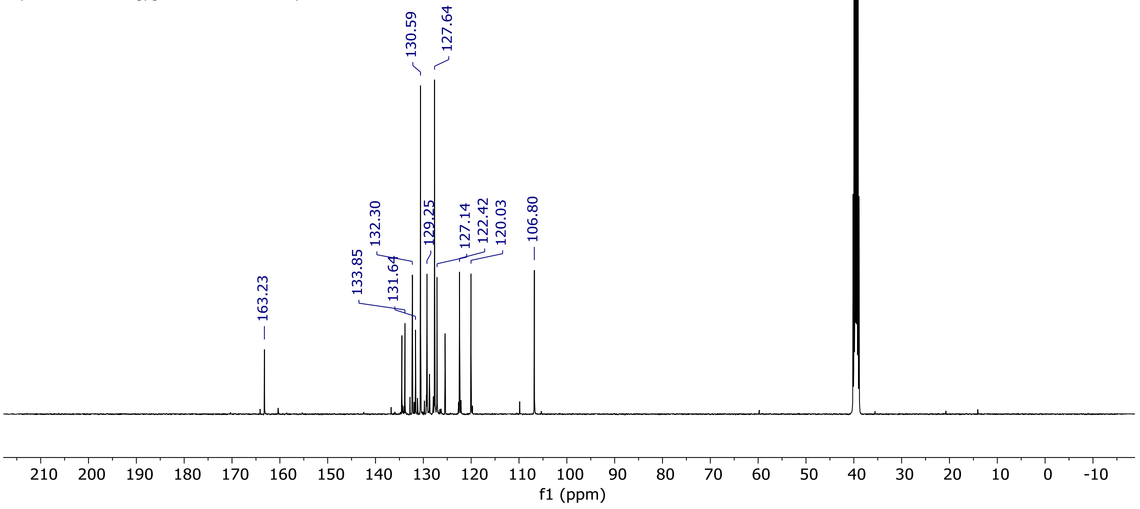

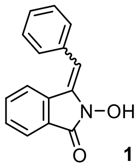

1

2D-COSY

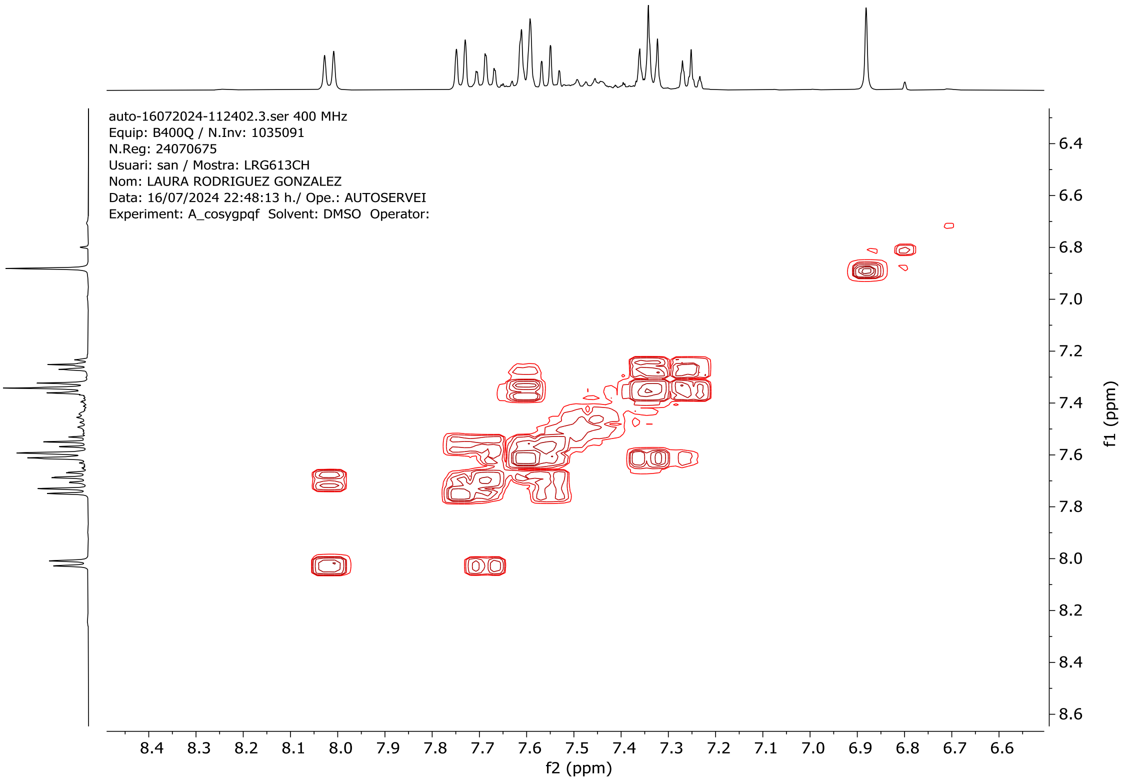

2D-HSQC

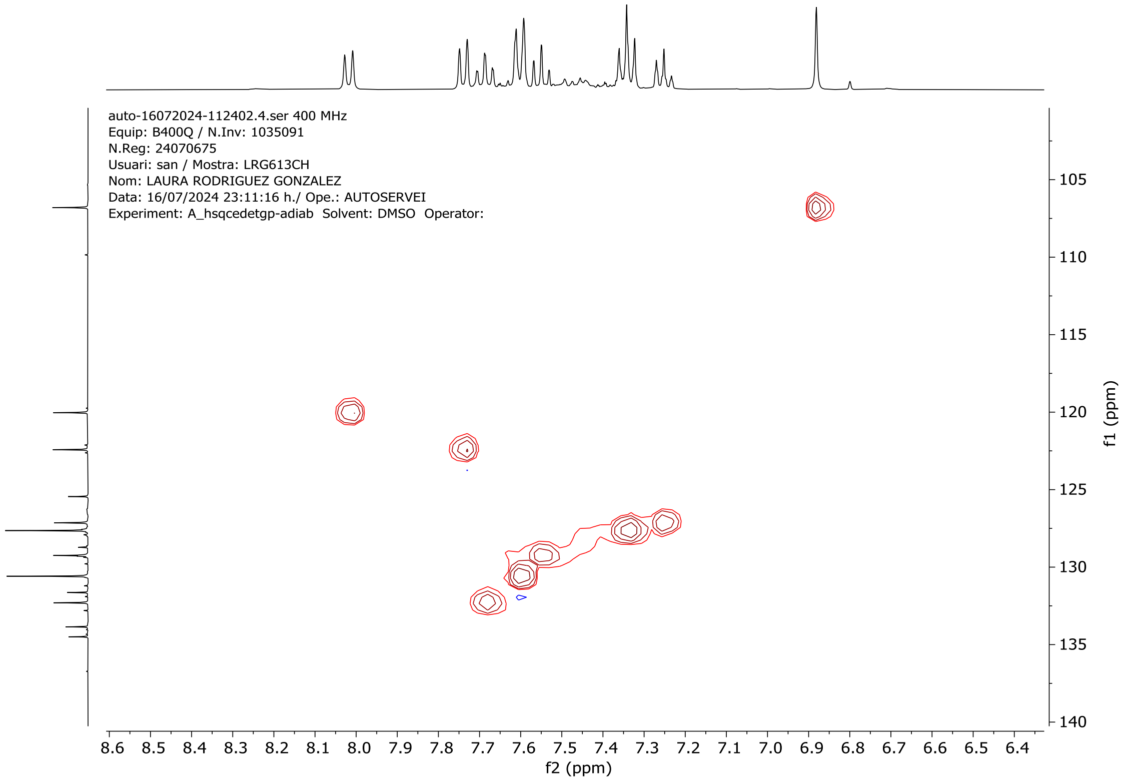

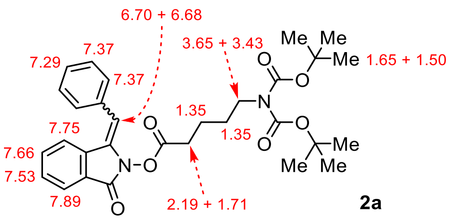

2a

24020357_B400FA_13022024_ASV005COLT25.1.fid 1H 400 MHz
Equip: B400F / N.Inv: 1037597
N.Reg: 24020357
Usuari: san / Mostra: ASV005COLT25
Nom: LAURA RODRIGUEZ GONZALEZ
Data: 13/02/2024 13:40:45 h./ Ope.: AUTOSERVEI
Experiment: A-H1-zg30 Solvent: CDCl3

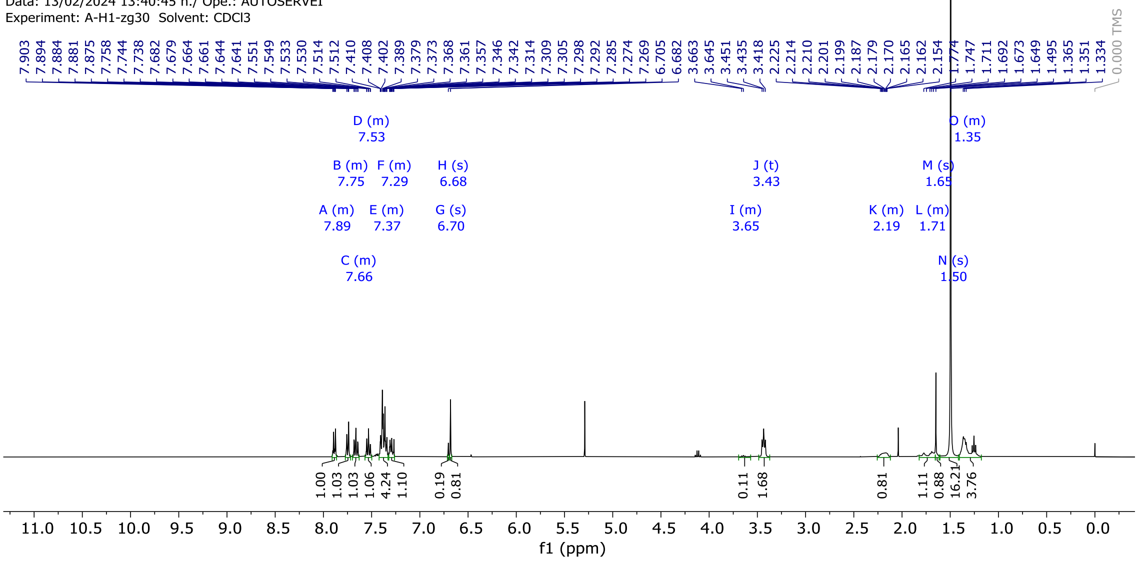

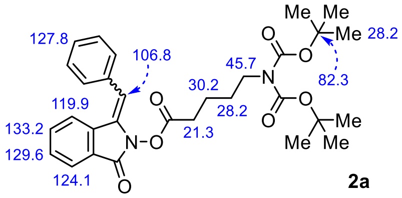

2a

24020357_B400FA_13022024_ASV005COLT25.2.fid 13C{1H} 101 MHz
Equip: B400F / N.Inv: 1037597
N.Reg: 24020357
Usuari: san / Mostra: ASV005COLT25
Nom: LAURA RODRIGUEZ GONZALEZ
Data: 13/02/2024 22:11:26 h./ Ope.: AUTOSERVEI
Experiment: A-C13-zgpg30 Solvent: CDCl3

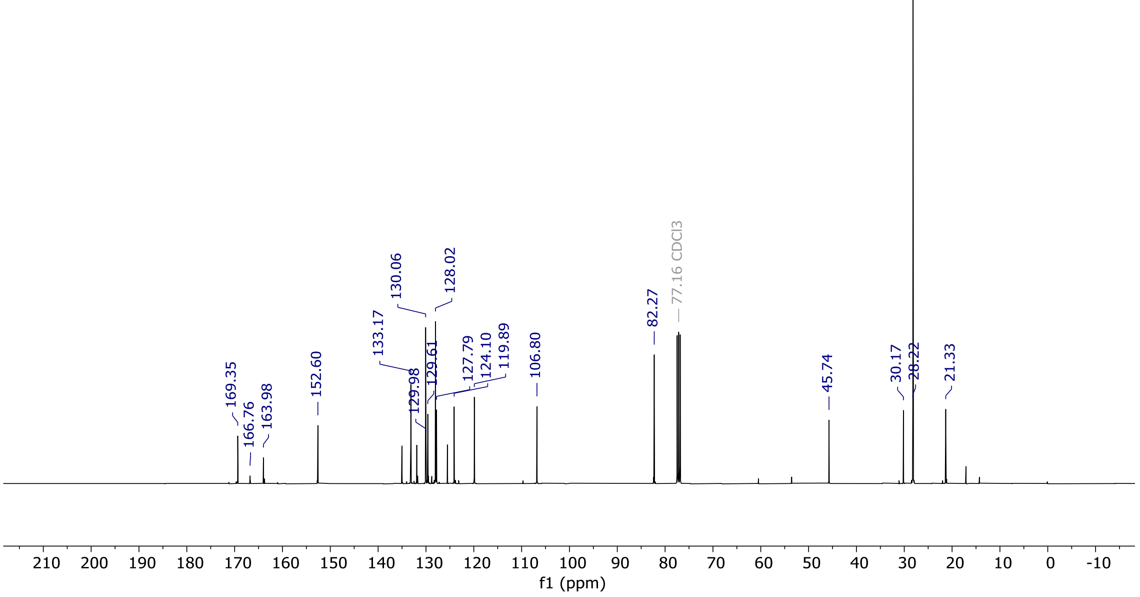

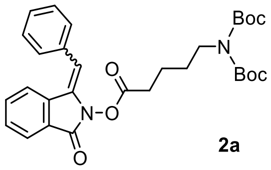

2a

2D-COSY

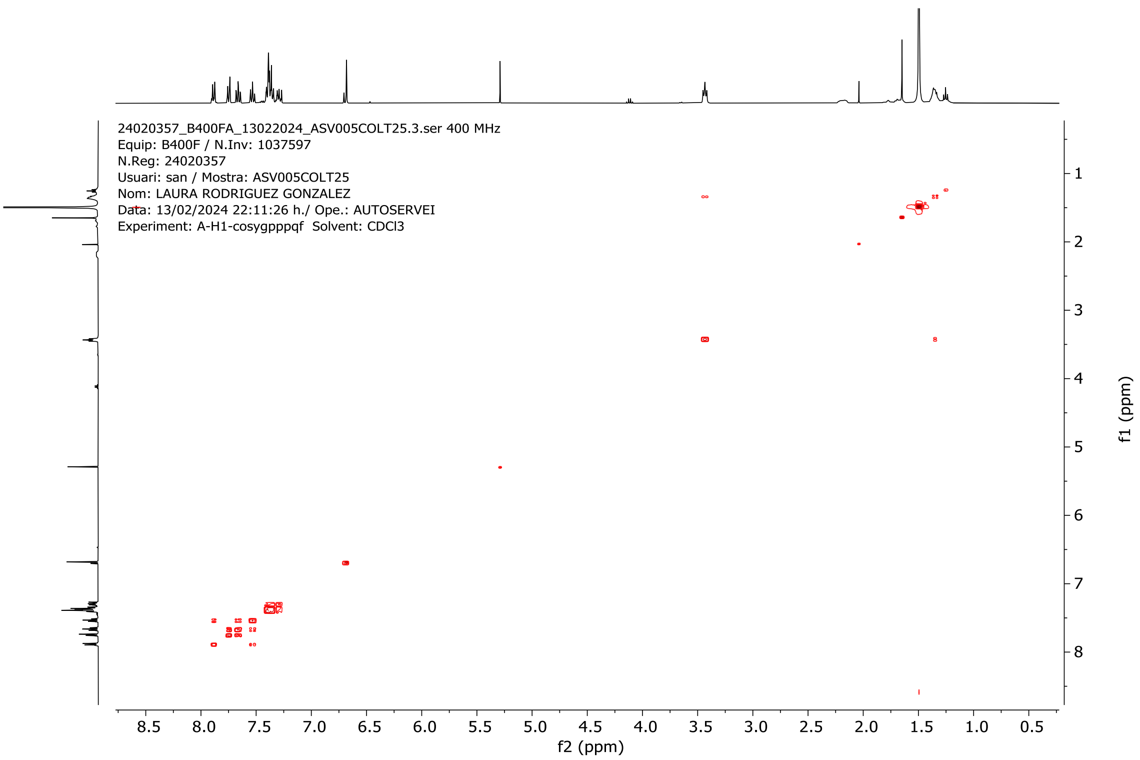

2D-HSQC

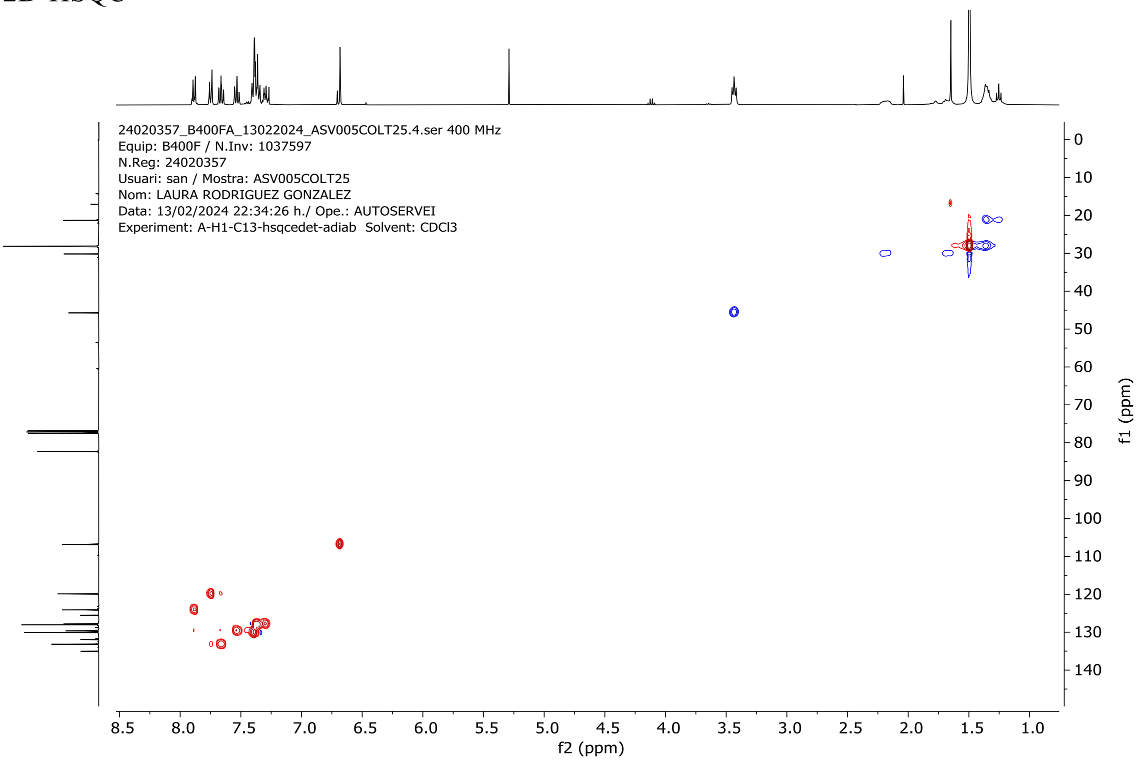

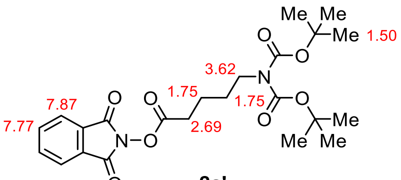
Me Me
Me 1.50
3.62
1.75
1.75
7.87
7.77
2.69
Me
Me Me

2a'

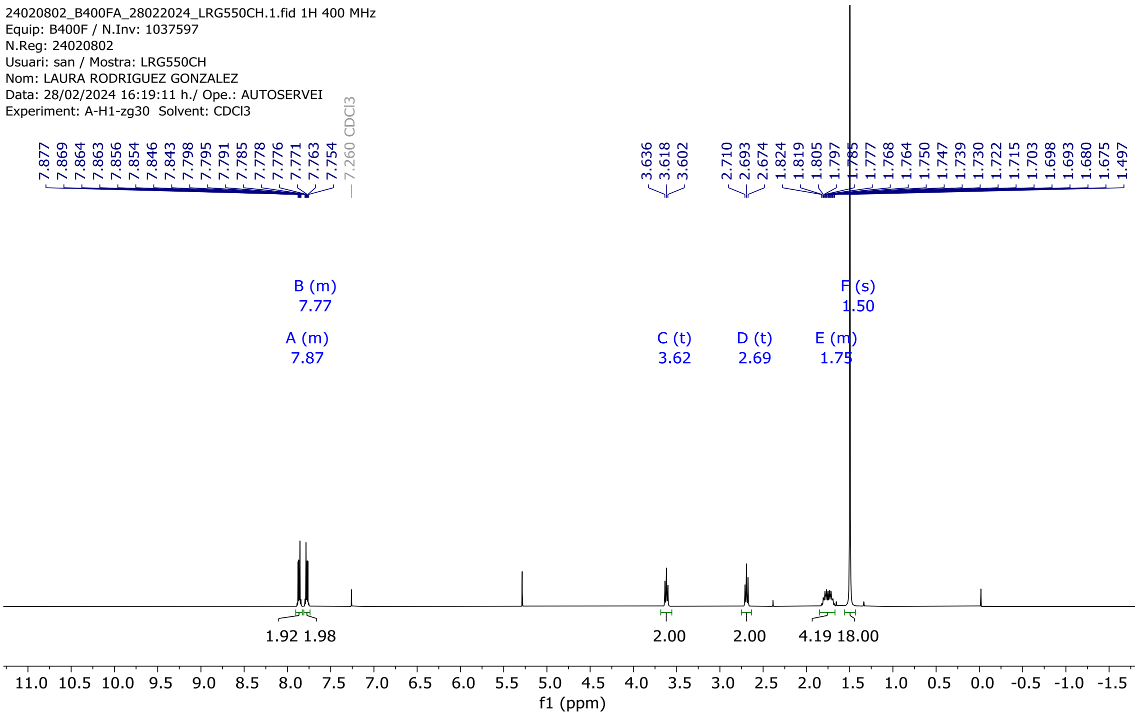
24020802_B400FA_28022024_LRG550CH.1.fid 1H 400 MHz
Equip: B400F / N.Inv: 1037597
N.Reg: 24020802
Usuari: san / Mostra: LRG550CH
Nom: LAURA RODRIGUEZ GONZALEZ
Data: 28/02/2024 16:19:11 h./ Ope.: AUTOSERVEI
Experiment: A-H1-zg30 Solvent: CDCl3
7.260 CDCl3
B (m) 7.77
A (m) 7.87
C (t) 3.62
D (t) 2.69
E (m) 1.75
F (s) 1.50
1.92 1.98
2.00
2.00
4.19 18.00
f1 (ppm)

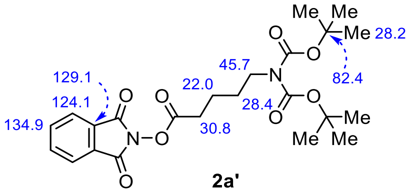
Me Me
Me 28.2
82.4
45.7
22.0
28.4
129.1
124.1
134.9
30.8
Me
Me Me

2a'

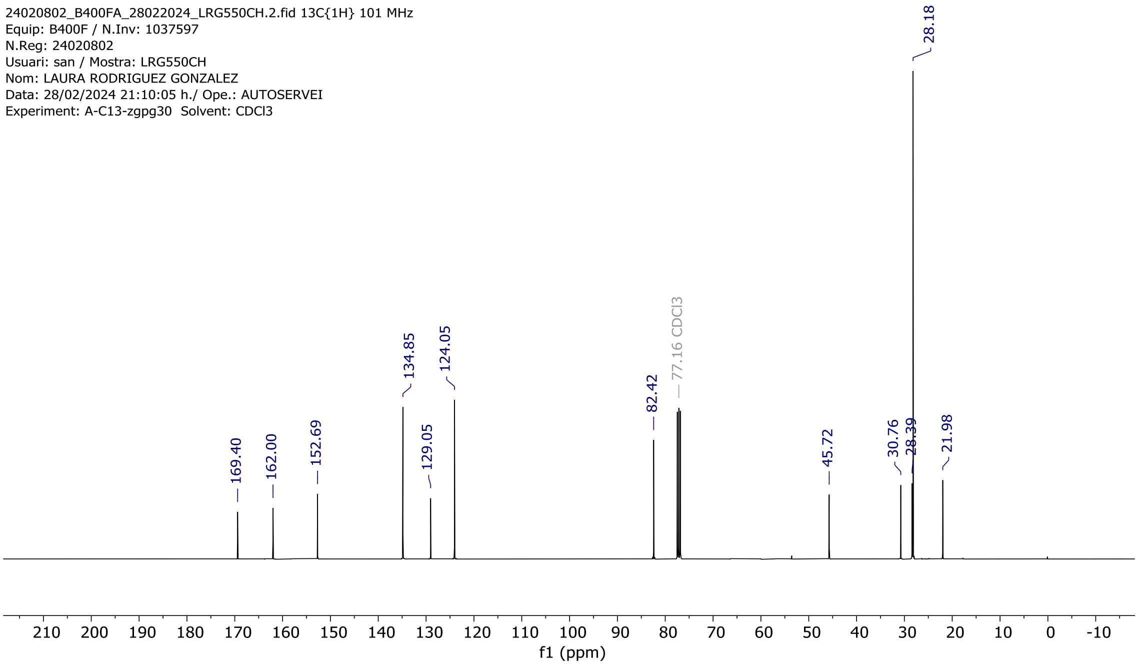
24020802_B400FA_28022024_LRG550CH.2.fid 13C{1H} 101 MHz
Equip: B400F / N.Inv: 1037597
N.Reg: 24020802
Usuari: san / Mostra: LRG550CH
Nom: LAURA RODRIGUEZ GONZALEZ
Data: 28/02/2024 21:10:05 h./ Ope.: AUTOSERVEI
Experiment: A-C13-zgpg30 Solvent: CDCl3
169.40
162.00
152.69
134.85
129.05
124.05
82.42
77.16 CDCl3
45.72
30.76
28.39
28.18
21.98
f1 (ppm)

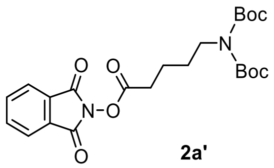

2a'

2D-COSY

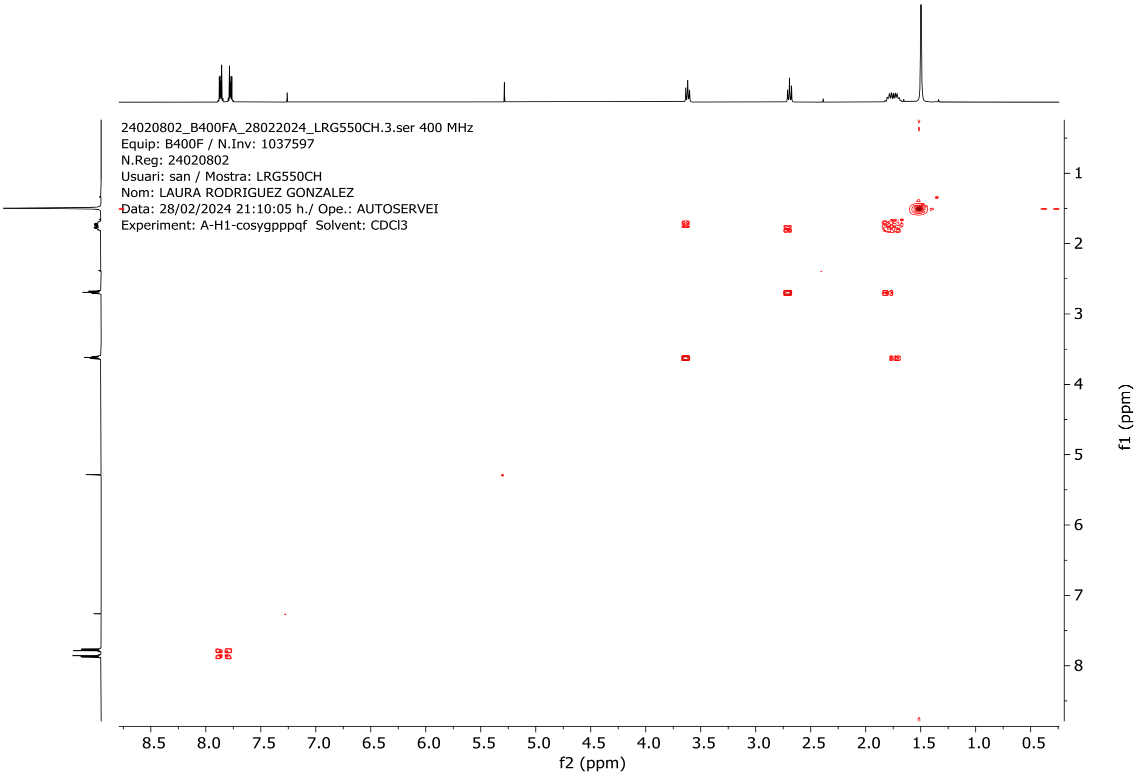

2D-HSQC

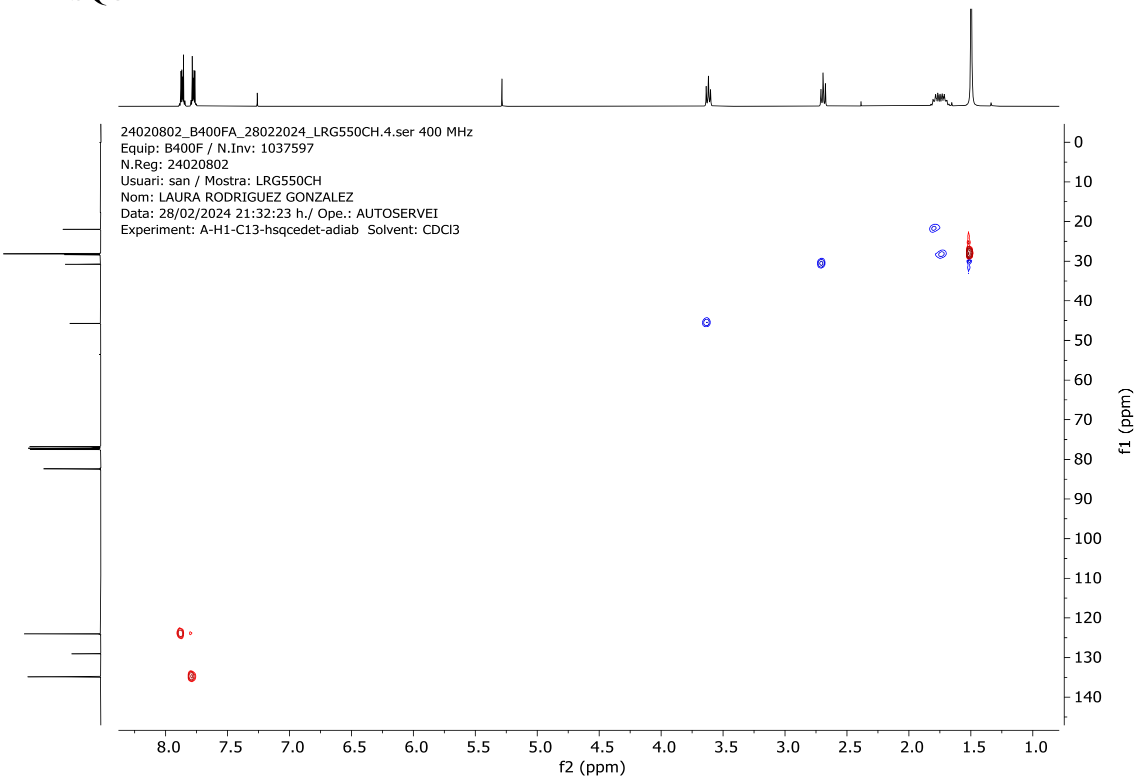

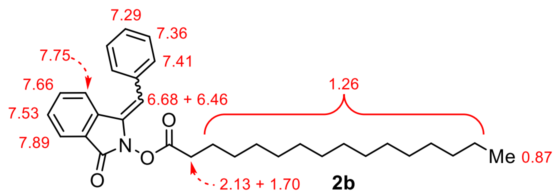

ASV029-20-25.1.fid 1H 400 MHz
Equip: B400F / N.Inv: 1037597
N.Reg: 24030748
Usuari: san / Mostra: ASV029-20-25
Nom: AINA SERRA VERT
Data: 19/03/2024 16:25:04 h./ Ope.: AUTOSERVEI
Experiment: A-H1-zg30 Solvent: CDCl3

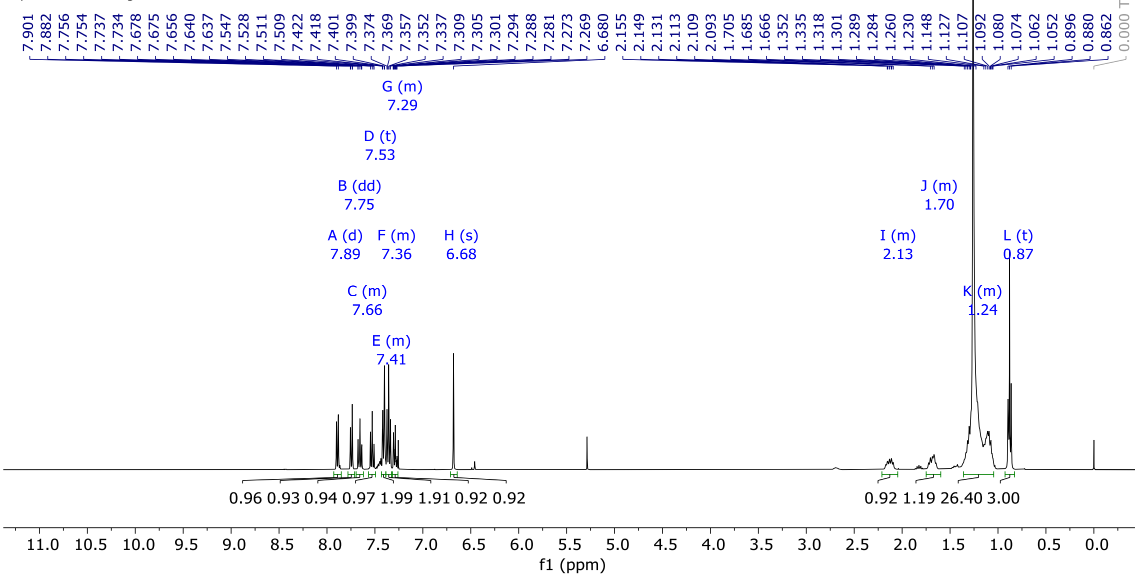

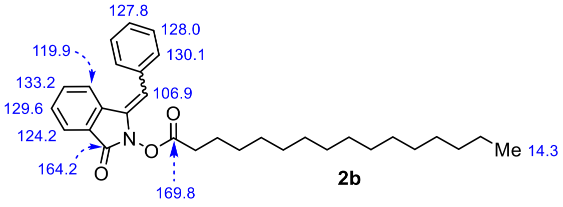

ASV029-20-25.2.fid 13C{1H} 101 MHz
Equip: B400F / N.Inv: 1037597
N.Reg: 24030799
Usuari: san / Mostra: ASV029-20-25
Nom: AINA SERRA VERT
Data: 20/03/2024 20:32:35 h./ Ope.: AUTOSERVEI
Experiment: A-C13-zgpg30 Solvent: CDCl3

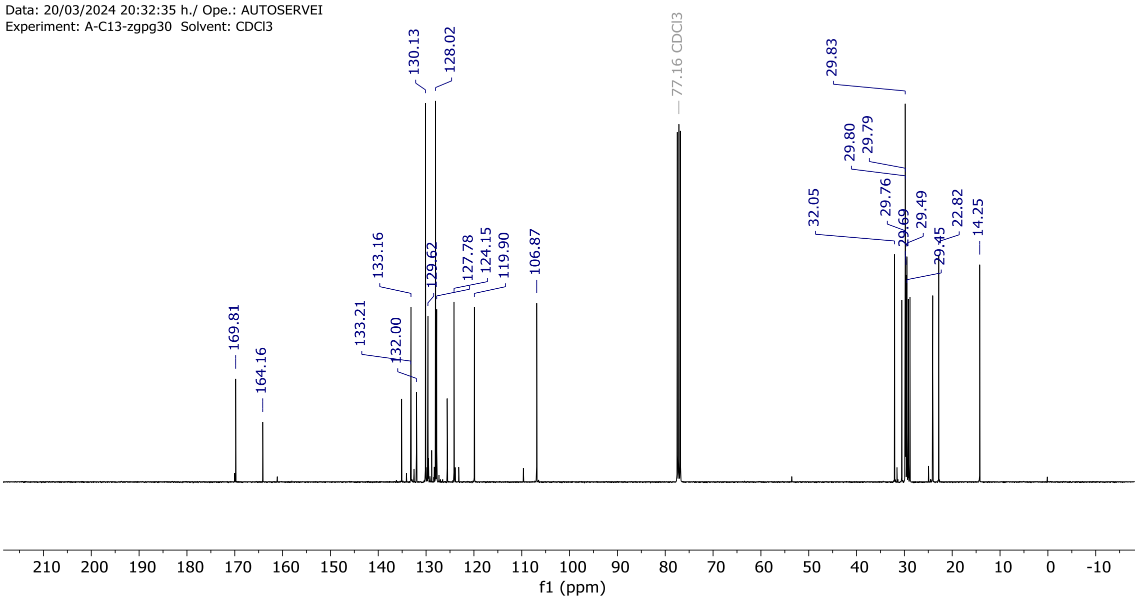

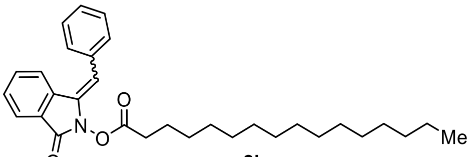

2b

2D-COSY

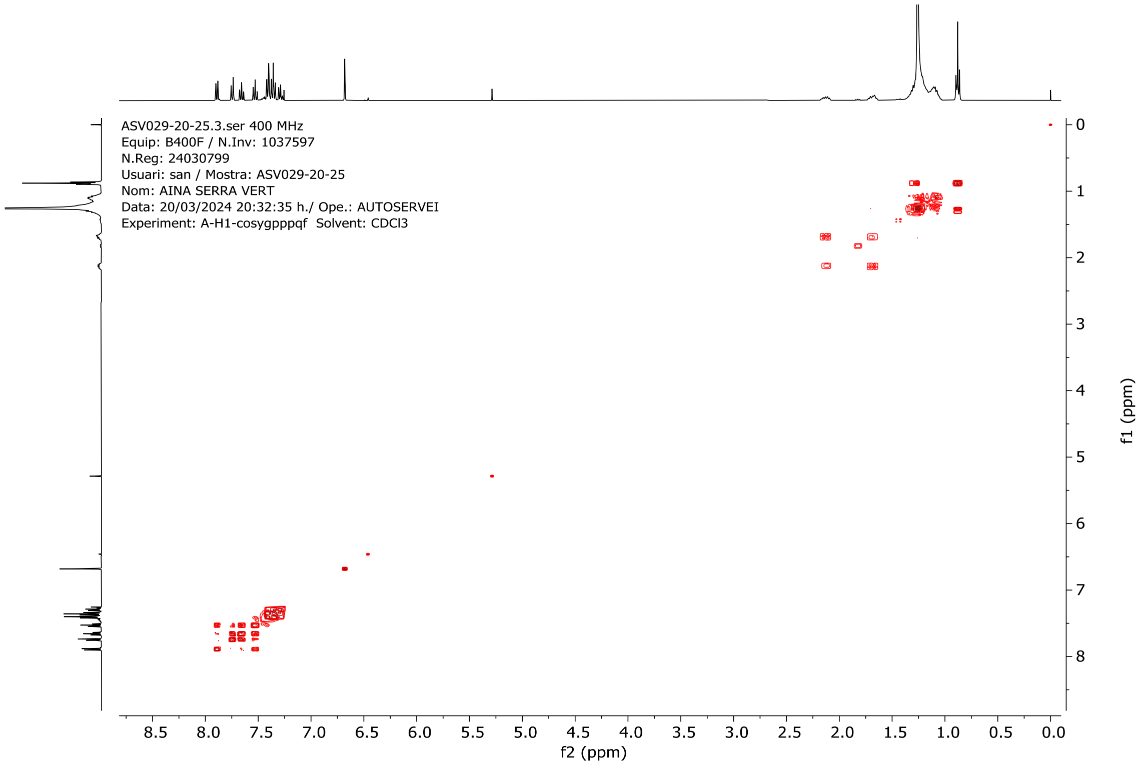

2D-HSQC

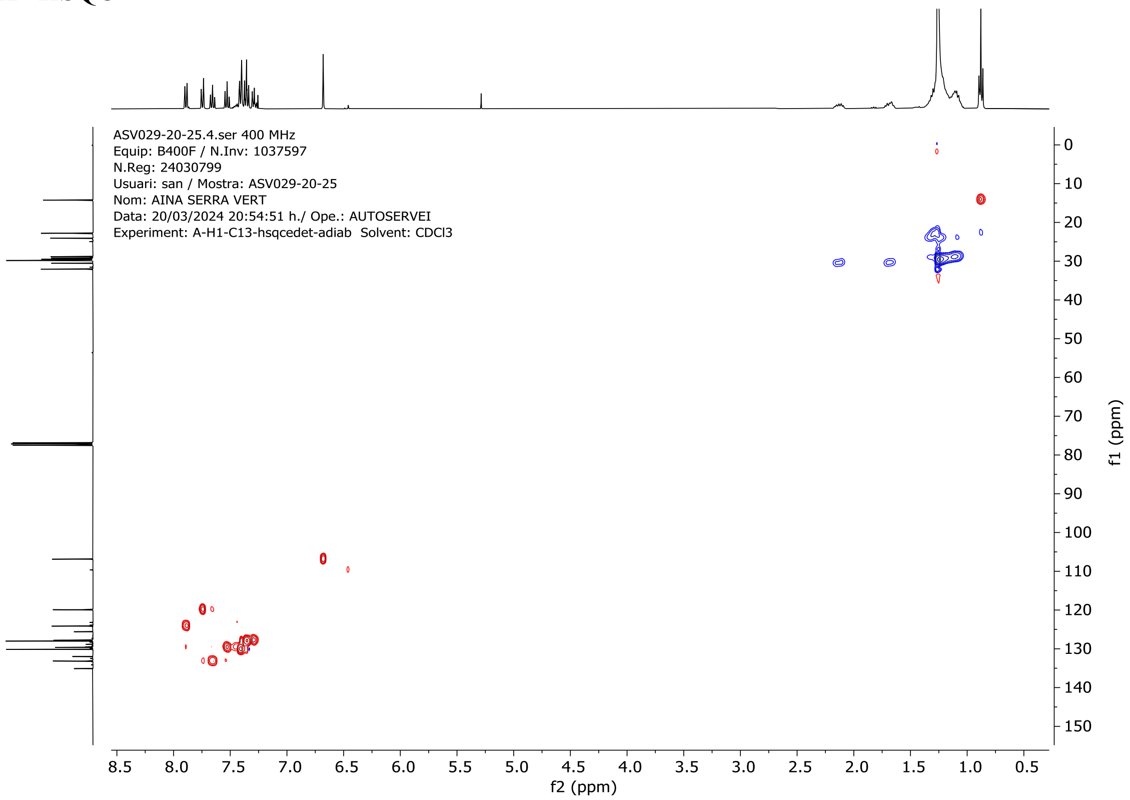

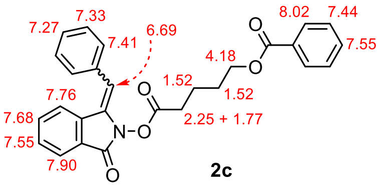

2c

5923-2024_B500QA_14062024_ASV061CH.10.fid 1H 500 MHz
Equip: B500Q / N.Inv: 1028917
N.Reg: 5923/2024
Usuari: san / Mostra: ASV061CH
Nom: LAURA RODRIGUEZ GONZALEZ
Data: 14/06/2024 14:13:22 h./ Ope.: servei Unitat RMN
Experiment: A_1H-zg30 Solvent: CDCl3 Operator: VICTOR MERIEL

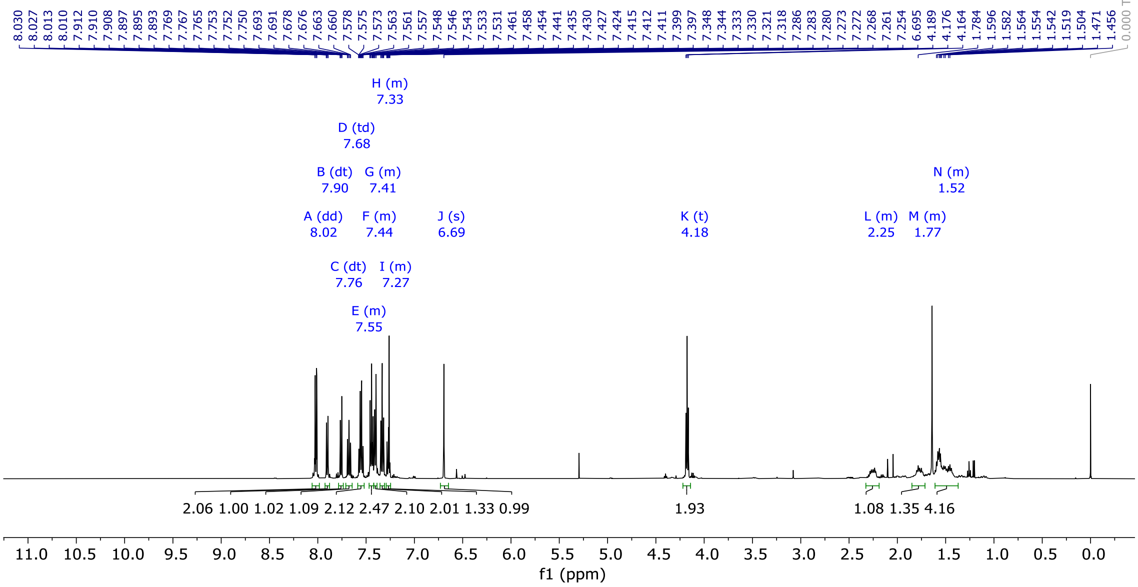

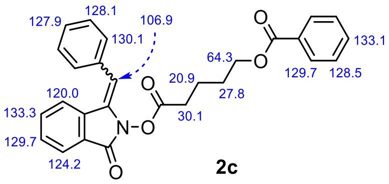

2c

5923-2024_B500QA_14062024_ASV061CH.11.fid 13C{1H} 126 MHz
Equip: B500Q / N.Inv: 1028917
N.Reg: 5923/2024
Usuari: san / Mostra: ASV061CH
Nom: LAURA RODRIGUEZ GONZALEZ
Data: 14/06/2024 14:13:22 h./ Ope.: servei Unitat RMN
Experiment: A_13C-zgpg30 Solvent: CDCl3 Operator: VICTOR MERIEL

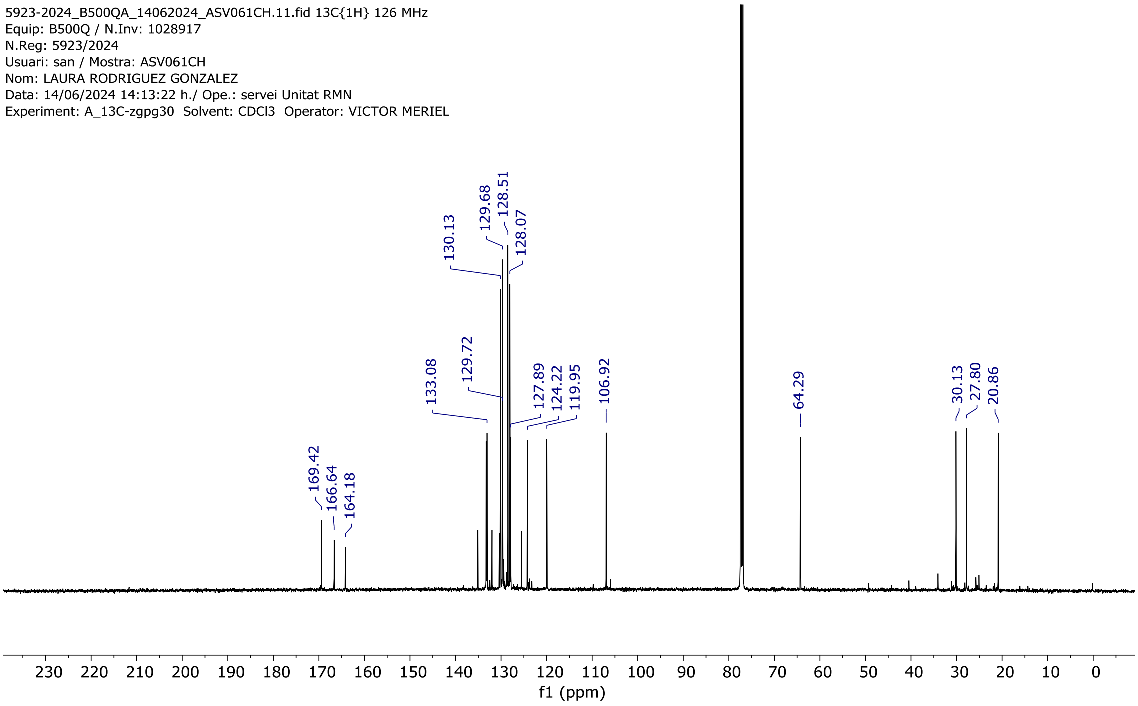

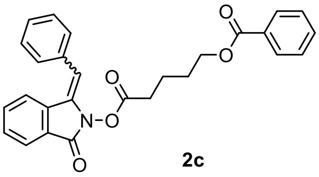

2c

2D-COSY

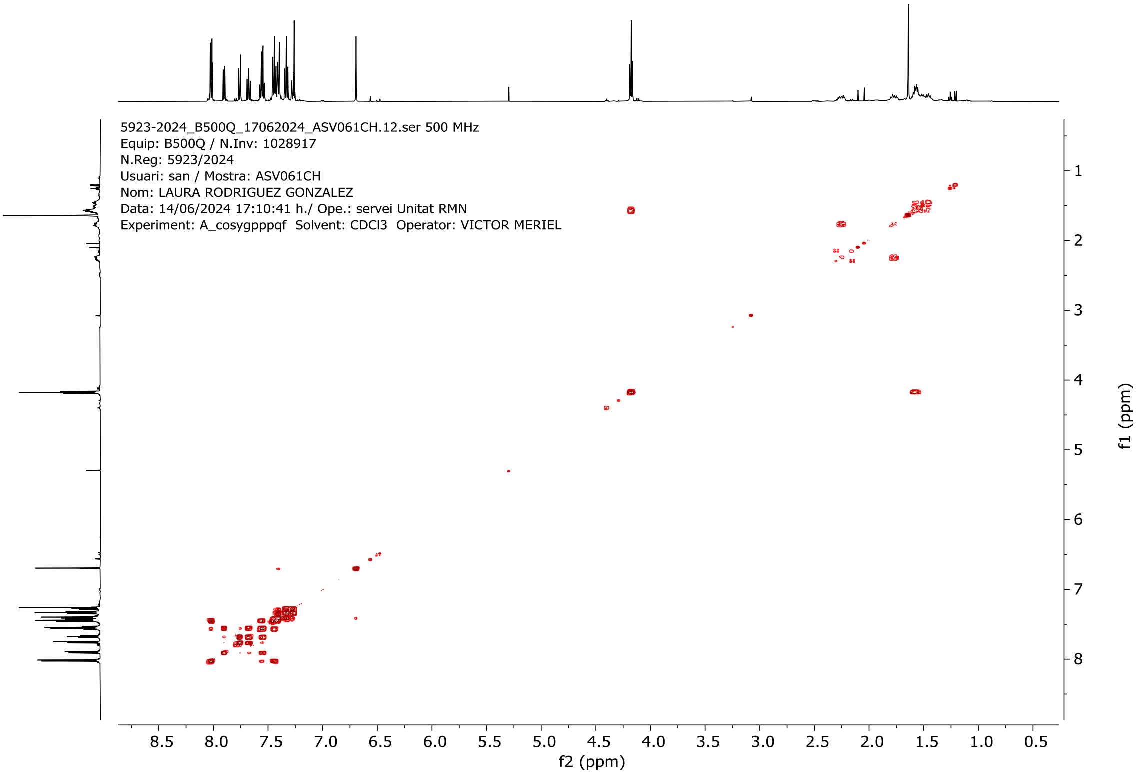

2D-HSQC

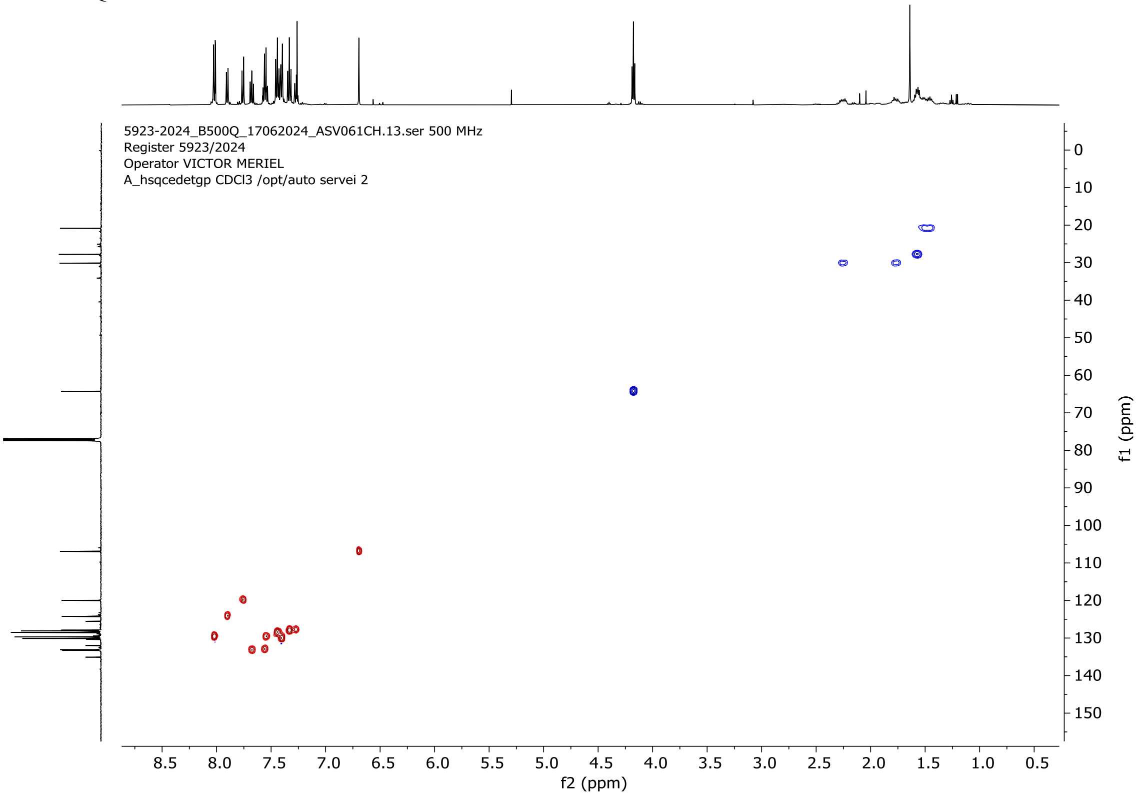

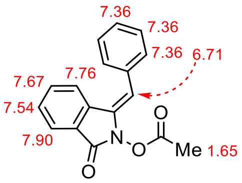

2d

auto-21062024-114127.1.fid 1H 400 MHz
Equip: B400Q / N.Inv: 1035091
N.Reg: 24061010
Usuari: san / Mostra: LRG614CH
Nom: LAURA RODRIGUEZ GONZALEZ
Data: 21/06/2024 11:58:22 h./ Ope.: AUTOSERVEI
Experiment: A_1H-zg30 Solvent: CDCl3 Operator:

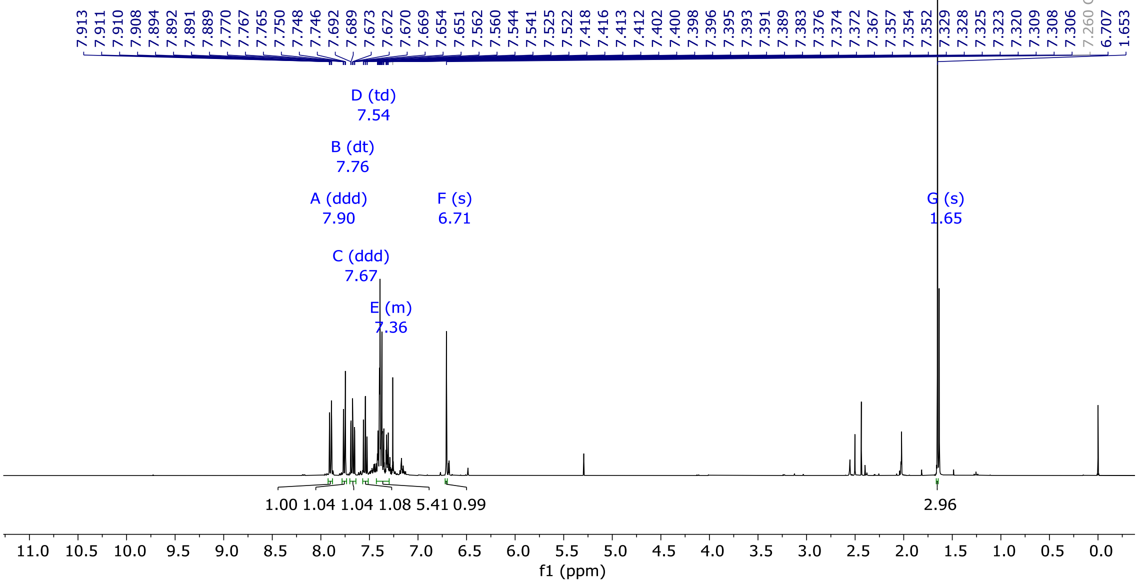

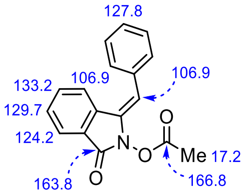

2d

auto-21062024-114127.2.fid 13C{1H} 101 MHz
Equip: B400Q / N.Inv: 1035091
N.Reg: 24061010
Usuari: san / Mostra: LRG614CH
Nom: LAURA RODRIGUEZ GONZALEZ
Data: 22/06/2024 19:43:22 h./ Ope.: AUTOSERVEI
Experiment: A_13C-zgpg30 Solvent: CDCl3 Operator:

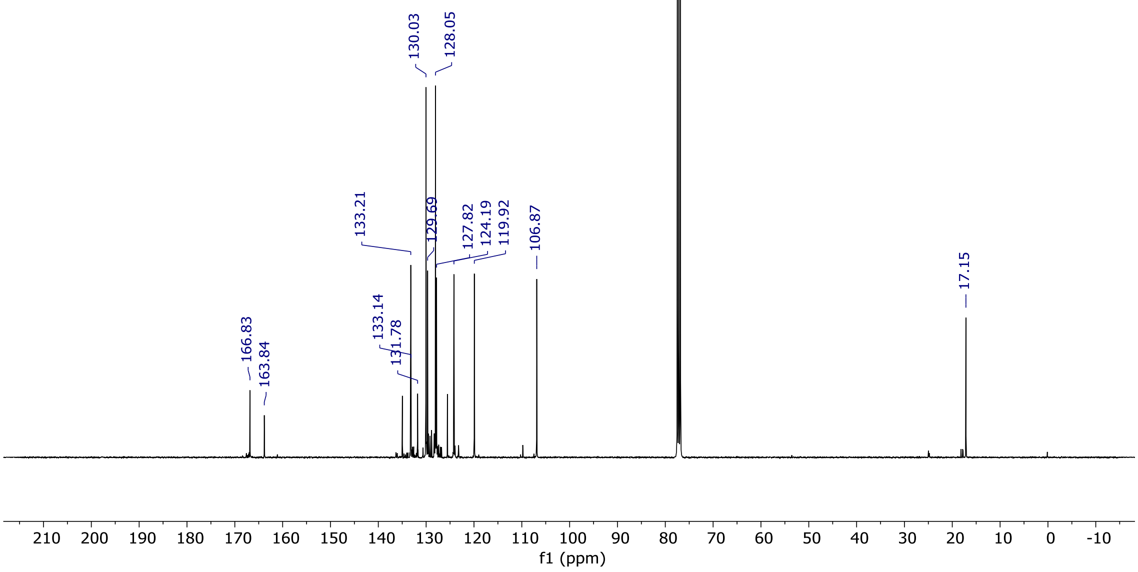

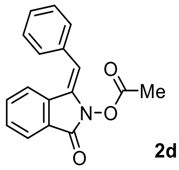

2d

2D-COSY

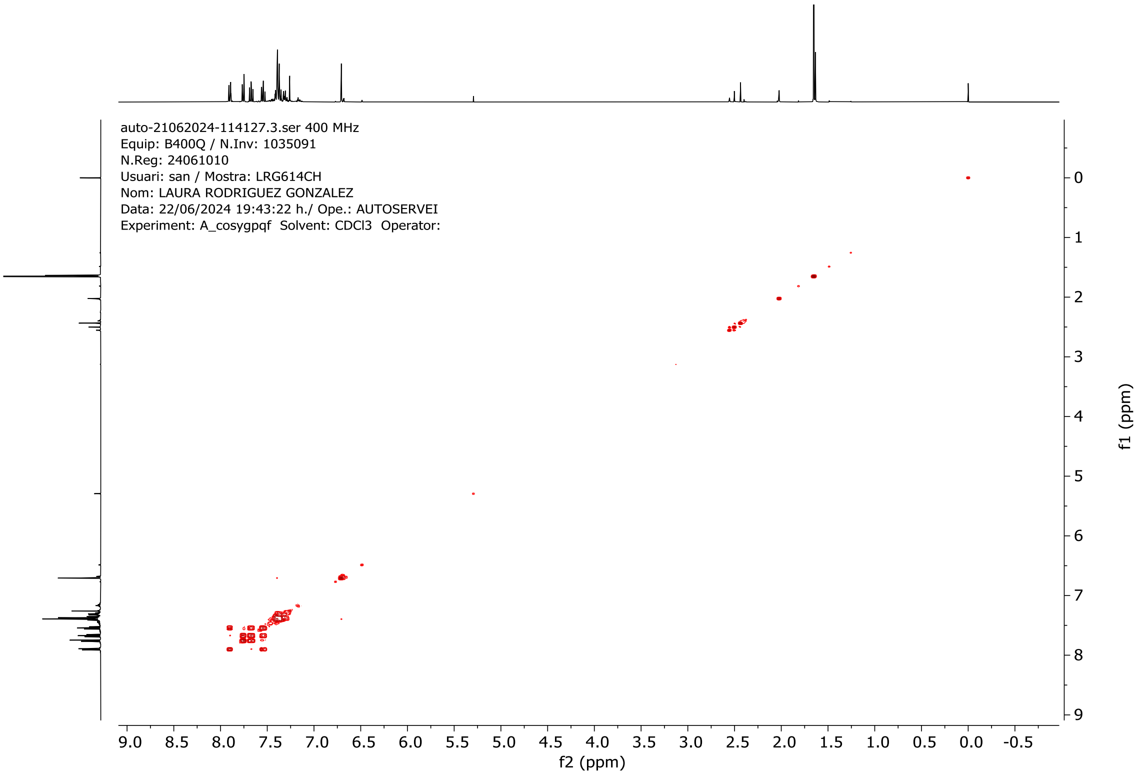

2D-HSQC

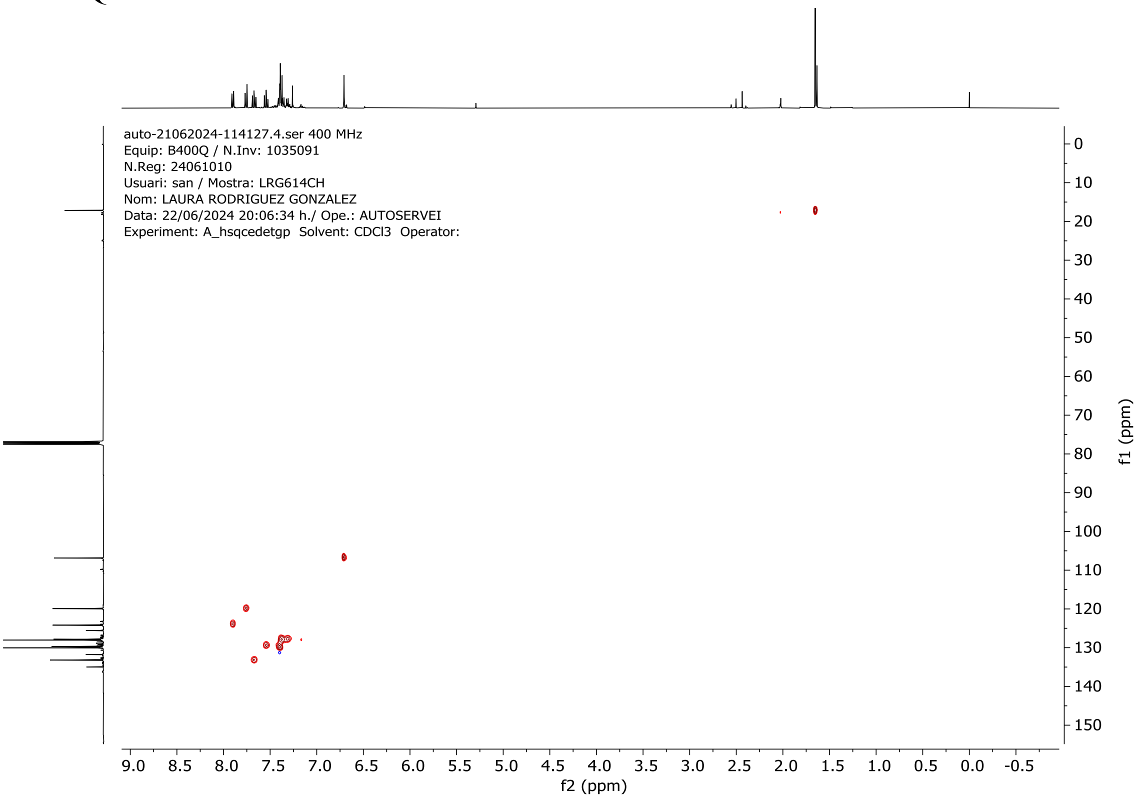

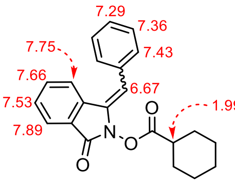

2e

ASV030-19-24.1.fid 1H 400 MHz
Equip: B400F / N.Inv: 1037597
N.Reg: 24030800
Usuari: san / Mostra: ASV030-19-24
Nom: AINA SERRA VERT
Data: 20/03/2024 09:48:45 h./ Ope.: AUTOSERVEI
Experiment: A-H1-zg30 Solvent: CDCl3

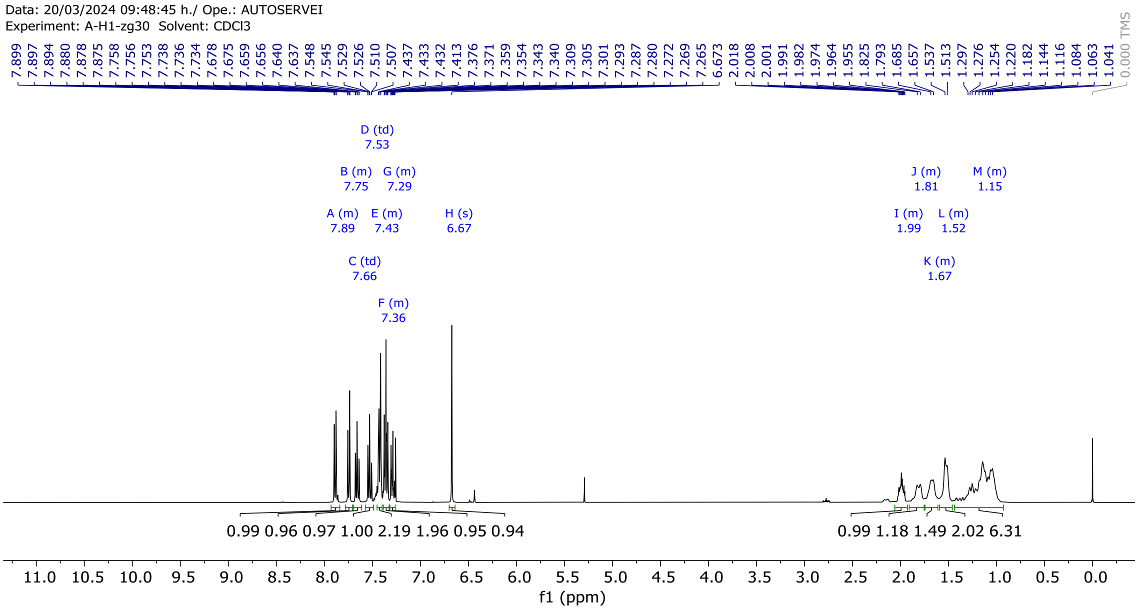

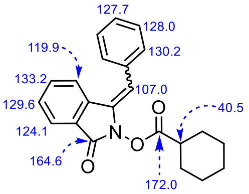

2e

ASV030-19-24.2.fid 13C{1H} 101 MHz
Equip: B400F / N.Inv: 1037597
N.Reg: 24030800
Usuari: san / Mostra: ASV030-19-24
Nom: AINA SERRA VERT
Data: 20/03/2024 23:25:24 h./ Ope.: AUTOSERVEI
Experiment: A-C13-zgpg30 Solvent: CDCl3

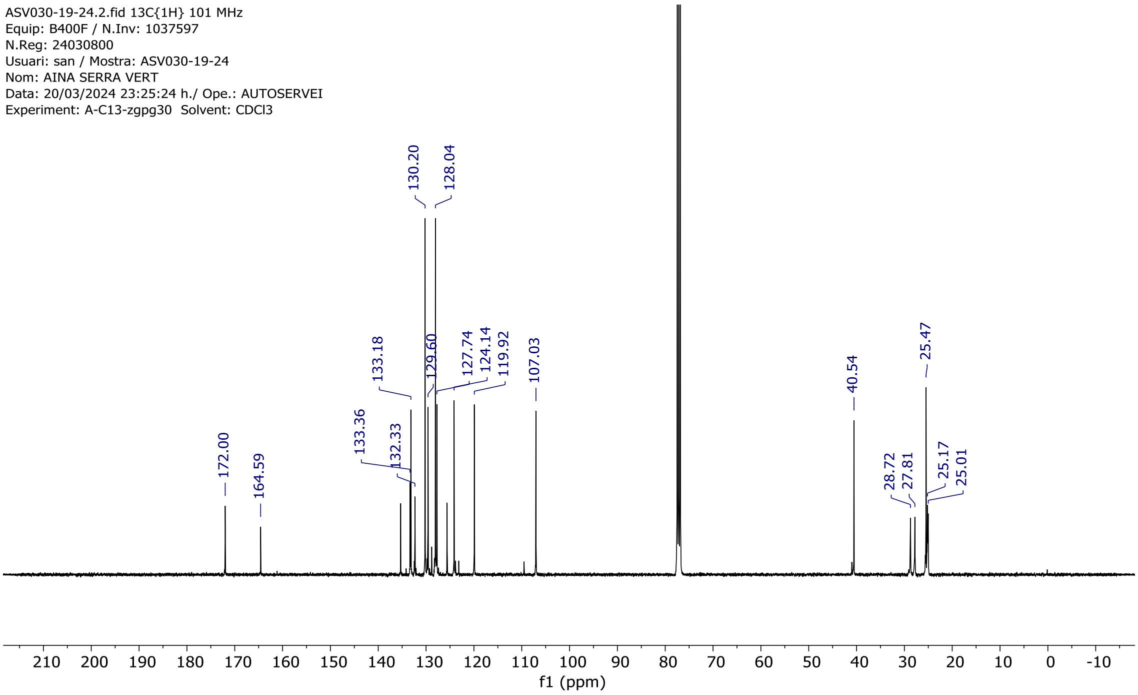

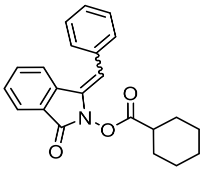

2e

2D-COSY

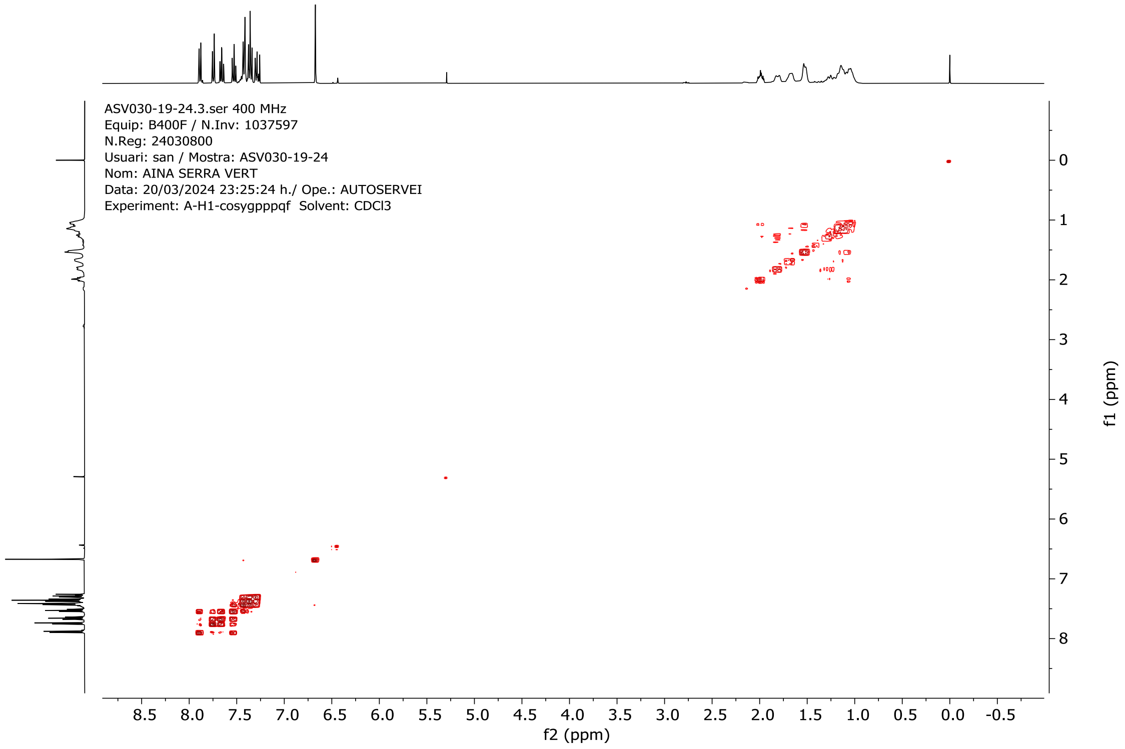

2D-HSQC

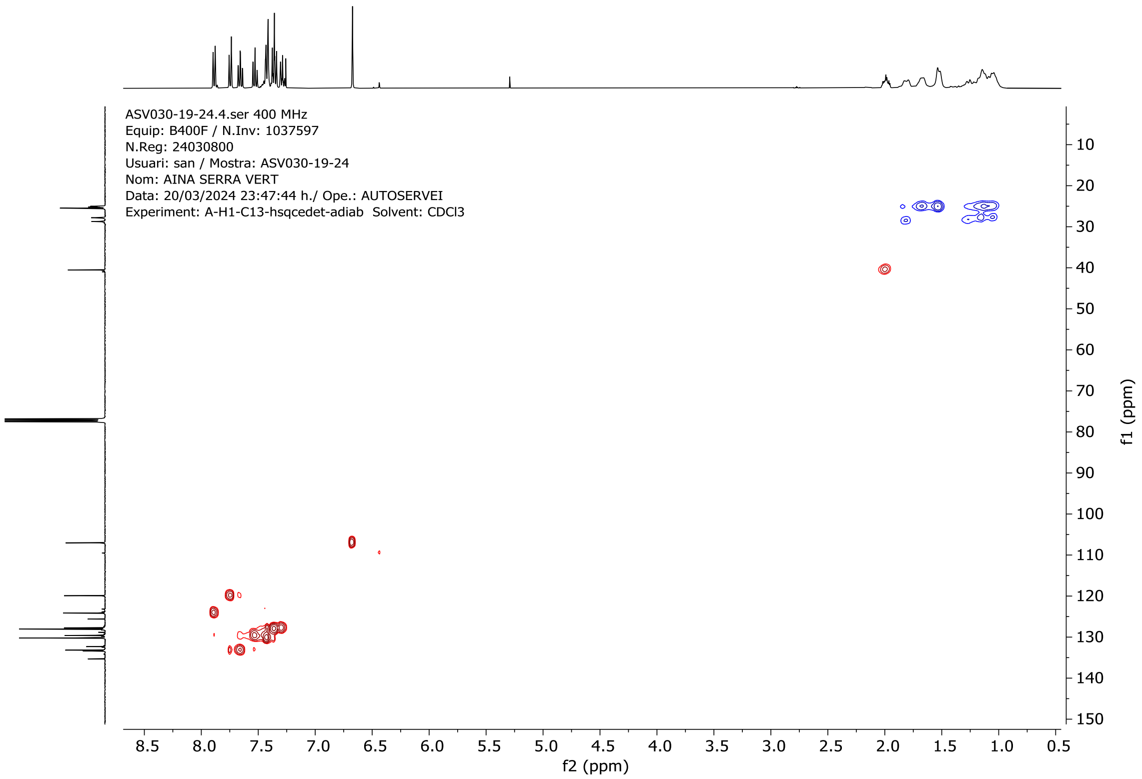

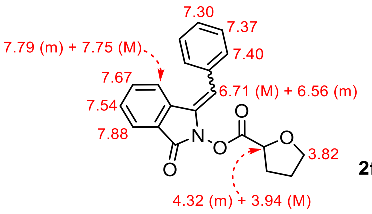

2f

24040948_B400FA_22042024_ASV036-2-23-24.1.fid 1H 400 MHz
Equip: B400F / N.Inv: 1037597
N.Reg: 24040948
Usuari: san / Mostra: ASV036-2-23-24
Nom: AINA SERRA VERT
Data: 22/04/2024 12:30:06 h./ Ope.: AUTOSERVEI
Experiment: A-H1-zg30 Solvent: CDCl3

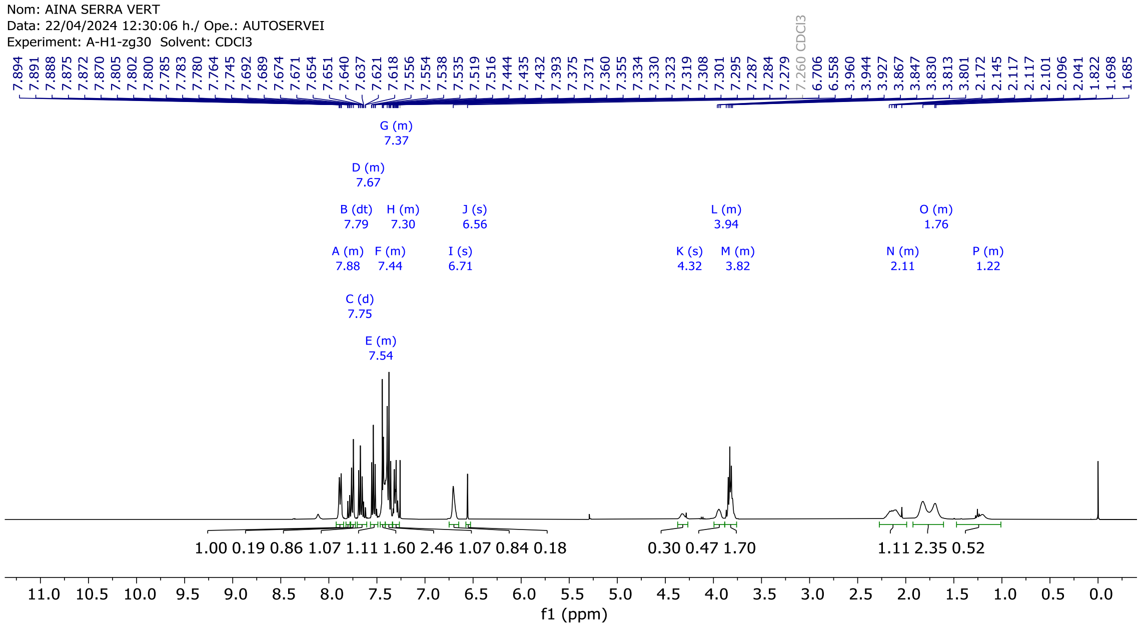

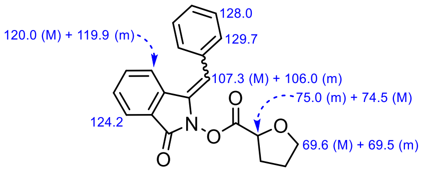

2f

24040948_B400FA_22042024_ASV036-2-23-24.2.fid 13C{1H} 101 MHz
Equip: B400F / N.Inv: 1037597
N.Reg: 24040948
Usuari: san / Mostra: ASV036-2-23-24
Nom: AINA SERRA VERT
Data: 22/04/2024 21:08:20 h./ Ope.: AUTOSERVEI
Experiment: A-C13-zgpg30 Solvent: CDCl3

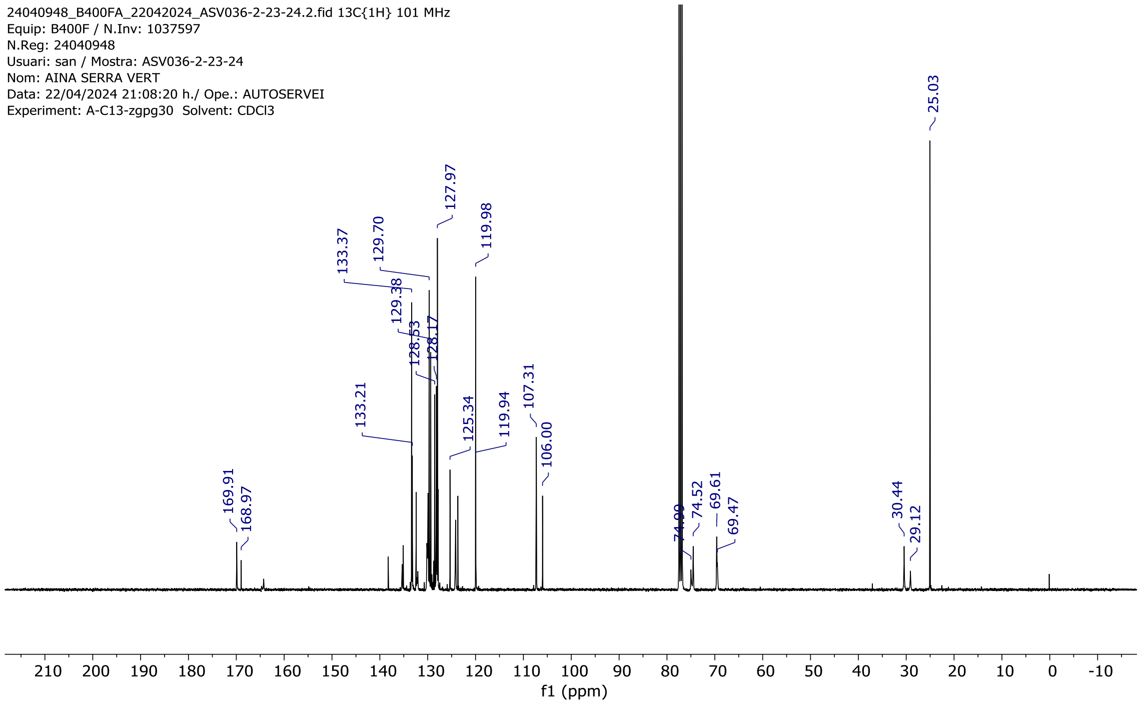

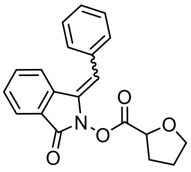

2f

2D-COSY

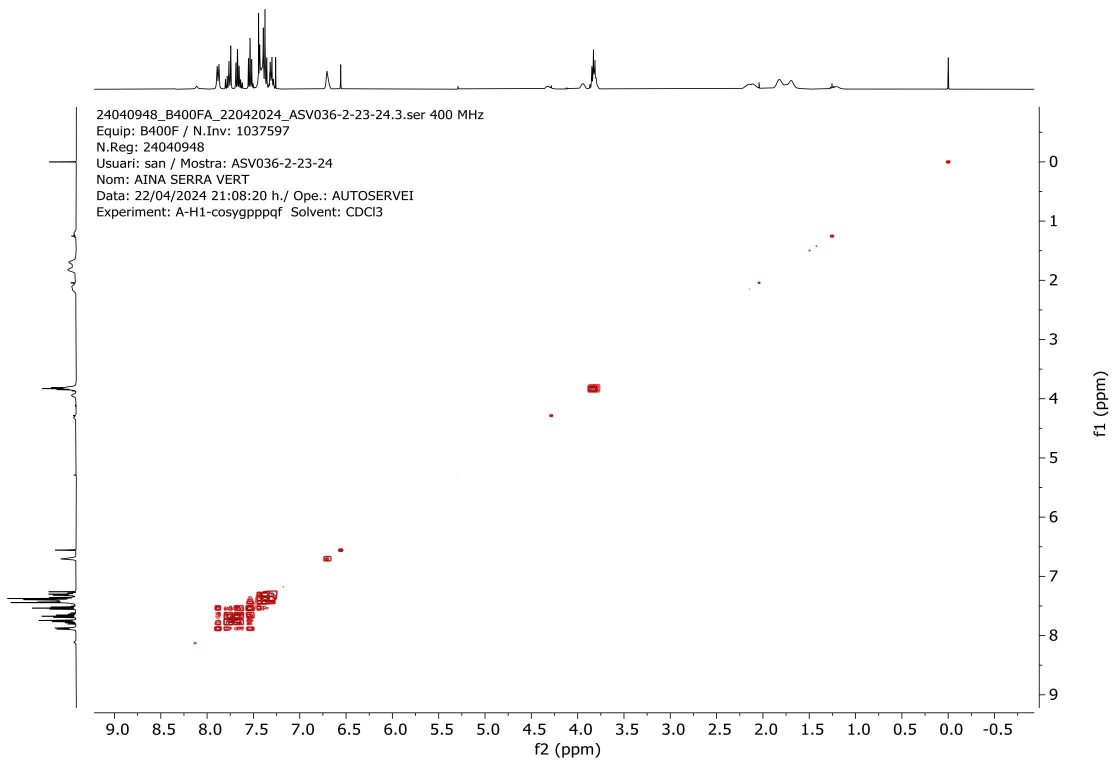

2D-HSQC

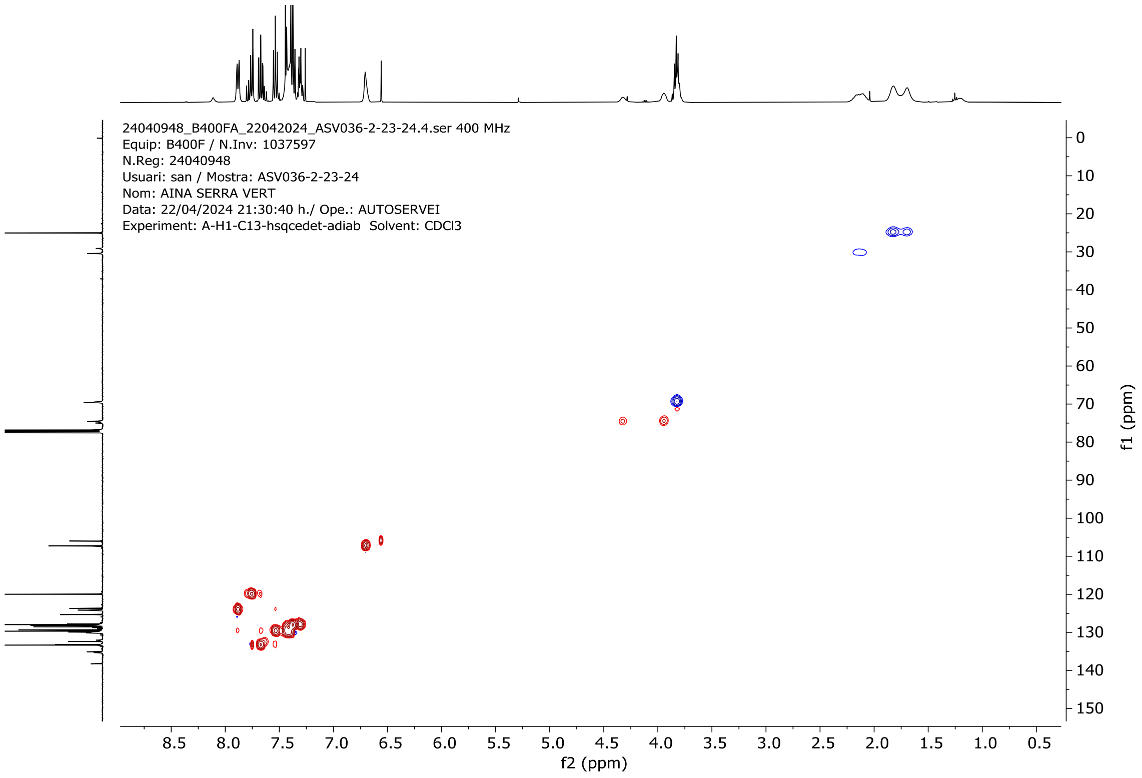

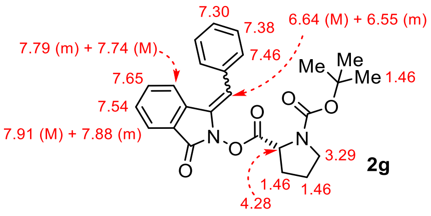

2g

LRG531T29DRY.1.fid 1H 400 MHz
Equip: B400F / N.Inv: 1037597
N.Reg: 24020028
Usuari: san / Mostra: LRG531T29DRY
Nom: LAURA RODRIGUEZ GONZALEZ
Data: 01/02/2024 15:44:51 h./ Ope.: AUTOSERVEI
Experiment: A-H1-zg30 Solvent: CDCl3

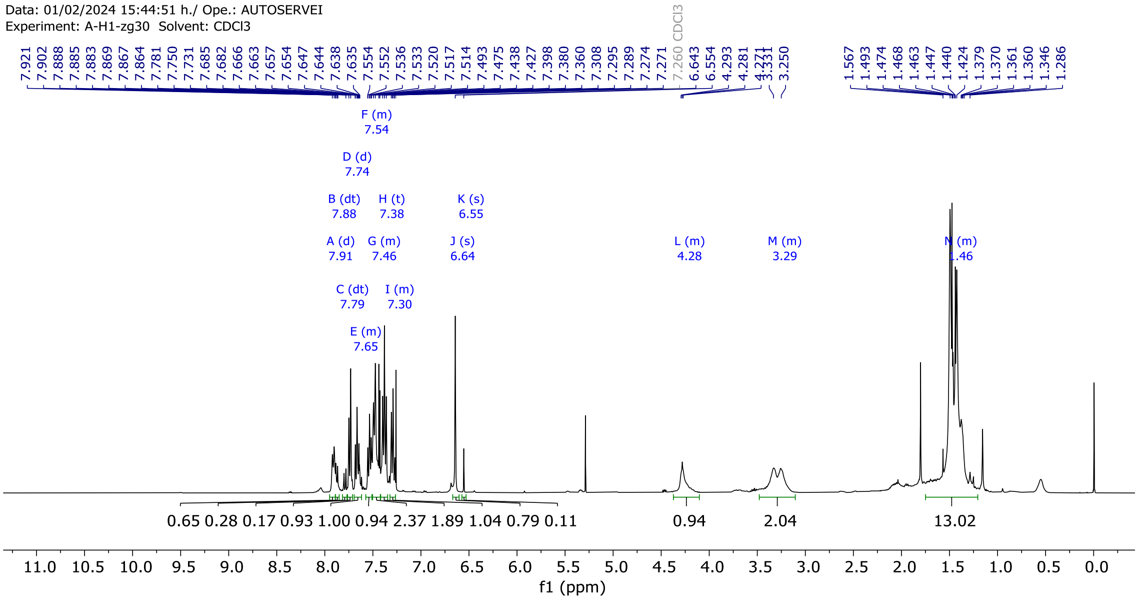

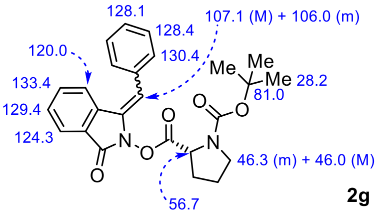

2g

LRG531CH.2.fid 13C{1H} 101 MHz
Equip: B400F / N.Inv: 1037597
N.Reg: 24020001
Usuari: san / Mostra: LRG531CH
Nom: LAURA RODRIGUEZ GONZALEZ
Data: 01/02/2024 21:14:25 h./ Ope.: AUTOSERVEI
Experiment: A-C13-zgpg30 Solvent: CDCl3

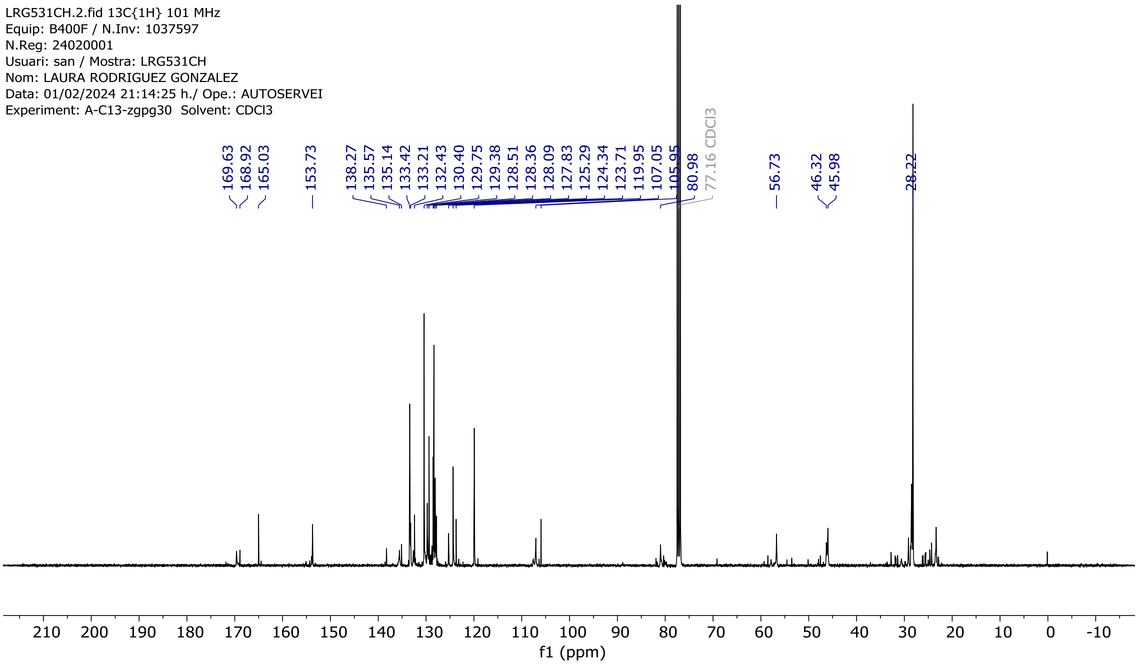

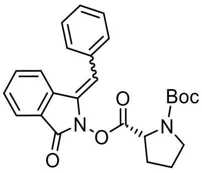

2g

2D-COSY

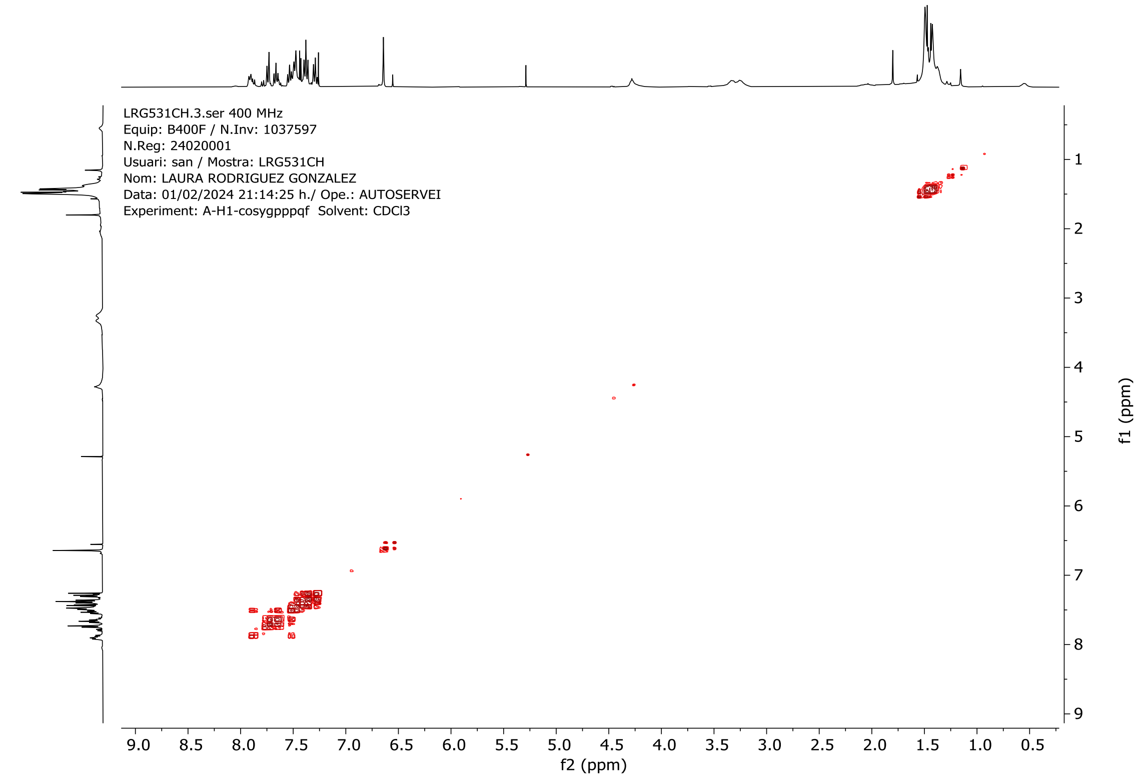

2D-HSQC

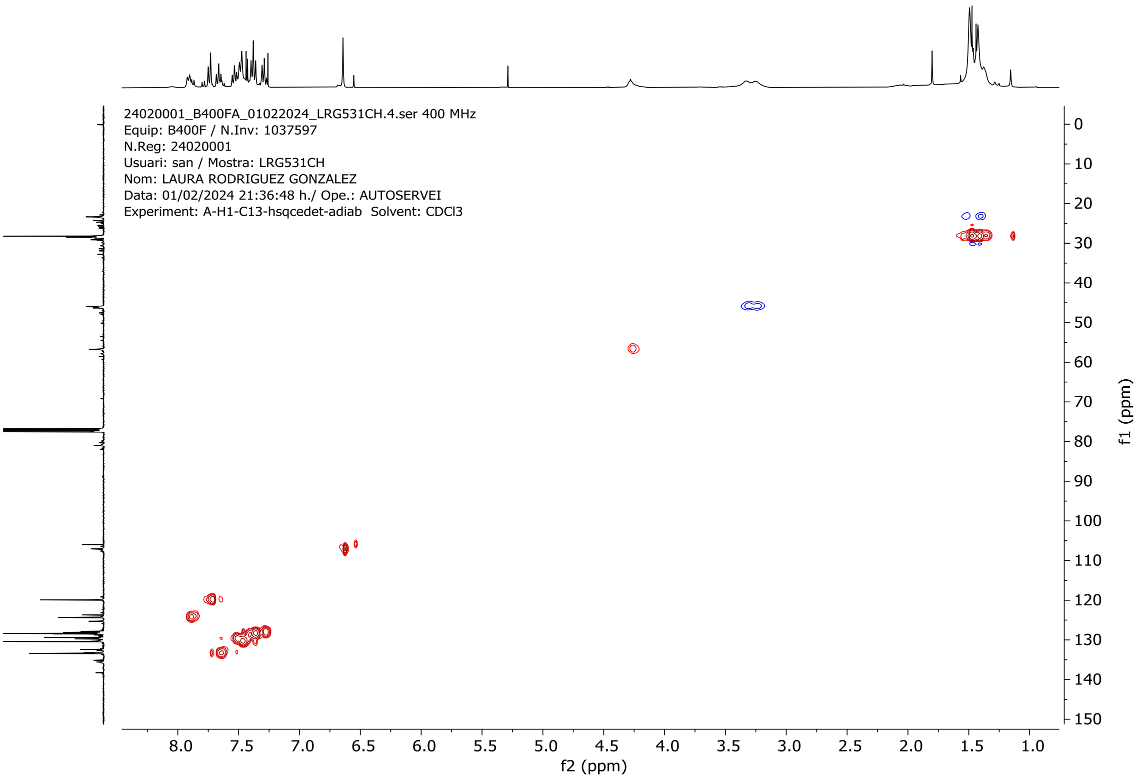

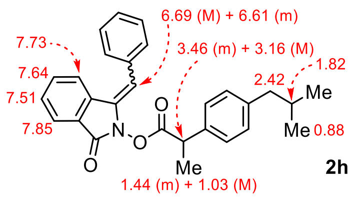

2h

24050623_B400FA_16052024_ASV054-PREP.1.fid 1H 400 MHz
Equip: B400F / N.Inv: 1037597
N.Reg: 24050623
Usuari: san / Mostra: ASV054-PREP
Nom: AINA SERRA VERT
Data: 15/05/2024 15:28:25 h./ Ope.: AUTOSERVEI
Experiment: A-H1-zg30 Solvent: CDCl3

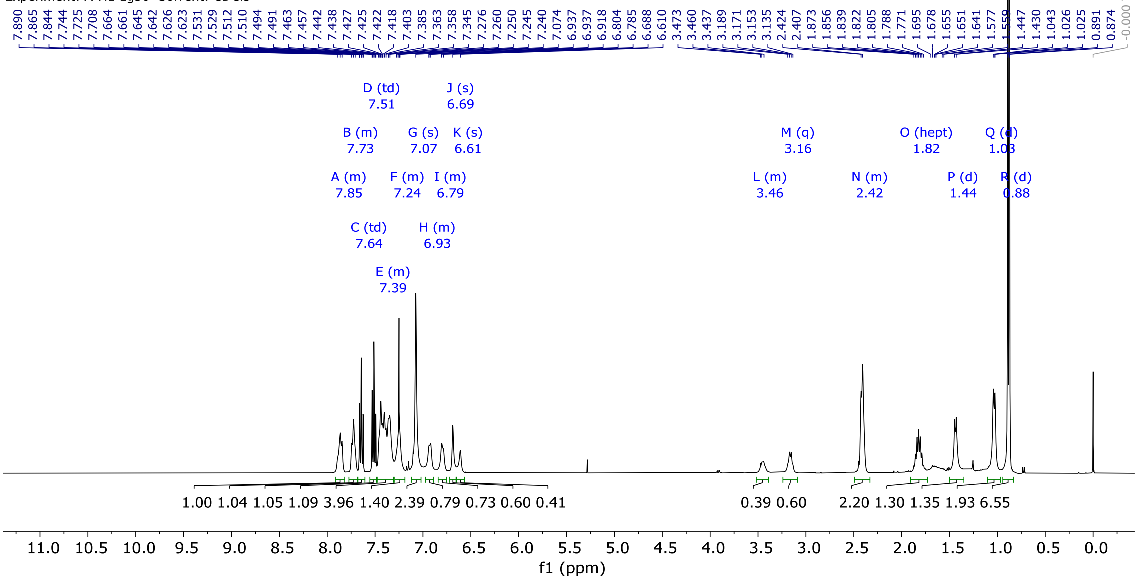

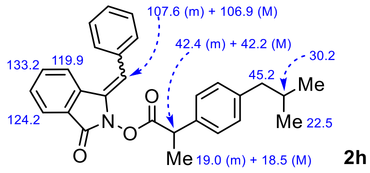

2h

24050623_B400FA_16052024_ASV054-PREP.2.fid 13C{1H} 101 MHz
Equip: B400F / N.Inv: 1037597
N.Reg: 24050623
Usuari: san / Mostra: ASV054-PREP
Nom: AINA SERRA VERT
Data: 16/05/2024 01:08:00 h./ Ope.: AUTOSERVEI
Experiment: A-C13-zgpg30 Solvent: CDCl3

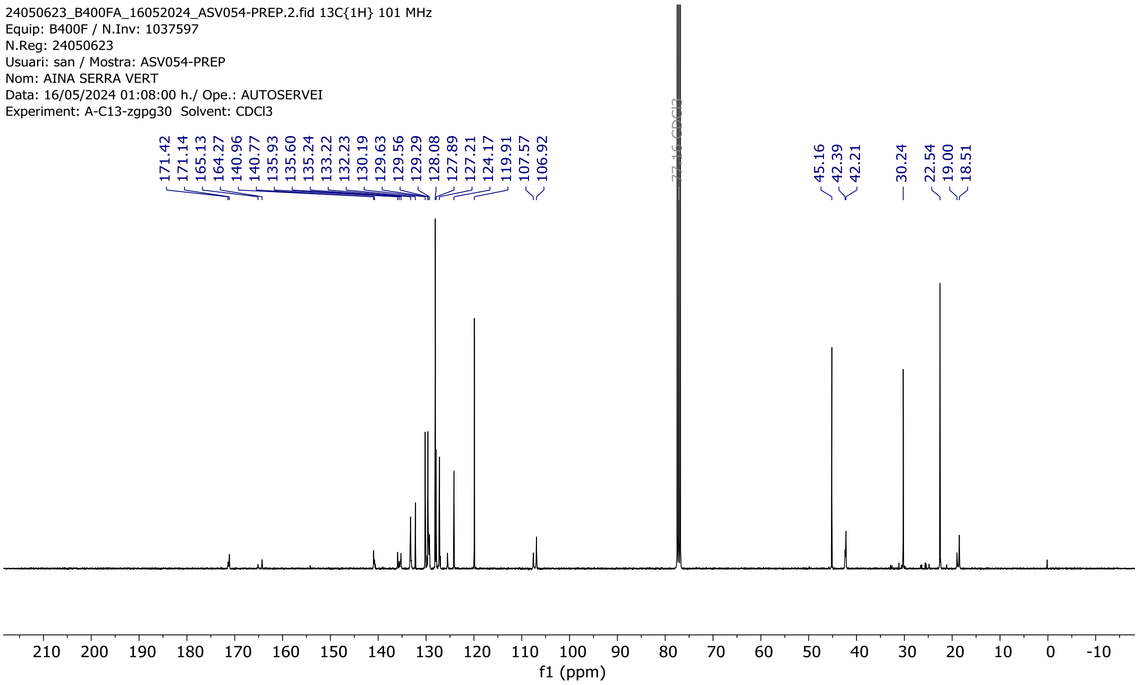

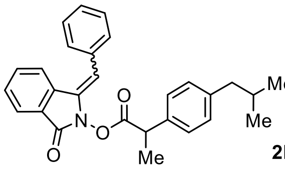

2h

2D-COSY

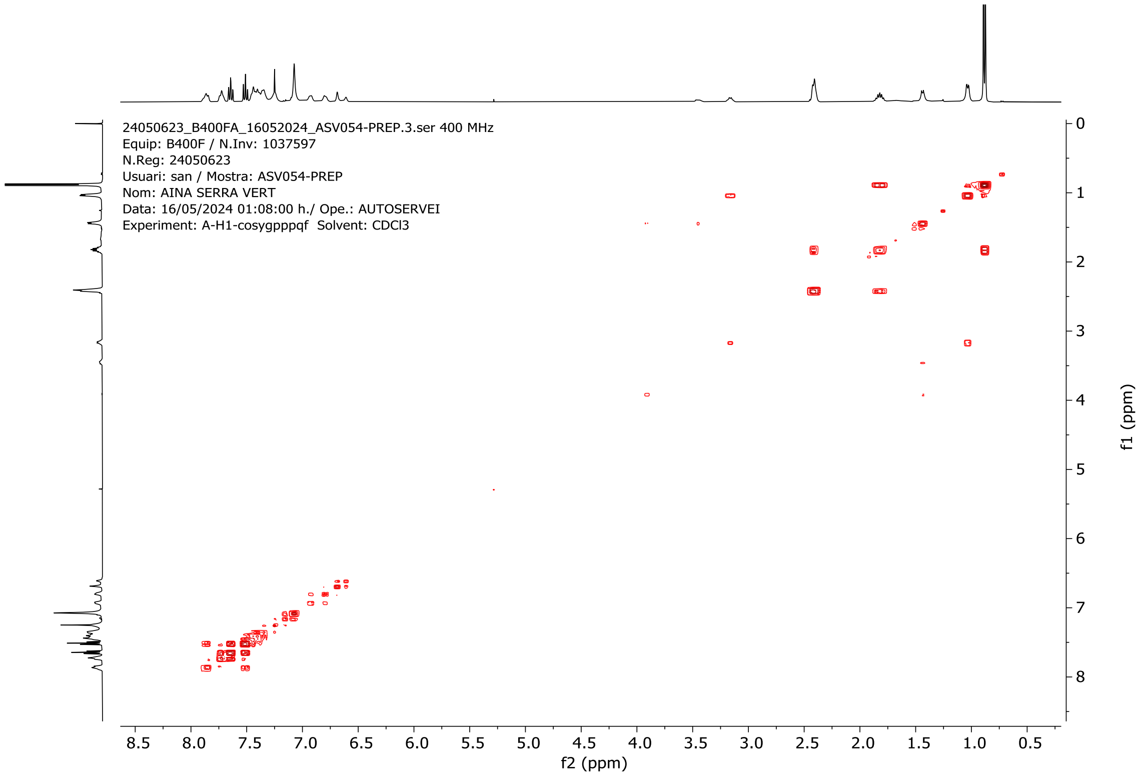

2D-HSQC

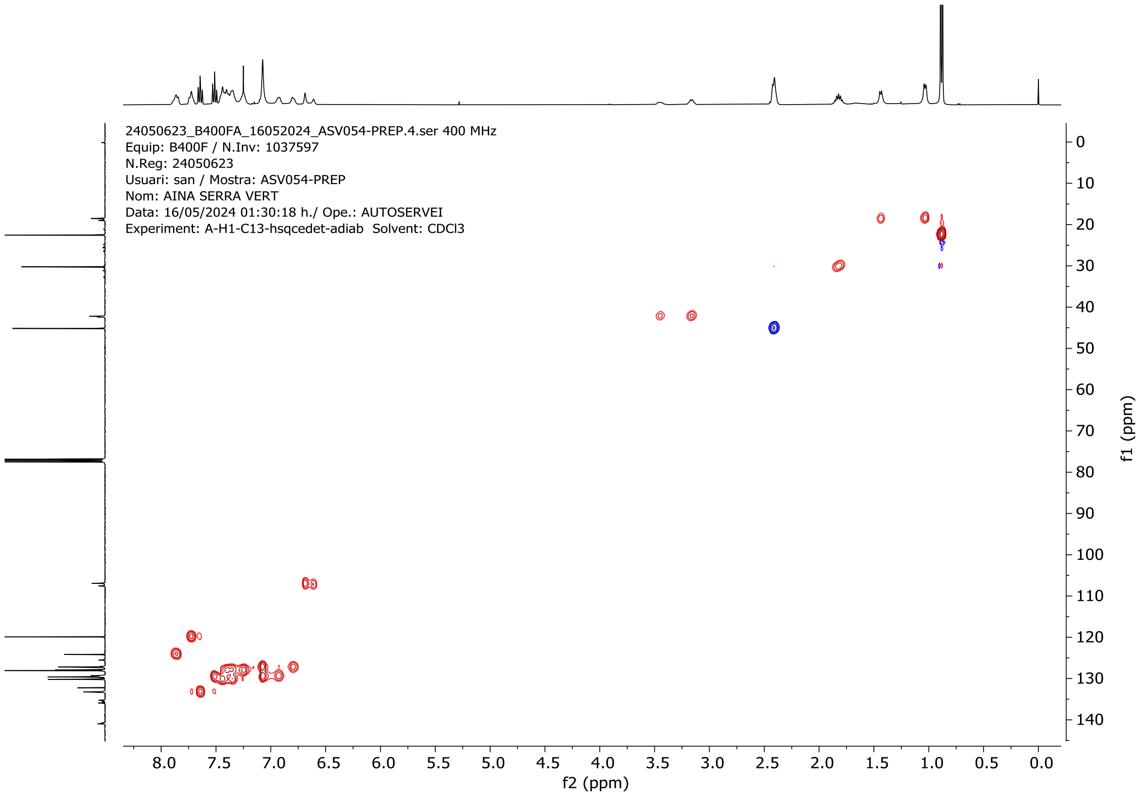

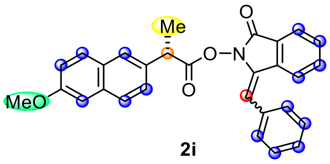

2i

This product was isolated as a complex mixture of isomers and rotamers that made it impossible to interpret de ^{13}C spectrum. Therefore, only the data corresponding to the ^{1}H NMR (400 MHz) spectrum is reported herein.

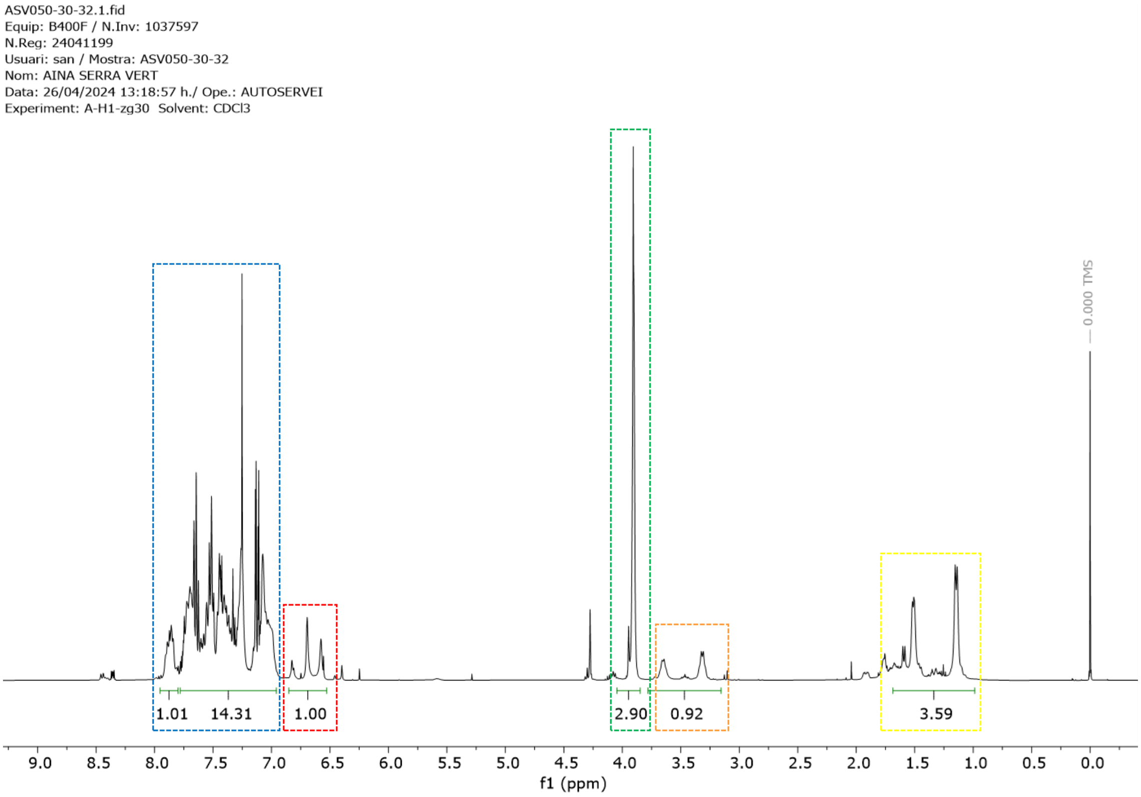

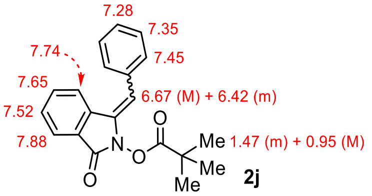

2j

ASV031-21-24CH.1.fid 1H 400 MHz
Equip: B400F / N.Inv: 1037597
N.Reg: 24030897
Usuari: san / Mostra: ASV031-21-24
Nom: AINA SERRA VERT
Data: 21/03/2024 14:28:39 h./ Ope.: AUTOSERVEI
Experiment: A-H1-zg30 Solvent: CDCl3

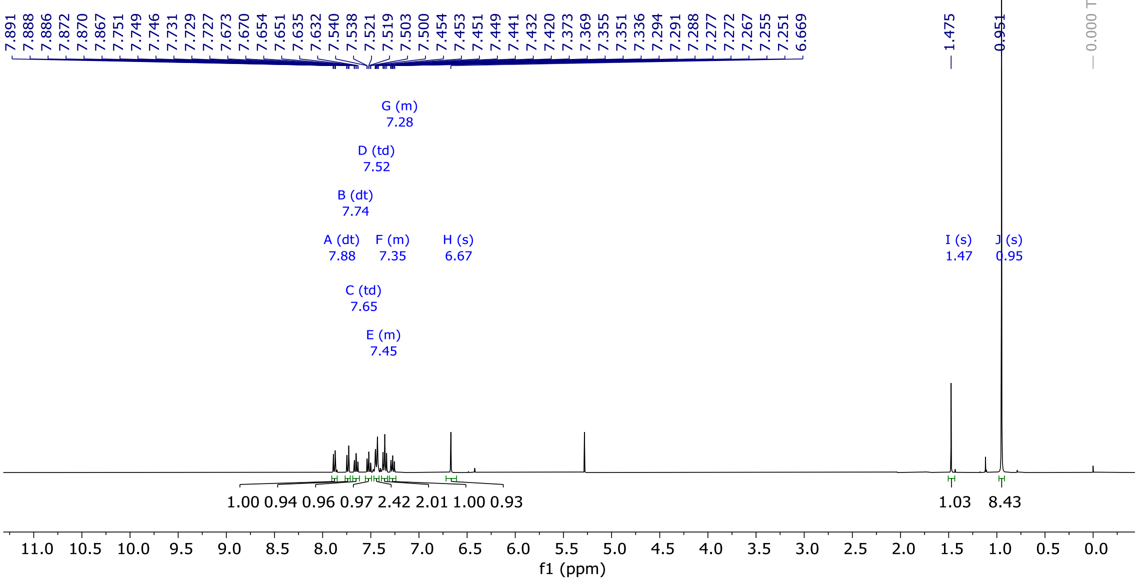

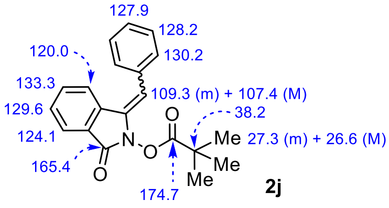

2j

ASV031-21-24CH.2.fid 13C{1H} 101 MHz
Equip: B400F / N.Inv: 1037597
N.Reg: 24030897
Usuari: san / Mostra: ASV031-21-24
Nom: AINA SERRA VERT
Data: 22/03/2024 00:01:22 h./ Ope.: AUTOSERVEI
Experiment: A-C13-zgpg30 Solvent: CDCl3

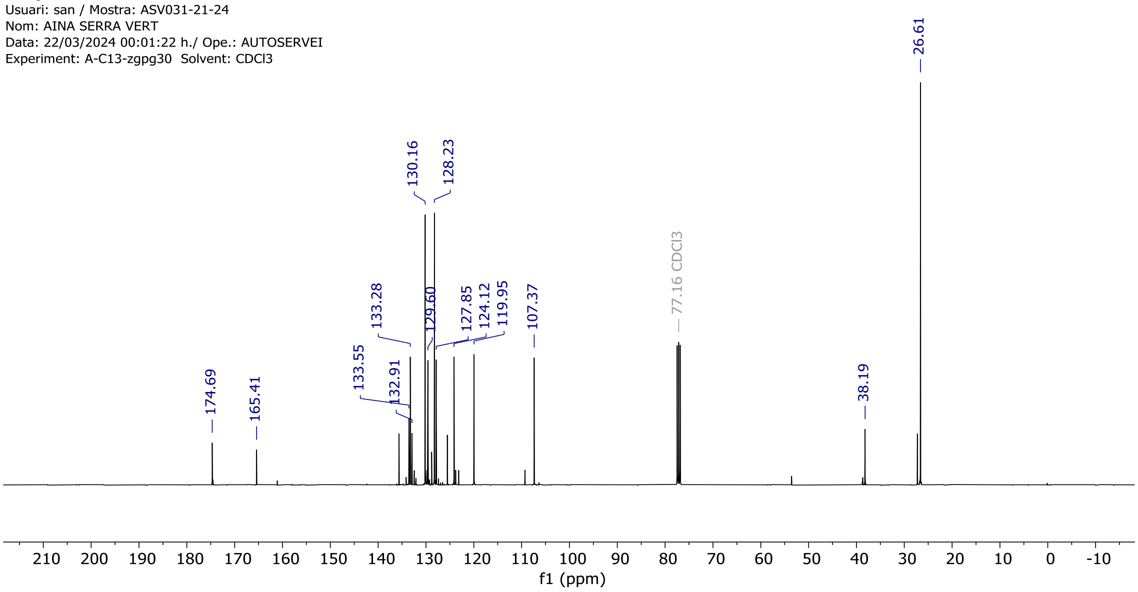

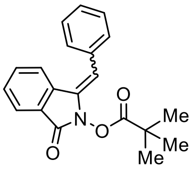

2j

2D-COSY

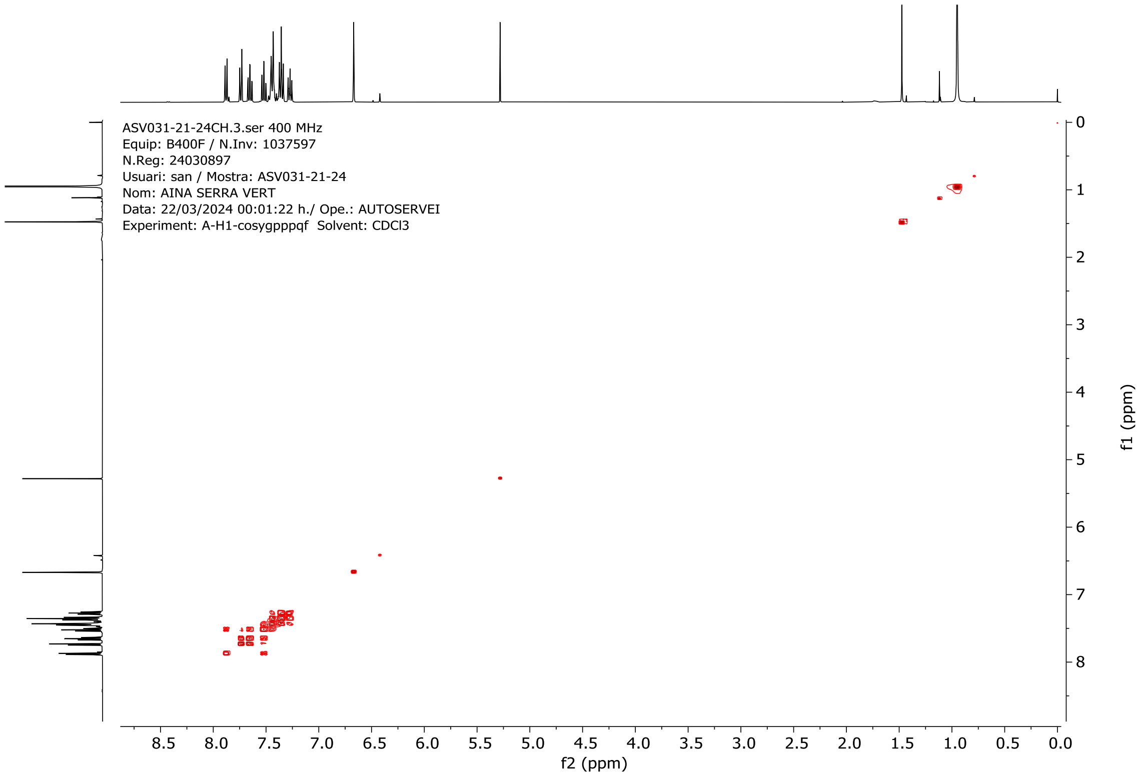

2D-HSQC

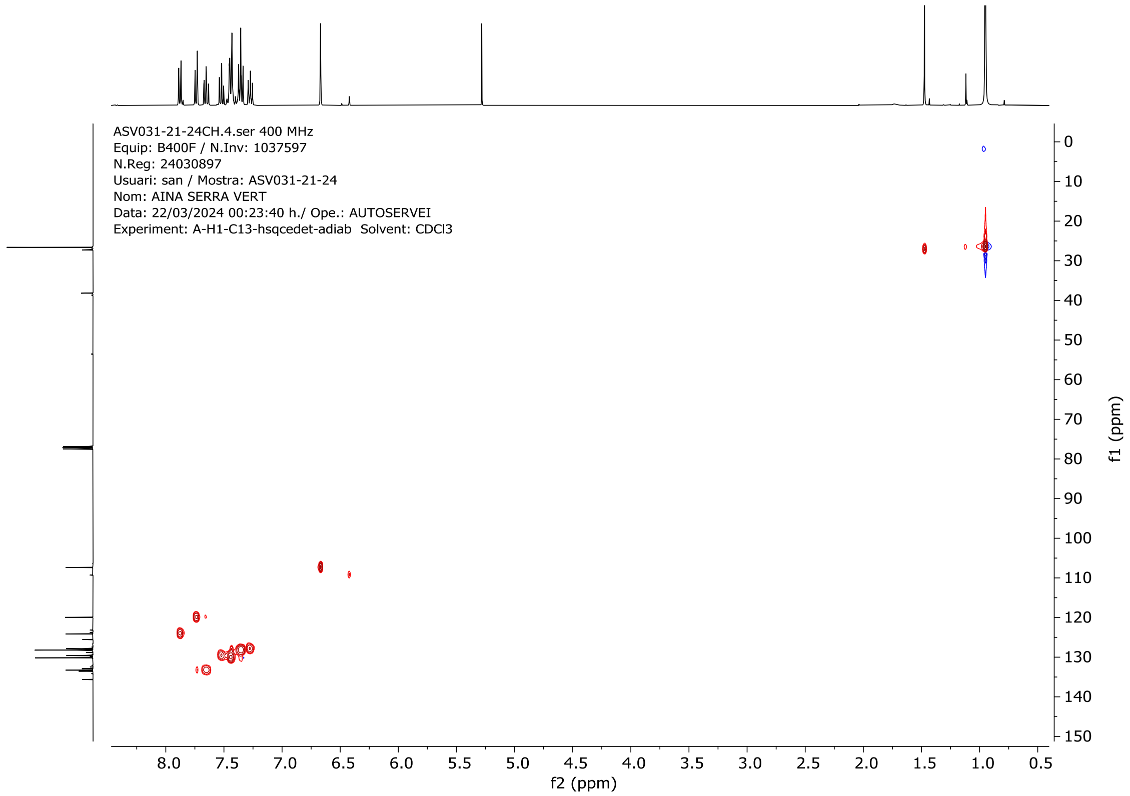

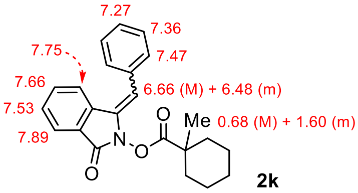

2k

24060403_B400FA_14062024_LRG611DRY.1.fid 1H 400 MHz
Equip: B400F / N.Inv: 1037597
N.Reg: 24060403
Usuari: san / Mostra: LRG611DRY
Nom: LAURA RODRIGUEZ GONZALEZ
Data: 14/06/2024 13:06:33 h./ Ope.: AUTOSERVEI
Experiment: A-H1-zg30 Solvent: CDCl3

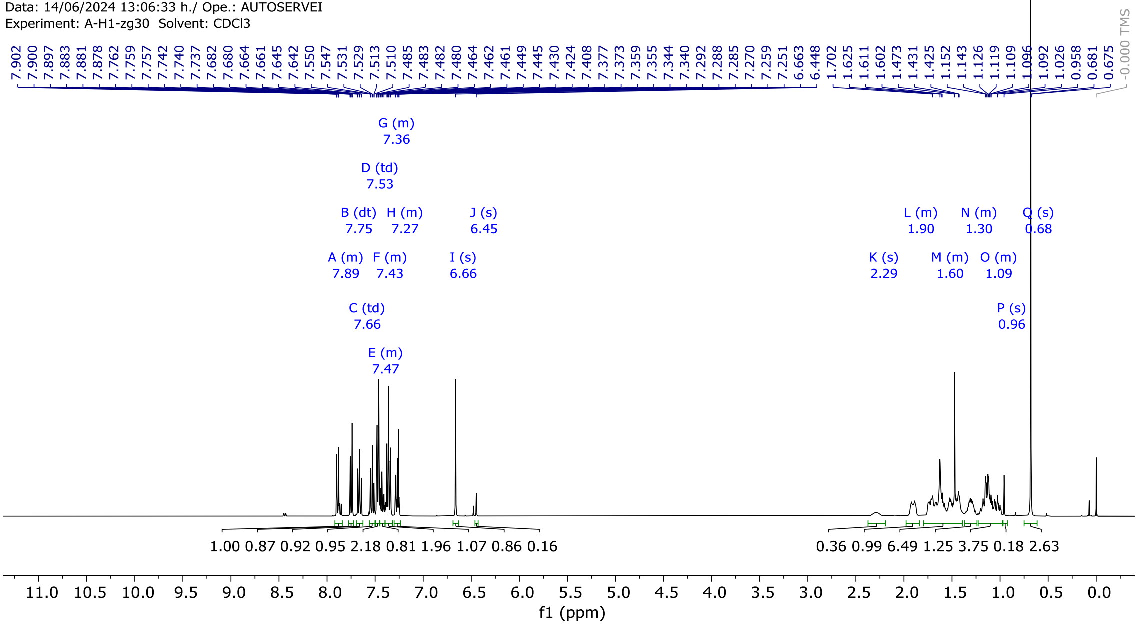

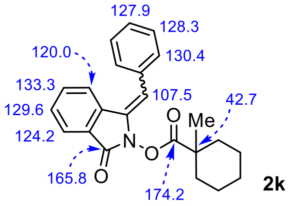

2k

24060346_B400FA_14062024_LRG611COLT21T23.2.fid 13C{1H} 101 MHz
Equip: B400F / N.Inv: 1037597
N.Reg: 24060346
Usuari: san / Mostra: LRG611COLT21T23
Nom: LAURA RODRIGUEZ GONZALEZ
Data: 14/06/2024 05:05:11 h./ Ope.: AUTOSERVEI
Experiment: A-C13-zgpg30 Solvent: CDCl3

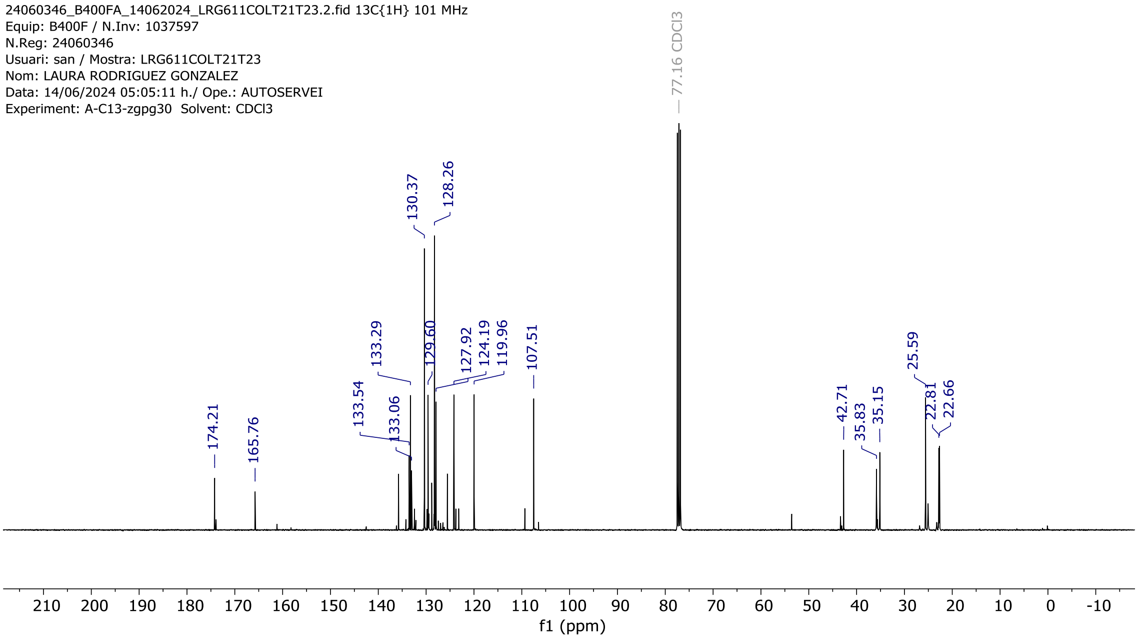

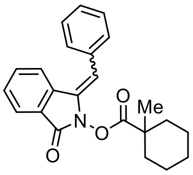

2k

2D-COSY

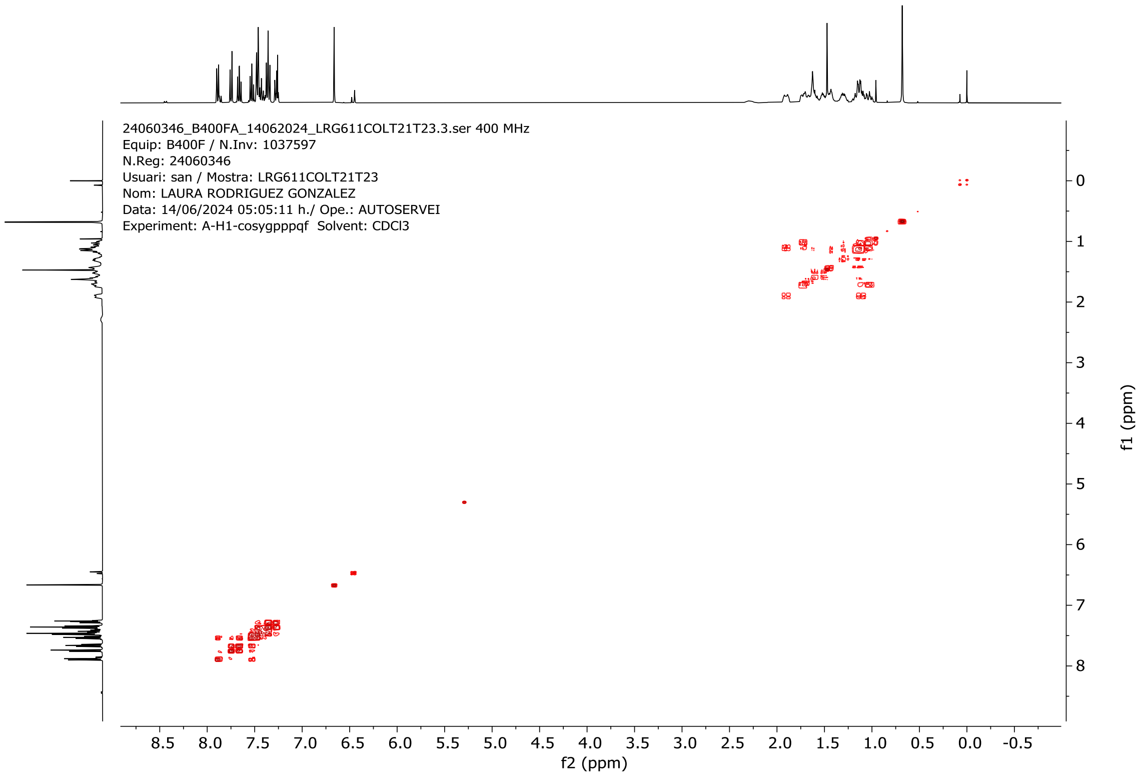

2D-HSQC

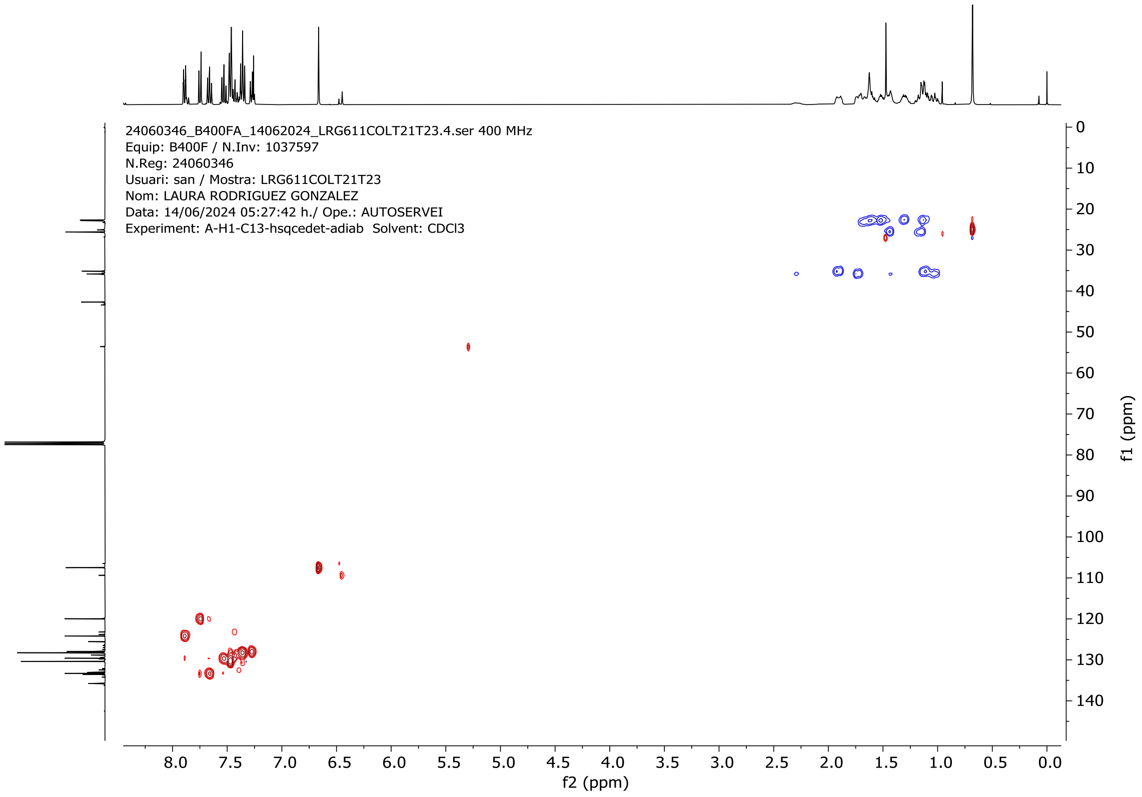

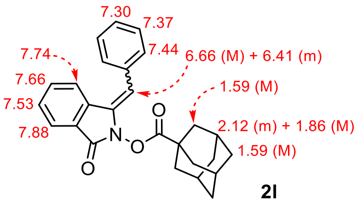

2l

24041075_B400FA_24042024_ASV048-19-21.1.fid 1H 400 MHz
Equip: B400F / N.Inv: 1037597
N.Reg: 24041075
Usuari: san / Mostra: ASV048-19-21
Nom: AINA SERRA VERT
Data: 24/04/2024 13:15:16 h./ Ope.: AUTOSERVEI
Experiment: A-H1-zg30 Solvent: CDCl3

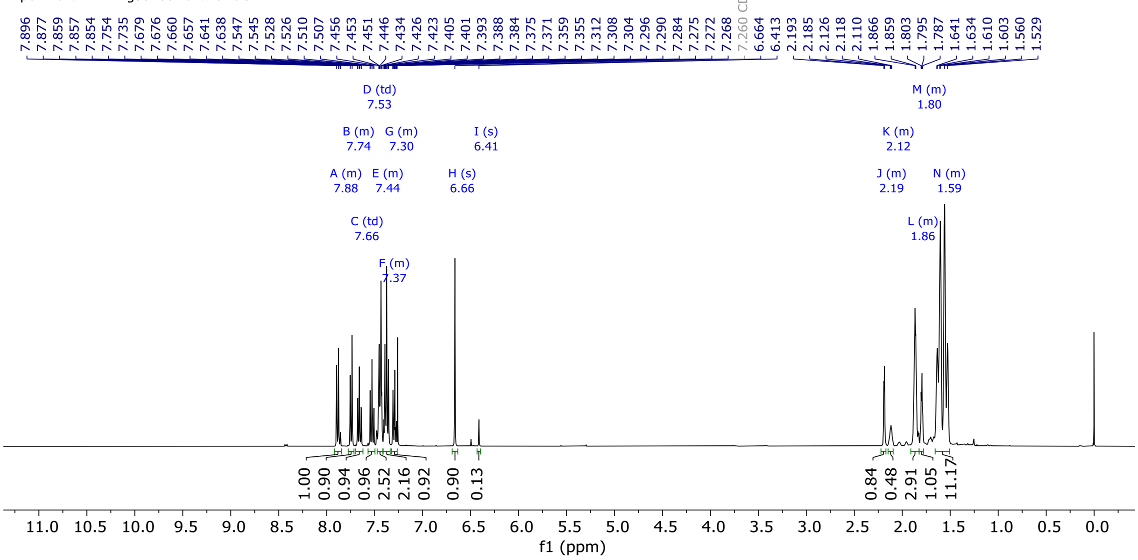

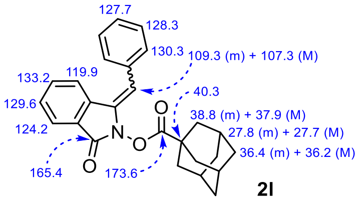

2l

24041075_B400FA_24042024_ASV048-19-21.2.fid 13C{1H} 101 MHz
Equip: B400F / N.Inv: 1037597
N.Reg: 24041075
Usuari: san / Mostra: ASV048-19-21
Nom: AINA SERRA VERT
Data: 24/04/2024 21:08:44 h./ Ope.: AUTOSERVEI
Experiment: A-C13-zgpg30 Solvent: CDCl3

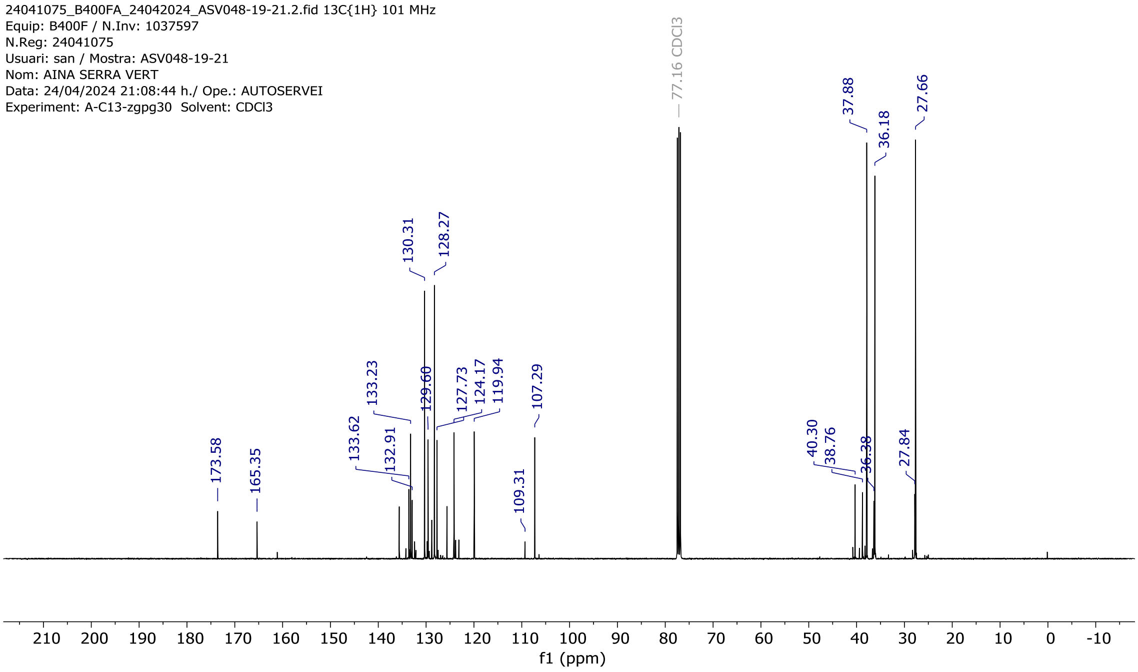

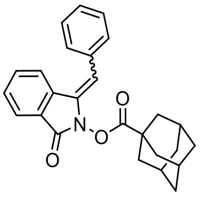

2l

2D-COSY

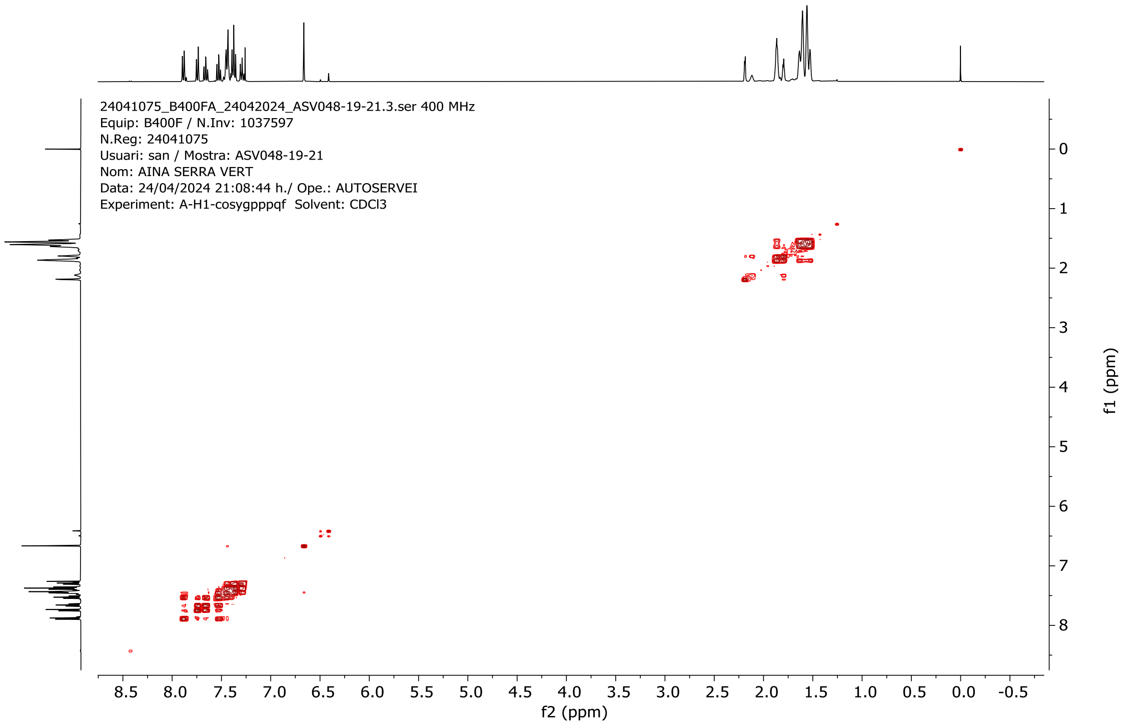

2D-HSQC

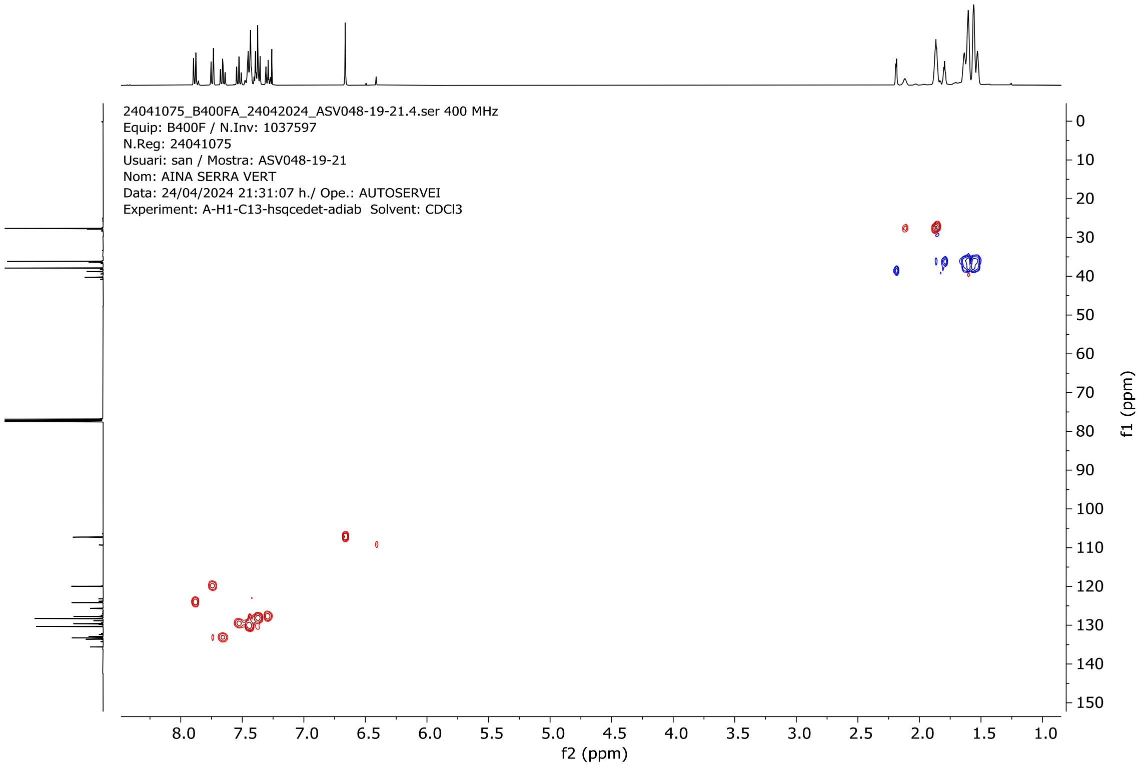

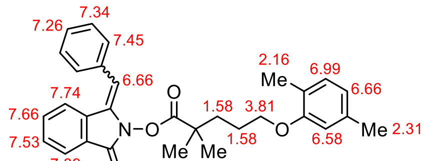

2m

24050020_B400FA_02052024_ASV052T22.1.fid 1H 400 MHz
Equip: B400F / N.Inv: 1037597
N.Reg: 24050020
Usuari: san / Mostra: ASV052T22
Nom: LAURA RODRIGUEZ GONZALEZ
Data: 02/05/2024 13:51:02 h./ Ope.: AUTOSERVEI
Experiment: A-H1-zg30 Solvent: CDCl3

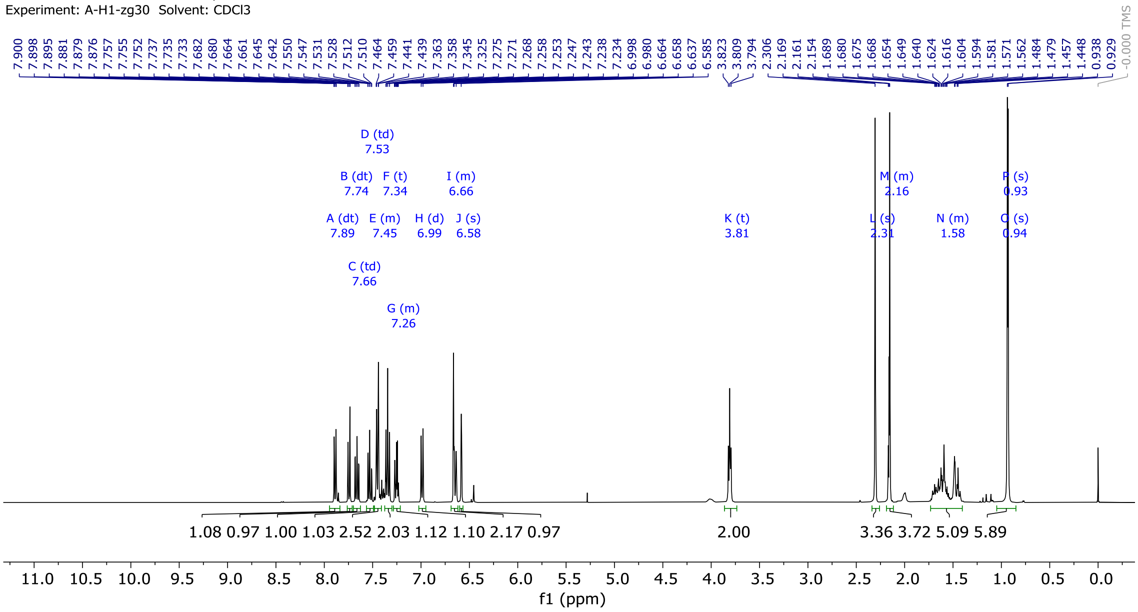

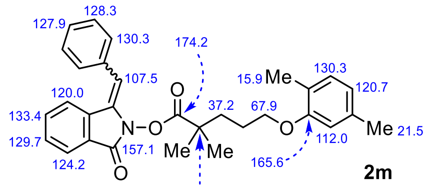

2m

24050033_B400FA_03052024_ASV052-22-24.2.fid 13C{1H} 101 MHz
Equip: B400F / N.Inv: 1037597
N.Reg: 24050033
Usuari: san / Mostra: ASV052-22-24
Nom: AINA SERRA VERT
Data: 03/05/2024 05:19:14 h./ Ope.: AUTOSERVEI
Experiment: A-C13-zgpg30 Solvent: CDCl3

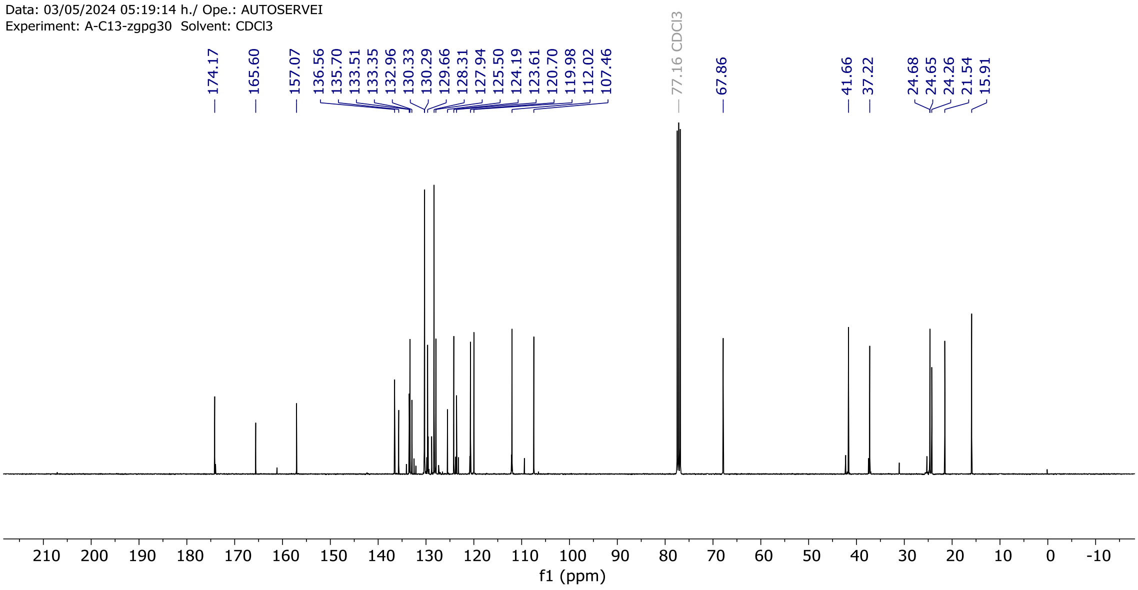

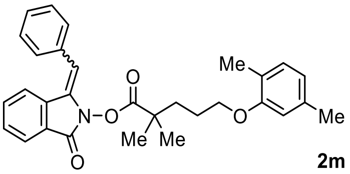

2m

2D-COSY

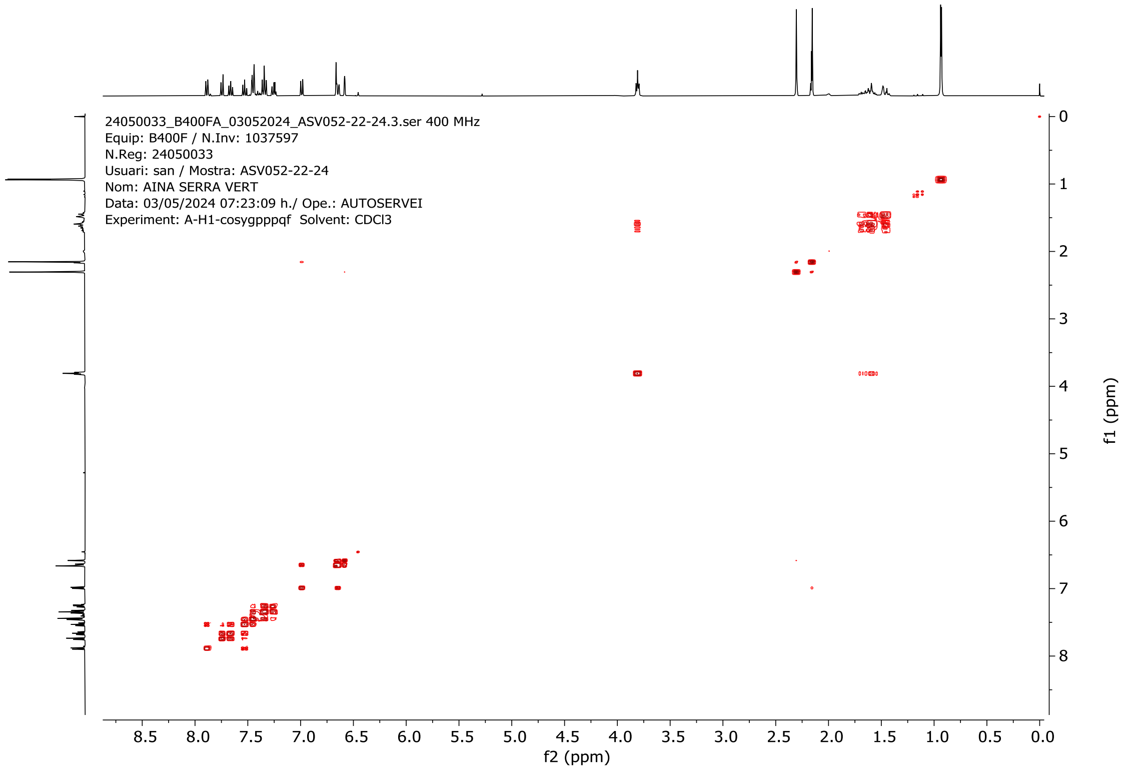

2D-HSQC

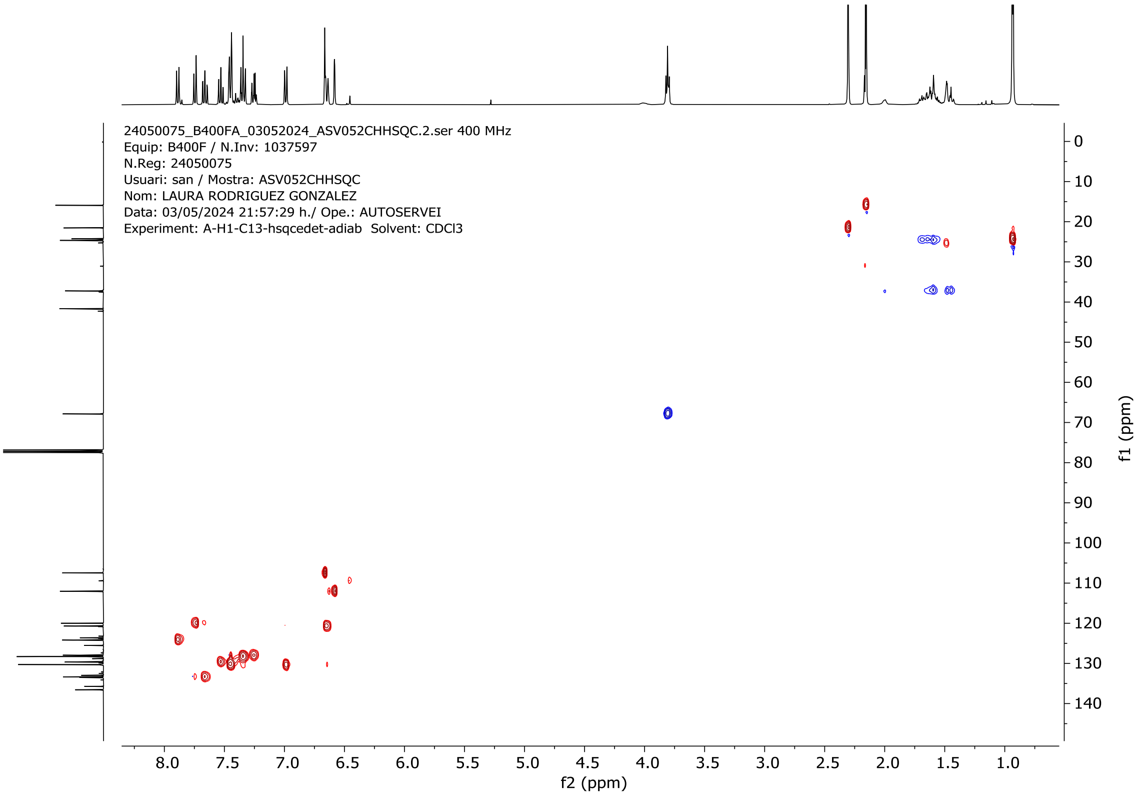

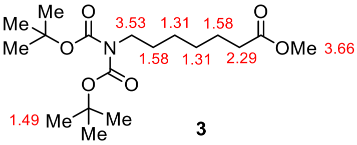

3

24030080_B400FA_04032024_LRG554PREP.1.fid 1H 400 MHz
Equip: B400F / N.Inv: 1037597
N.Reg: 24030080
Usuari: san / Mostra: LRG554PREP
Nom: LAURA RODRIGUEZ GONZALEZ
Data: 04/03/2024 16:50:23 h./ Ope.: AUTOSERVEI
Experiment: A-H1-zg30 Solvent: CDCl3

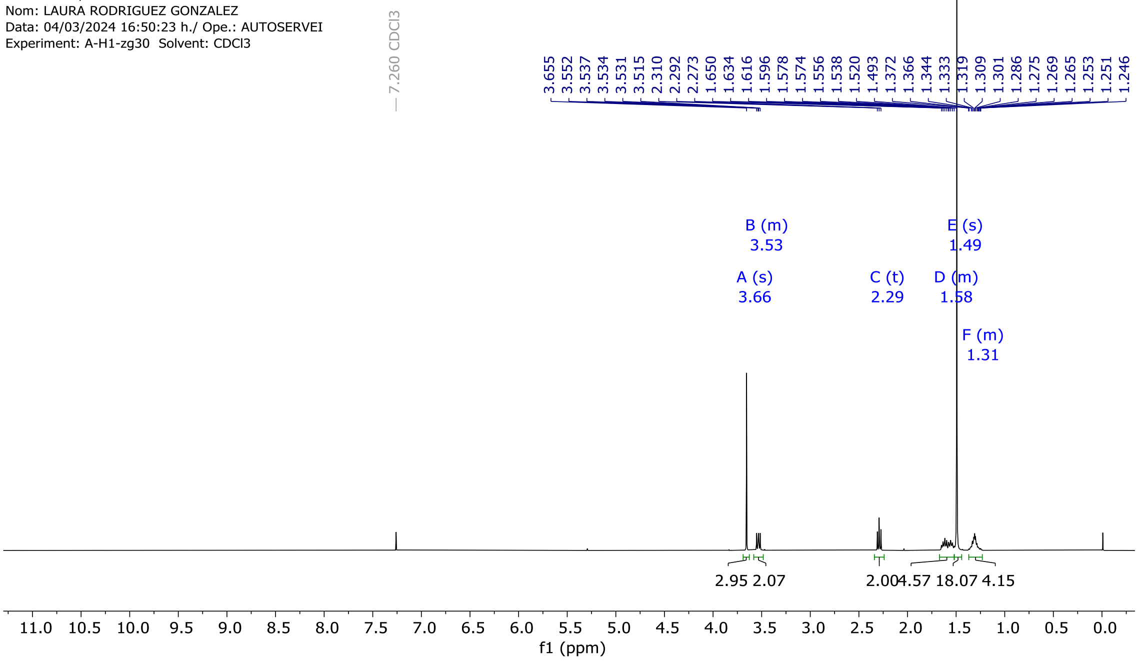

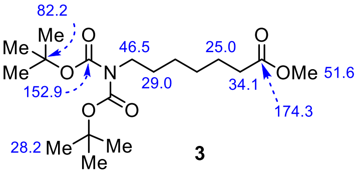

3

24030080_B400FA_05032024_LRG554PREP.2.fid 13C{1H} 101 MHz
Equip: B400F / N.Inv: 1037597
N.Reg: 24030080
Usuari: san / Mostra: LRG554PREP
Nom: LAURA RODRIGUEZ GONZALEZ
Data: 05/03/2024 07:30:57 h./ Ope.: AUTOSERVEI
Experiment: A-C13-zgpg30 Solvent: CDCl3

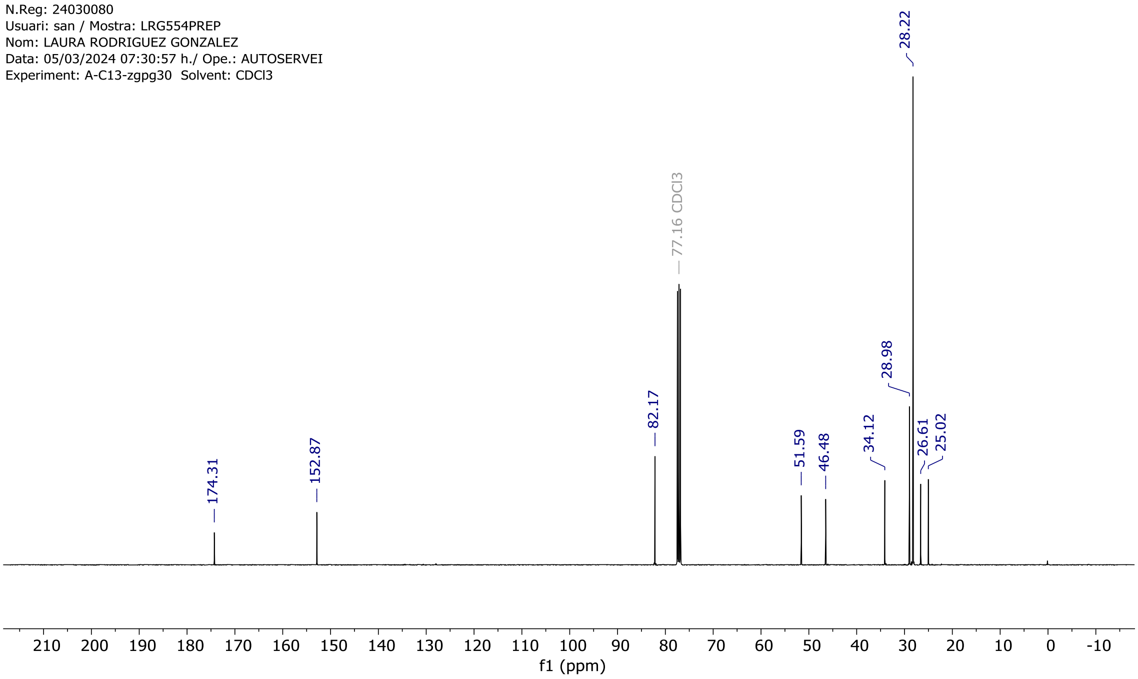

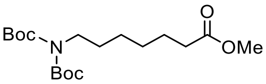

3

2D-COSY

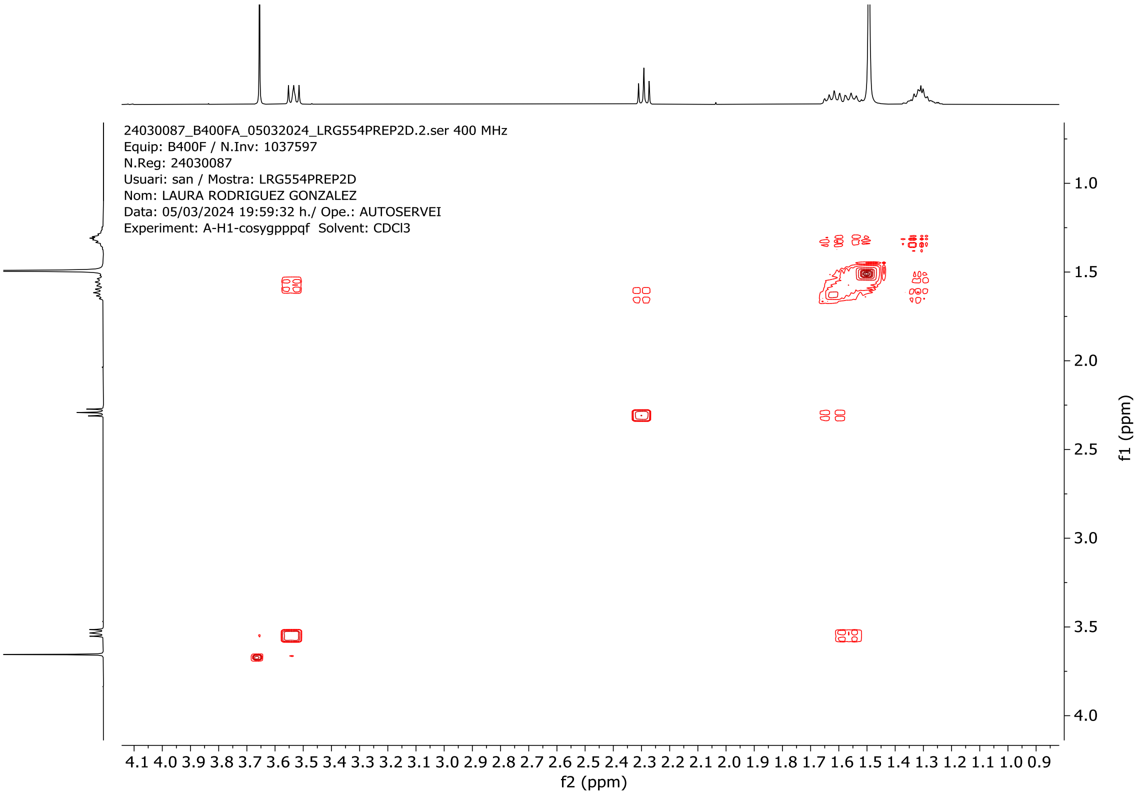

2D-HSQC

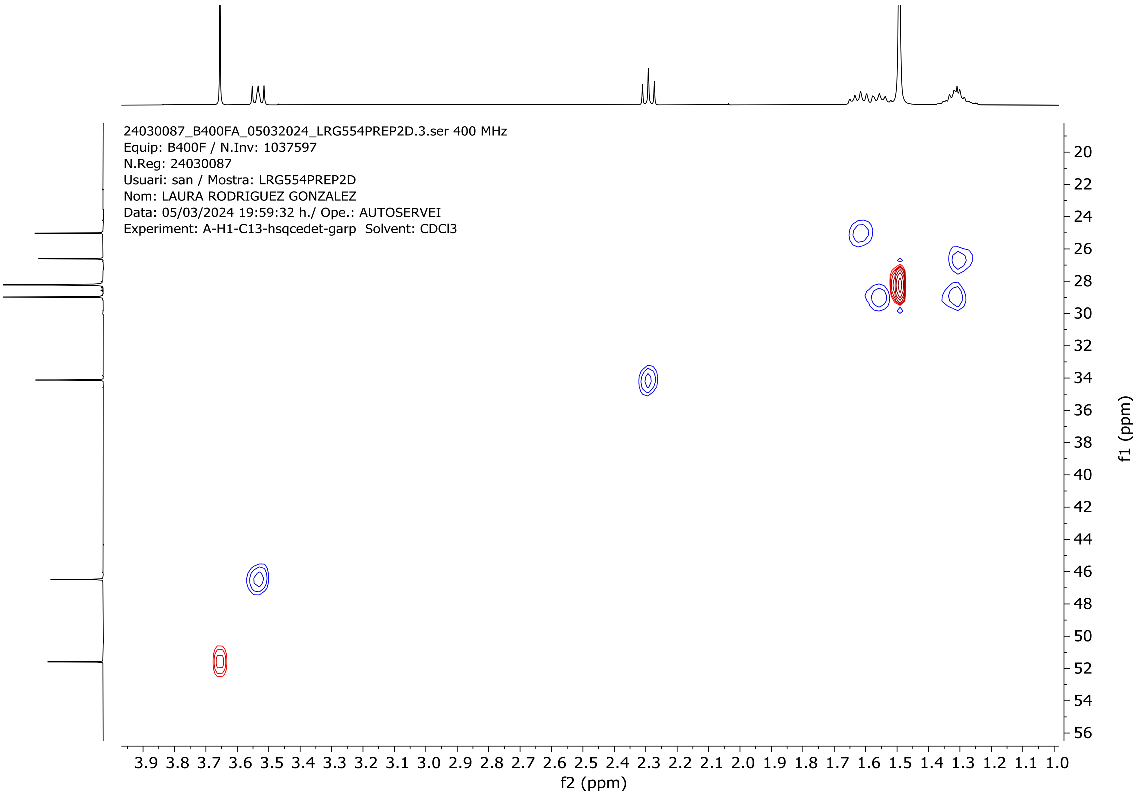

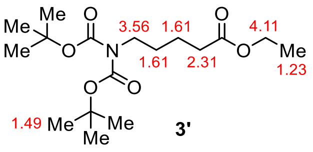

24020563_B400FA_20022024_ASV009COLT19T21CH.1.fid 1H 400 MHz
Equip: B400F / N.Inv: 1037597
N.Reg: 24020563
Usuari: san / Mostra: ASV009COLT19T21CH
Nom: LAURA RODRIGUEZ GONZALEZ
Data: 19/02/2024 16:25:56 h./ Ope.: AUTOSERVEI
Experiment: A-H1-zg30 Solvent: CDCl3

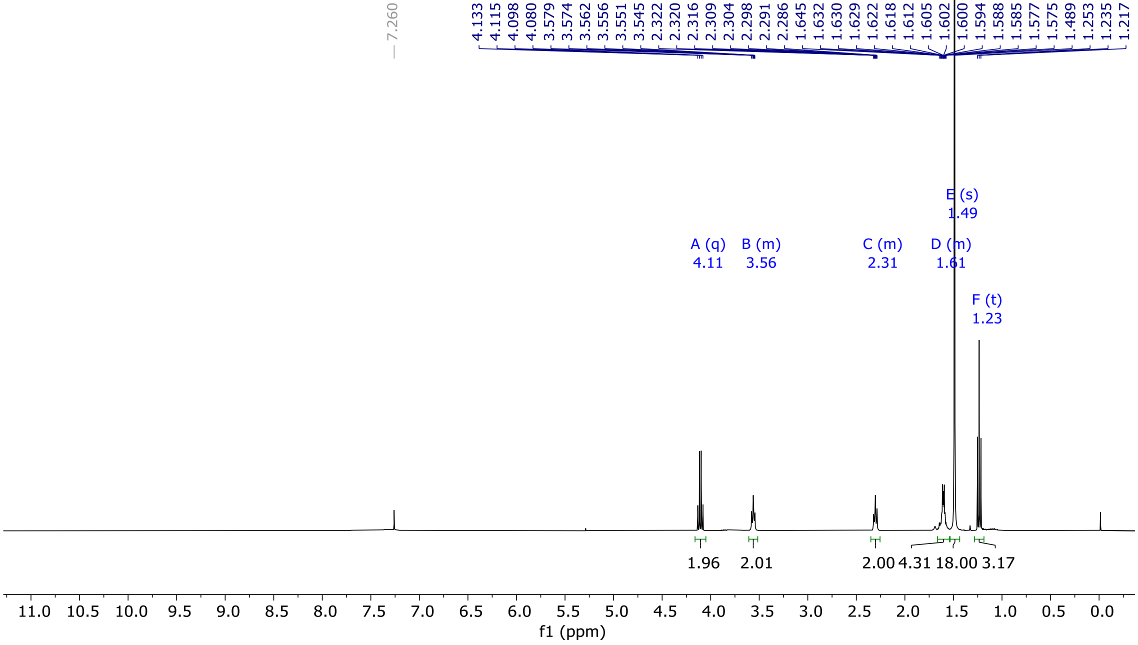

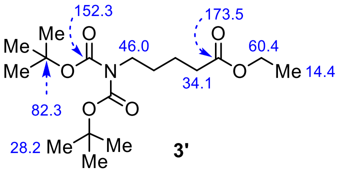

24020563_B400FA_20022024_ASV009COLT19T21CH.2.fid 13C{1H} 101 MHz
Equip: B400F / N.Inv: 1037597
N.Reg: 24020563
Usuari: san / Mostra: ASV009COLT19T21CH
Nom: LAURA RODRIGUEZ GONZALEZ
Data: 20/02/2024 01:23:54 h./ Ope.: AUTOSERVEI
Experiment: A-C13-zgpg30 Solvent: CDCl3

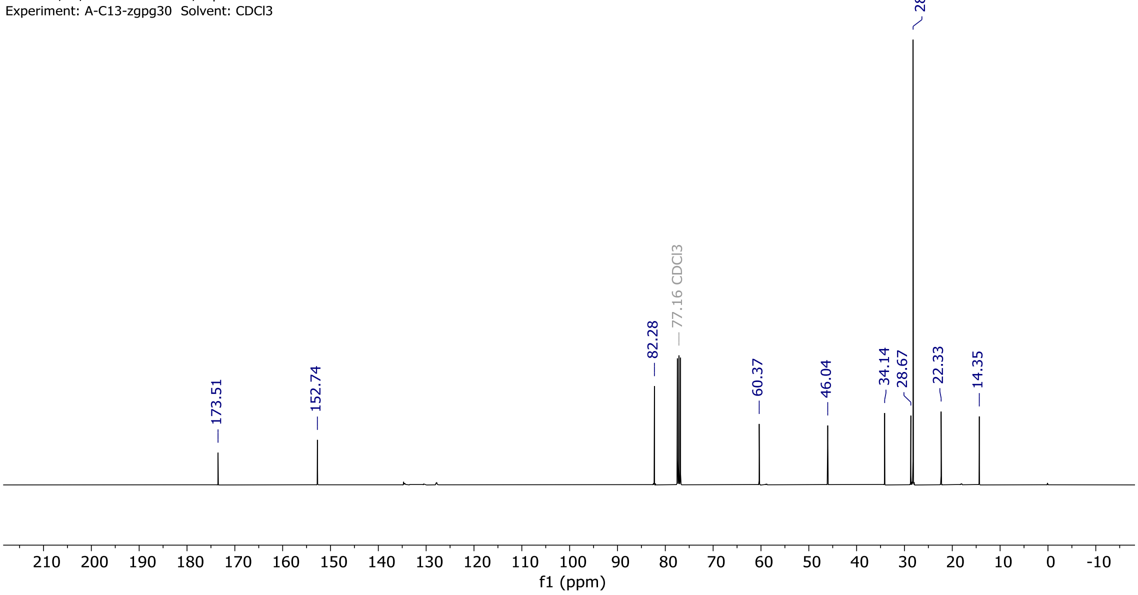

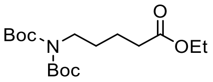

3'

2D-COSY

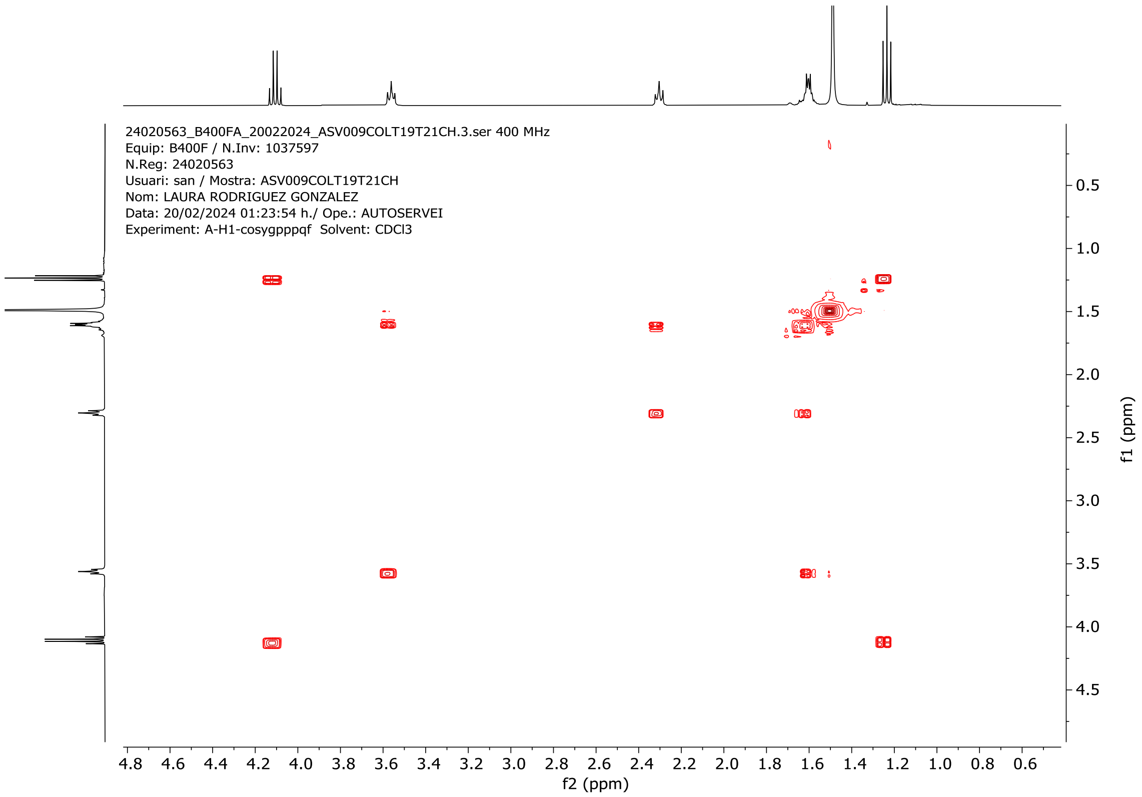

2D-HSQC

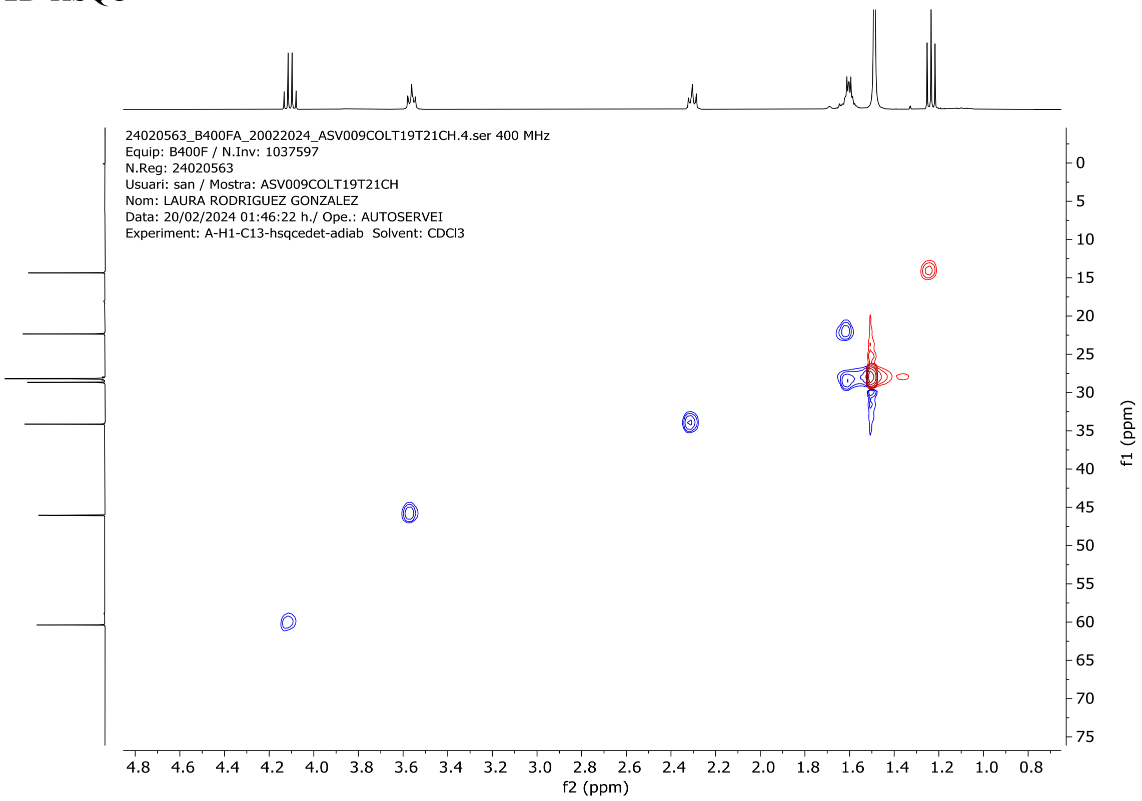

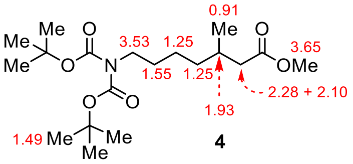

4

24030370_B400FA_13032024_LRG566COLT24T25.1.fid 1H 400 MHz
Equip: B400F / N.Inv: 1037597
N.Reg: 24030370
Usuari: san / Mostra: LRG566COLT24T25
Nom: LAURA RODRIGUEZ GONZALEZ
Data: 13/03/2024 11:37:53 h./ Ope.: AUTOSERVEI
Experiment: A-H1-zg30 Solvent: CDCl3

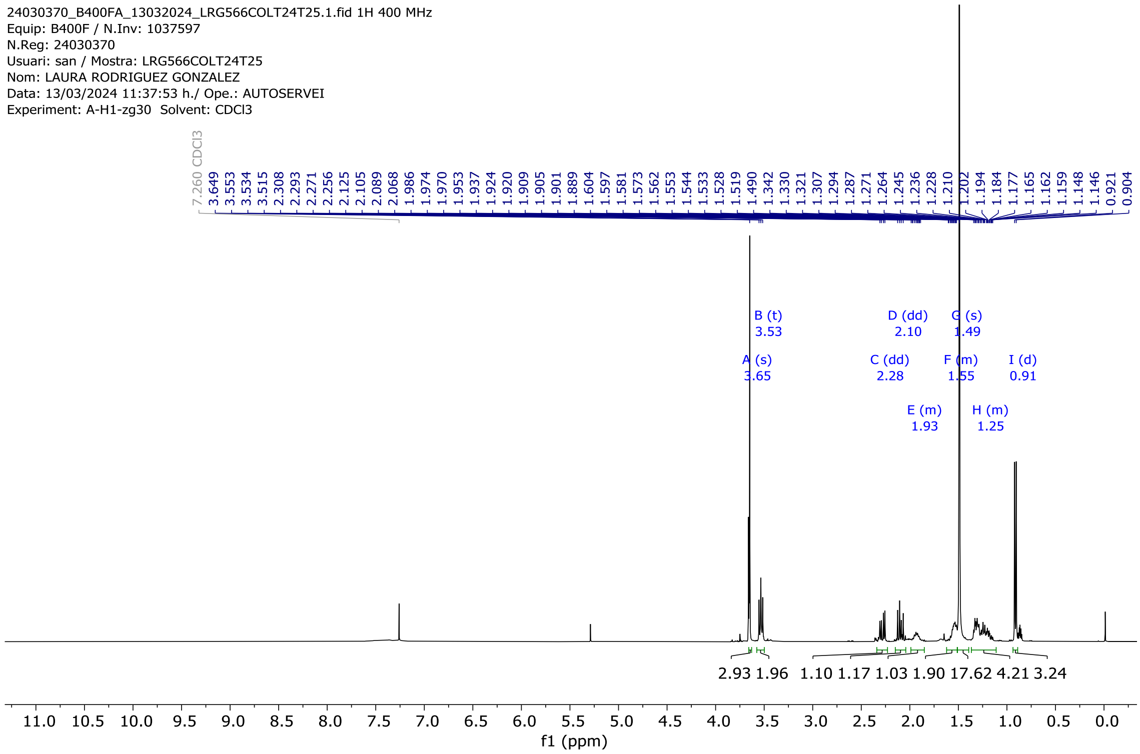

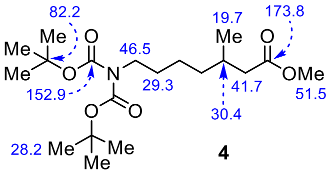

4

24030370_B400FA_14032024_LRG566COLT24T25.2.fid 13C{1H} 101 MHz
Equip: B400F / N.Inv: 1037597
N.Reg: 24030370
Usuari: san / Mostra: LRG566COLT24T25
Nom: LAURA RODRIGUEZ GONZALEZ
Data: 14/03/2024 03:19:08 h./ Ope.: AUTOSERVEI
Experiment: A-C13-zgpg30 Solvent: CDCl3

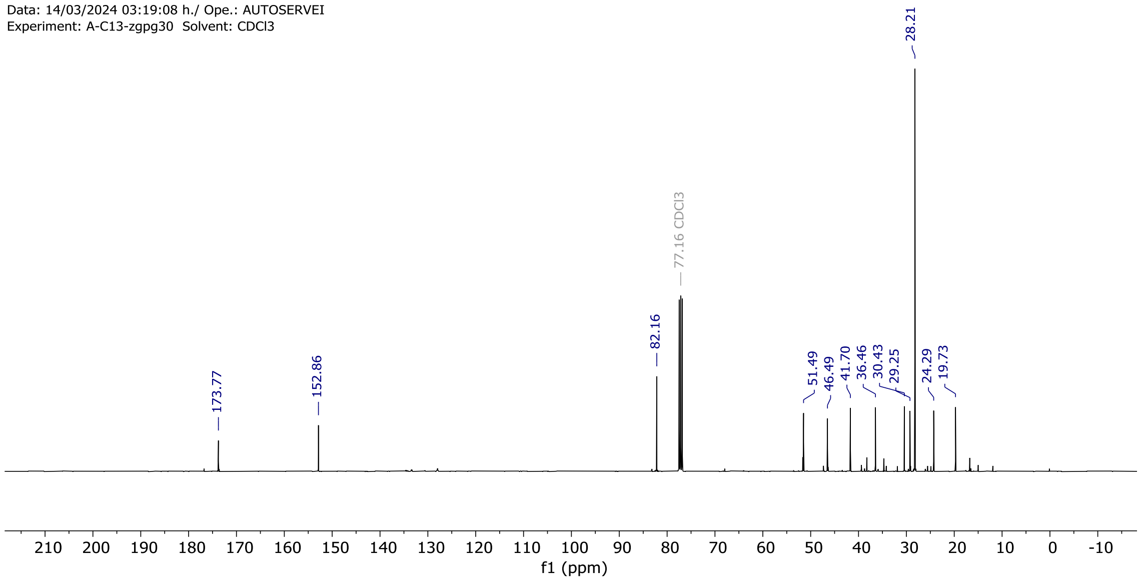

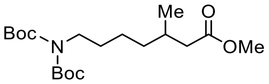

4

2D-COSY

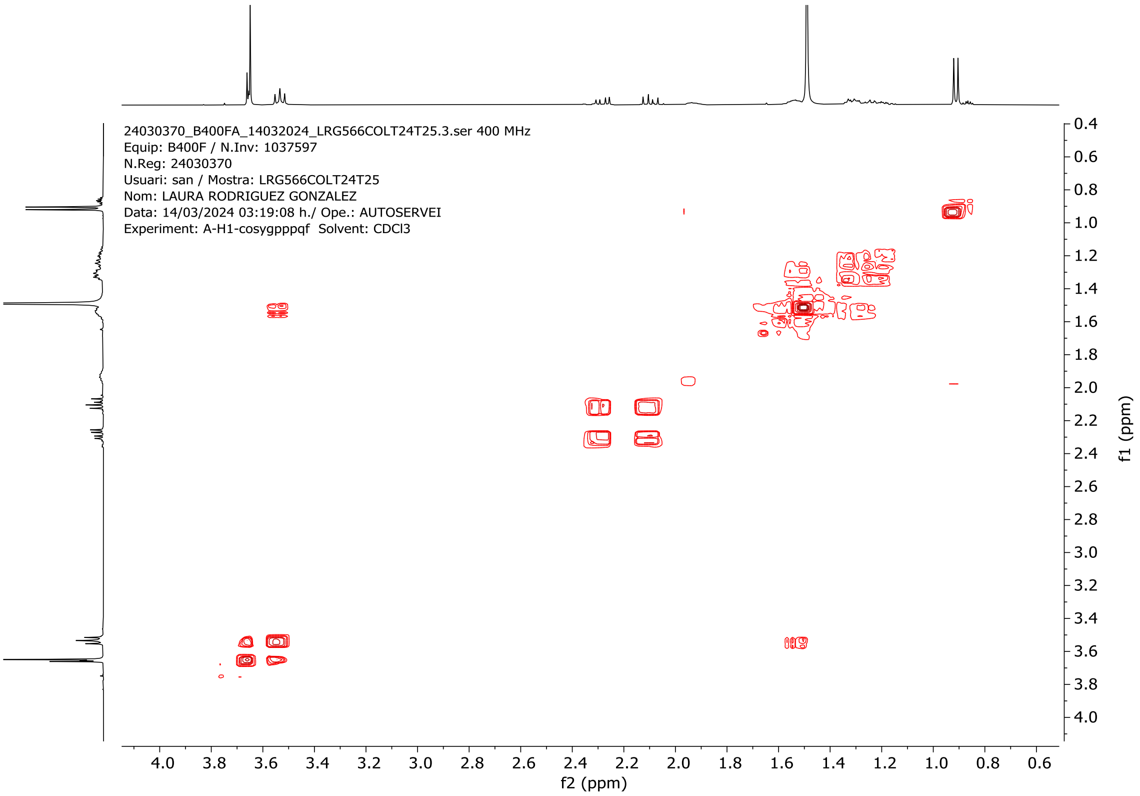

2D-HSQC

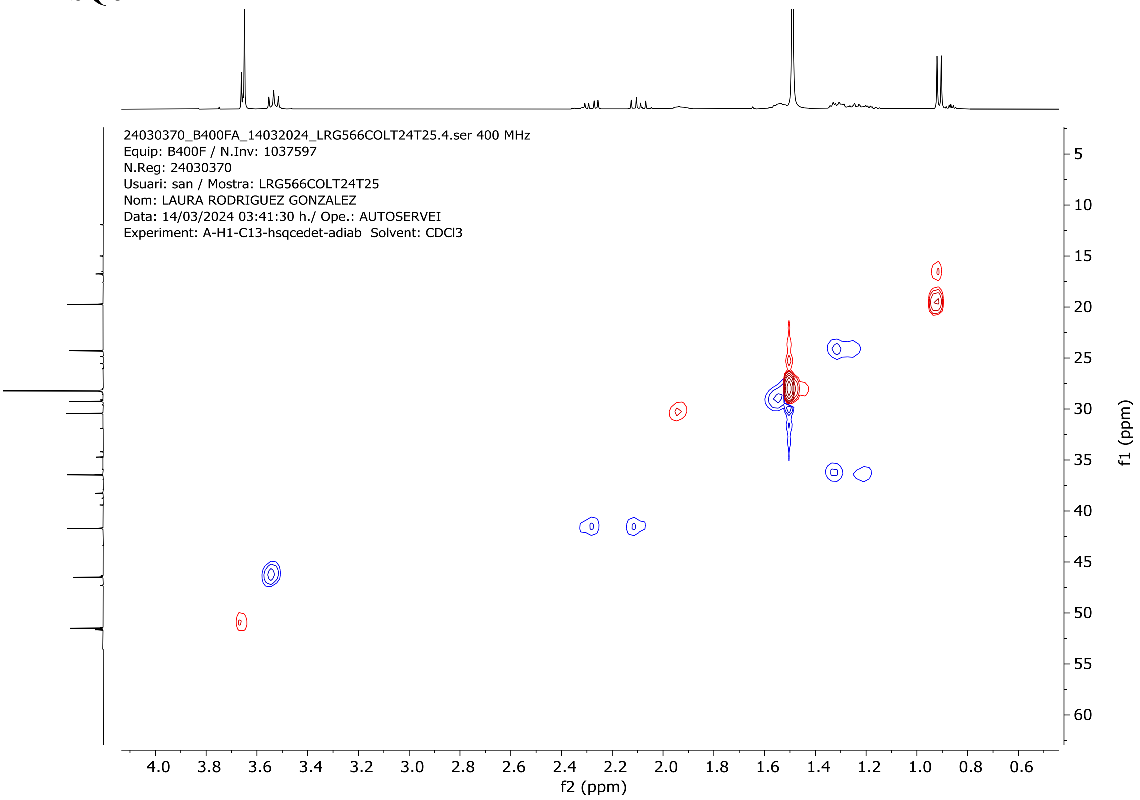

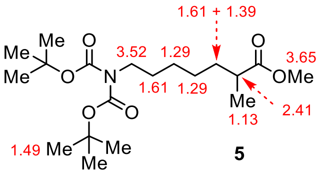

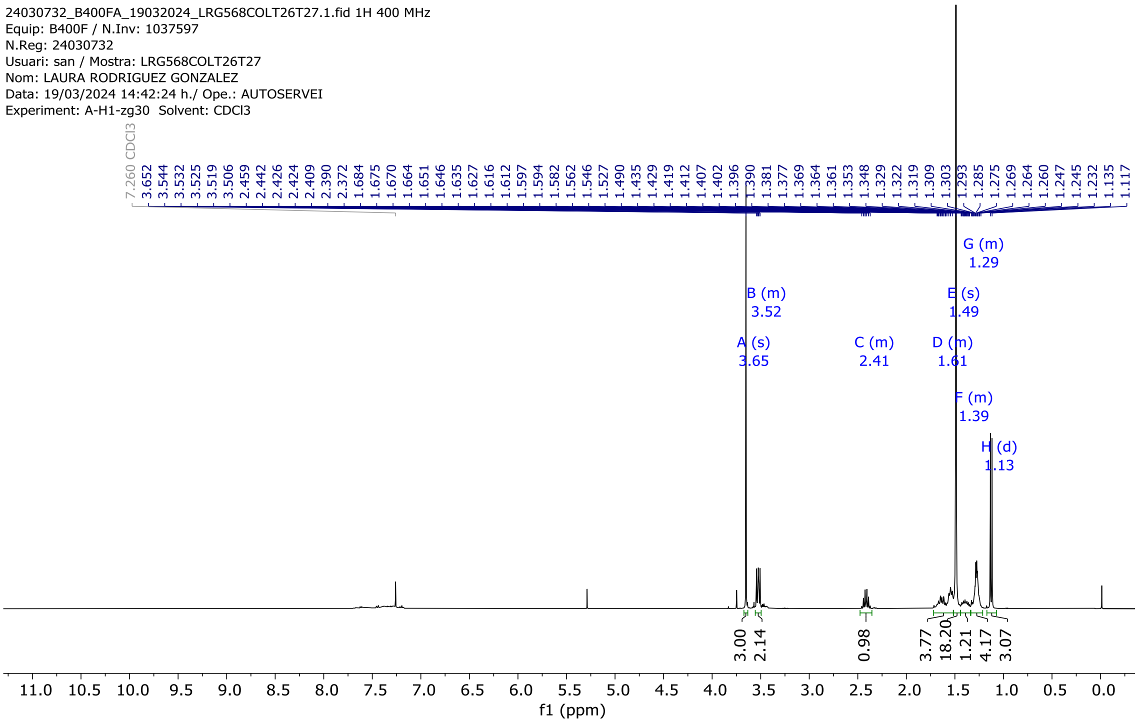

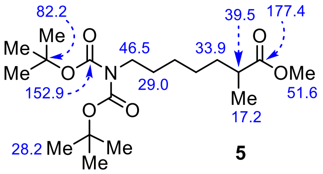

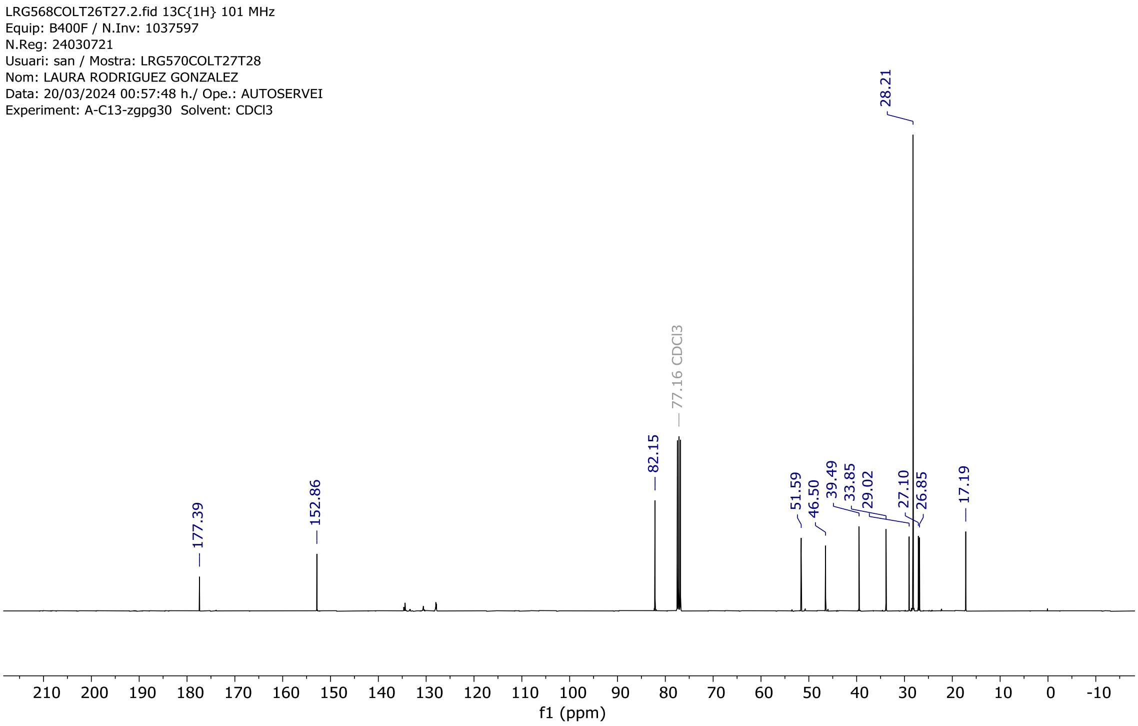

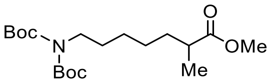

5

2D-COSY

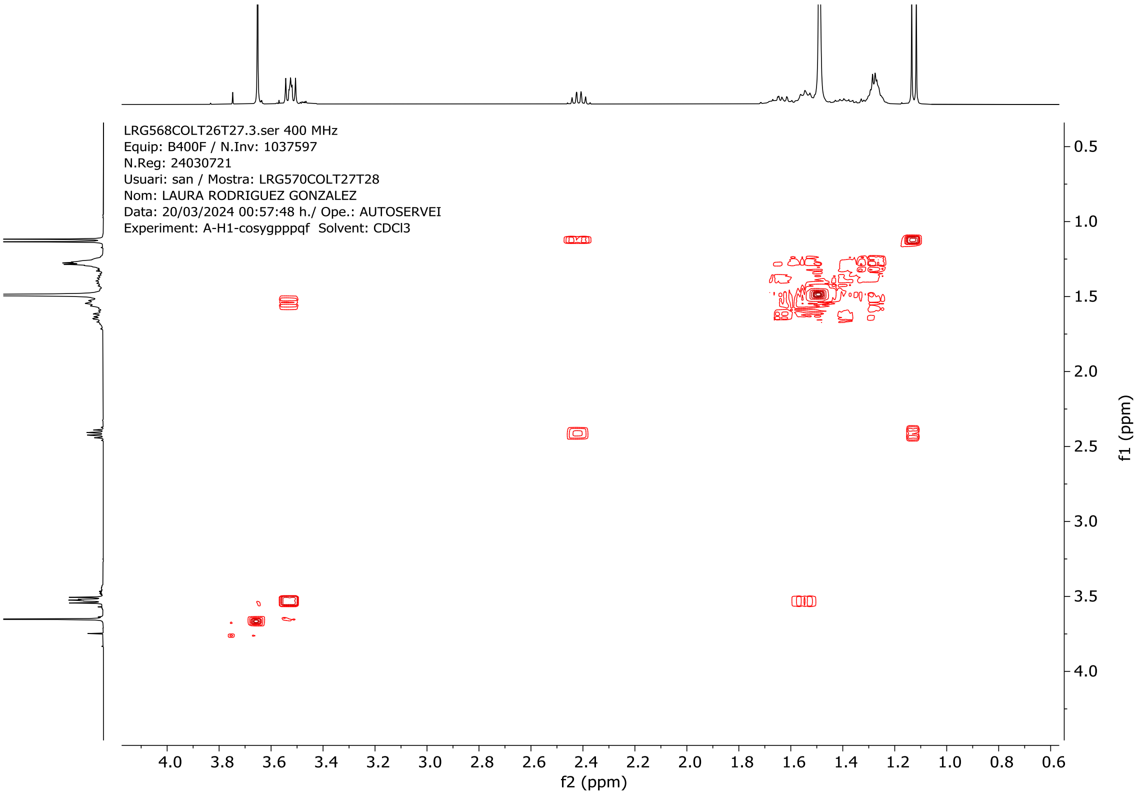

2D-HSQC

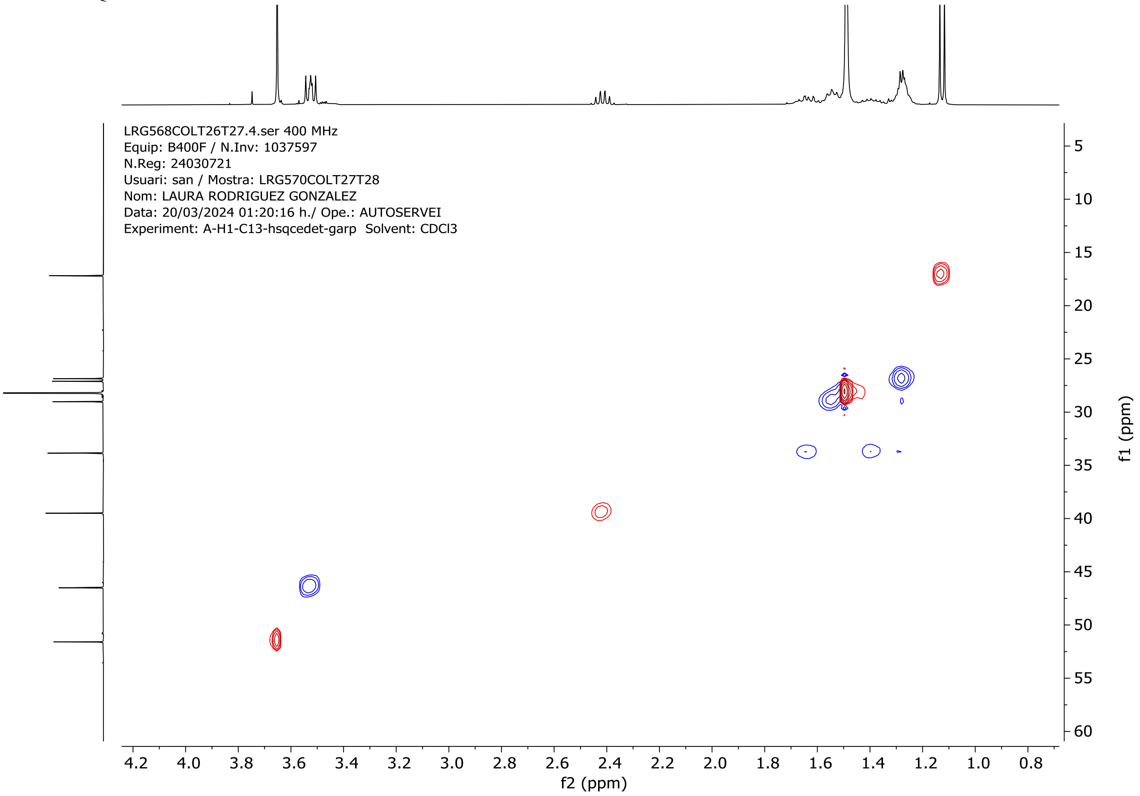

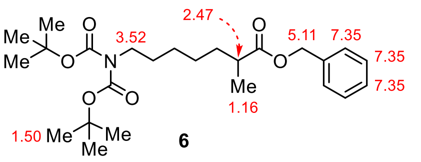

6

24030199_B400FA_07032024_ASV020-18-19.1.fid 1H 400 MHz
Equip: B400F / N.Inv: 1037597
N.Reg: 24030199
Usuari: san / Mostra: ASV020-18-19
Nom: LAURA RODRIGUEZ GONZALEZ
Data: 07/03/2024 16:15:15 h./ Ope.: AUTOSERVEI
Experiment: A-H1-zg30 Solvent: CDCl3

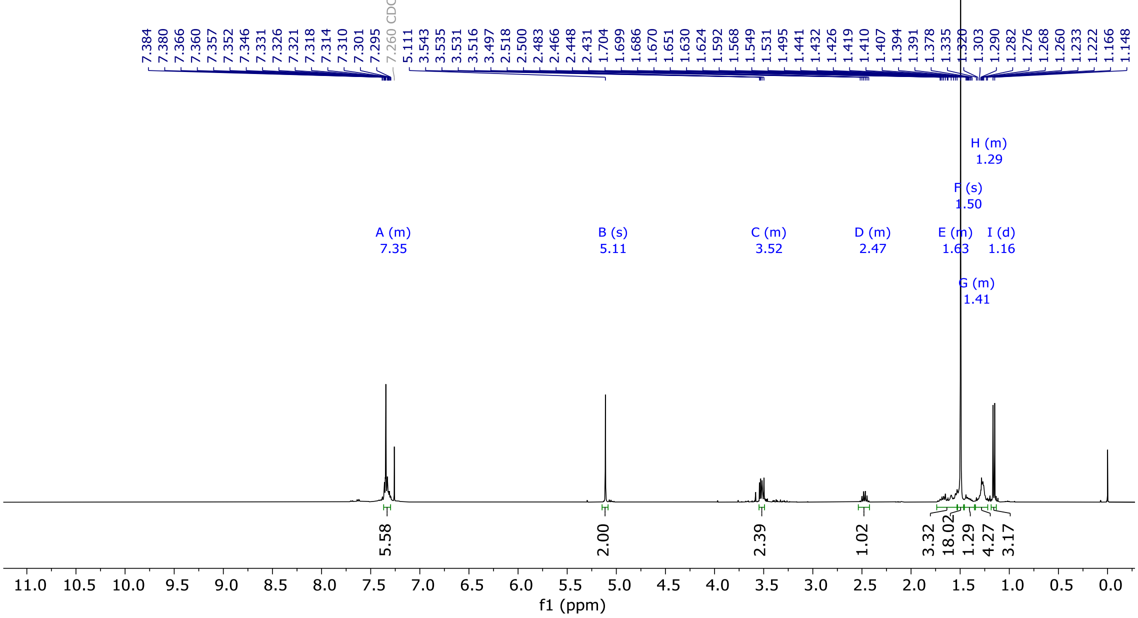

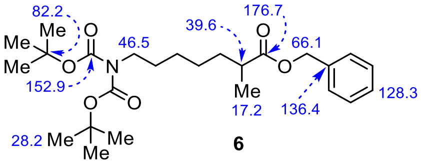

6

24030186_B400FA_08032024_ASV020colt14t16.2.fid 13C{1H} 101 MHz
Equip: B400F / N.Inv: 1037597
N.Reg: 24030186
Usuari: san / Mostra: ASV020colt14t16
Nom: LAURA RODRIGUEZ GONZALEZ
Data: 08/03/2024 04:02:59 h./ Ope.: AUTOSERVEI
Experiment: A-C13-zgpg30 Solvent: CDCl3

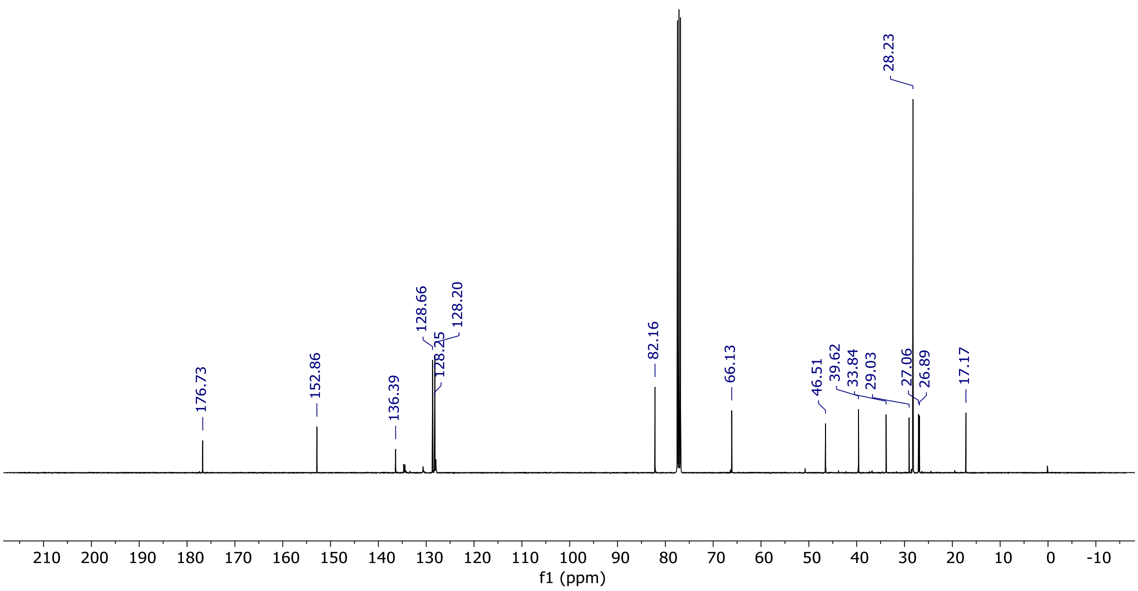

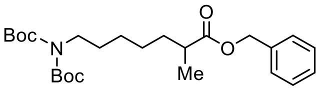

6

2D-COSY

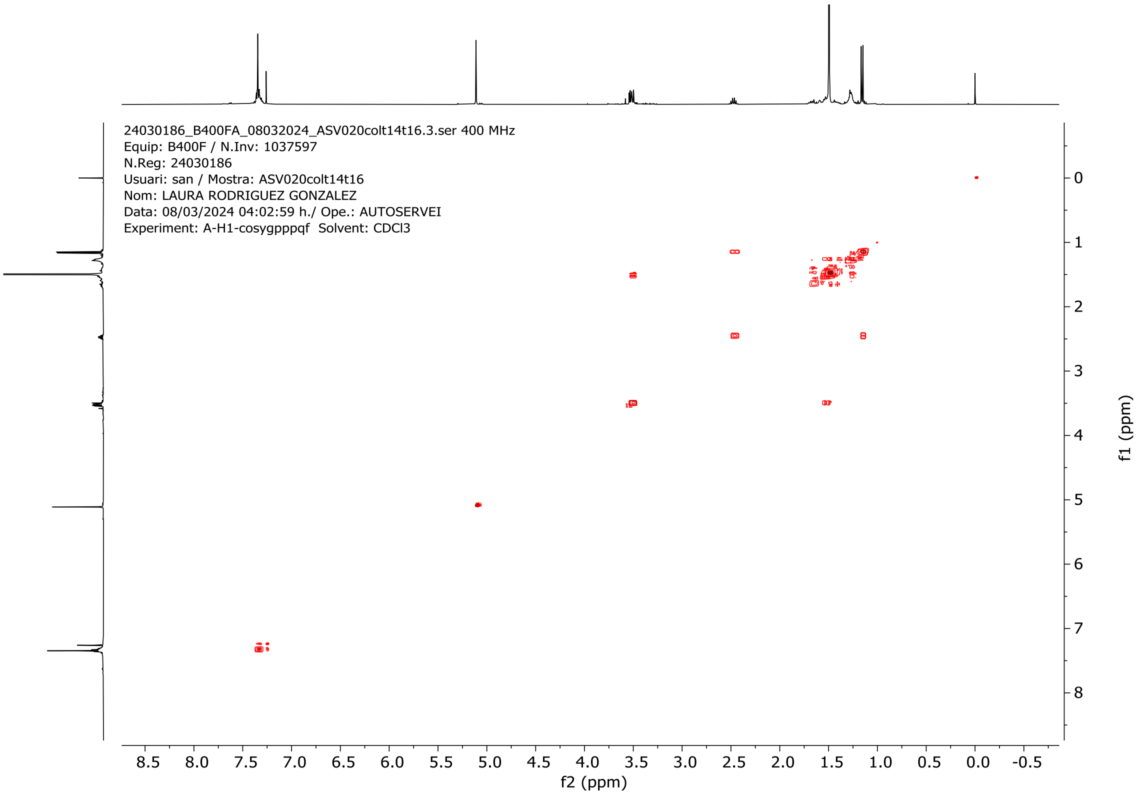

2D-HSQC

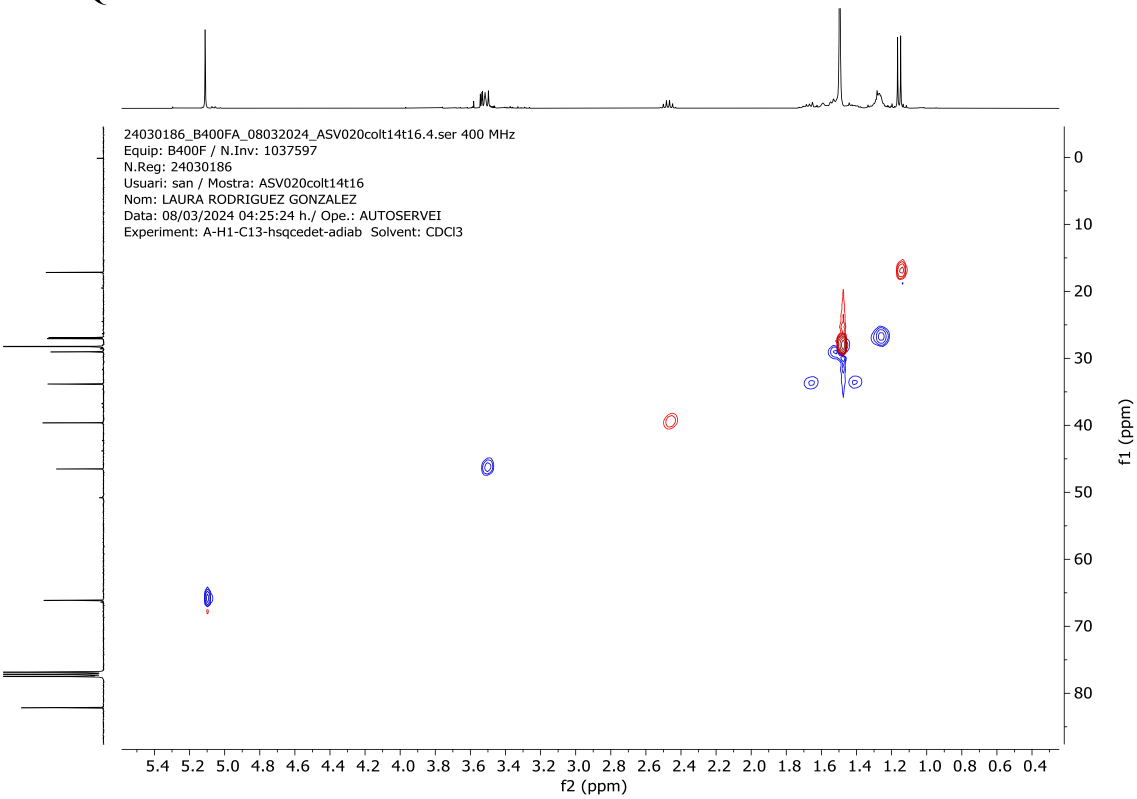

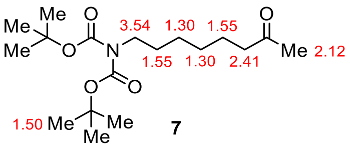

24030306_B400FA_12032024_ASV023-27-28.1.fid 1H 400 MHz
Equip: B400F / N.Inv: 1037597
N.Reg: 24030306
Usuari: san / Mostra: ASV023-27-28
Nom: AINA SERRA VERT
Data: 12/03/2024 12:37:41 h./ Ope.: AUTOSERVEI
Experiment: A-H1-zg30 Solvent: CDCl3

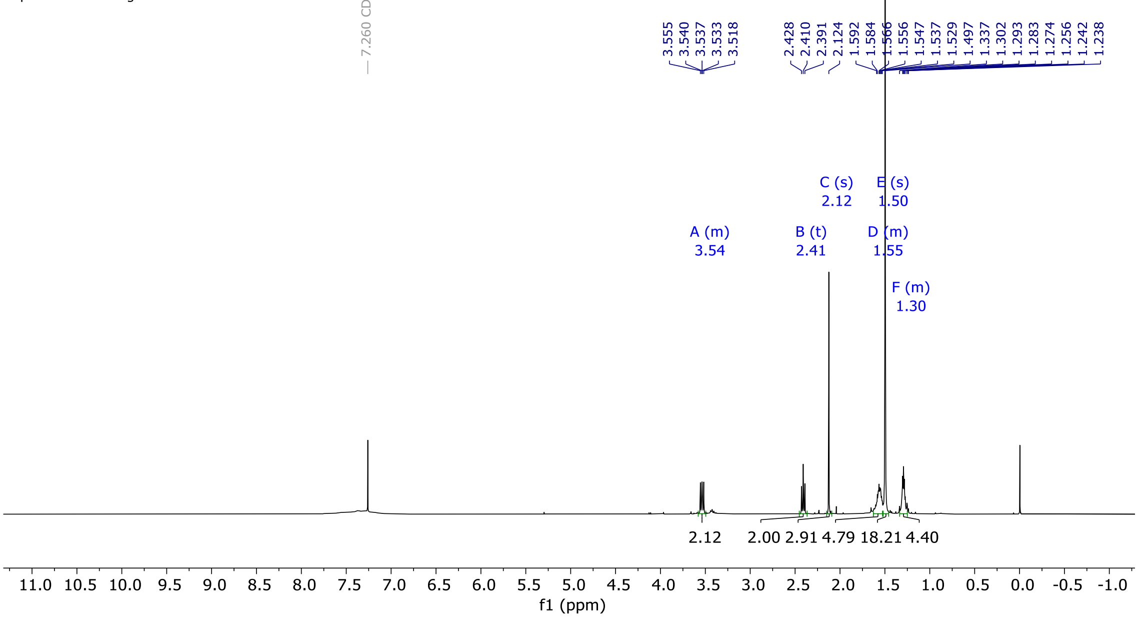

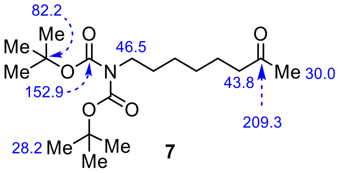

ASV023COLT28T29.2.fid 13C{1H} 101 MHz
Equip: B400F / N.Inv: 1037597
N.Reg: 24030301
Usuari: san / Mostra: LRG563COLT15T19
Nom: LAURA RODRIGUEZ GONZALEZ
Data: 13/03/2024 03:49:33 h./ Ope.: AUTOSERVEI
Experiment: A-C13-zgpg30 Solvent: CDCl3

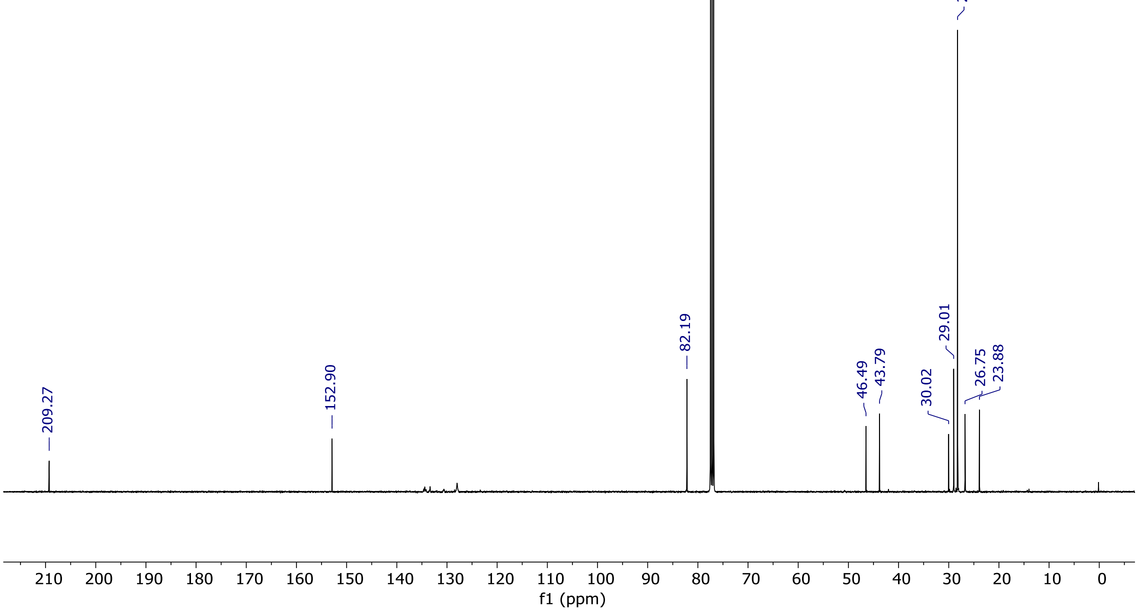

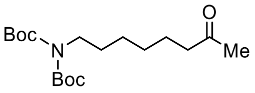

7

2D-COSY

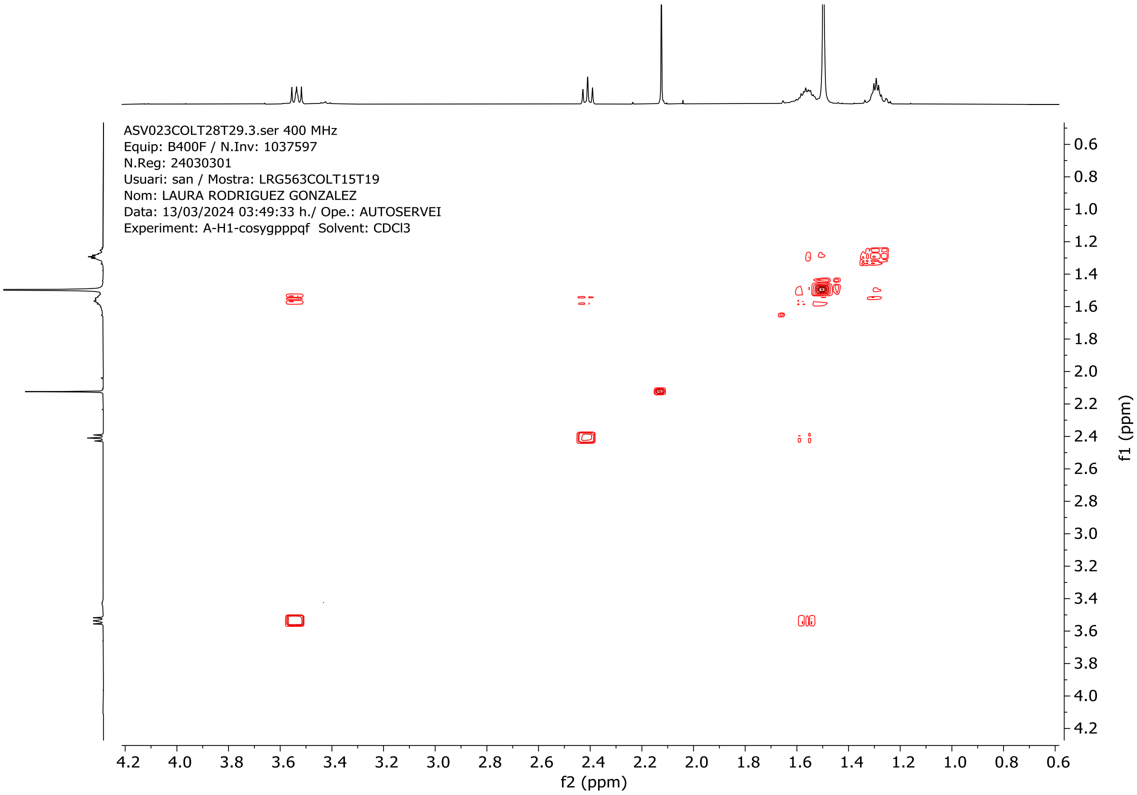

2D-HSQC

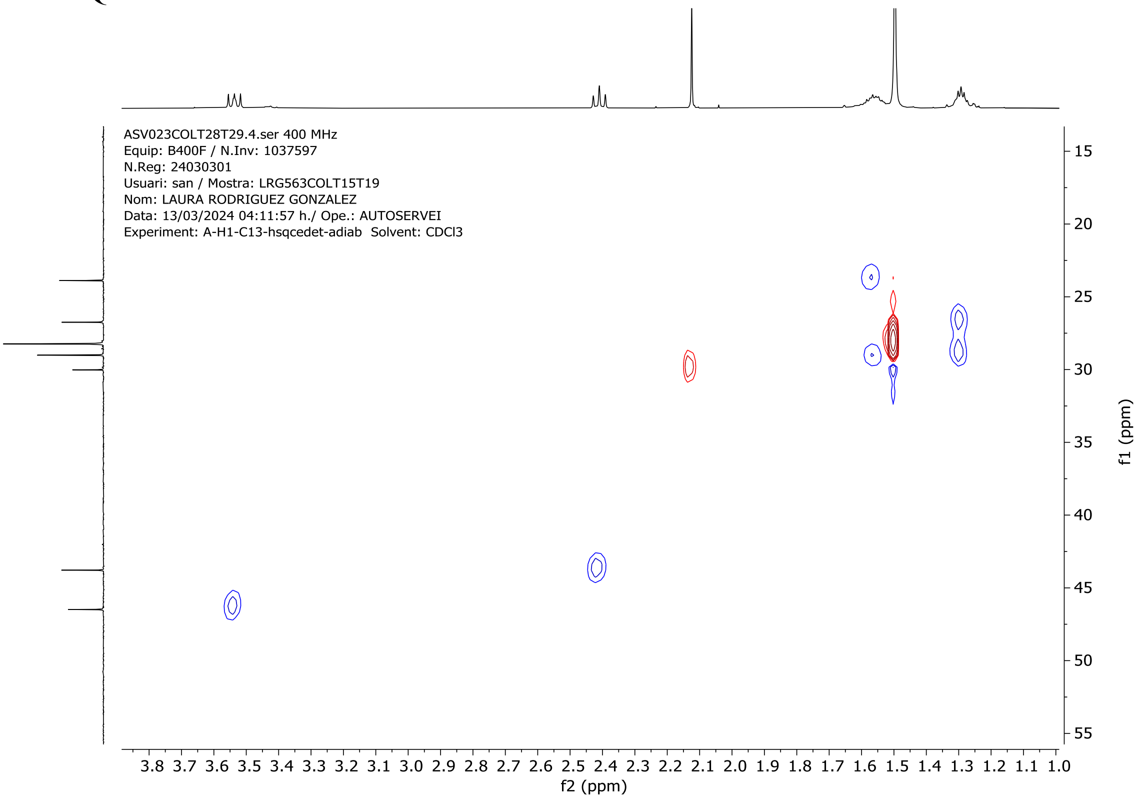

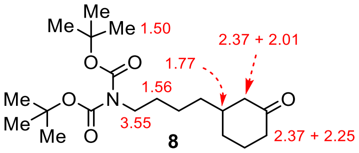

24030532_B400FA_15032024_ASV026PREPCH.1.fid 1H 400 MHz
Equip: B400F / N.Inv: 1037597
N.Reg: 24030532
Usuari: san / Mostra: ASV026PREPCH
Nom: LAURA RODRIGUEZ GONZALEZ
Data: 15/03/2024 09:59:31 h./ Ope.: AUTOSERVEI
Experiment: A-H1-zg30 Solvent: CDCl3

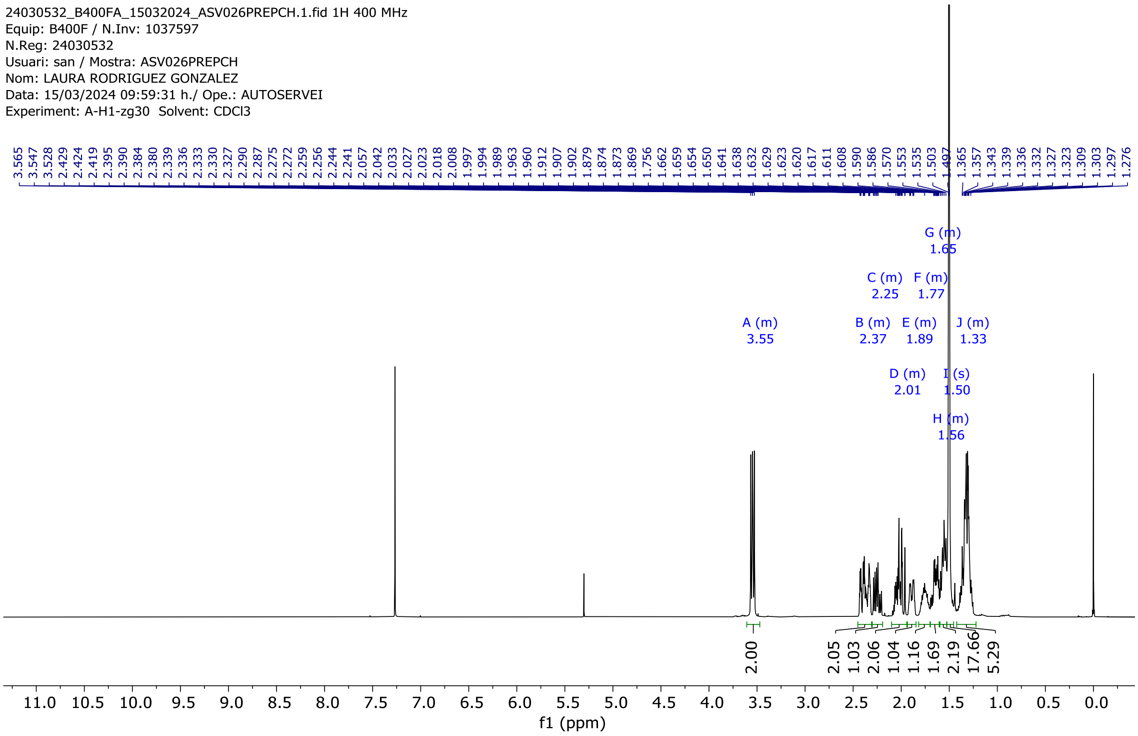

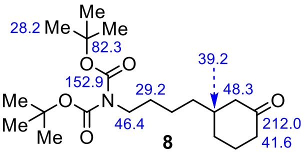

24030539_B400FA_16032024_ASV026FULL.2.fid 13C{1H} 101 MHz
Equip: B400F / N.Inv: 1037597
N.Reg: 24030539
Usuari: san / Mostra: ASV026FULL
Nom: LAURA RODRIGUEZ GONZALEZ
Data: 15/03/2024 22:28:50 h./ Ope.: AUTOSERVEI
Experiment: A-C13-zgpg30 Solvent: CDCl3

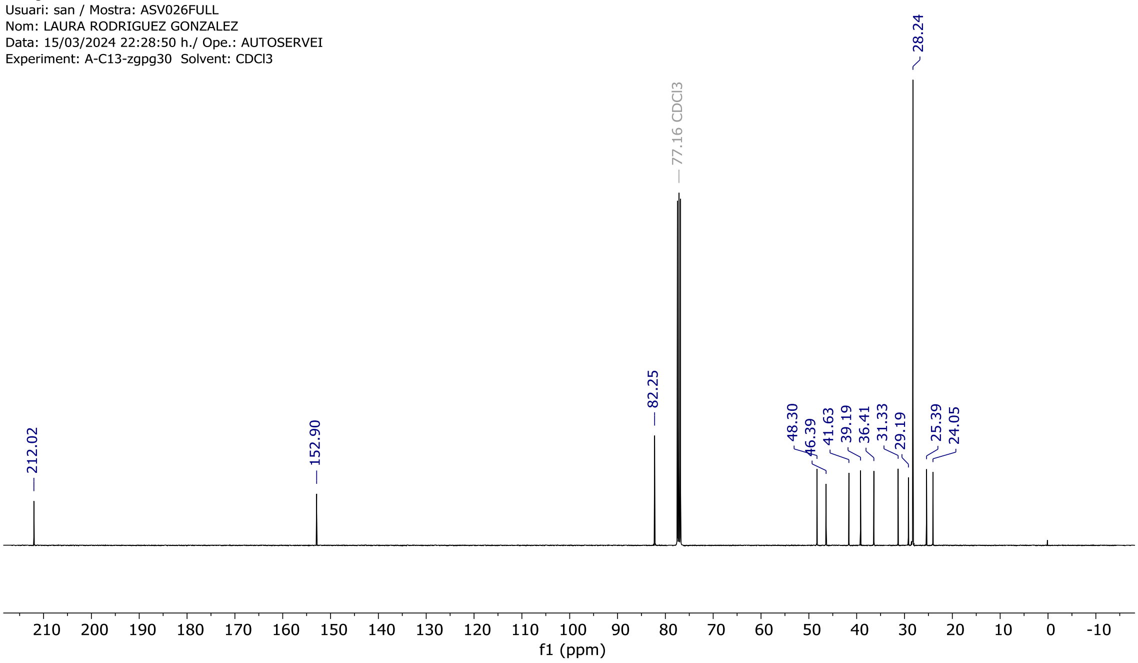

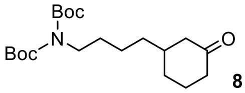

8

2D-COSY

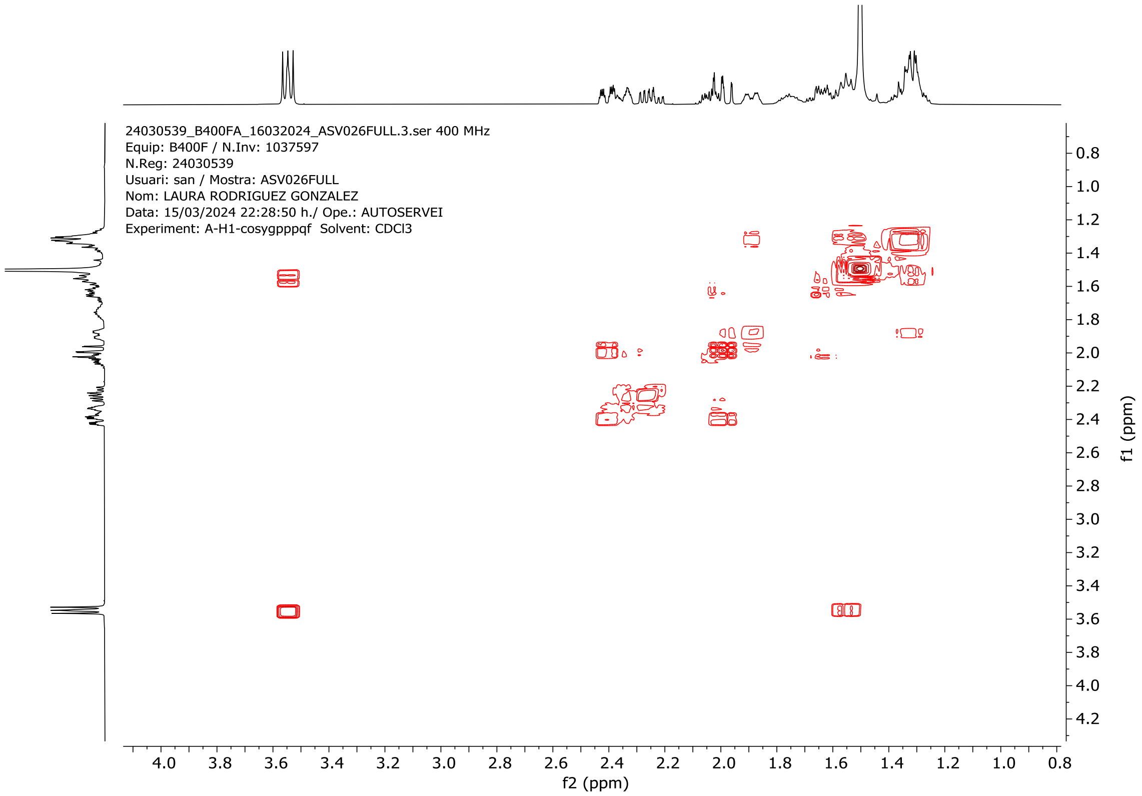

2D-HSQC

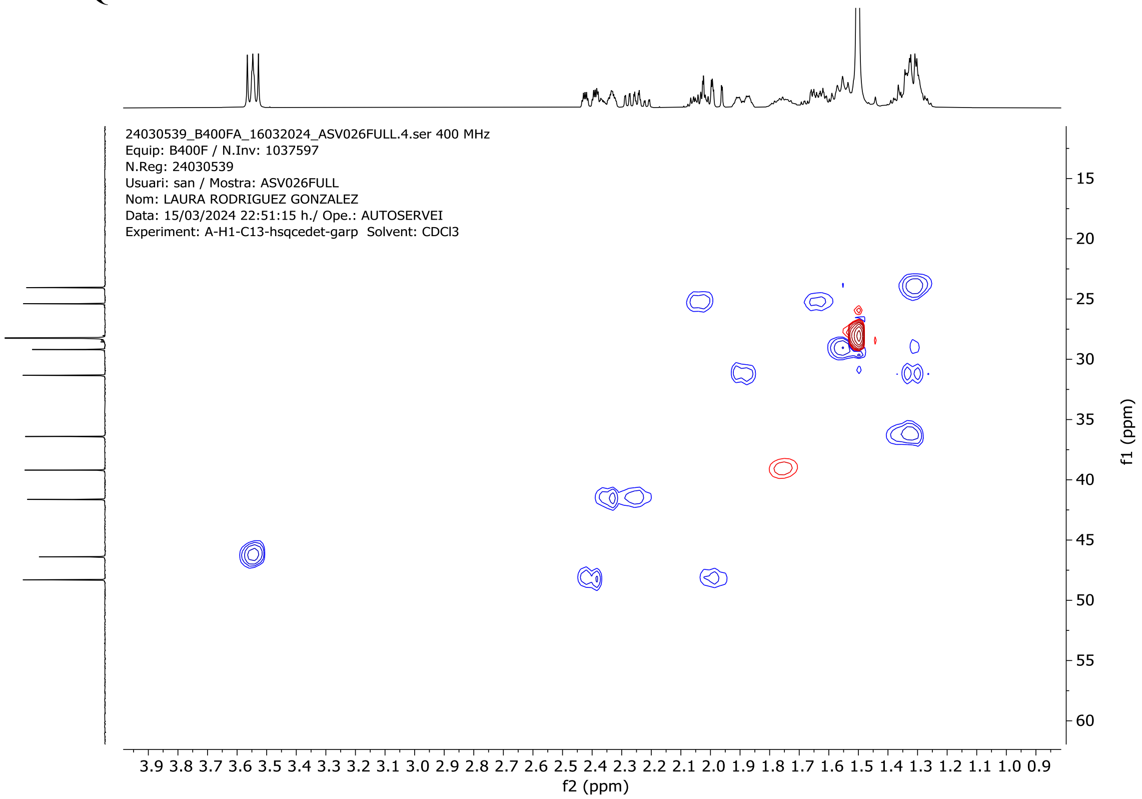

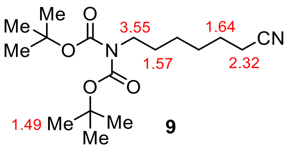

24030228_B400FA_08032024_ASV021-28-29.1.fid 1H 400 MHz
Equip: B400F / N.Inv: 1037597
N.Reg: 24030228
Usuari: san / Mostra: ASV021-28-29
Nom: LAURA RODRIGUEZ GONZALEZ
Data: 08/03/2024 11:48:18 h./ Ope.: AUTOSERVEI
Experiment: A-H1-zg30 Solvent: CDCl3

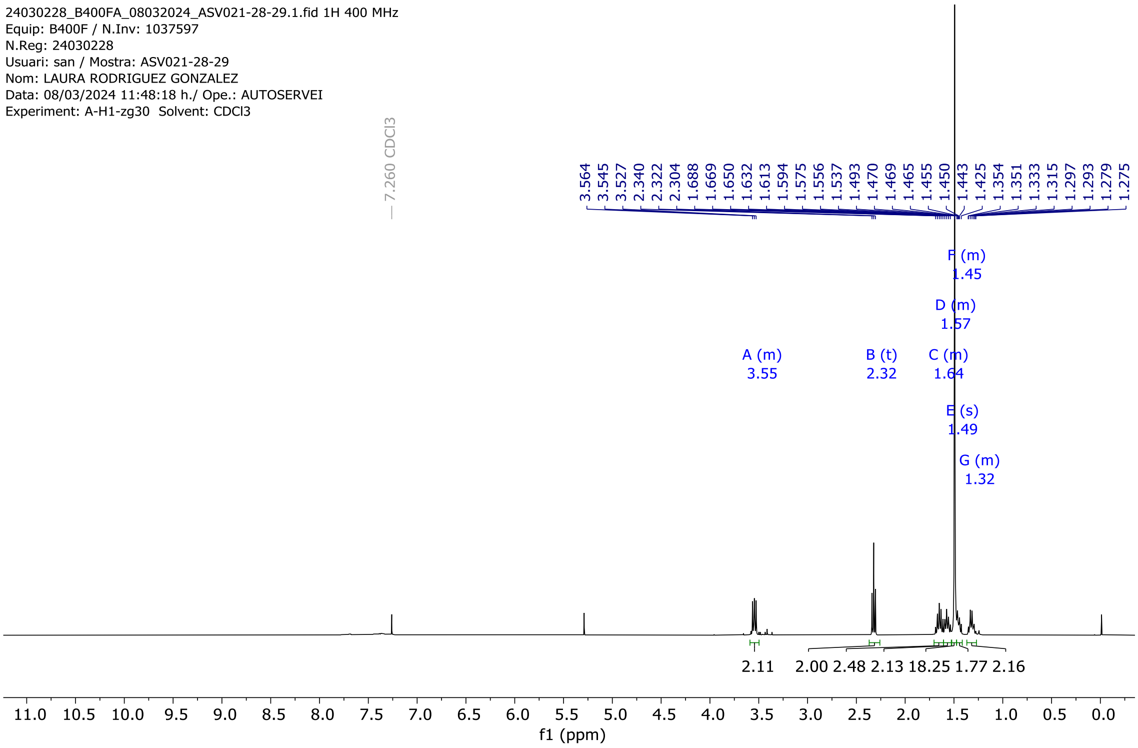

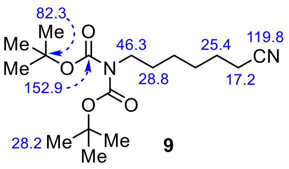

24030228_B400FA_08032024_ASV021-28-29.2.fid 13C{1H} 101 MHz
Equip: B400F / N.Inv: 1037597
N.Reg: 24030228
Usuari: san / Mostra: ASV021-28-29
Nom: LAURA RODRIGUEZ GONZALEZ
Data: 08/03/2024 21:49:00 h./ Ope.: AUTOSERVEI
Experiment: A-C13-zgpg30 Solvent: CDCl3

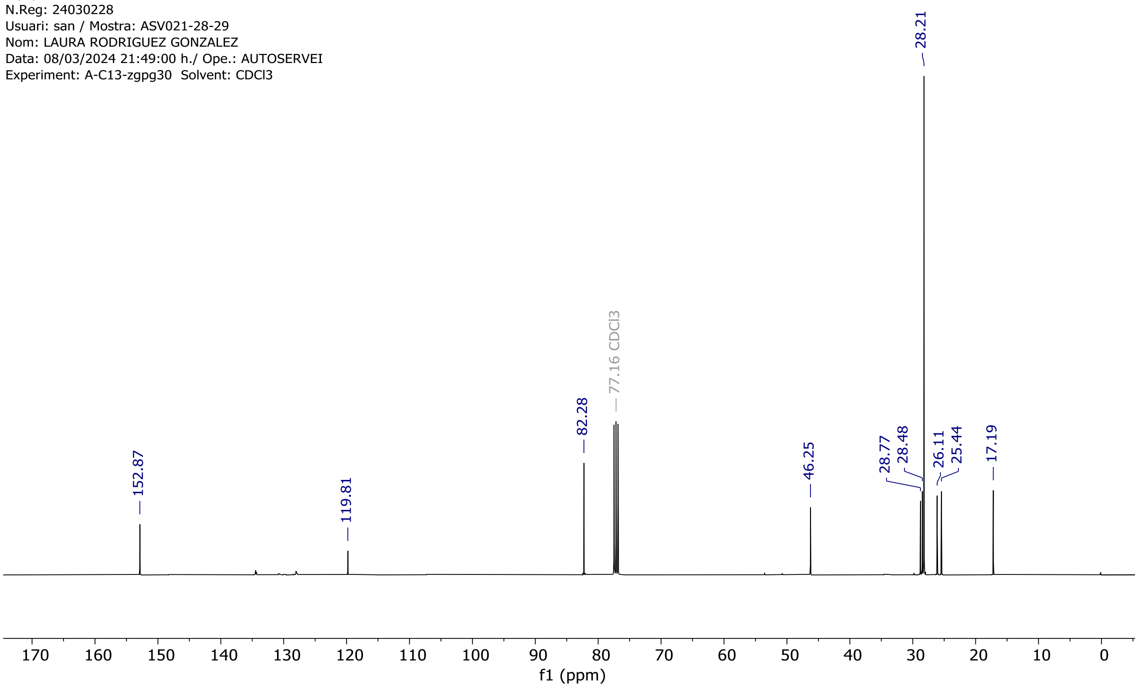

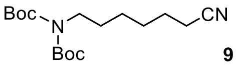

9

2D-COSY

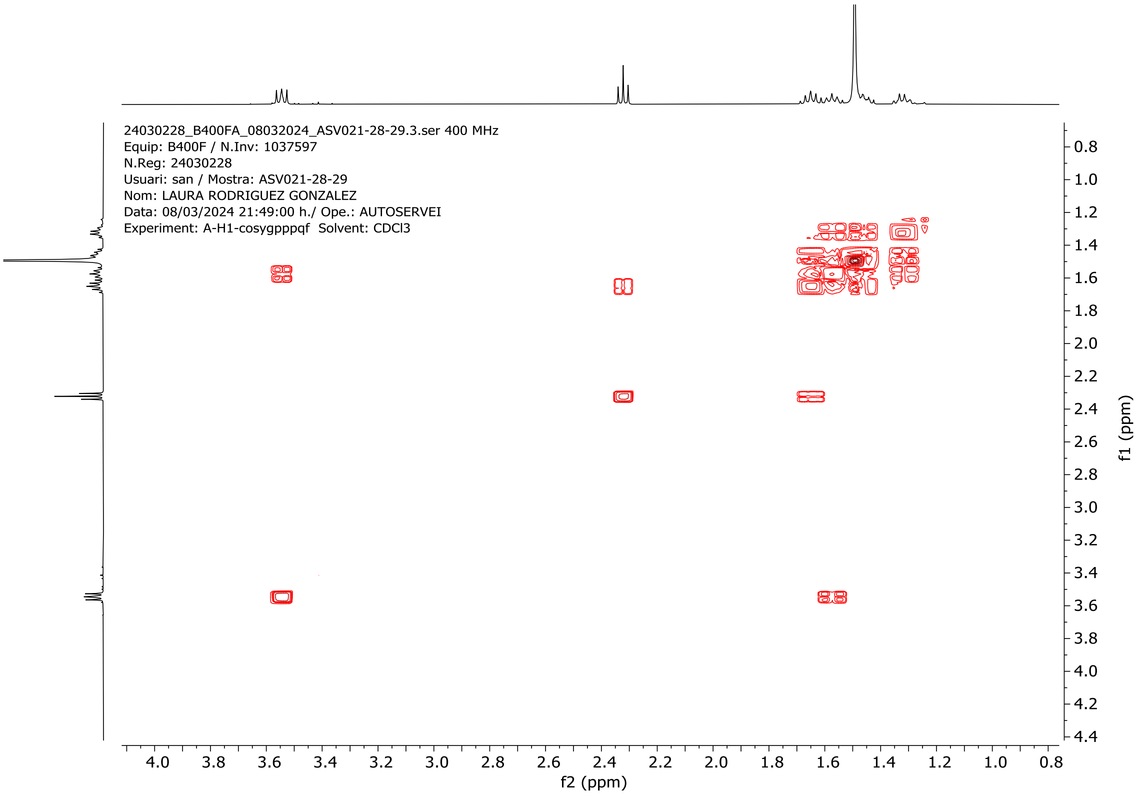

2D-HSQC

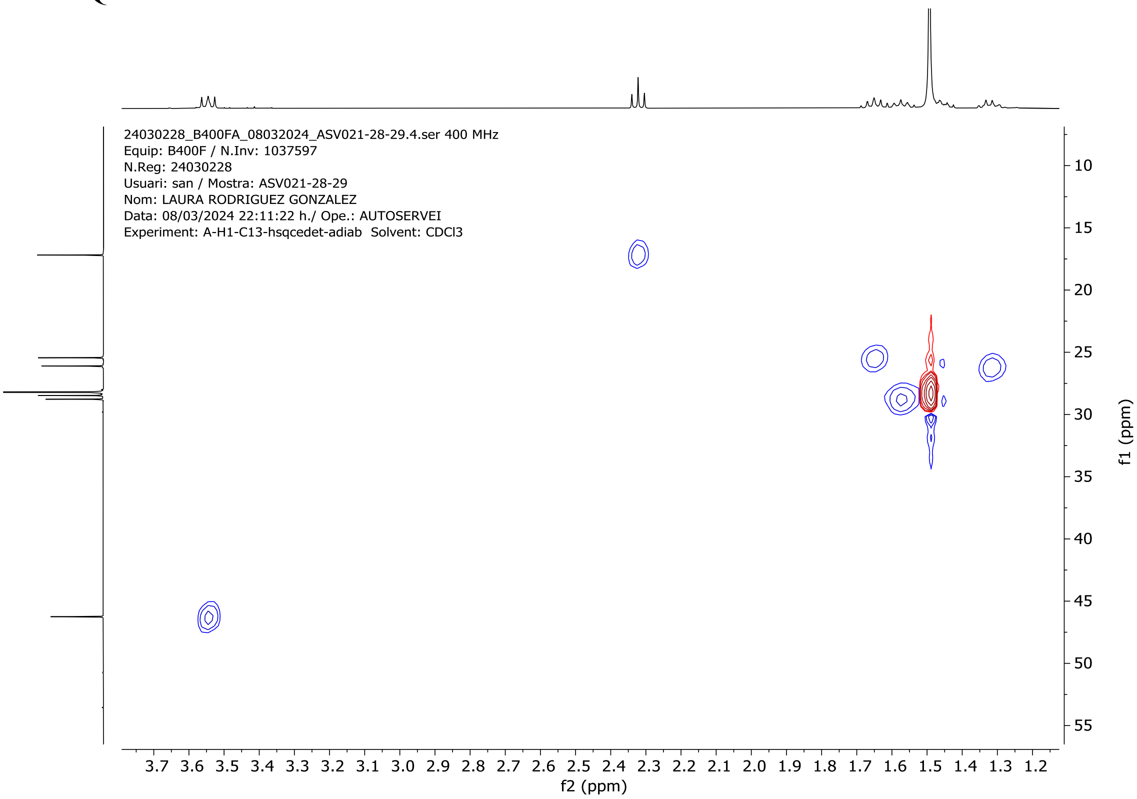

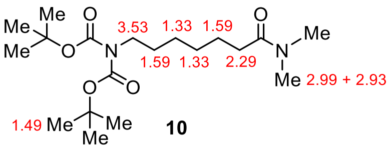

10

24031035_B400FA_22032024_ASV033PREP.1.fid 1H 400 MHz
Equip: B400F / N.Inv: 1037597
N.Reg: 24031035
Usuari: san / Mostra: ASV033PREP
Nom: LAURA RODRIGUEZ GONZALEZ
Data: 22/03/2024 18:03:28 h./ Ope.: AUTOSERVEI
Experiment: A-H1-zg30 Solvent: CDCl3

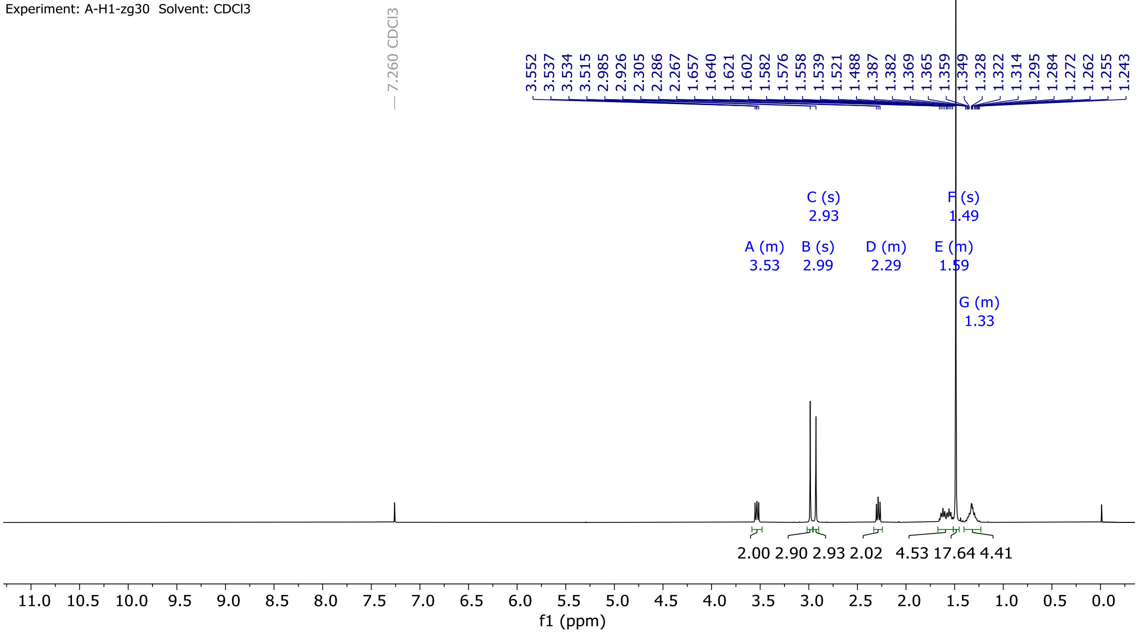

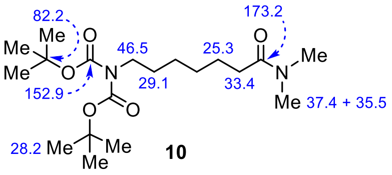

10

24031035_B400FA_23032024_ASV033PREP.2.fid 13C{1H} 101 MHz
Equip: B400F / N.Inv: 1037597
N.Reg: 24031035
Usuari: san / Mostra: ASV033PREP
Nom: LAURA RODRIGUEZ GONZALEZ
Data: 23/03/2024 14:39:37 h./ Ope.: AUTOSERVEI
Experiment: A-C13-zgpg30 Solvent: CDCl3

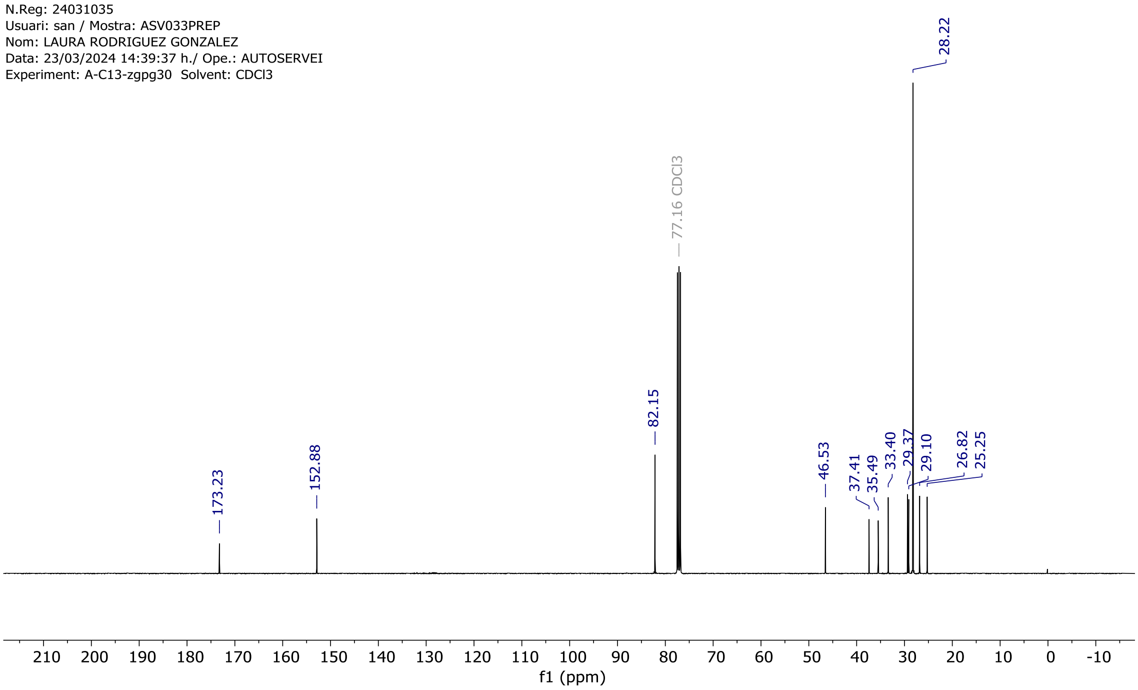

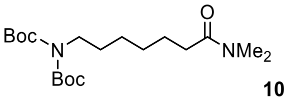

10

2D-COSY

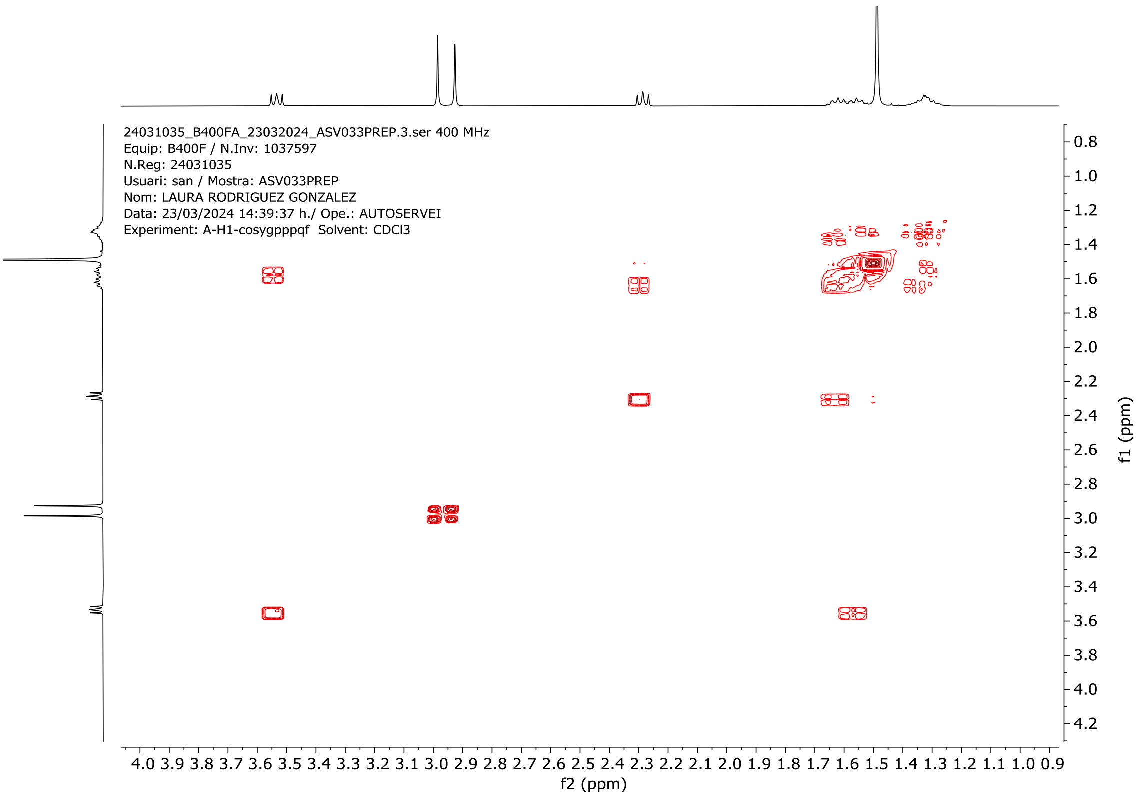

2D-HSQC

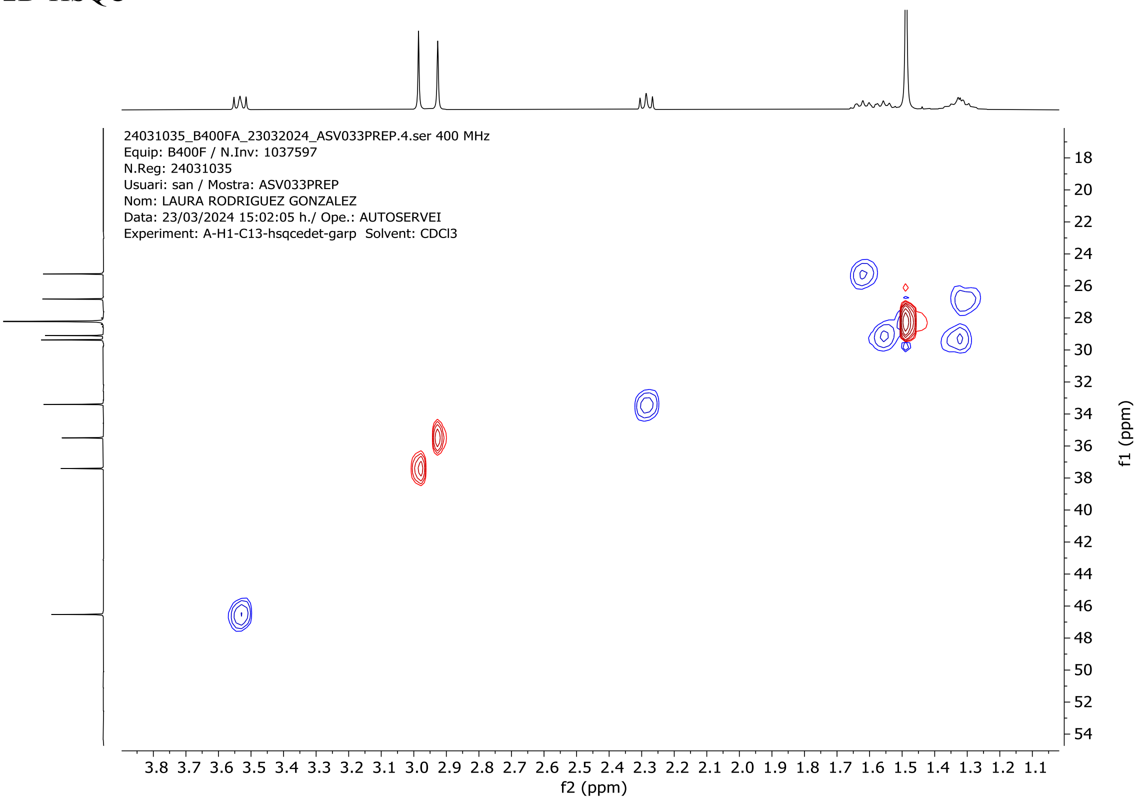

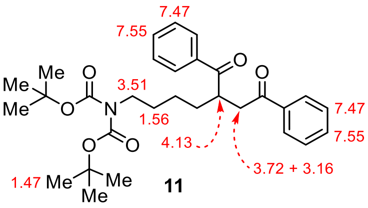

11

24030576_B400FA_16032024_ASV027T25CH.1.fid 1H 400 MHz
Equip: B400F / N.Inv: 1037597
N.Reg: 24030576
Usuari: san / Mostra: ASV027T25CH
Nom: LAURA RODRIGUEZ GONZALEZ
Data: 15/03/2024 17:08:40 h./ Ope.: AUTOSERVEI
Experiment: A-H1-zg30 Solvent: CDCl3

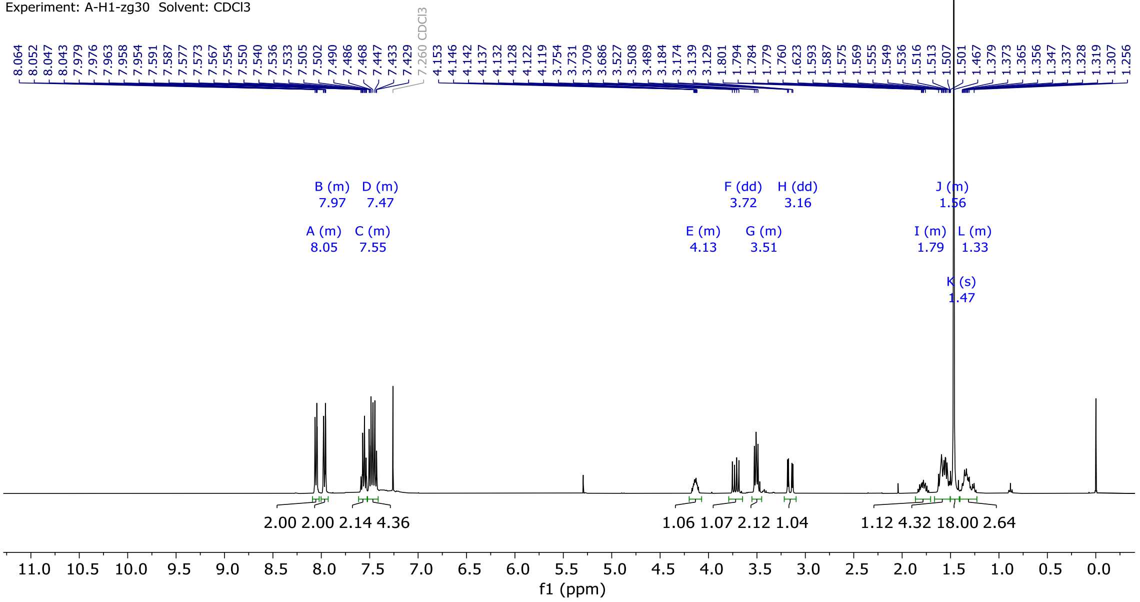

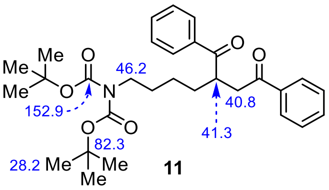

11

24030576_B400FA_16032024_ASV027T25CH.2.fid 13C{1H} 101 MHz
Equip: B400F / N.Inv: 1037597
N.Reg: 24030576
Usuari: san / Mostra: ASV027T25CH
Nom: LAURA RODRIGUEZ GONZALEZ
Data: 16/03/2024 10:17:16 h./ Ope.: AUTOSERVEI
Experiment: A-C13-zgpg30 Solvent: CDCl3

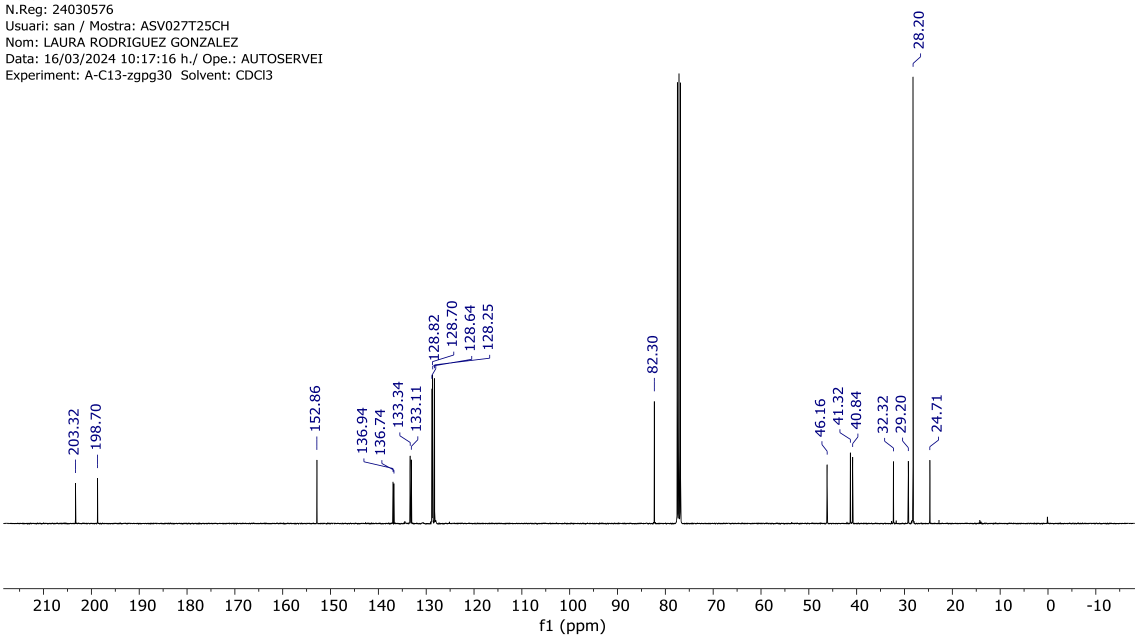

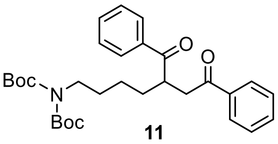

11

2D-COSY

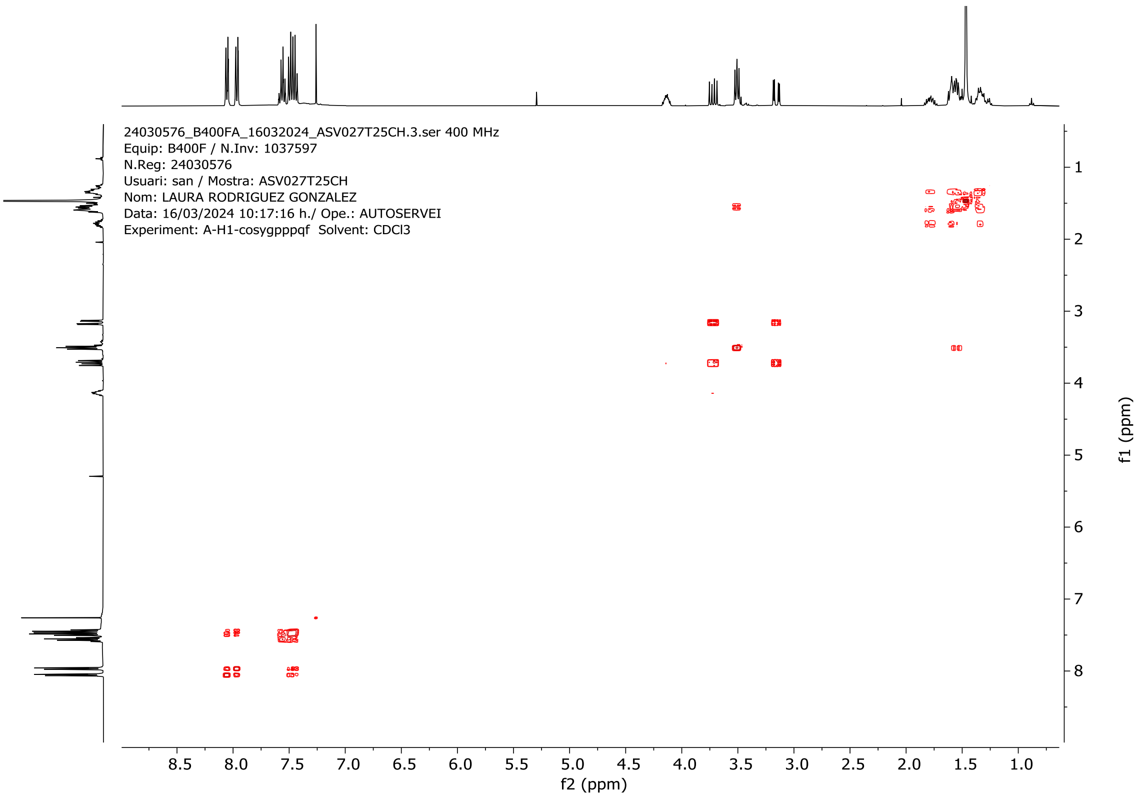

2D-HSQC

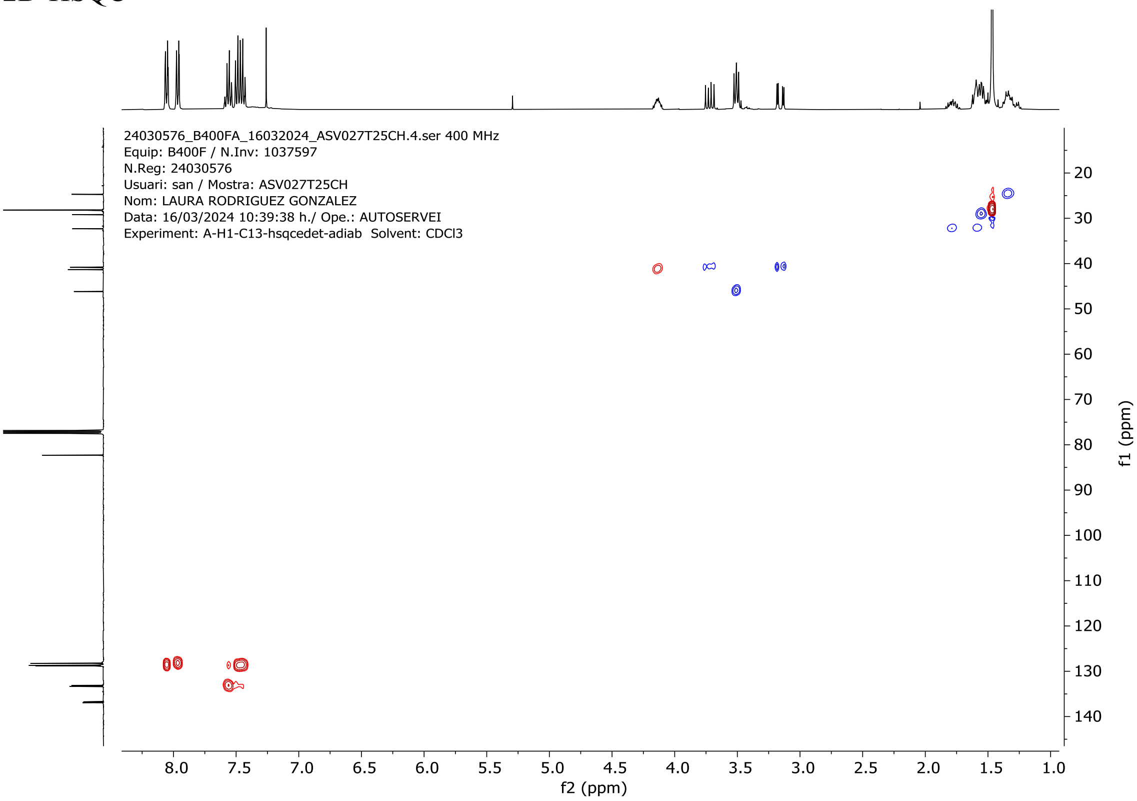

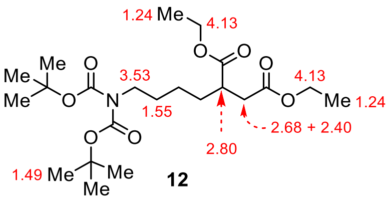

12

24030464_B400FA_14032024_LRG567COLT29.1.fid 1H 400 MHz
Equip: B400F / N.Inv: 1037597
N.Reg: 24030464
Usuari: san / Mostra: LRG567COLT29
Nom: LAURA RODRIGUEZ GONZALEZ
Data: 14/03/2024 12:23:46 h./ Ope.: AUTOSERVEI
Experiment: A-H1-zg30 Solvent: CDCl3

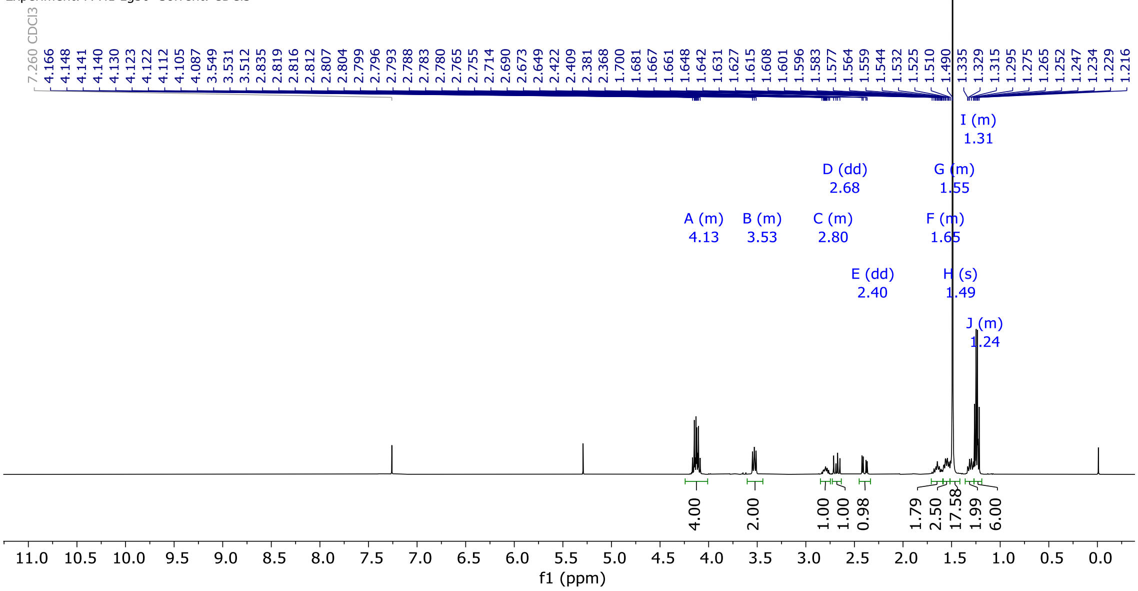

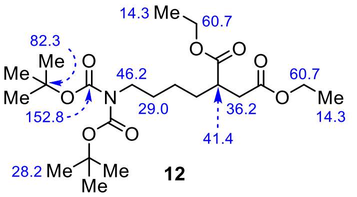

12

24030464_B400FA_15032024_LRG567COLT29.2.fid 13C{1H} 101 MHz
Equip: B400F / N.Inv: 1037597
N.Reg: 24030464
Usuari: san / Mostra: LRG567COLT29
Nom: LAURA RODRIGUEZ GONZALEZ
Data: 15/03/2024 02:40:21 h./ Ope.: AUTOSERVEI
Experiment: A-C13-zgpg30 Solvent: CDCl3

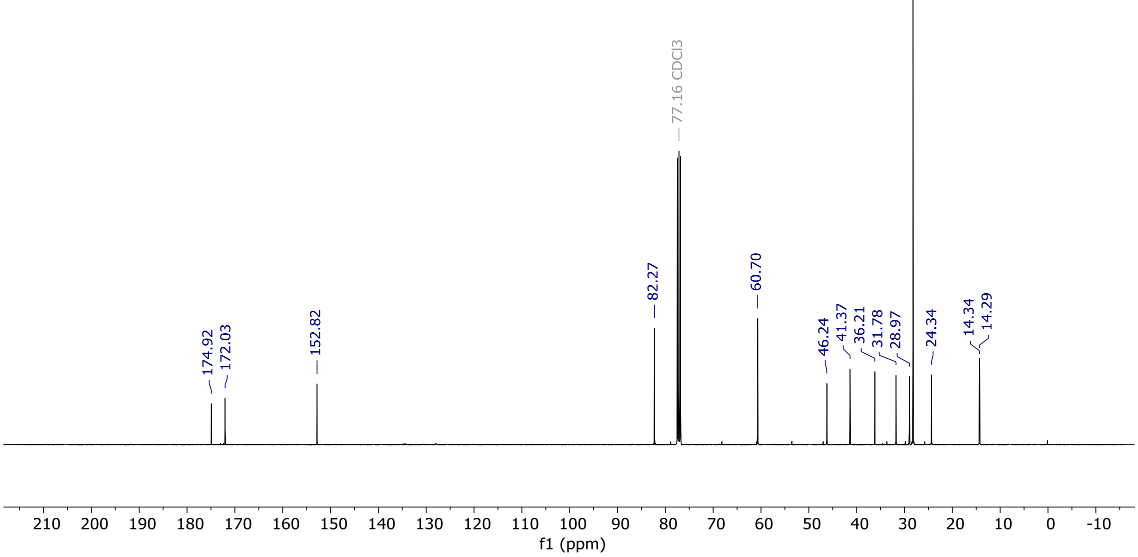

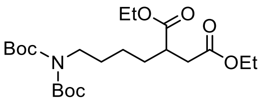

12

2D-COSY

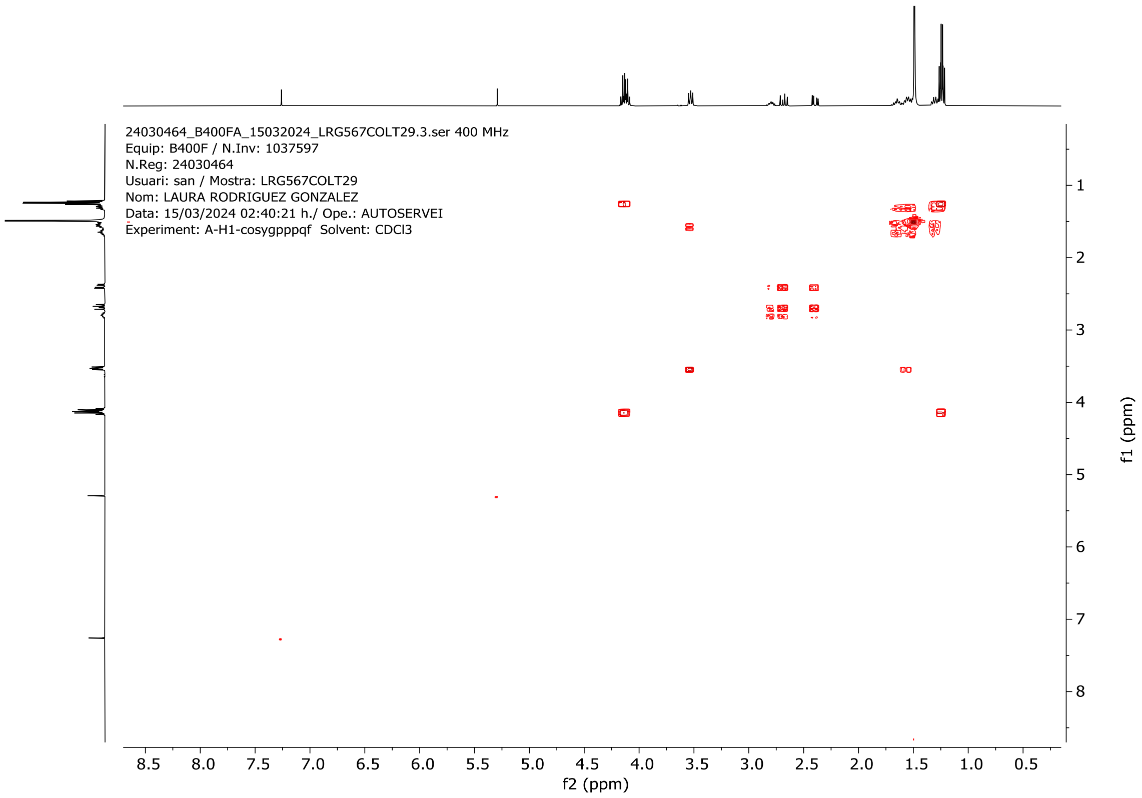

2D-HSQC

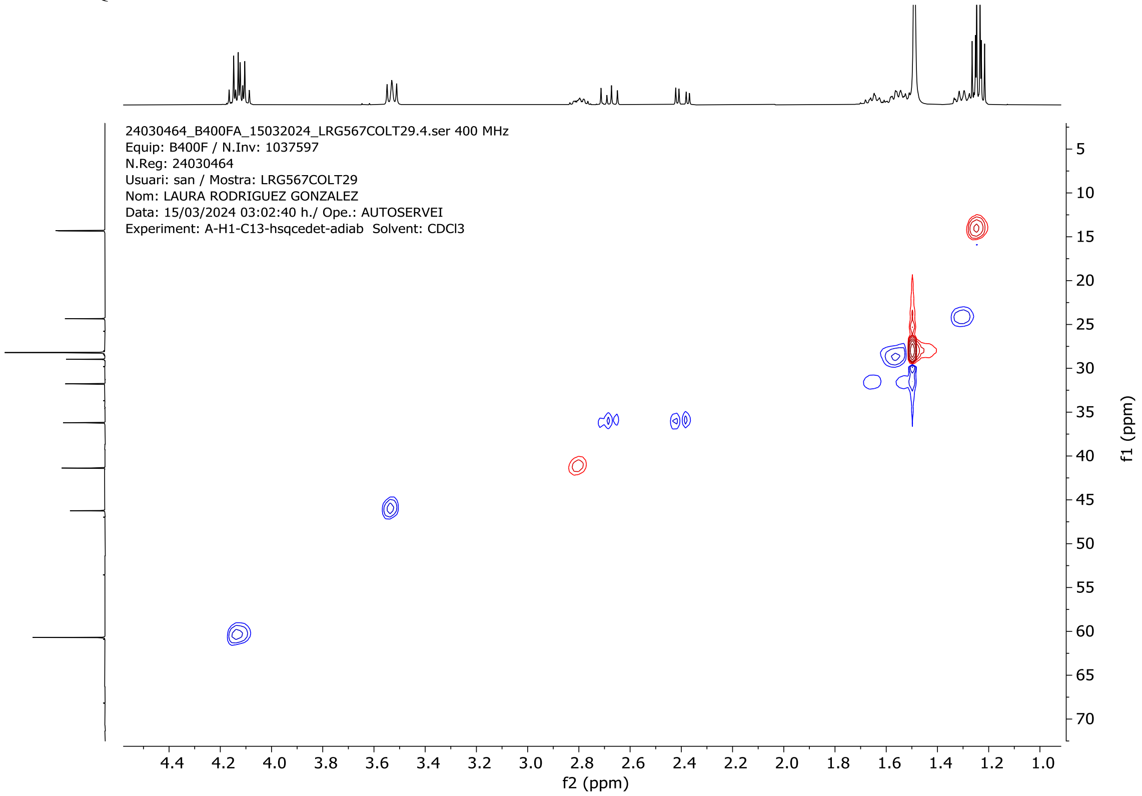

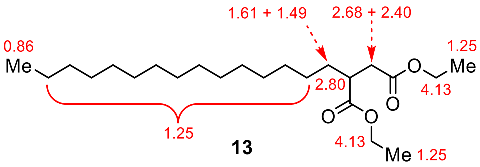

24040027_B400FA_02042024_ASV032-prep.1.fid 1H 400 MHz
Equip: B400F / N.Inv: 1037597
N.Reg: 24040027
Usuari: san / Mostra: ASV032-prep
Nom: AINA SERRA VERT
Data: 02/04/2024 15:08:09 h./ Ope.: AUTOSERVEI
Experiment: A-H1-zg30 Solvent: CDCl3

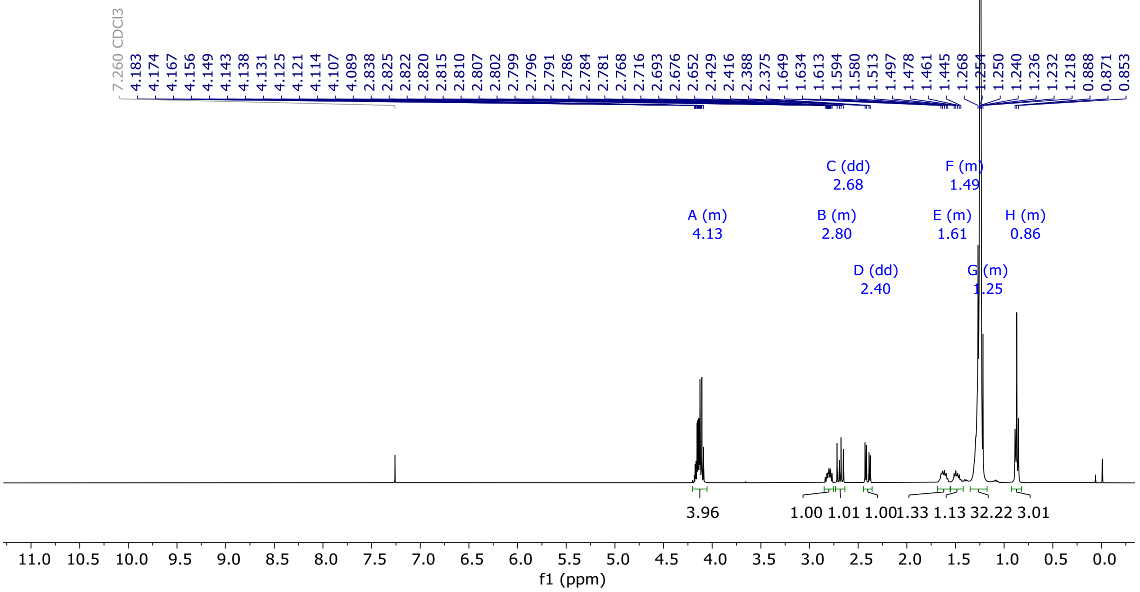

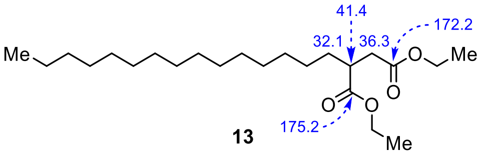

24040027_B400FA_02042024_ASV032-prep.2.fid 13C{1H} 101 MHz
Equip: B400F / N.Inv: 1037597
N.Reg: 24040027
Usuari: san / Mostra: ASV032-prep
Nom: AINA SERRA VERT
Data: 02/04/2024 22:58:04 h./ Ope.: AUTOSERVEI
Experiment: A-C13-zgpg30 Solvent: CDCl3

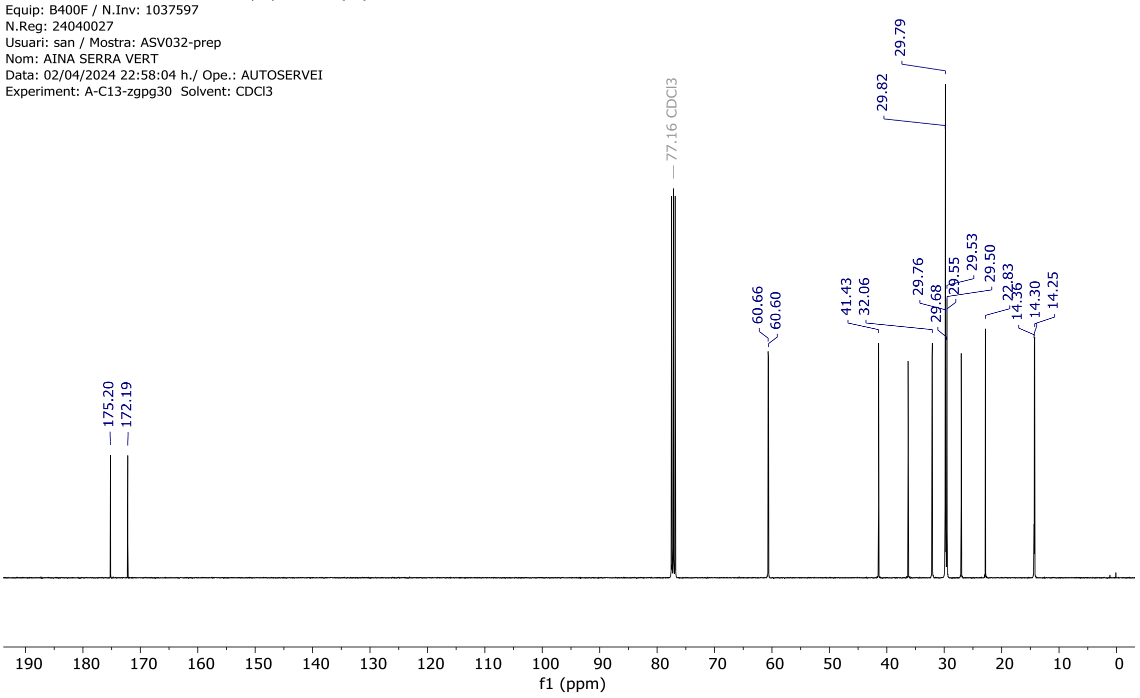

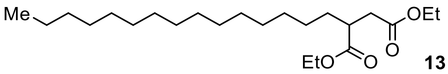

13

2D-COSY

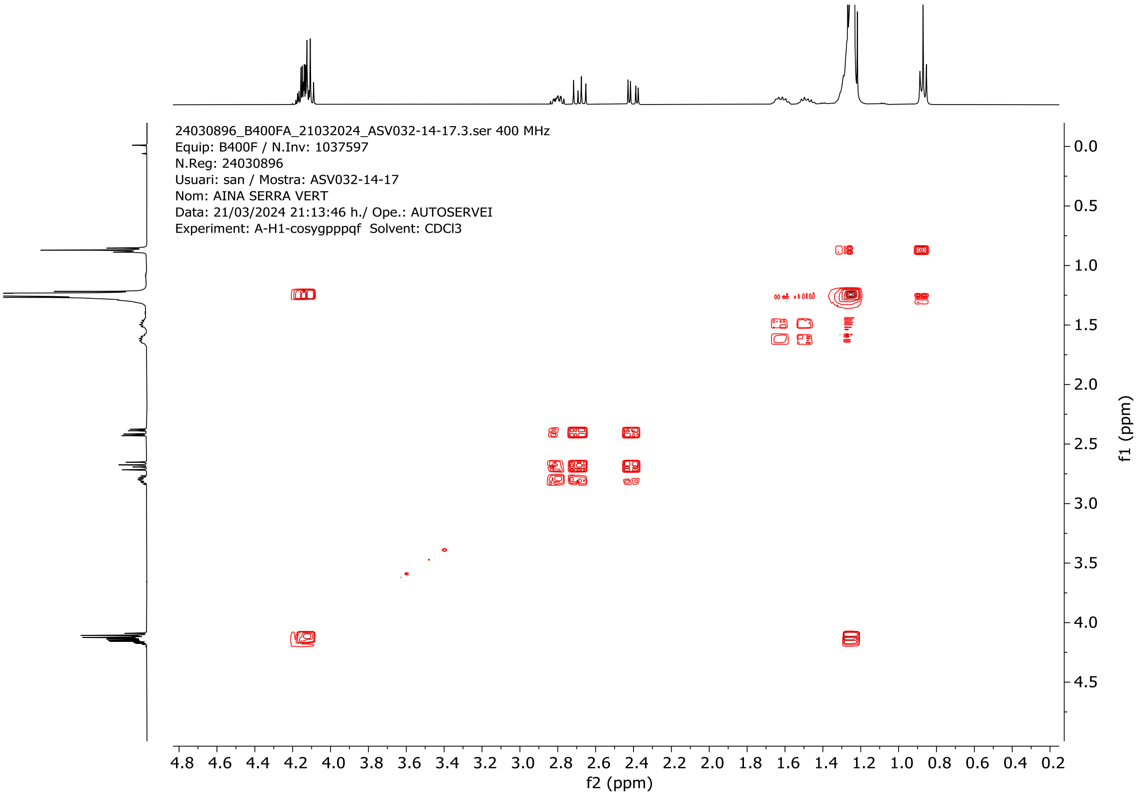

2D-HSQC

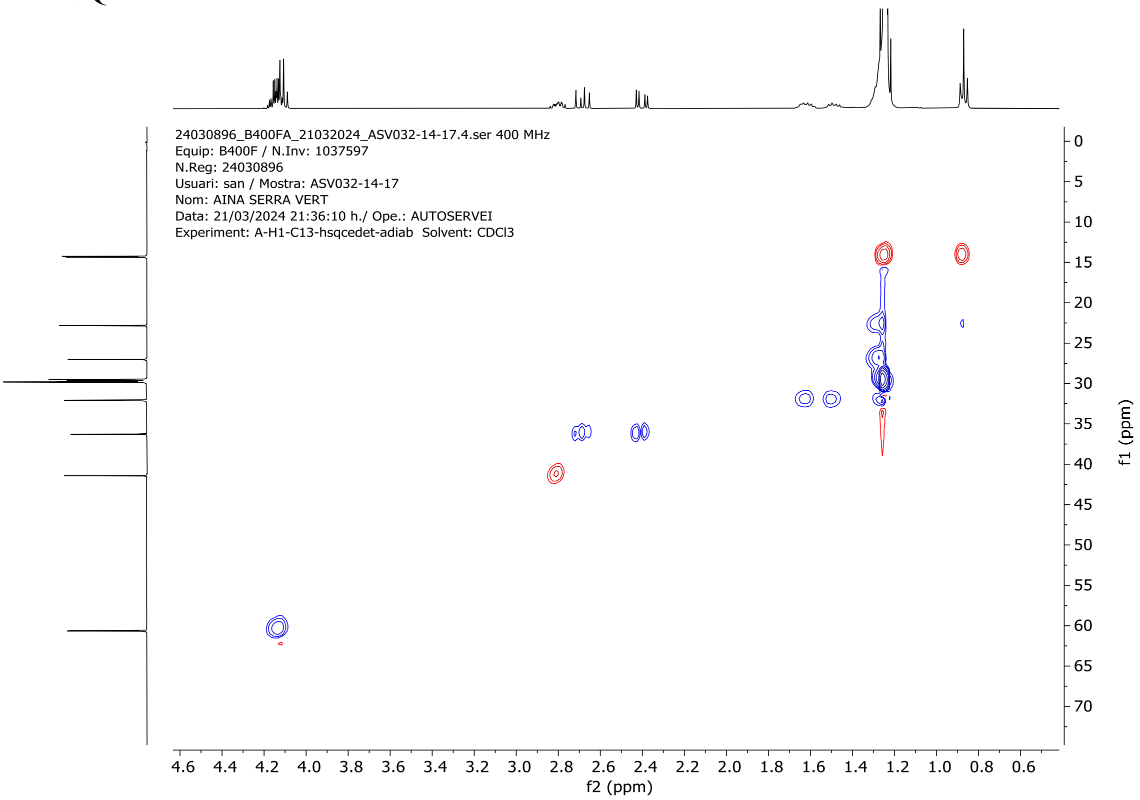

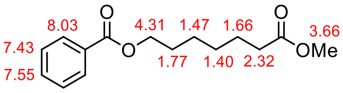

14

5924-2024_B500QA_14062024_ASV062CH.10.fid 1H 500 MHz
Equip: B500Q / N.Inv: 1028917
N.Reg: 5924/2024
Usuari: san / Mostra: ASV062CH
Nom: LAURA RODRIGUEZ GONZALEZ
Data: 14/06/2024 18:36:45 h./ Ope.: servei Unitat RMN
Experiment: A_1H-zg30 Solvent: CDCl3 Operator: VICTOR MERIEL

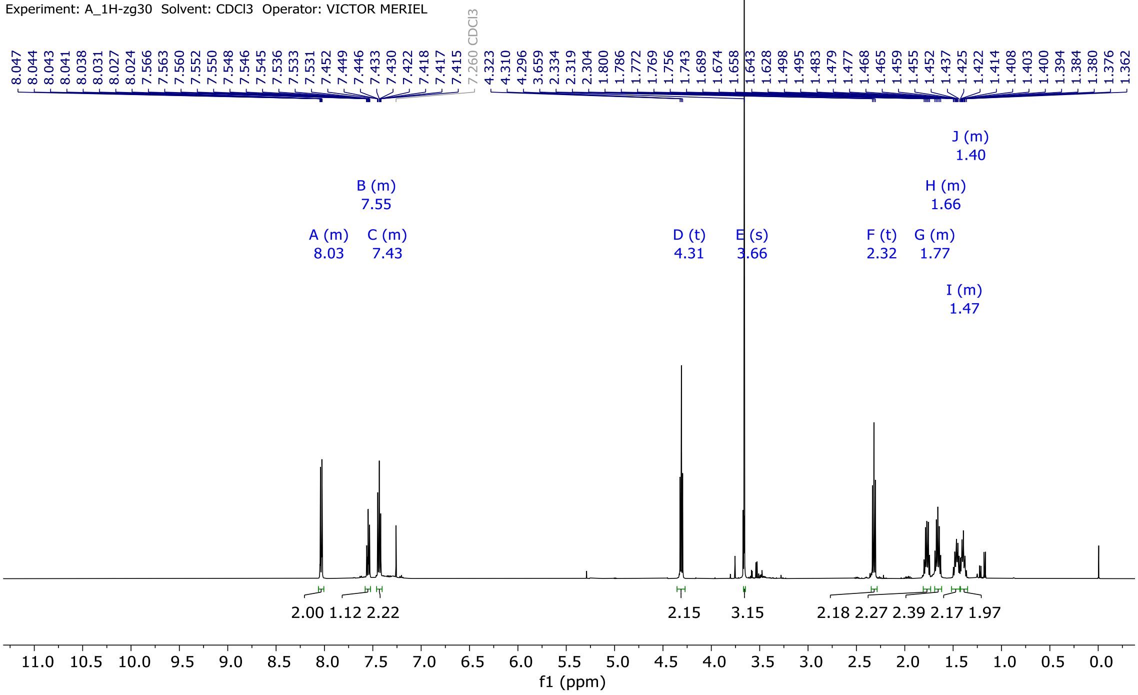

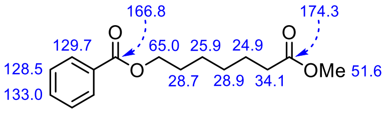

14

5924-2024_B500QA_14062024_ASV062CH.11.fid 13C{1H} 126 MHz
Equip: B500Q / N.Inv: 1028917
N.Reg: 5924/2024
Usuari: san / Mostra: ASV062CH
Nom: LAURA RODRIGUEZ GONZALEZ
Data: 14/06/2024 18:36:45 h./ Ope.: servei Unitat RMN
Experiment: A_13C-zgpg30 Solvent: CDCl3 Operator: VICTOR MERIEL

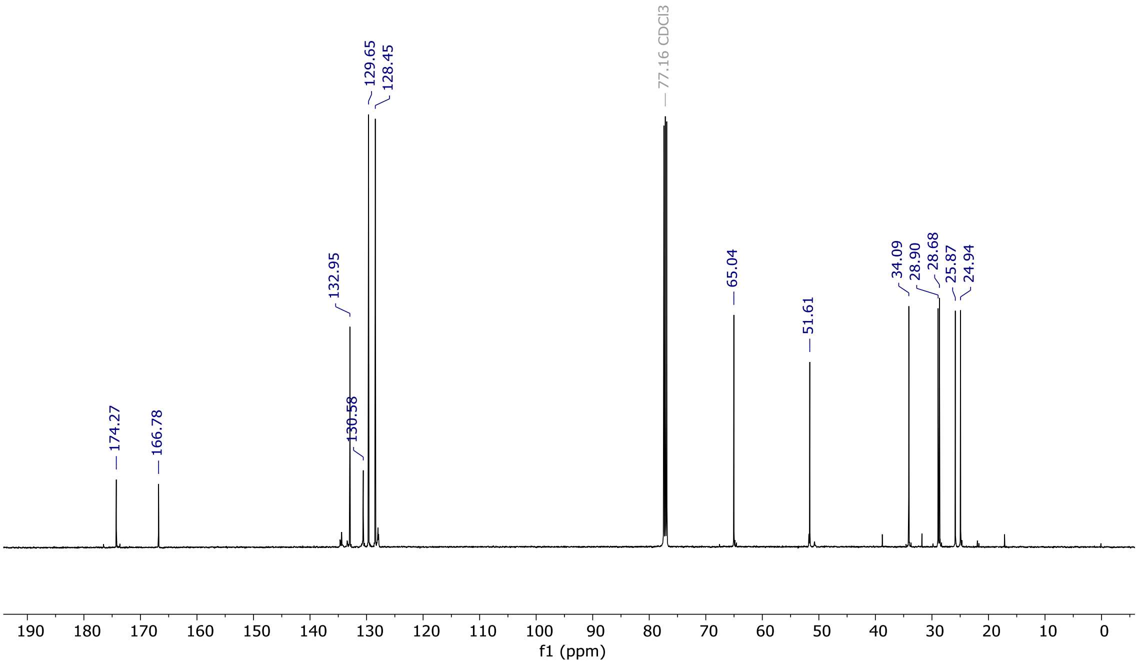

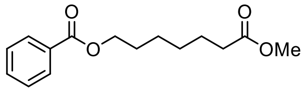

14

2D-COSY

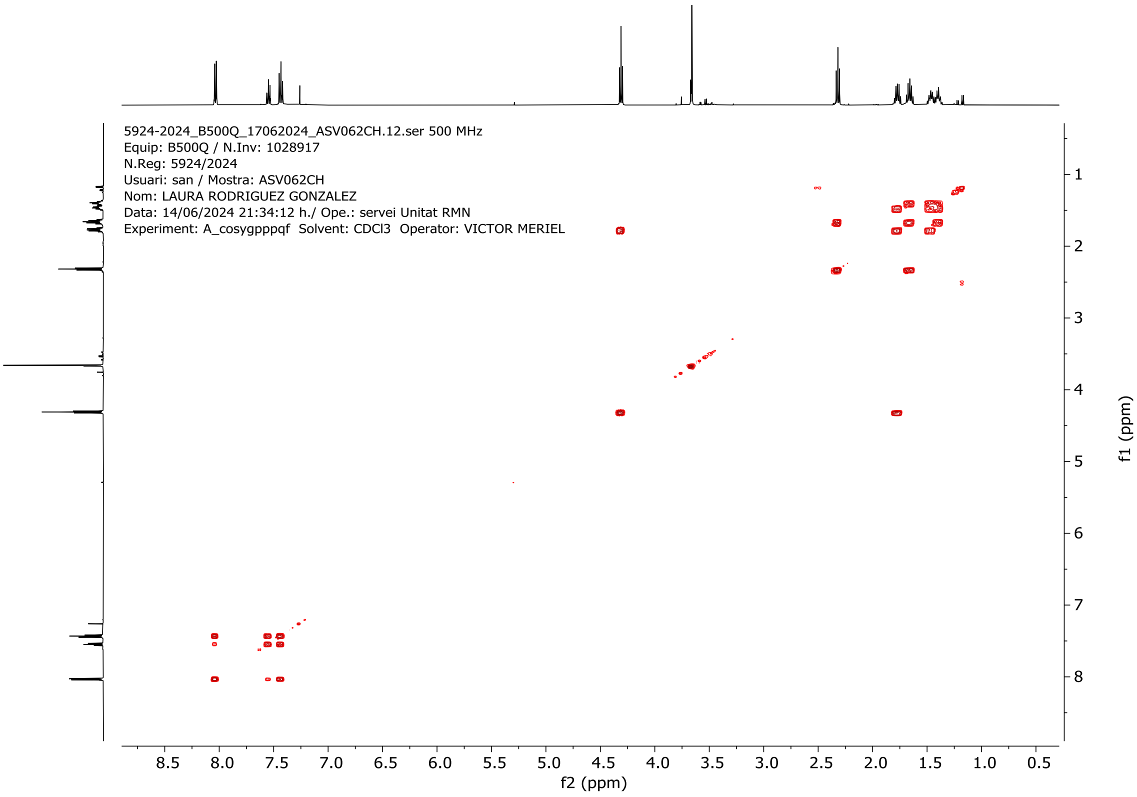

2D-HSQC

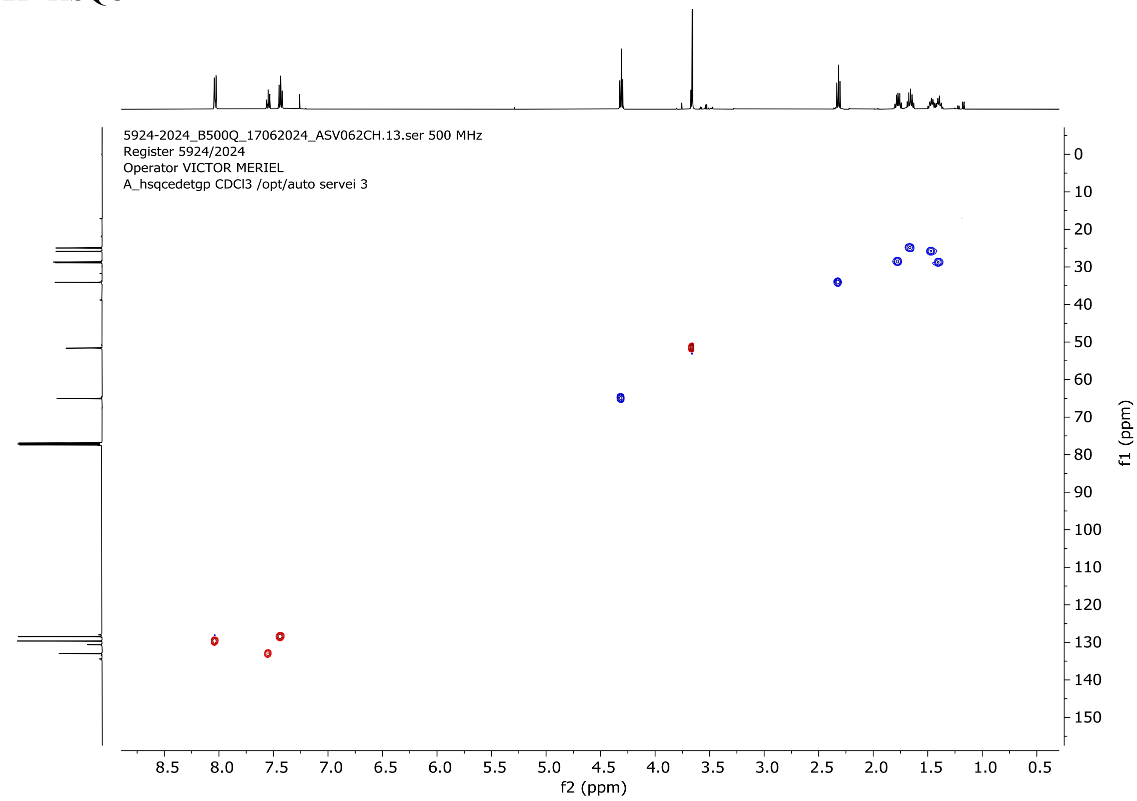

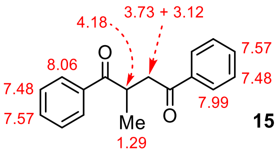

15

auto-21062024-113955.1.fid 1H 400 MHz
Equip: B400Q / N.Inv: 1035091
N.Reg: 24061009
Usuari: san / Mostra: LRG615CH
Nom: LAURA RODRIGUEZ GONZALEZ
Data: 21/06/2024 11:52:04 h./ Ope.: AUTOSERVEI
Experiment: A_1H-zg30 Solvent: CDCl3 Operator:

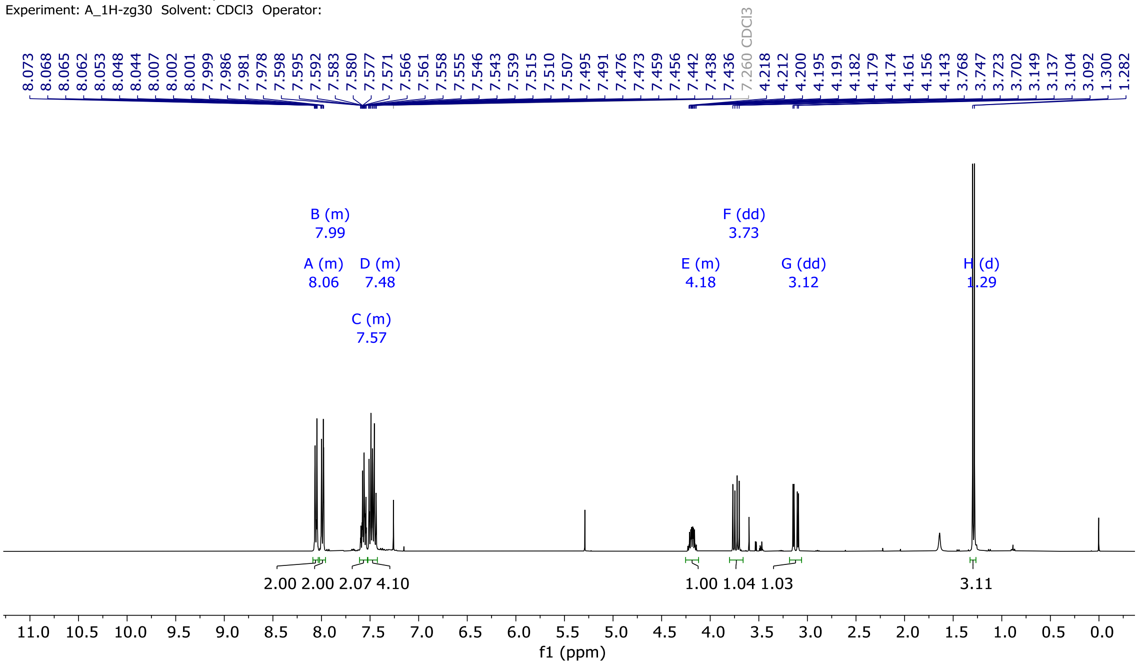

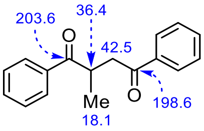

15

auto-21062024-113955.2.fid 13C{1H} 101 MHz
Equip: B400Q / N.Inv: 1035091
N.Reg: 24061009
Usuari: san / Mostra: LRG615CH
Nom: LAURA RODRIGUEZ GONZALEZ
Data: 22/06/2024 16:44:52 h./ Ope.: AUTOSERVEI
Experiment: A_13C-zgpg30 Solvent: CDCl3 Operator:

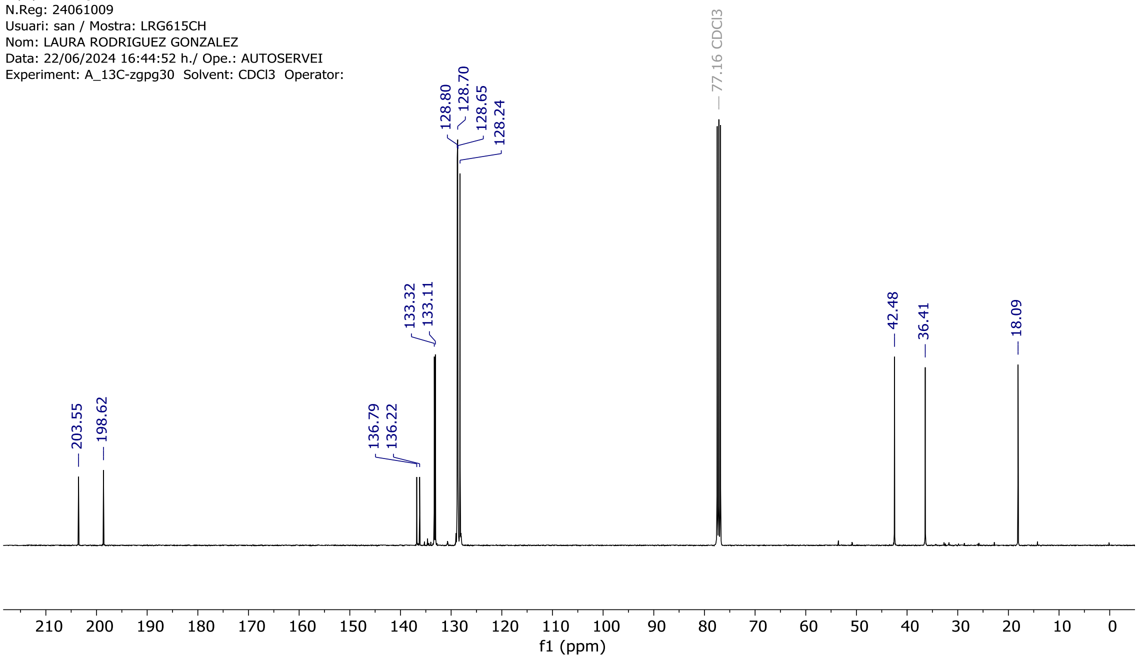

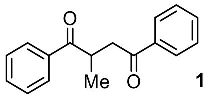

15

2D-COSY

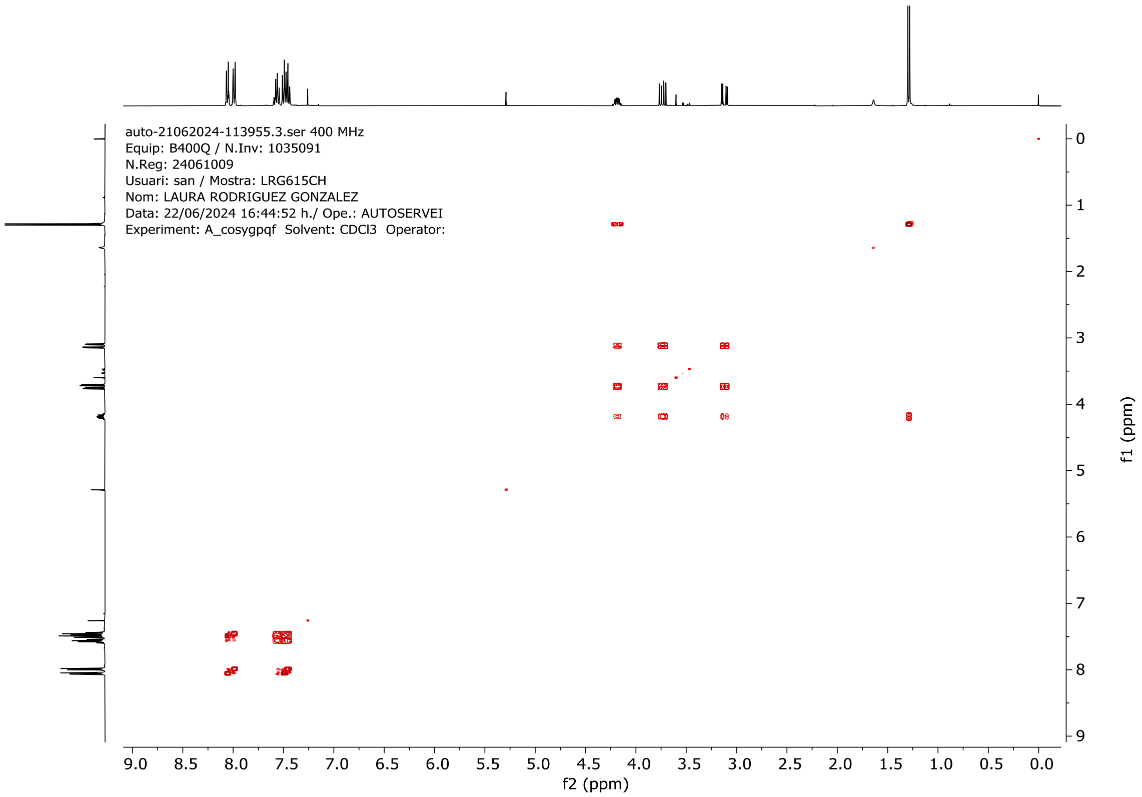

2D-HSQC

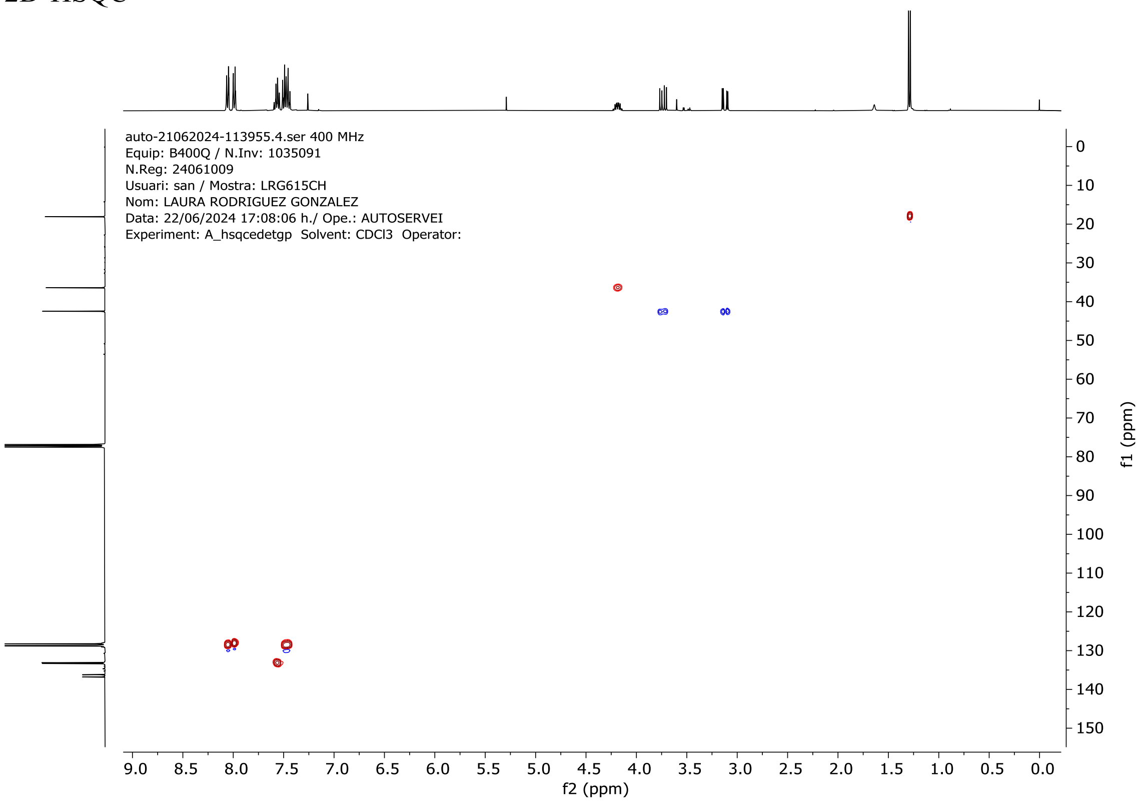

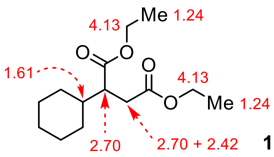

16

24040210_B400FA_05042024_ASV034PREP.1.fid 1H 400 MHz
Equip: B400F / N.Inv: 1037597
N.Reg: 24040210
Usuari: san / Mostra: ASV034PREP
Nom: LAURA RODRIGUEZ GONZALEZ
Data: 05/04/2024 11:21:41 h./ Ope.: AUTOSERVEI
Experiment: A-H1-zg30 Solvent: CDCl3

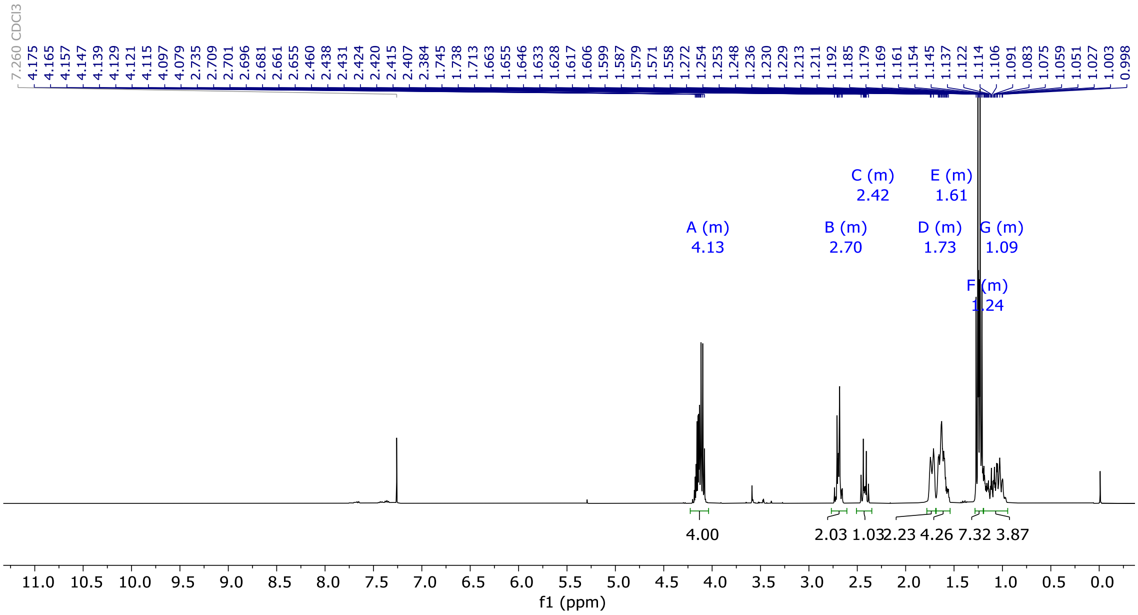

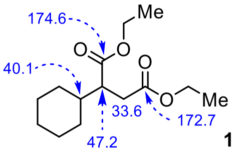

16

san-3850-2024.2.fid 13C{1H} 101 MHz
Equip: B400Q / N.Inv: 1035091
N.Reg: 3850/2024
Usuari: san / Mostra: ASV034PREP
Nom: LAURA RODRIGUEZ GONZALEZ
Data: 08/04/2024 16:38:03 h./ Ope.: servei Unitat RMN
Experiment: A_13C-zgpg30 Solvent: CDCl3 Operator: VICTOR MERIEL

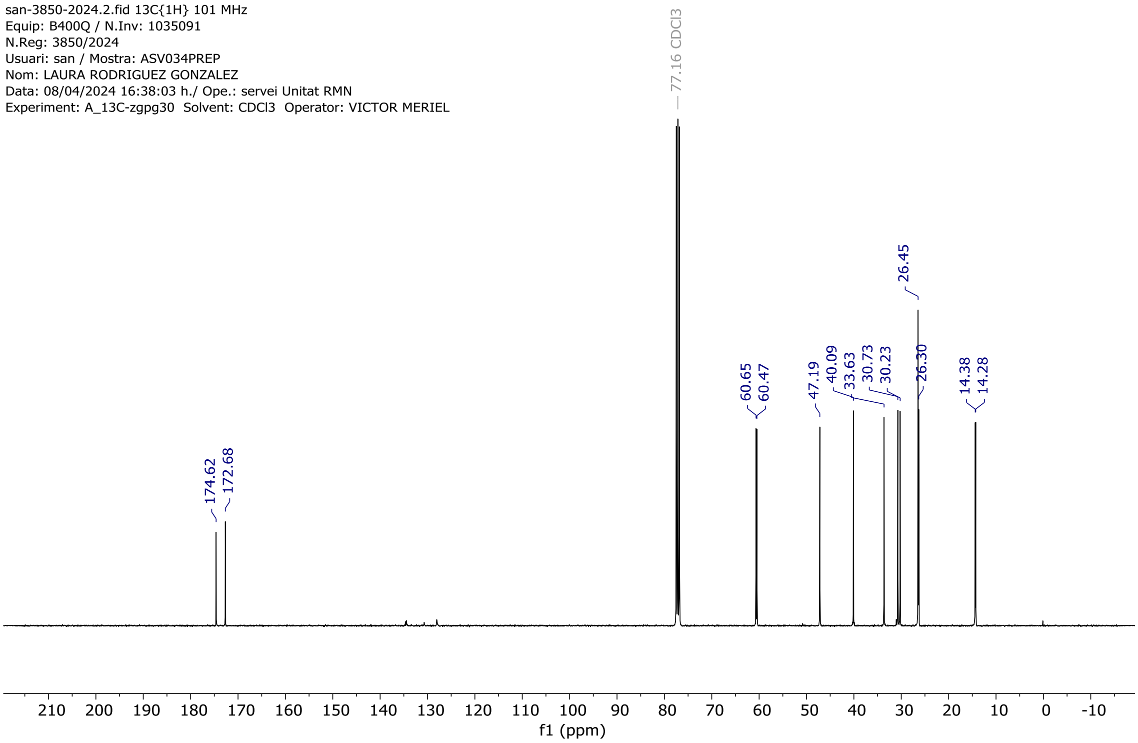

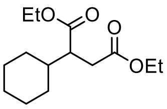

16

2D-COSY

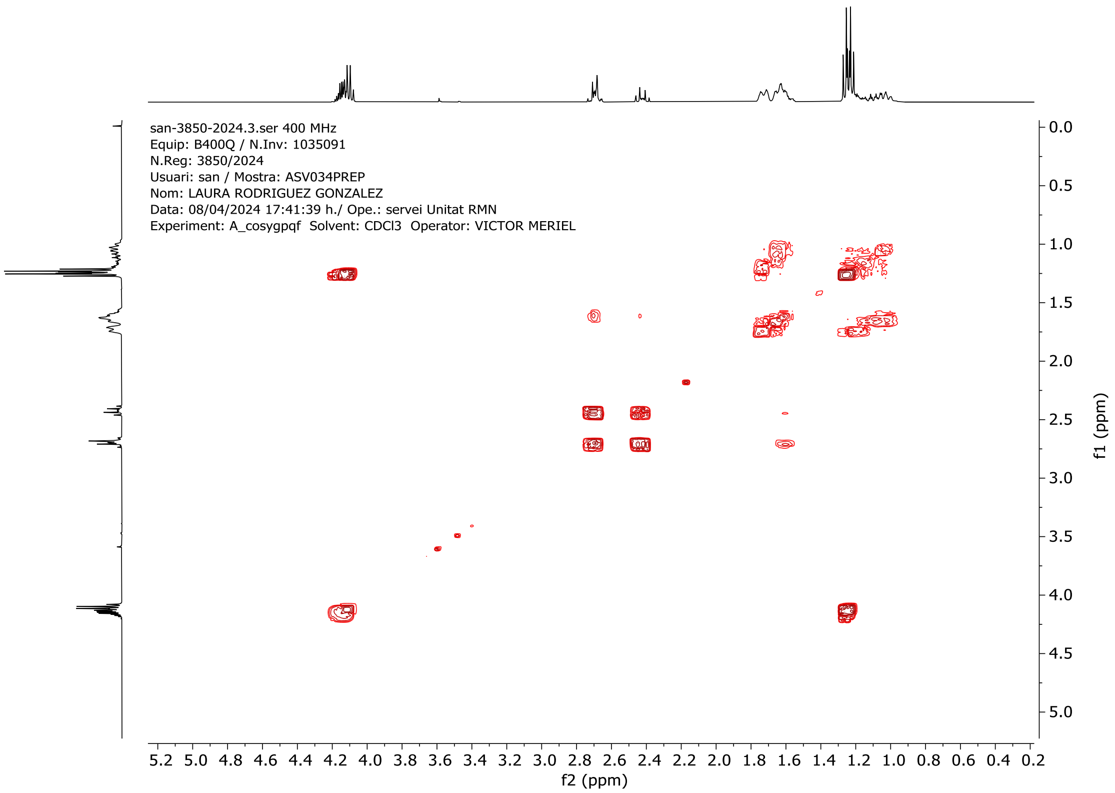

2D-HSQC

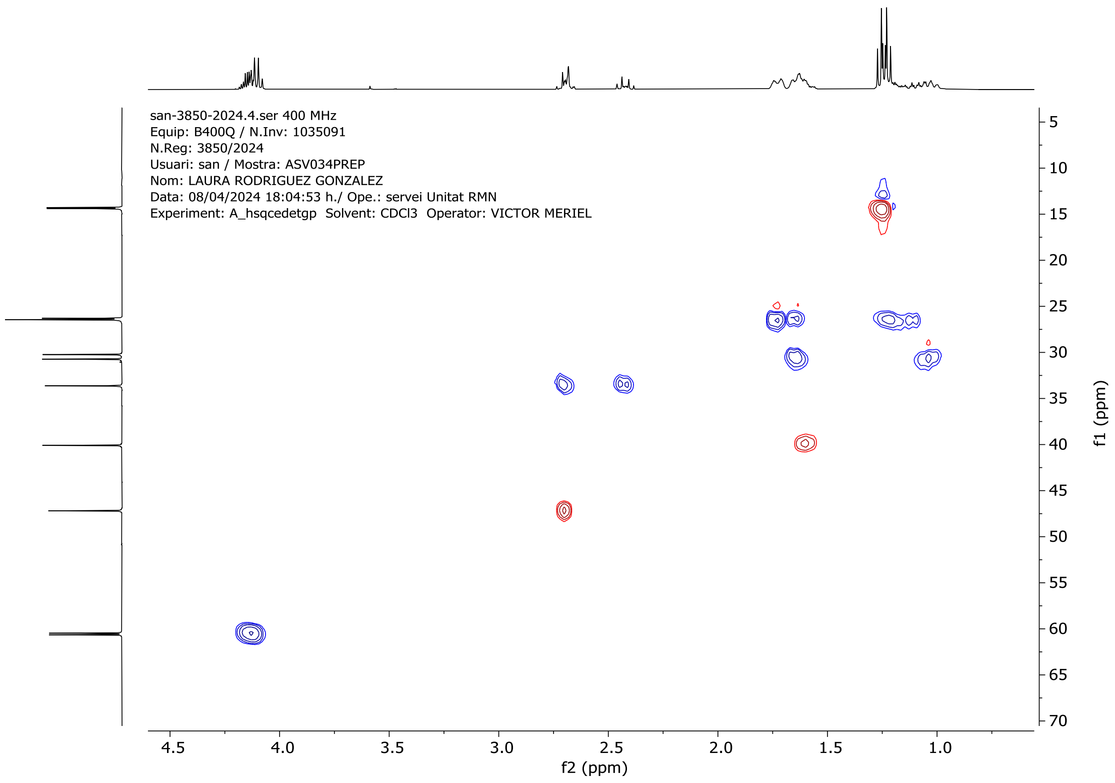

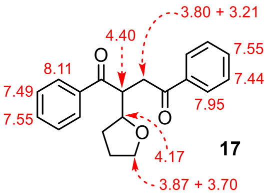

24040898_B400FA_19042024_ASV044PREPMID.1.fid 1H 400 MHz
Equip: B400F / N.Inv: 1037597
N.Reg: 24040898
Usuari: san / Mostra: ASV044PREPMID
Nom: LAURA RODRIGUEZ GONZALEZ
Data: 19/04/2024 16:09:56 h./ Ope.: AUTOSERVEI
Experiment: A-H1-zg30 Solvent: CDCl3

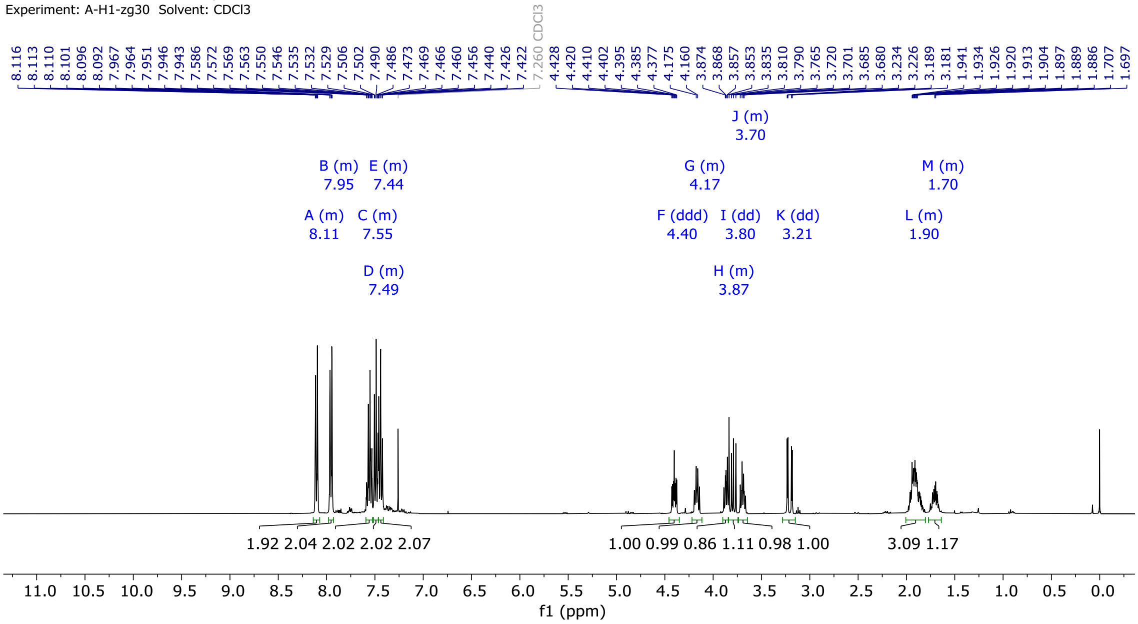

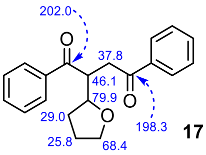

24040898_B400FA_20042024_ASV044PREPMID.2.fid 13C{1H} 101 MHz
Equip: B400F / N.Inv: 1037597
N.Reg: 24040898
Usuari: san / Mostra: ASV044PREPMID
Nom: LAURA RODRIGUEZ GONZALEZ
Data: 20/04/2024 16:16:09 h./ Ope.: AUTOSERVEI
Experiment: A-C13-zgpg30 Solvent: CDCl3

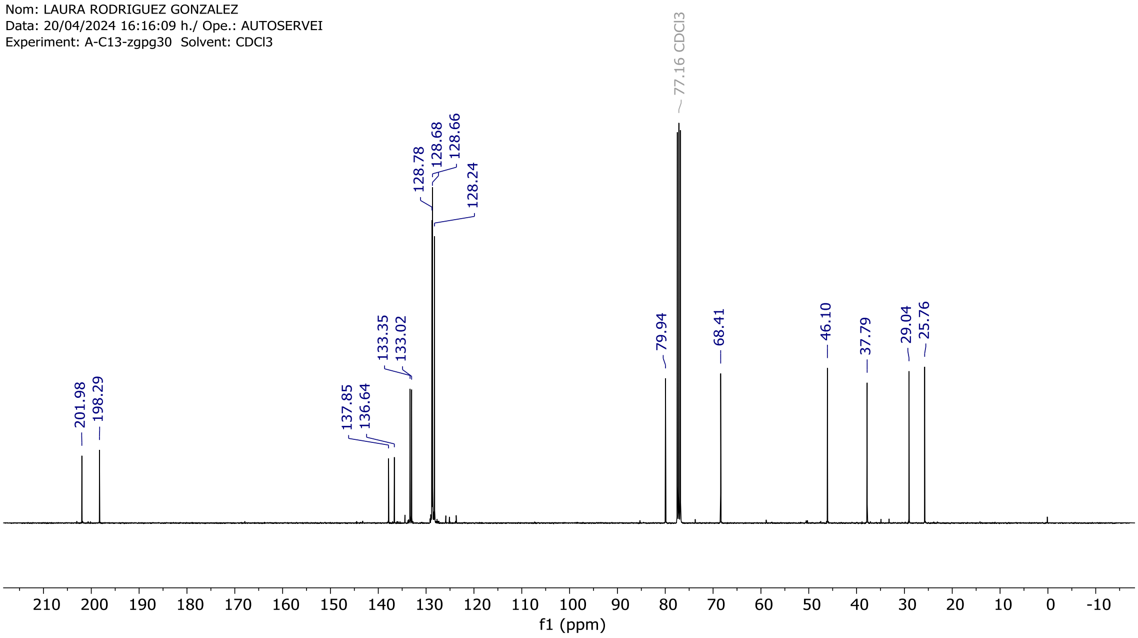

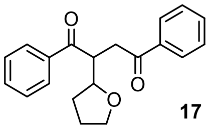

17

2D-COSY

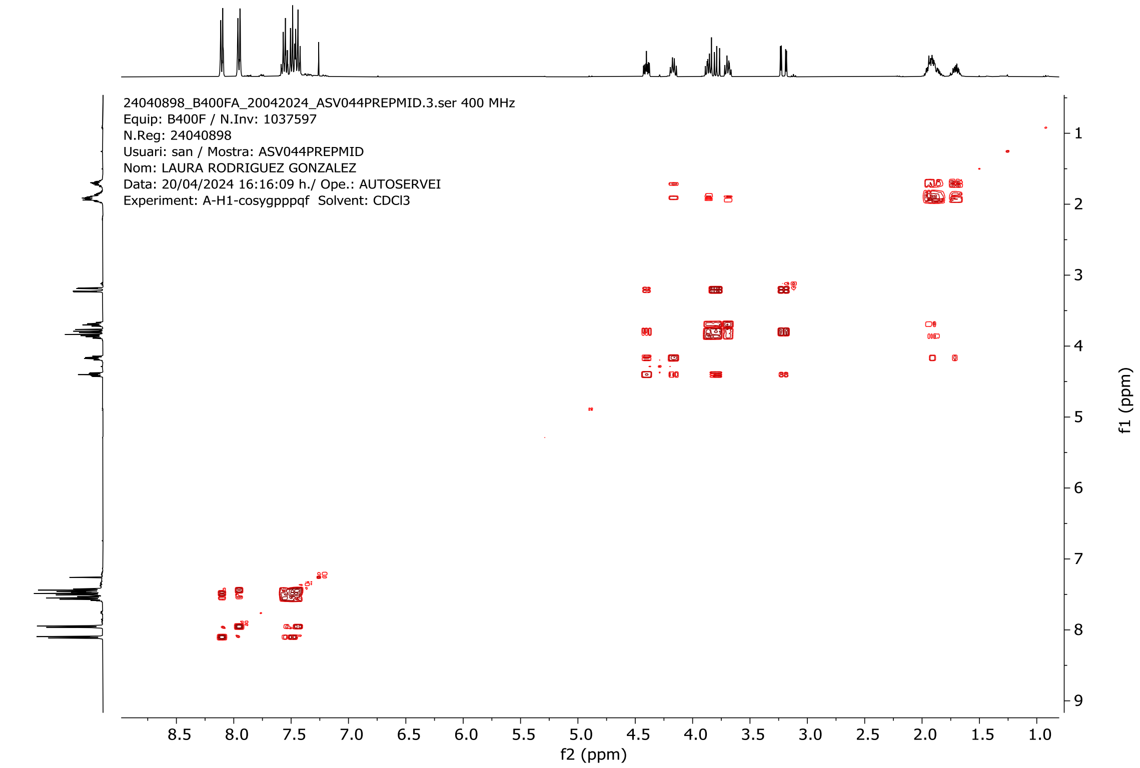

2D-HSQC

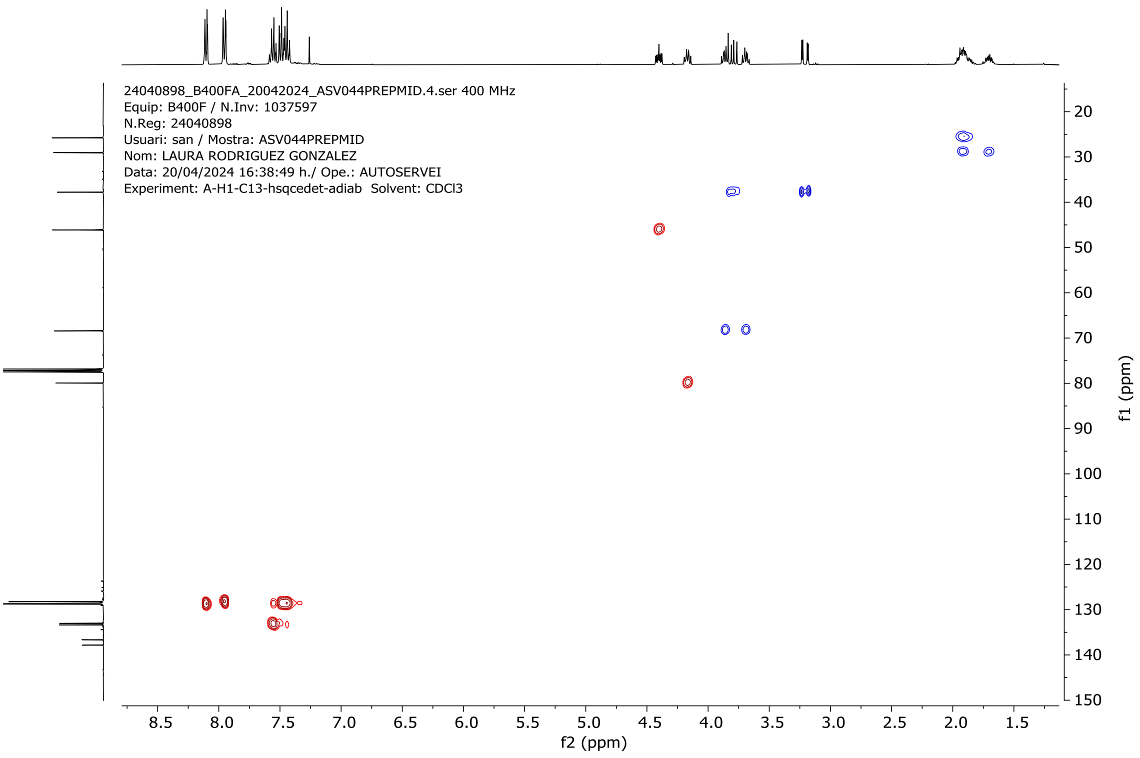

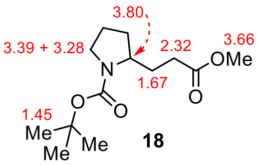

18

24040554_B400FA_12042024_ASV039-PREP.1.fid 1H 400 MHz
Equip: B400F / N.Inv: 1037597
N.Reg: 24040554
Usuari: san / Mostra: ASV039-PREP
Nom: AINA SERRA VERT
Data: 12/04/2024 11:17:58 h./ Ope.: AUTOSERVEI
Experiment: A-H1-zg30 Solvent: CDCl3

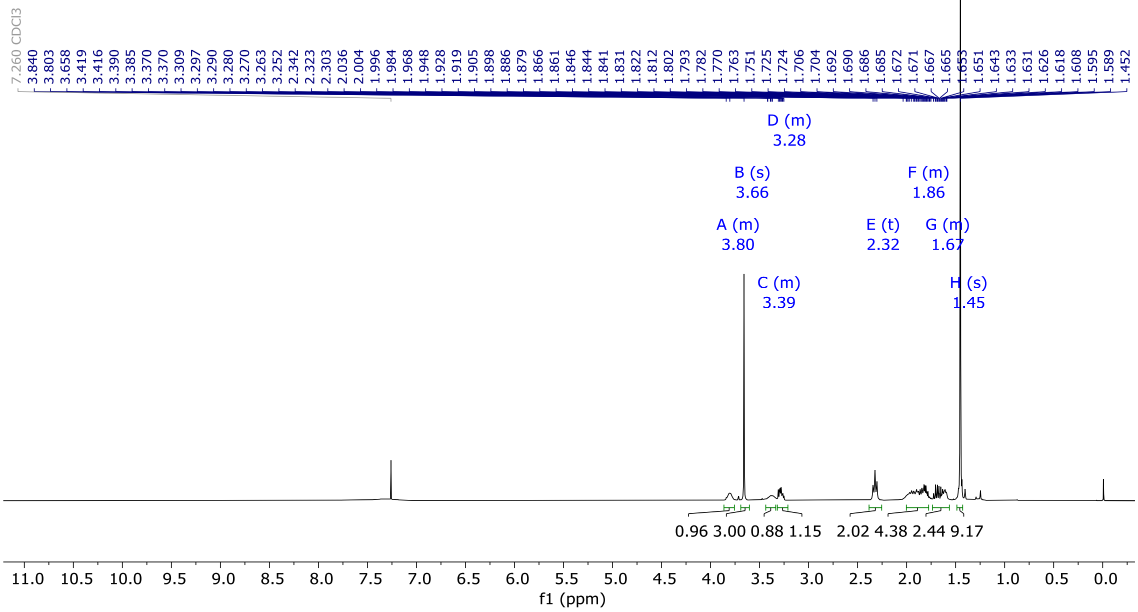

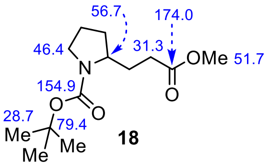

18

24040554_B400FA_14042024_ASV039-PREP.3.fid 13C{1H} 101 MHz
Equip: B400F / N.Inv: 1037597
N.Reg: 24040554
Usuari: san / Mostra: ASV039-PREP
Nom: AINA SERRA VERT
Data: 14/04/2024 07:41:06 h./ Ope.: AUTOSERVEI
Experiment: A-C13-zgpg30 Solvent: CDCl3

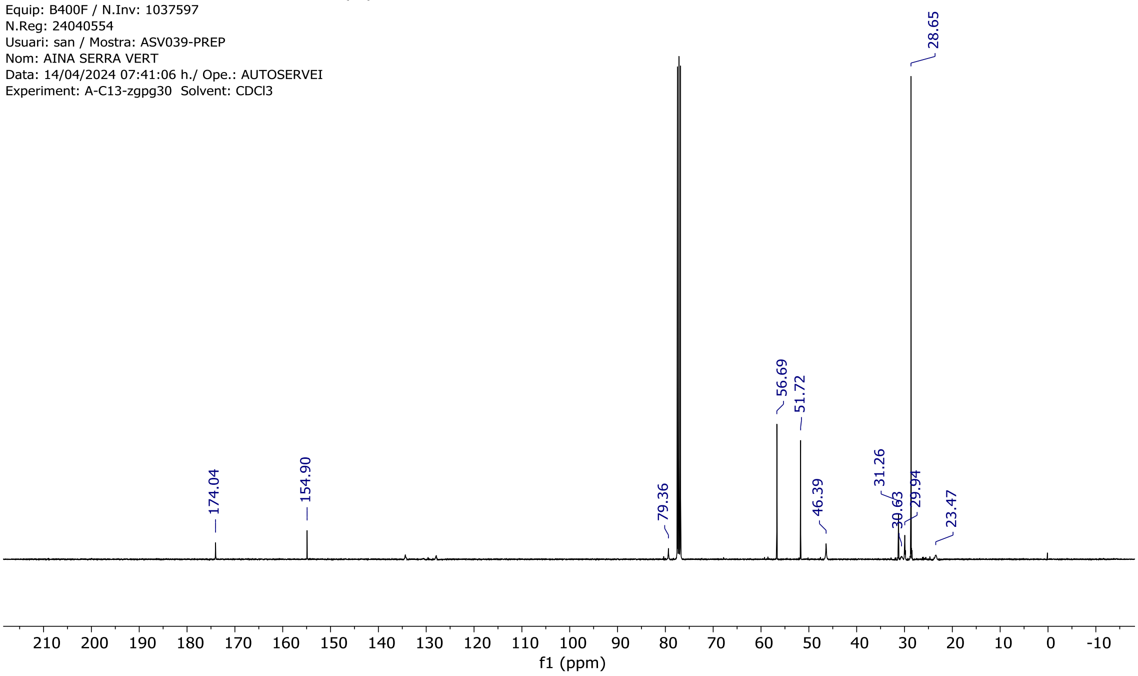

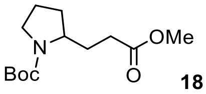

18

2D-COSY

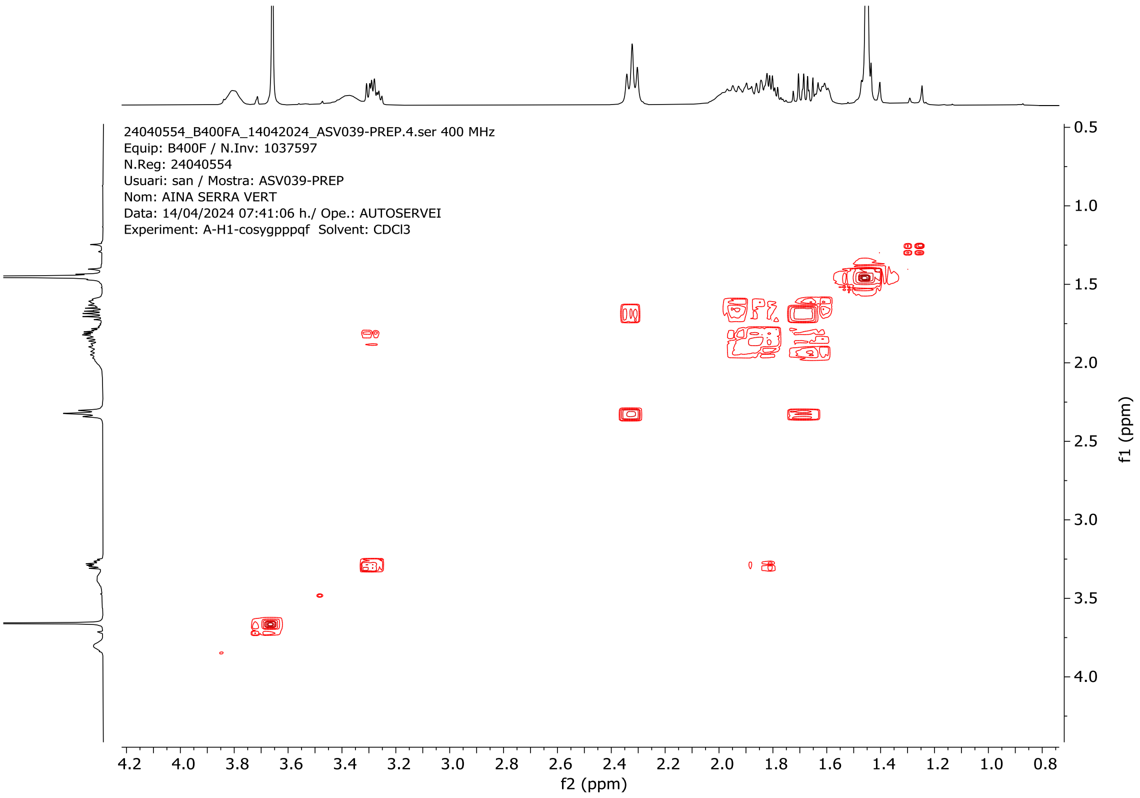

2D-HSQC

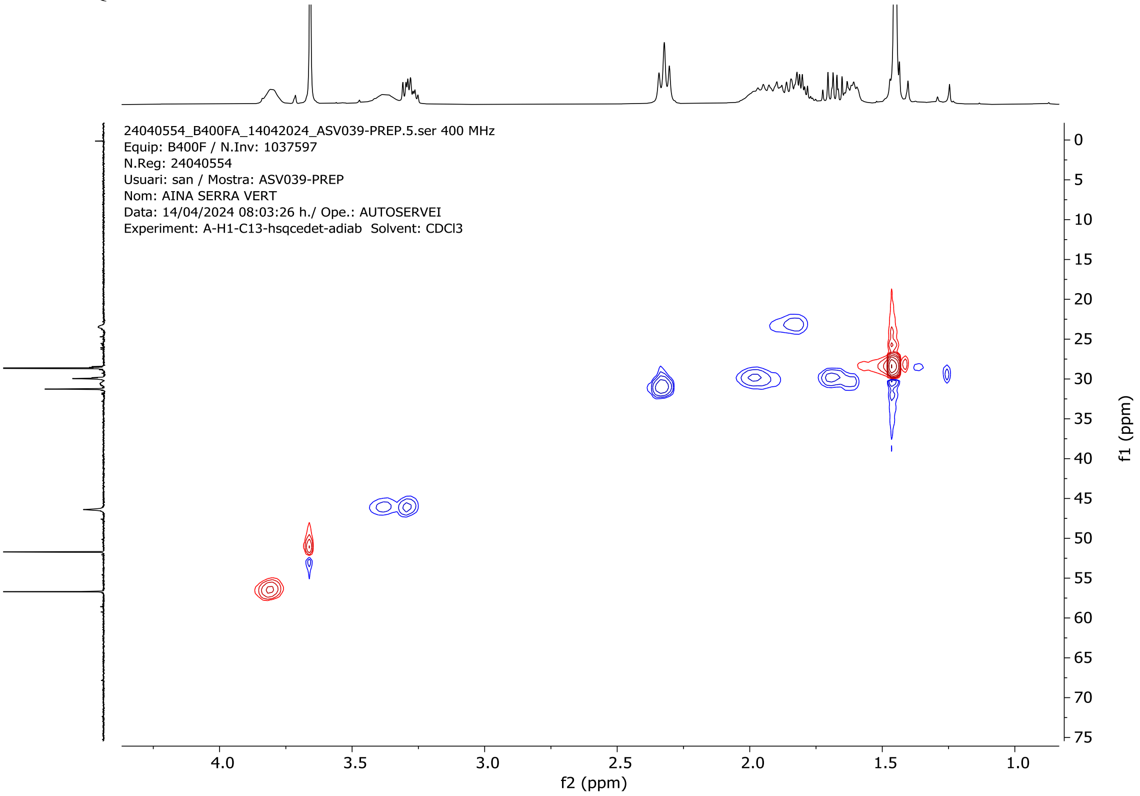

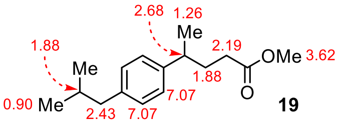

24050321_B400FA_09052024_ASV055-PREP.1.fid 1H 400 MHz
Equip: B400F / N.Inv: 1037597
N.Reg: 24050321
Usuari: san / Mostra: ASV055-PREP
Nom: AINA SERRA VERT
Data: 09/05/2024 13:12:17 h./ Ope.: AUTOSERVEI
Experiment: A-H1-zg30 Solvent: CDCl3

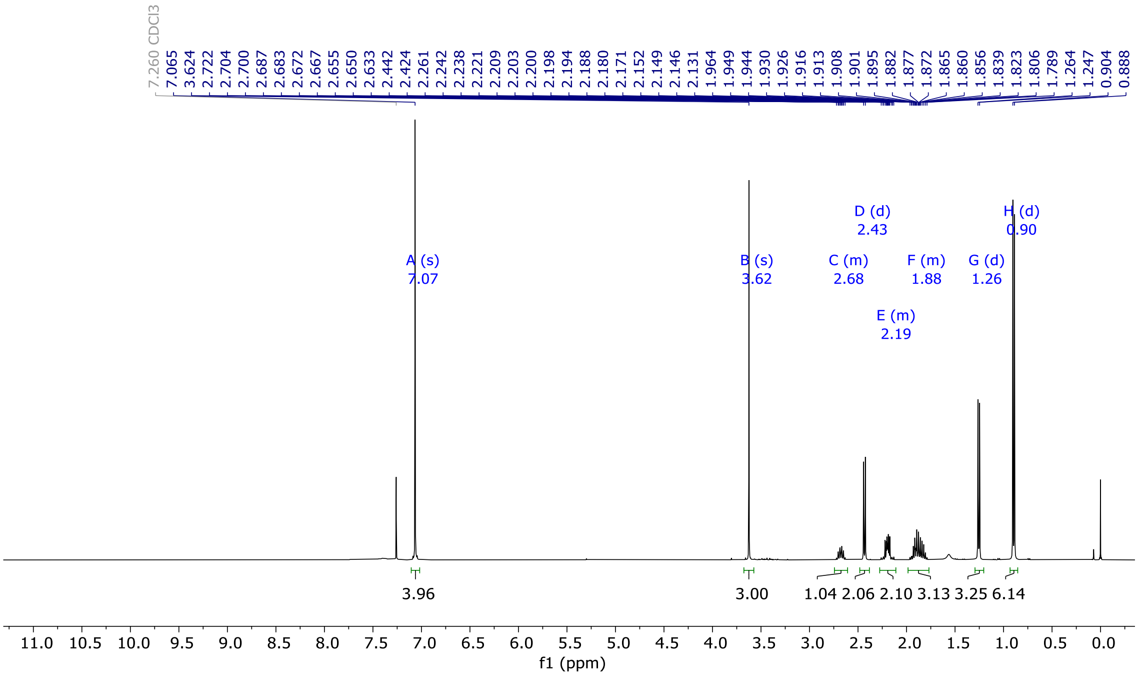

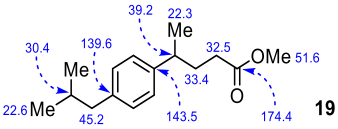

24050321_B400FA_09052024_ASV055-PREP.2.fid 13C{1H} 101 MHz
Equip: B400F / N.Inv: 1037597
N.Reg: 24050321
Usuari: san / Mostra: ASV055-PREP
Nom: AINA SERRA VERT
Data: 09/05/2024 21:58:25 h./ Ope.: AUTOSERVEI
Experiment: A-C13-zgpg30 Solvent: CDCl3

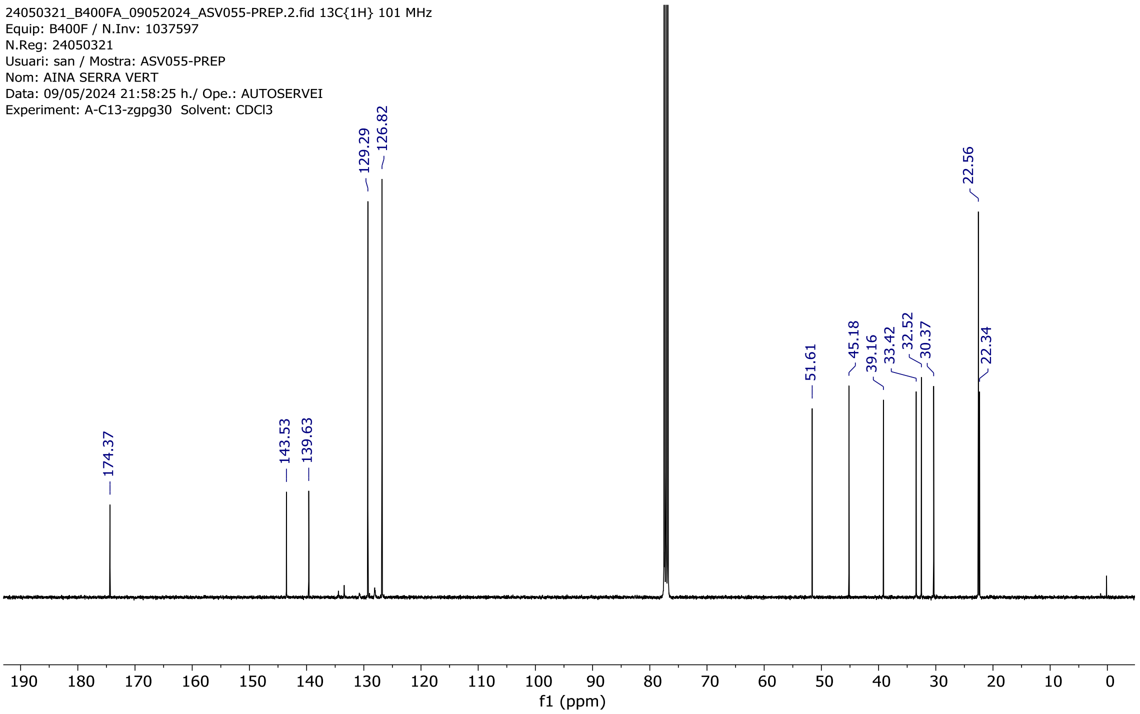

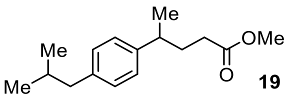

19

2D-COSY

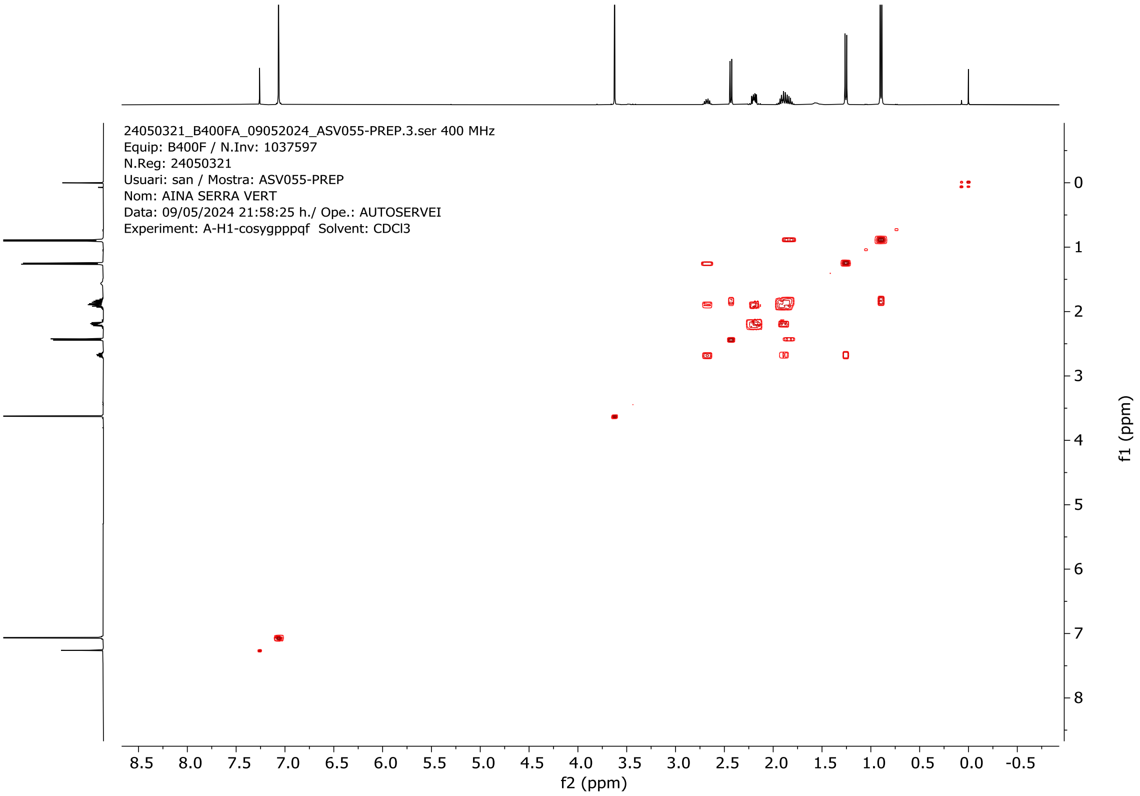

2D-HSQC

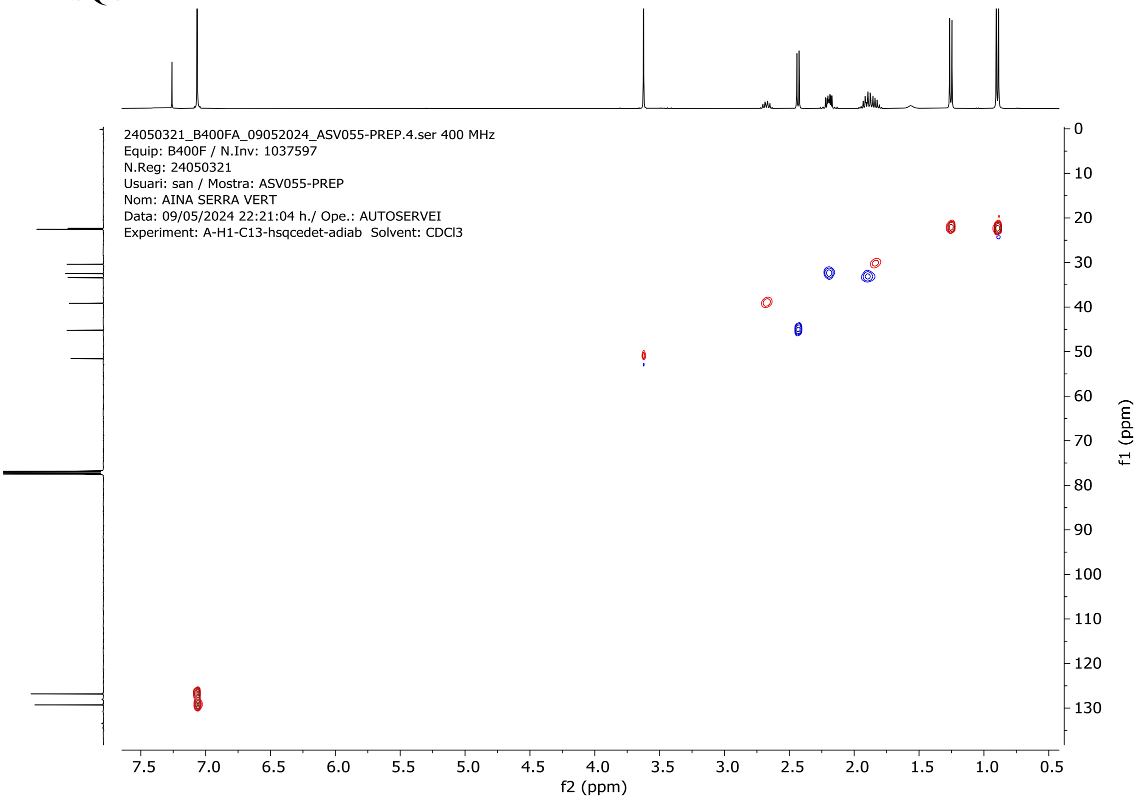

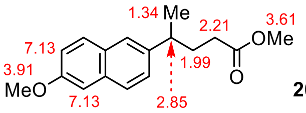

20

24041304_B400FA_30042024_ASV051-17-19.1.fid 1H 400 MHz
Equip: B400F / N.Inv: 1037597
N.Reg: 24041304
Usuari: san / Mostra: ASV051-17-19
Nom: AINA SERRA VERT
Data: 30/04/2024 13:35:23 h./ Ope.: AUTOSERVEI
Experiment: A-H1-zg30 Solvent: CDCl3

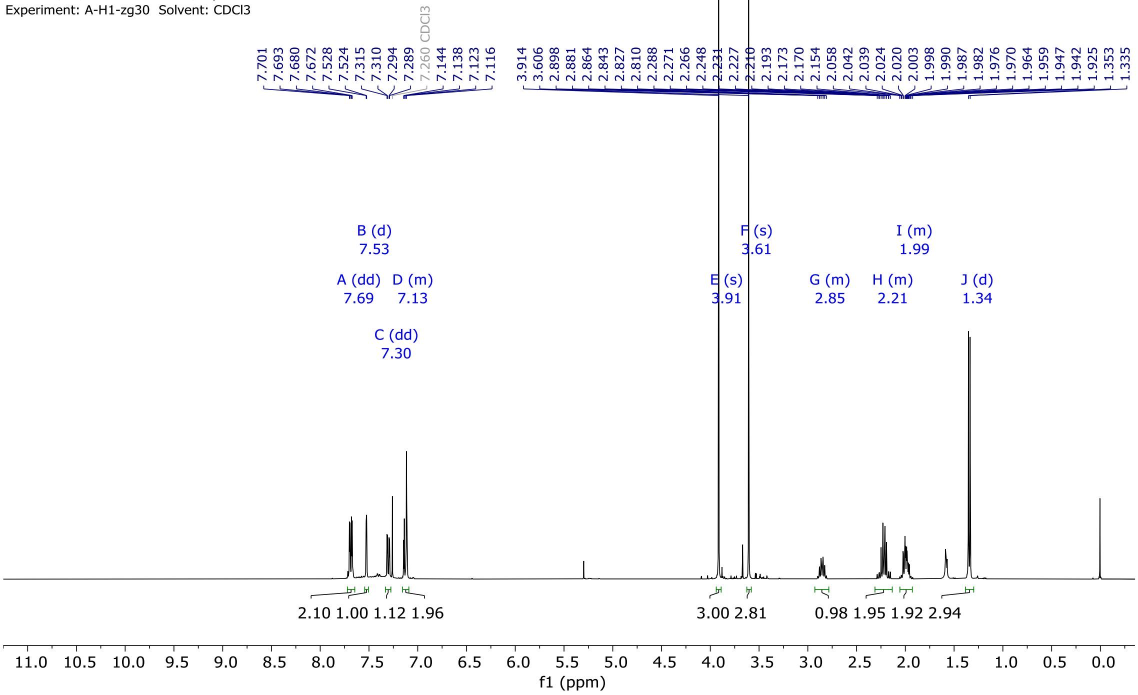

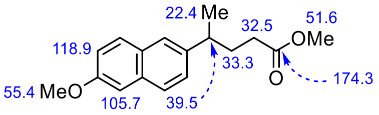

20

24041311_B400FA_01052024_ASV051-17-19-BO.2.fid 13C{1H} 101 MHz
Equip: B400F / N.Inv: 1037597
N.Reg: 24041311
Usuari: san / Mostra: ASV051-17-19-BO
Nom: AINA SERRA VERT
Data: 30/04/2024 21:58:08 h./ Ope.: AUTOSERVEI
Experiment: A-C13-zgpg30 Solvent: CDCl3

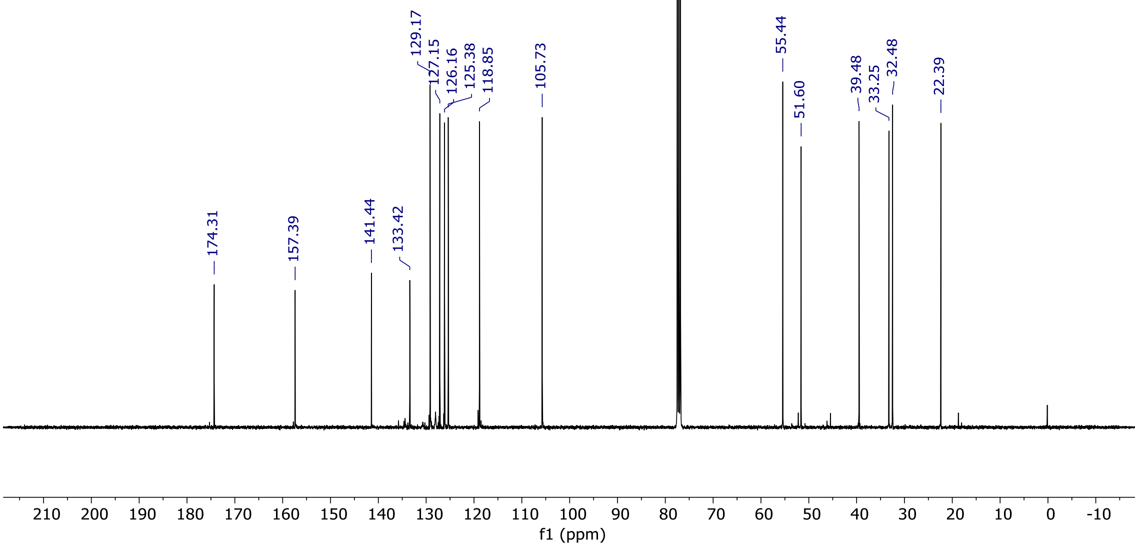

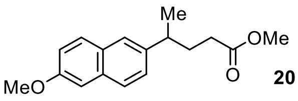

20

2D-COSY

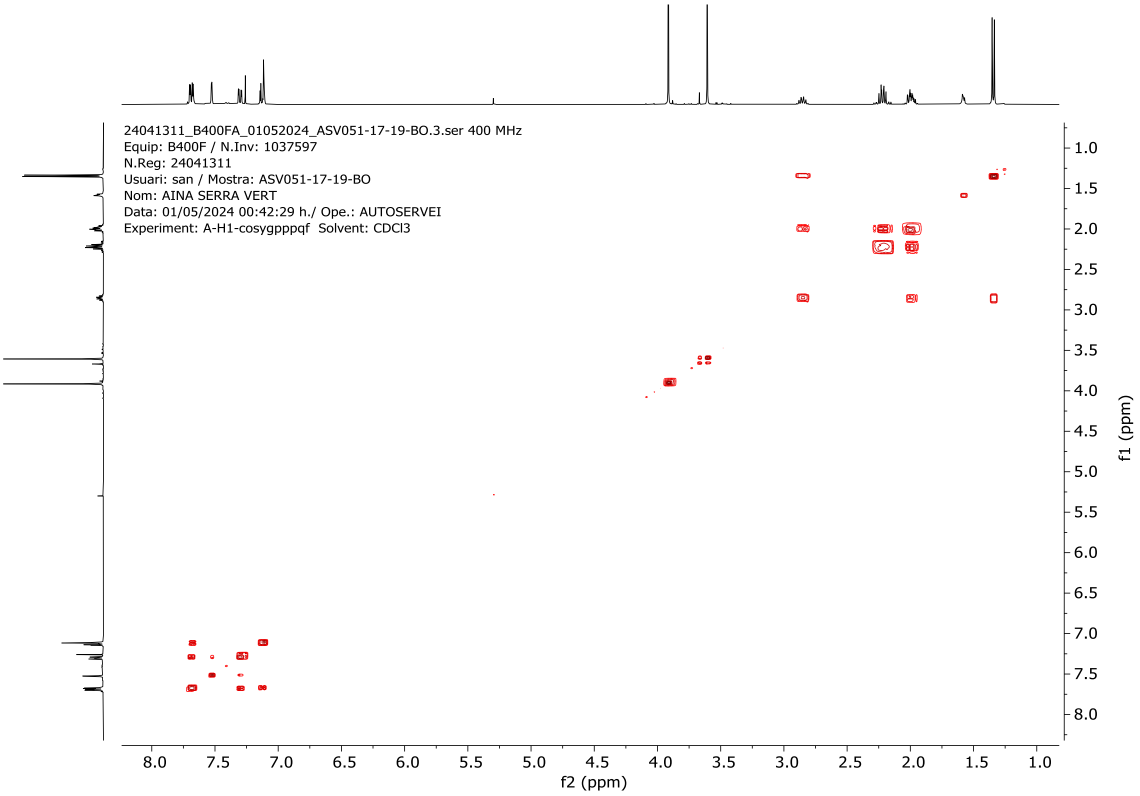

2D-HSQC

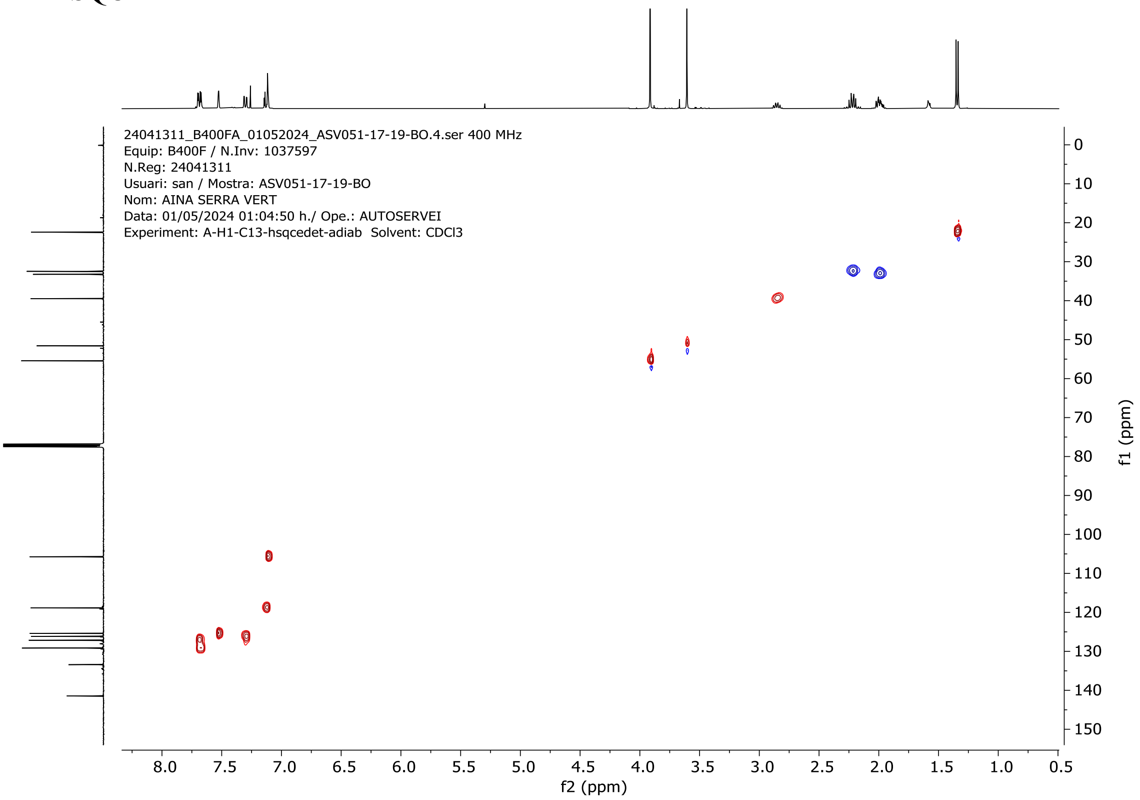

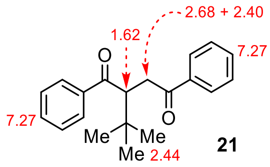

21

24040432_B400FA_11042024_LRG579CH.1.fid 1H 400 MHz
Equip: B400F / N.Inv: 1037597
N.Reg: 24040432
Usuari: san / Mostra: LRG579CH
Nom: LAURA RODRIGUEZ GONZALEZ
Data: 10/04/2024 15:09:50 h./ Ope.: AUTOSERVEI
Experiment: A-H1-zg30 Solvent: CDCl3

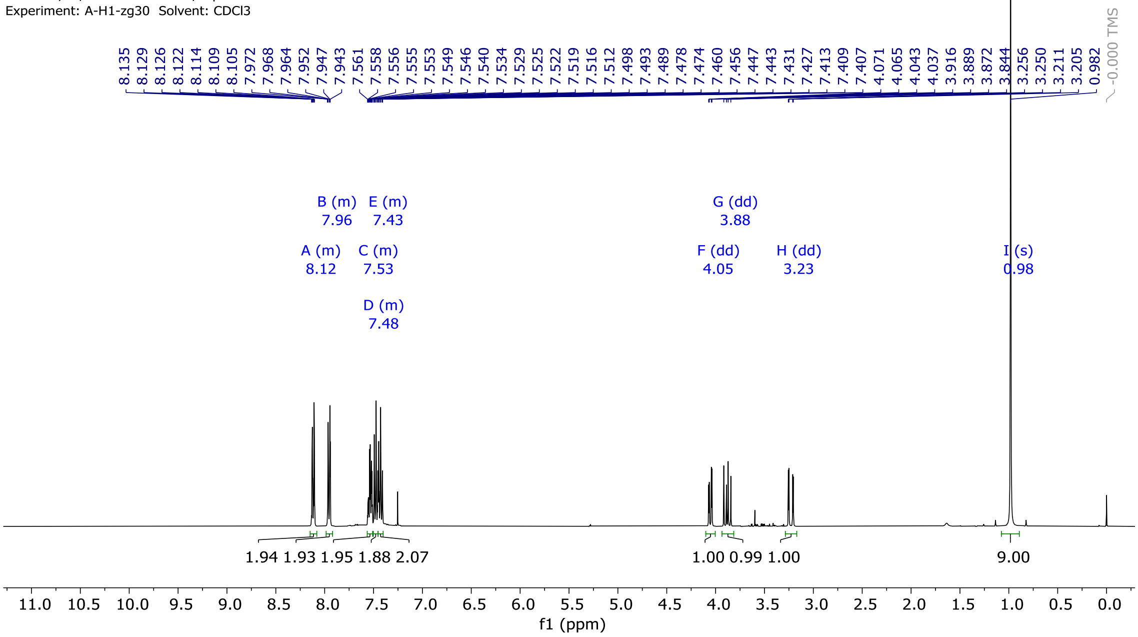

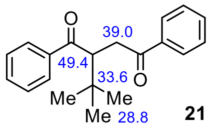

21

24040432_B400FA_11042024_LRG579CH.2.fid 13C{1H} 101 MHz
Equip: B400F / N.Inv: 1037597
N.Reg: 24040432
Usuari: san / Mostra: LRG579CH
Nom: LAURA RODRIGUEZ GONZALEZ
Data: 11/04/2024 06:34:03 h./ Ope.: AUTOSERVEI
Experiment: A-C13-zgpg30 Solvent: CDCl3

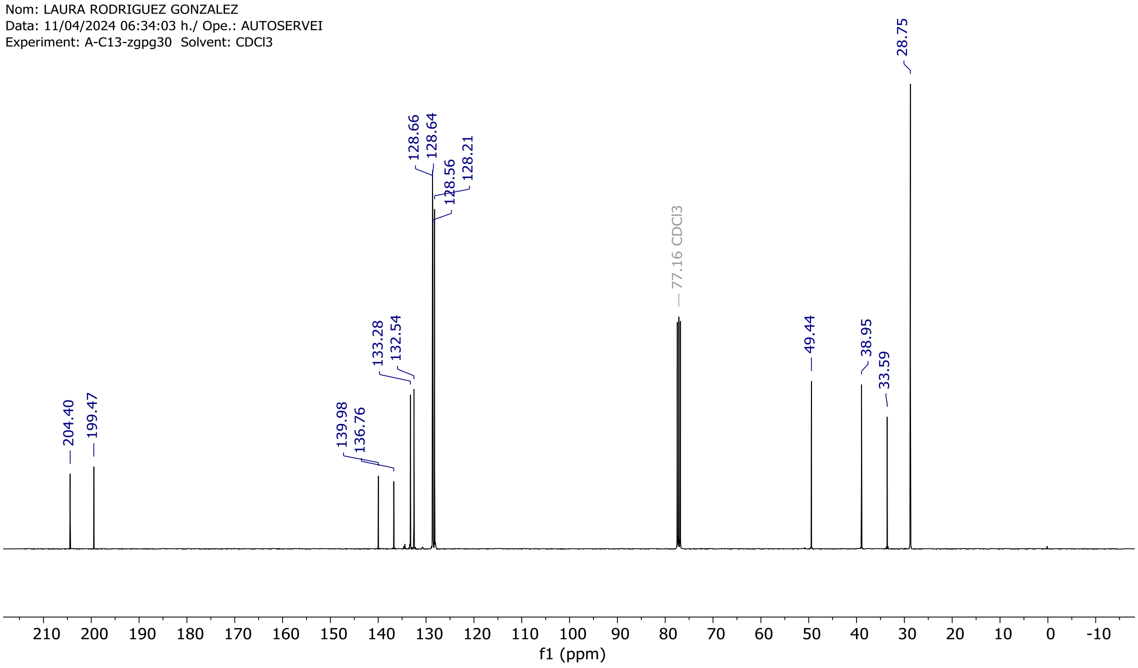

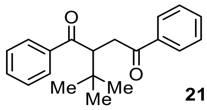

21

2D-COSY

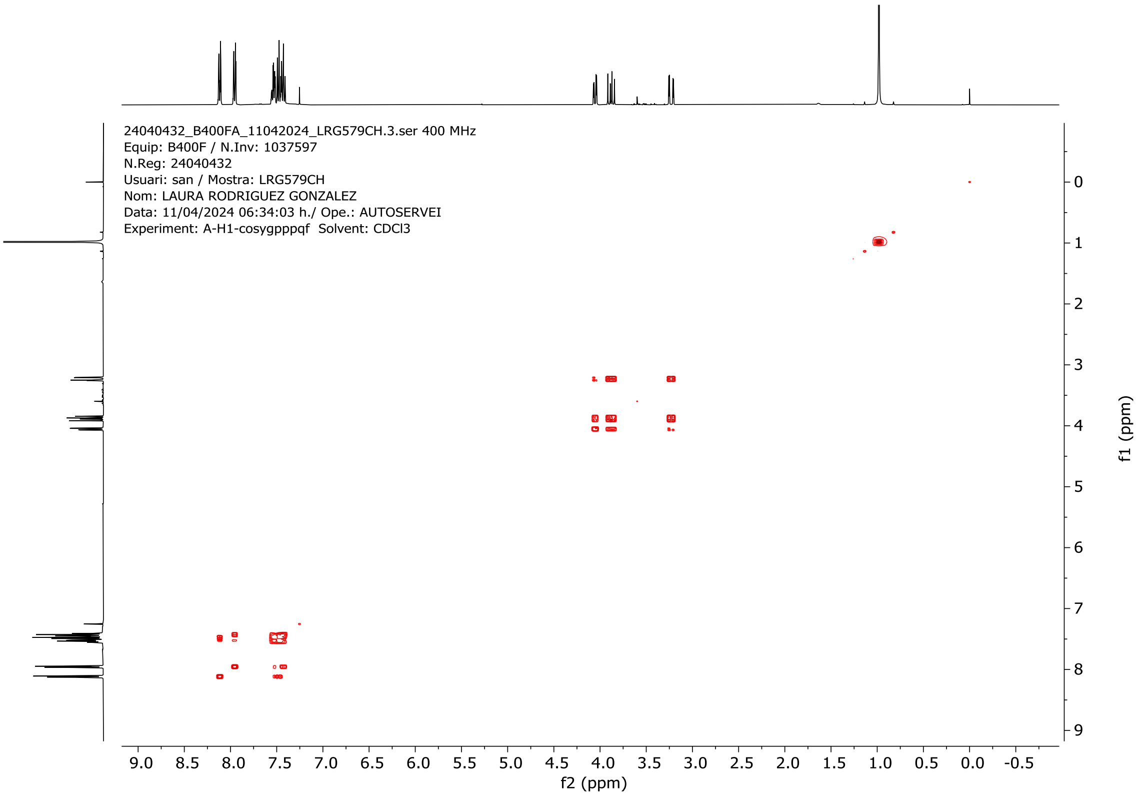

2D-HSQC

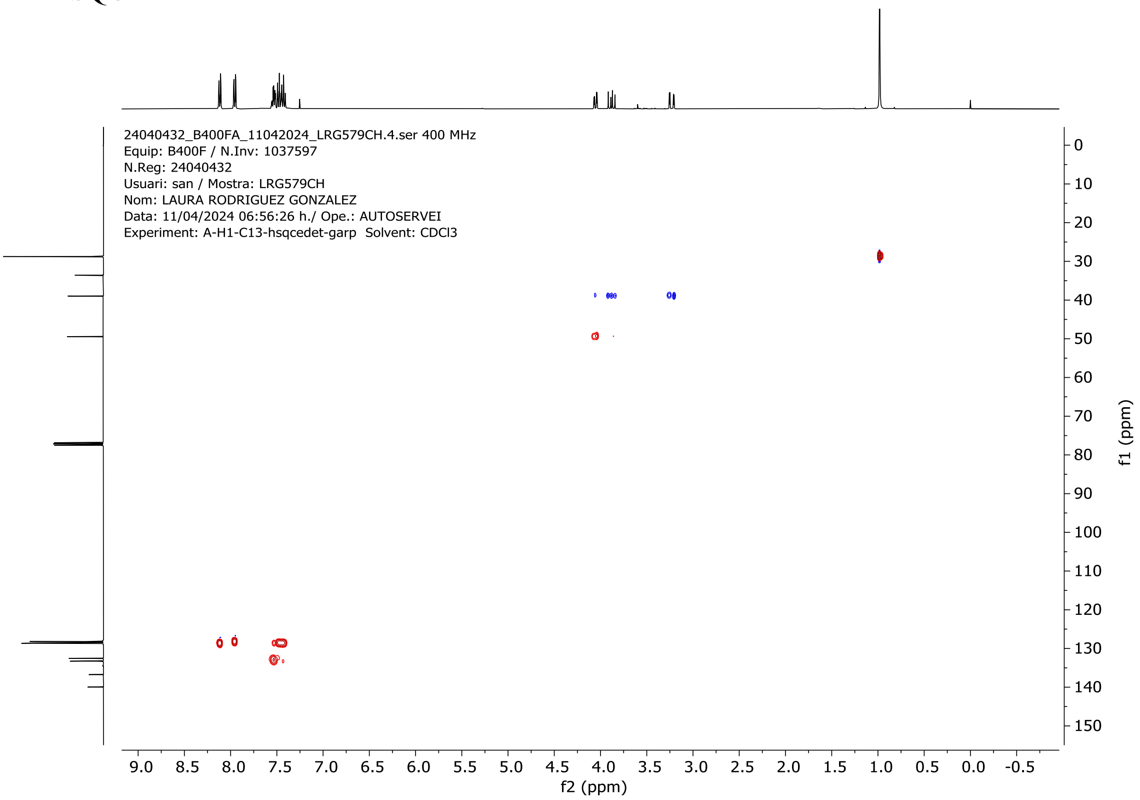

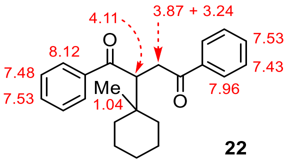

22

auto-17062024-091614.1.fid 1H 400 MHz
Equip: B400Q / N.Inv: 1035091
N.Reg: 24060824
Usuari: san / Mostra: LRG612CH
Nom: LAURA RODRIGUEZ GONZALEZ
Data: 17/06/2024 09:26:03 h./ Ope.: AUTOSERVEI
Experiment: A_1H-zg30 Solvent: CDCl3 Operator:

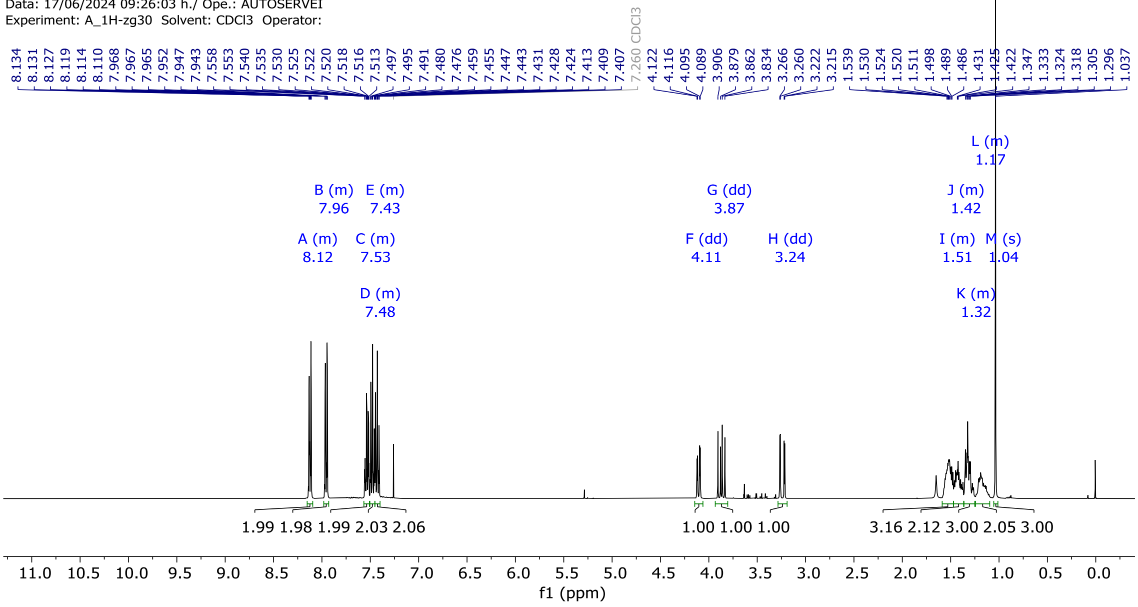

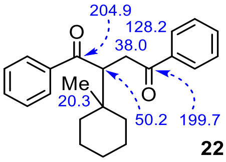

22

auto-17062024-091614.2.fid 13C{1H} 101 MHz
Equip: B400Q / N.Inv: 1035091
N.Reg: 24060824
Usuari: san / Mostra: LRG612CH
Nom: LAURA RODRIGUEZ GONZALEZ
Data: 17/06/2024 21:07:04 h./ Ope.: AUTOSERVEI
Experiment: A_13C-zgpg30 Solvent: CDCl3 Operator:

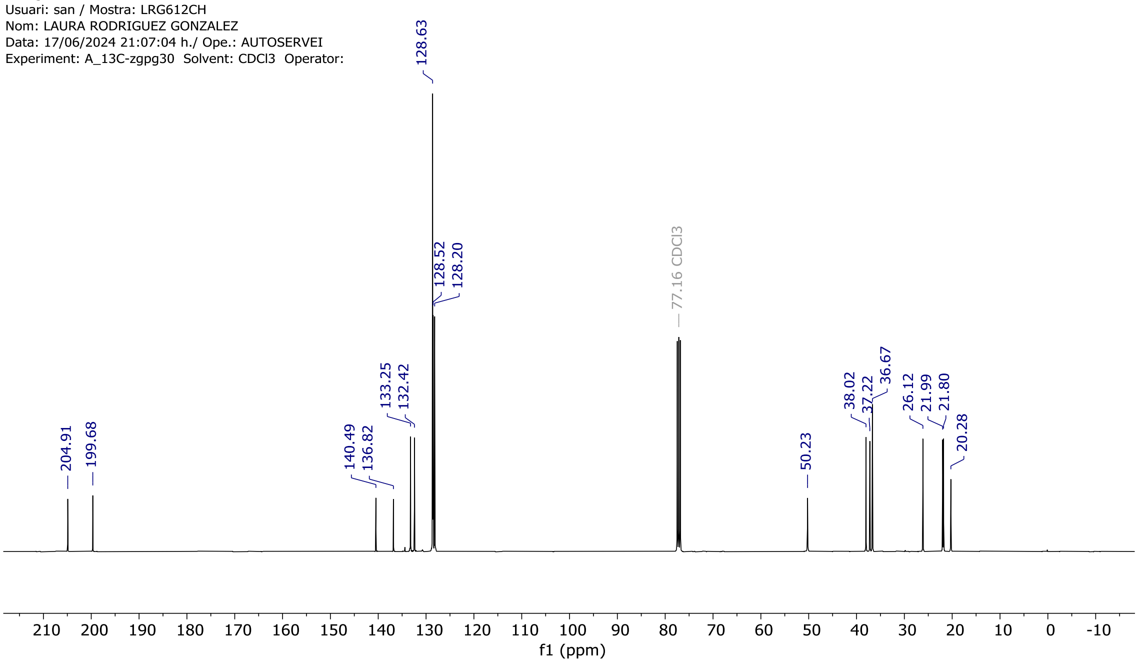

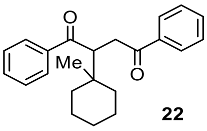

22

2D-COSY

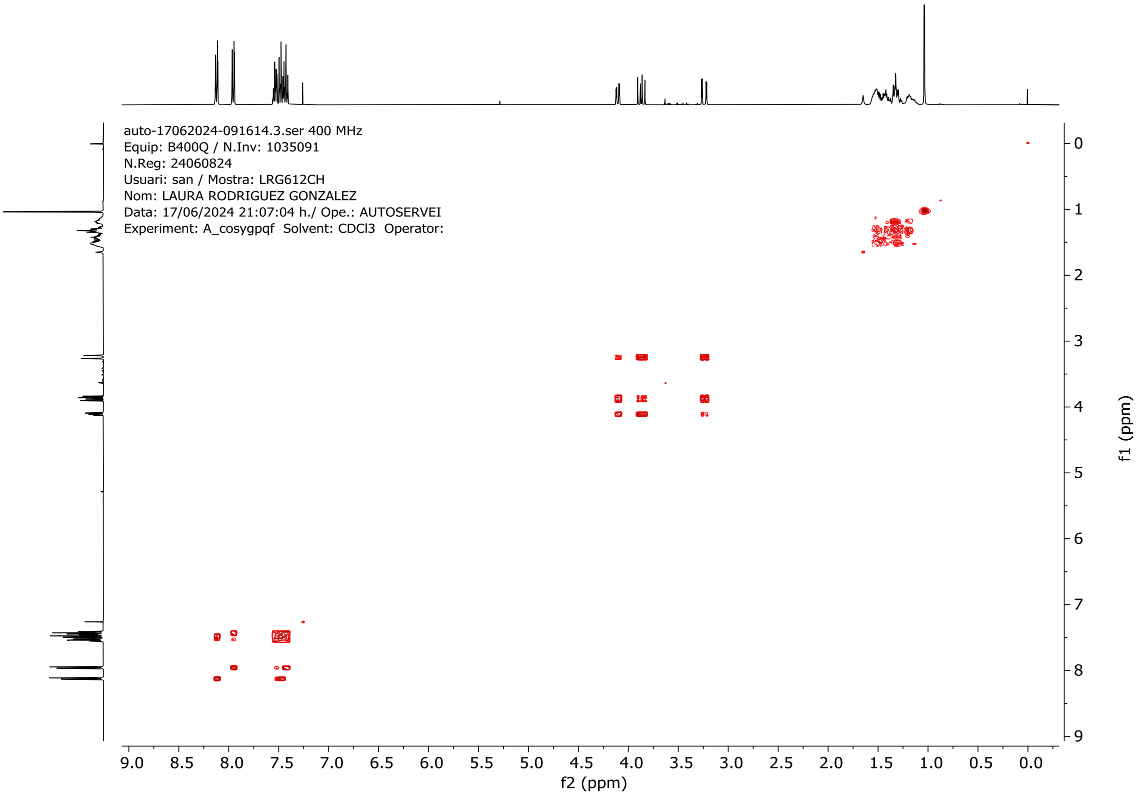

2D-HSQC

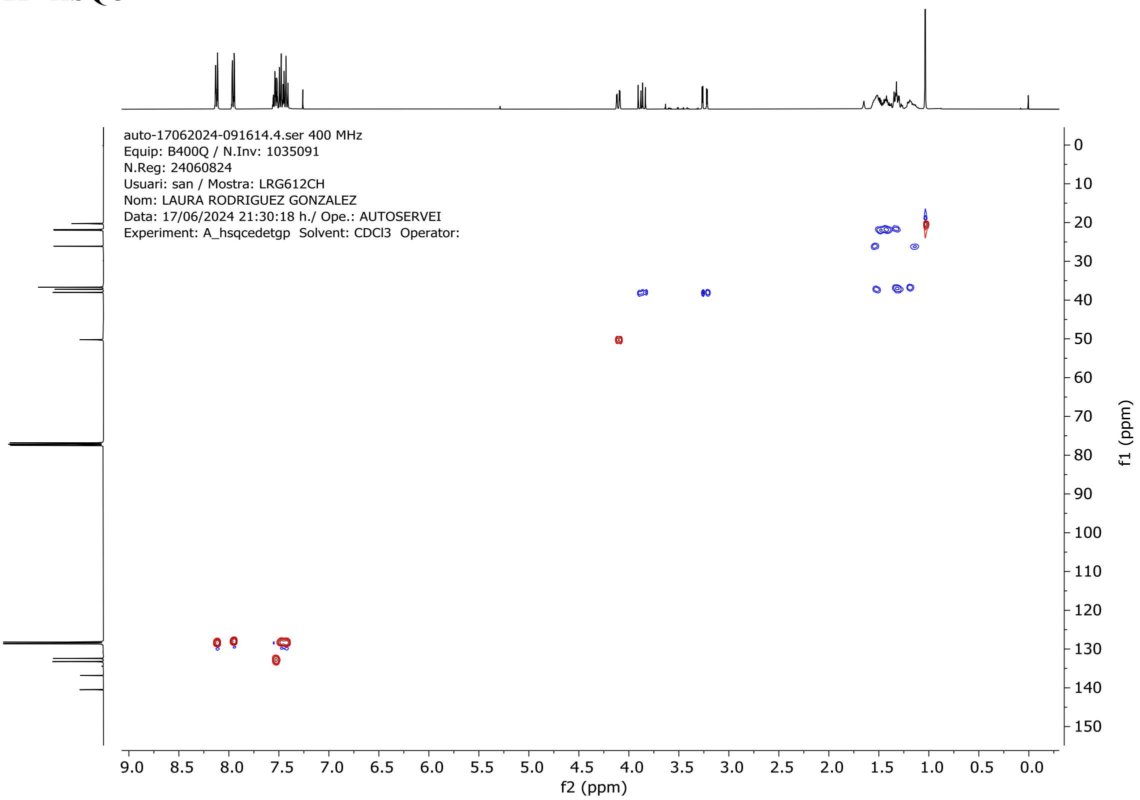

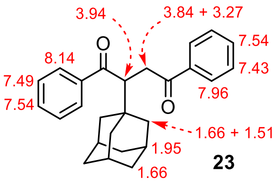

23

24041200_B400FA_26042024_ASV049-PREP-1.1.fid 1H 400 MHz
Equip: B400F / N.Inv: 1037597
N.Reg: 24041200
Usuari: san / Mostra: ASV049-PREP-1
Nom: AINA SERRA VERT
Data: 26/04/2024 13:25:03 h./ Ope.: AUTOSERVEI
Experiment: A-H1-zg30 Solvent: CDCl3

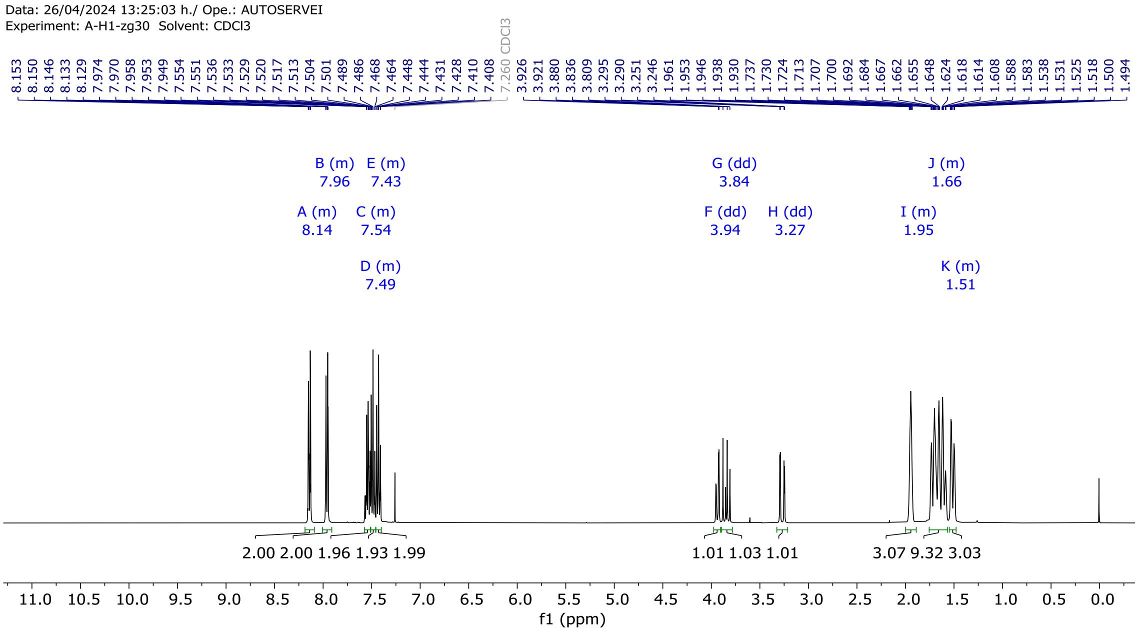

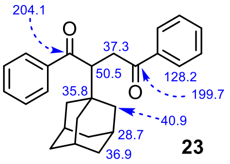

23

24041218_B400FA_27042024_ASV049PREP1CH.2.fid 13C{1H} 101 MHz
Equip: B400F / N.Inv: 1037597
N.Reg: 24041218
Usuari: san / Mostra: ASV049PREP1CH
Nom: LAURA RODRIGUEZ GONZALEZ
Data: 27/04/2024 03:53:22 h./ Ope.: AUTOSERVEI
Experiment: A-C13-zgpg30 Solvent: CDCl3

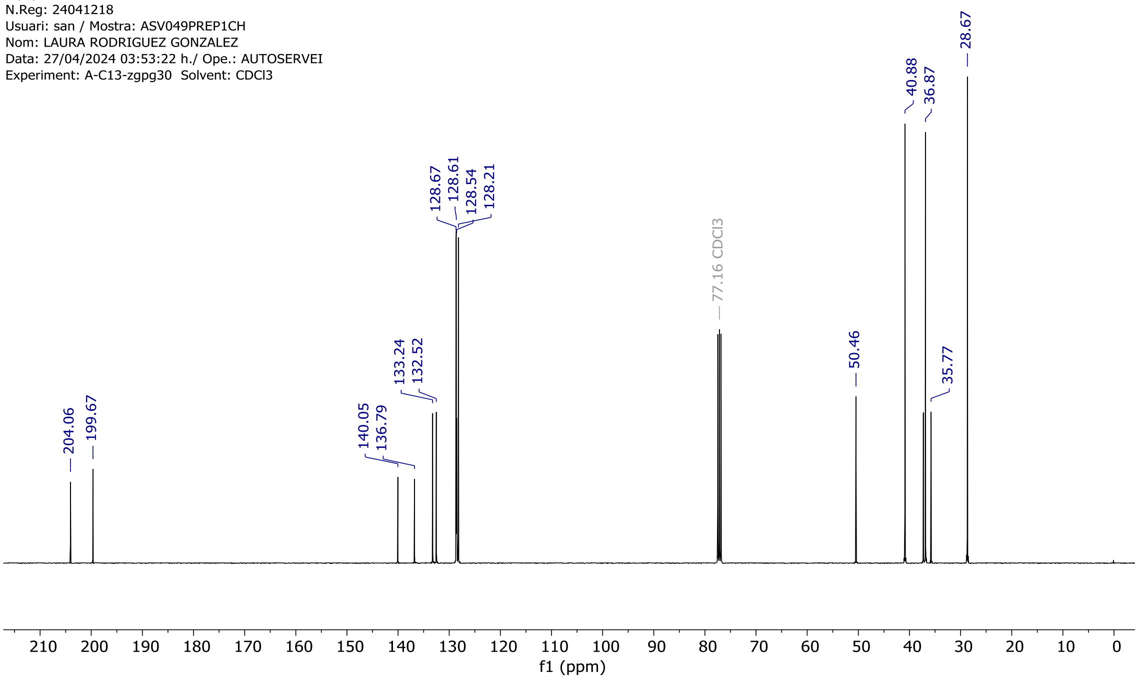

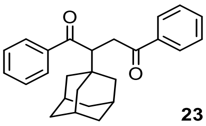

23

2D-COSY

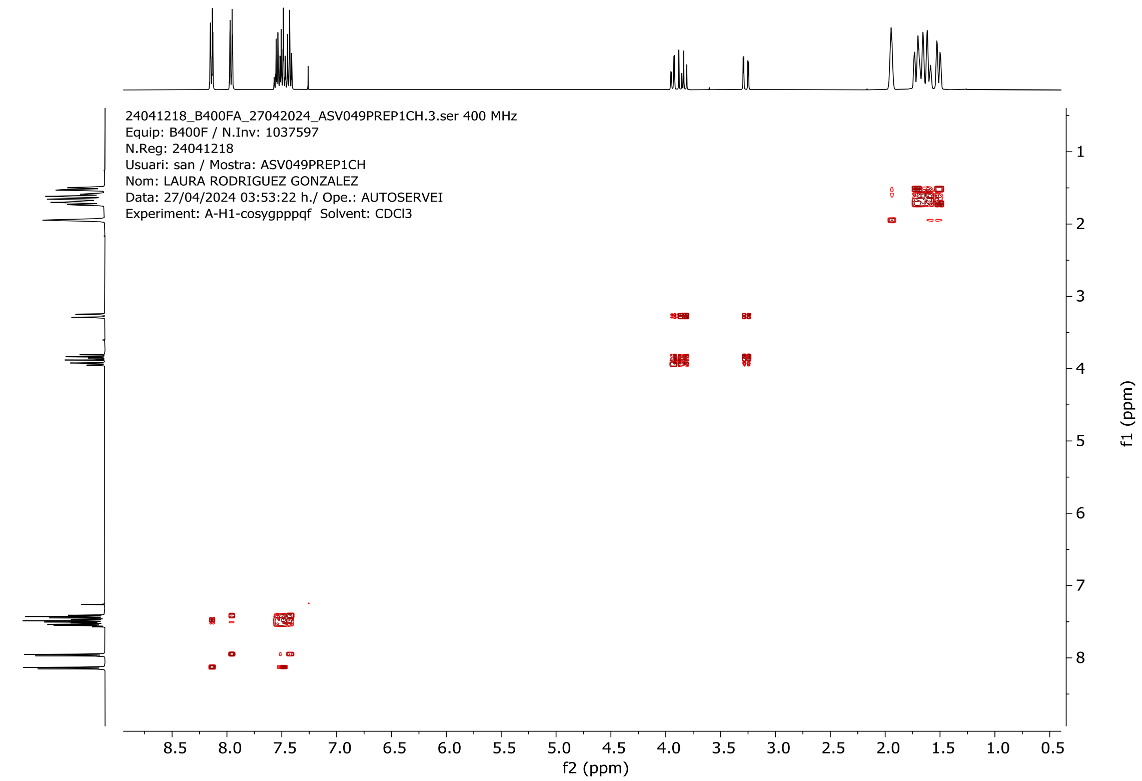

2D-HSQC

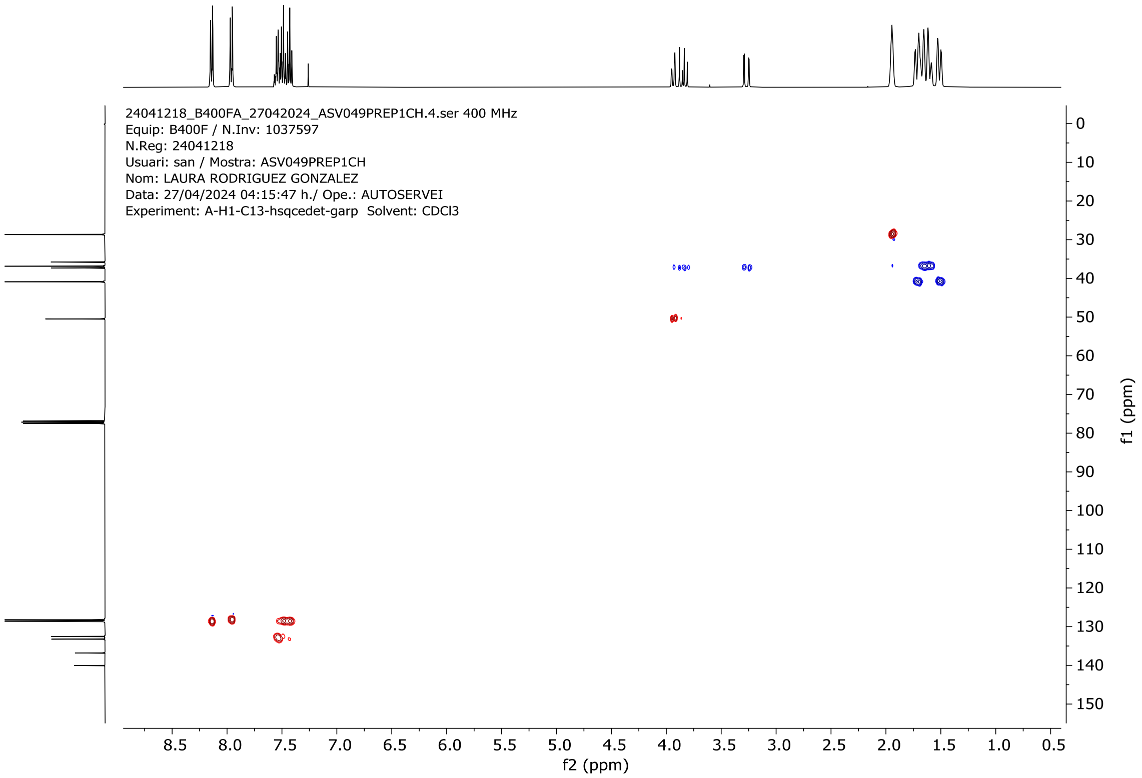

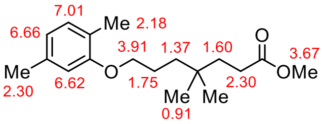

24

24050271_B400FA_08052024_ASV053-PREP1.1.fid 1H 400 MHz
Equip: B400F / N.Inv: 1037597
N.Reg: 24050271
Usuari: san / Mostra: ASV053-PREP1
Nom: AINA SERRA VERT
Data: 08/05/2024 15:33:28 h./ Ope.: AUTOSERVEI
Experiment: A-H1-zg30 Solvent: CDCl3

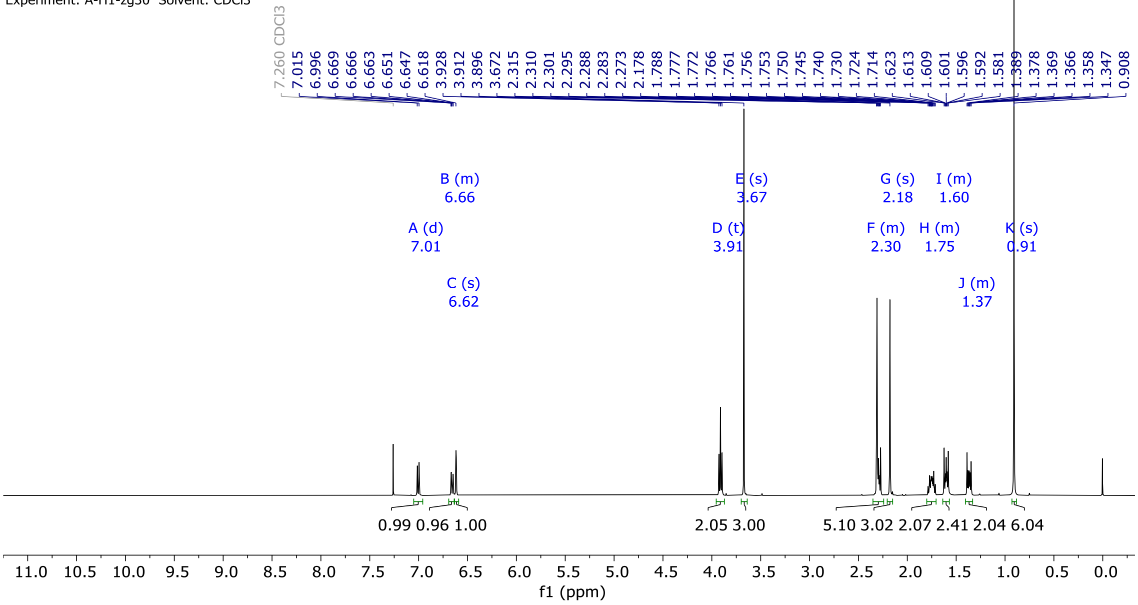

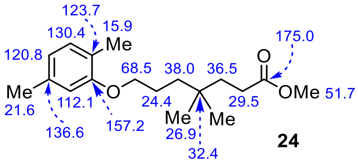

24

24050271_B400FA_08052024_ASV053-PREP1.2.fid 13C{1H} 101 MHz
Equip: B400F / N.Inv: 1037597
N.Reg: 24050271
Usuari: san / Mostra: ASV053-PREP1
Nom: AINA SERRA VERT
Data: 08/05/2024 21:08:53 h./ Ope.: AUTOSERVEI
Experiment: A-C13-zgpg30 Solvent: CDCl3

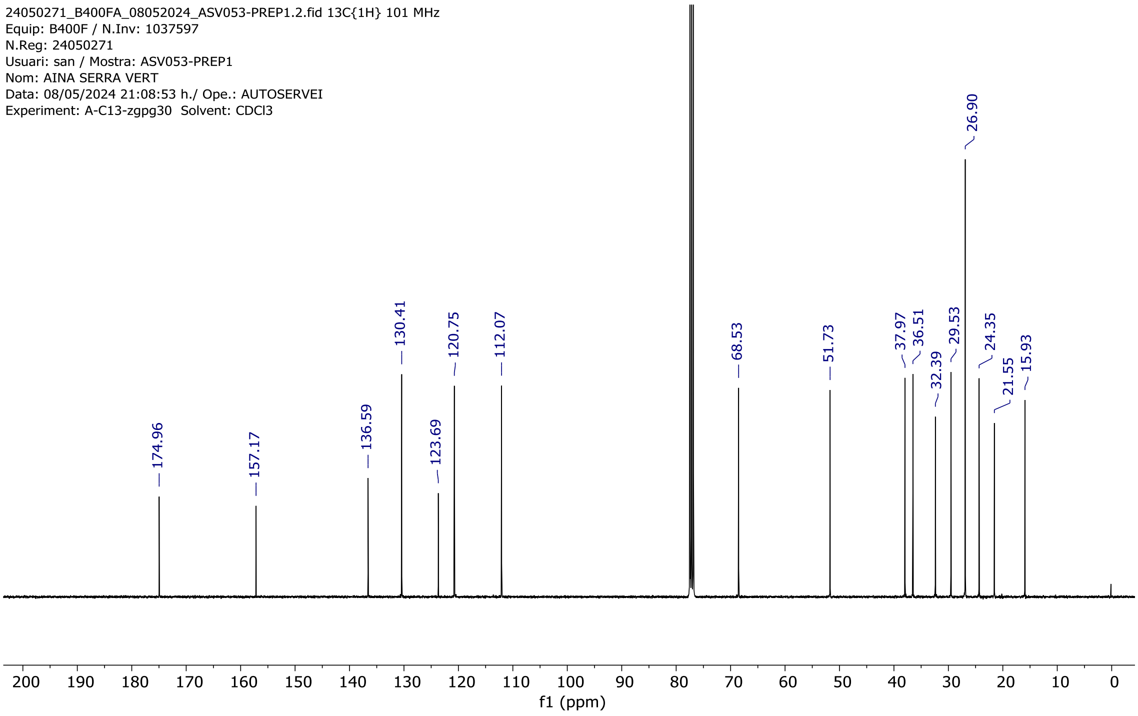

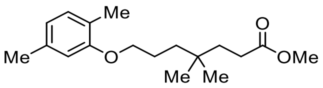

24

2D-COSY

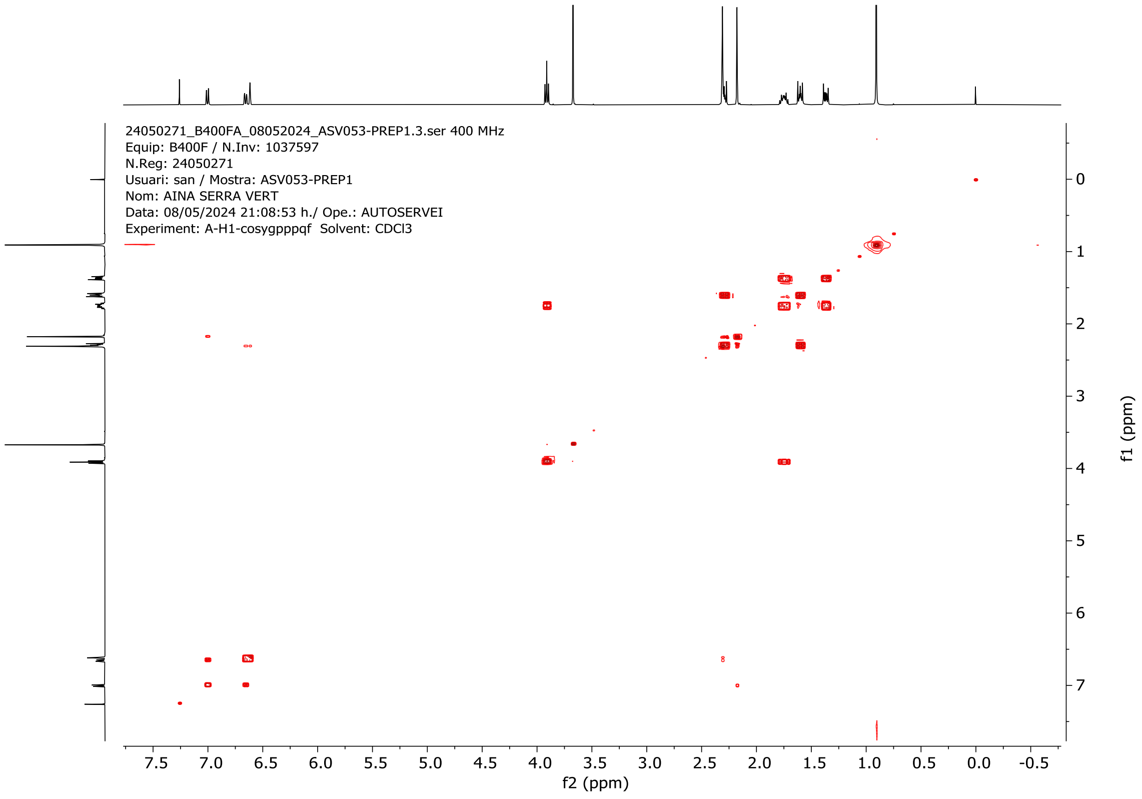

2D-HSQC

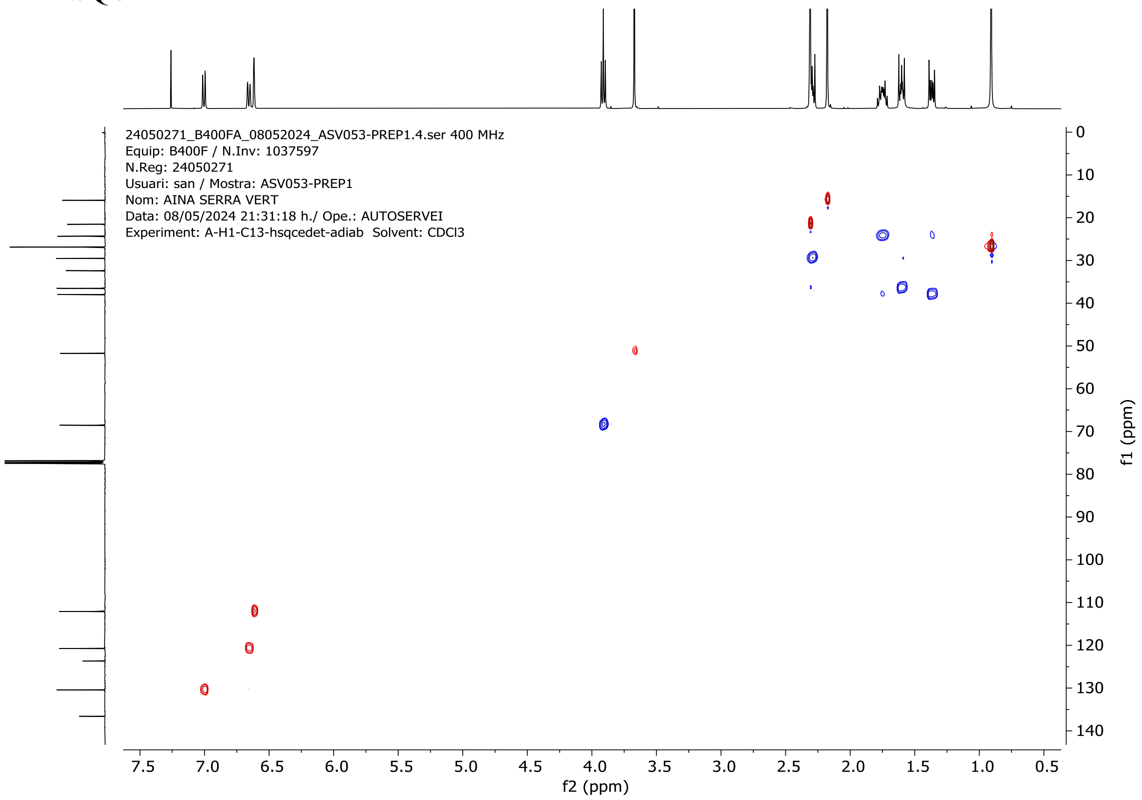

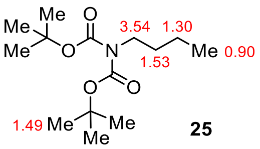

25

24020514_B400FA_17022024_LRG542COLT11T13.1.fid 1H 400 MHz
Equip: B400F / N.Inv: 1037597
N.Reg: 24020514
Usuari: san / Mostra: LRG542COLT11T13
Nom: LAURA RODRIGUEZ GONZALEZ
Data: 16/02/2024 15:15:24 h./ Ope.: AUTOSERVEI
Experiment: A-H1-zg30 Solvent: CDCl3

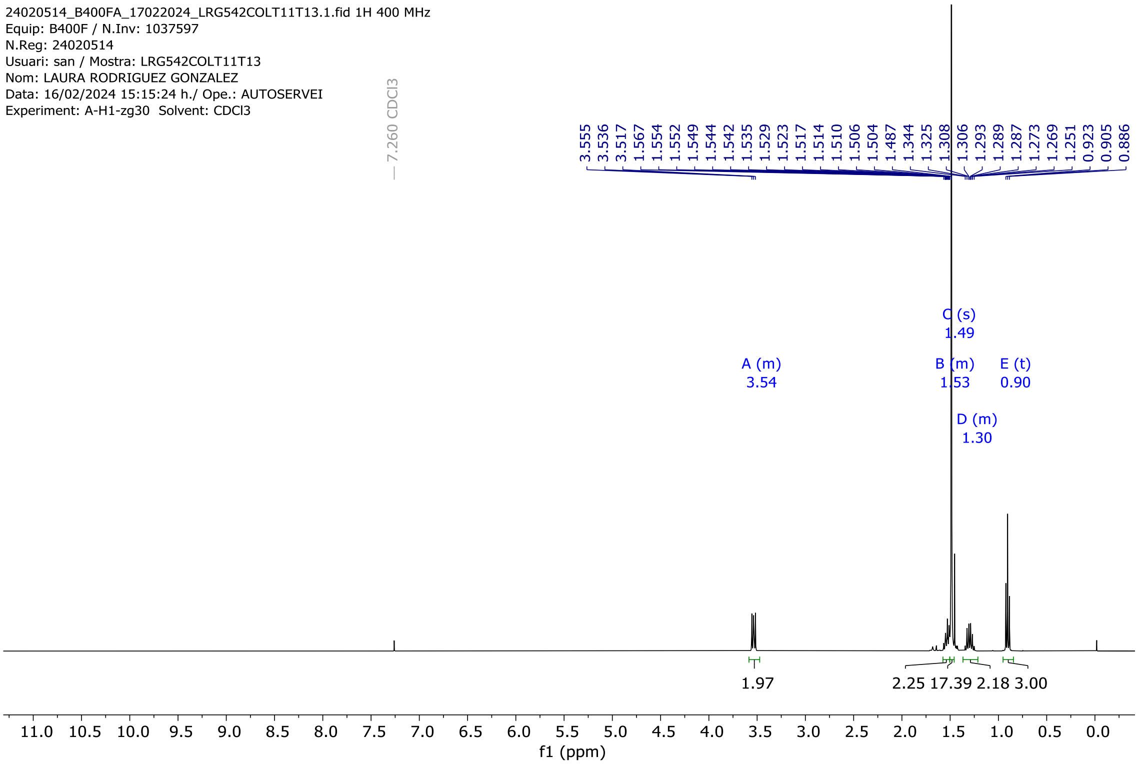

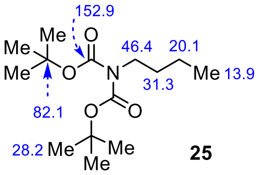

25

24020514_B400FA_17022024_LRG542COLT11T13.2.fid 13C{1H} 101 MHz
Equip: B400F / N.Inv: 1037597
N.Reg: 24020514
Usuari: san / Mostra: LRG542COLT11T13
Nom: LAURA RODRIGUEZ GONZALEZ
Data: 17/02/2024 06:46:03 h./ Ope.: AUTOSERVEI
Experiment: A-C13-zgpg30 Solvent: CDCl3

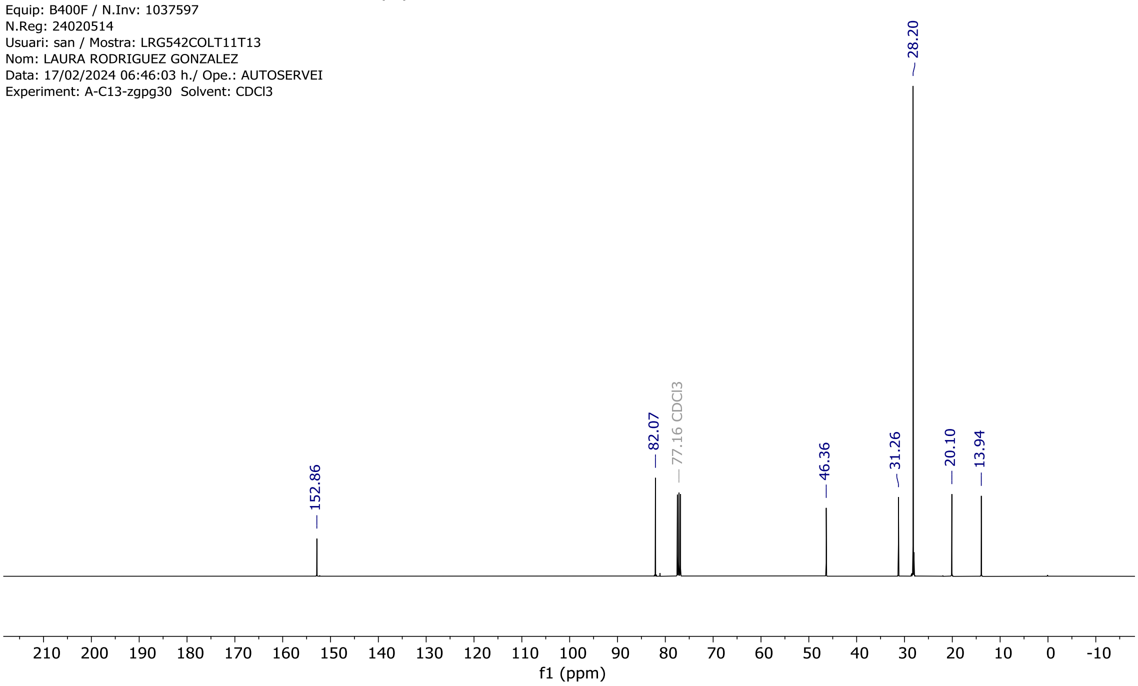

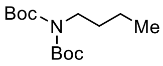

25

2D-COSY

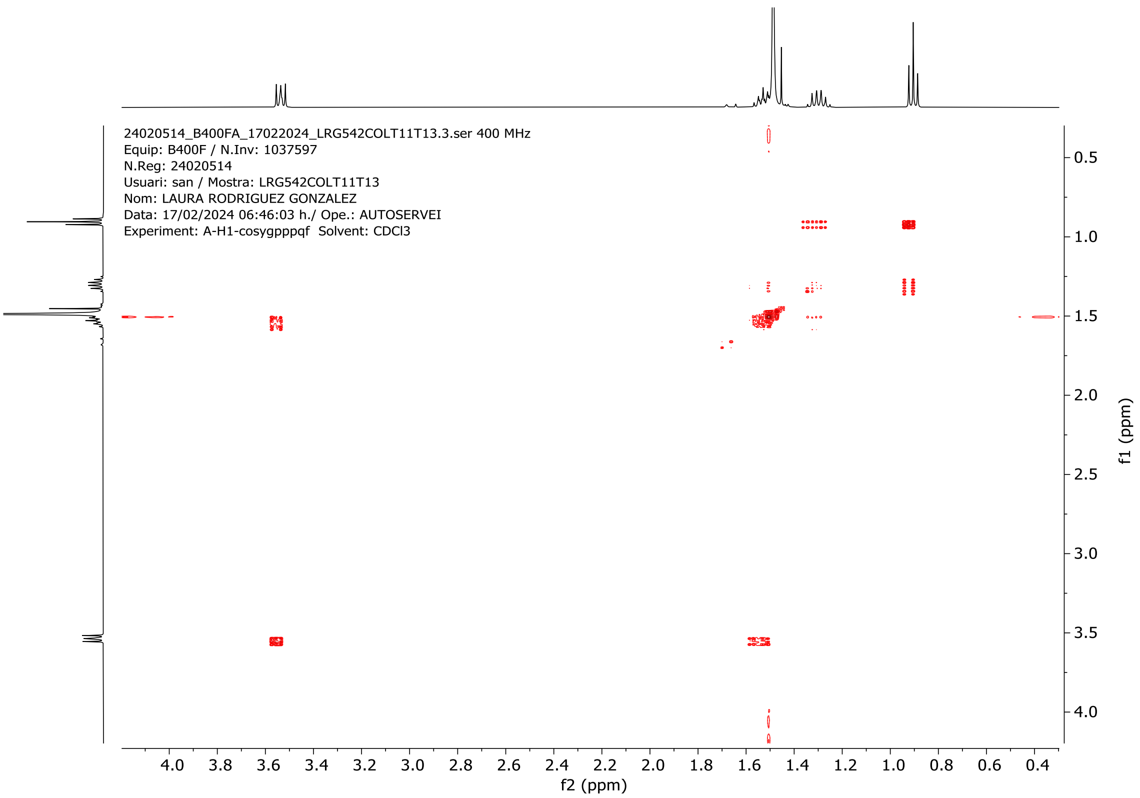

2D-HSQC

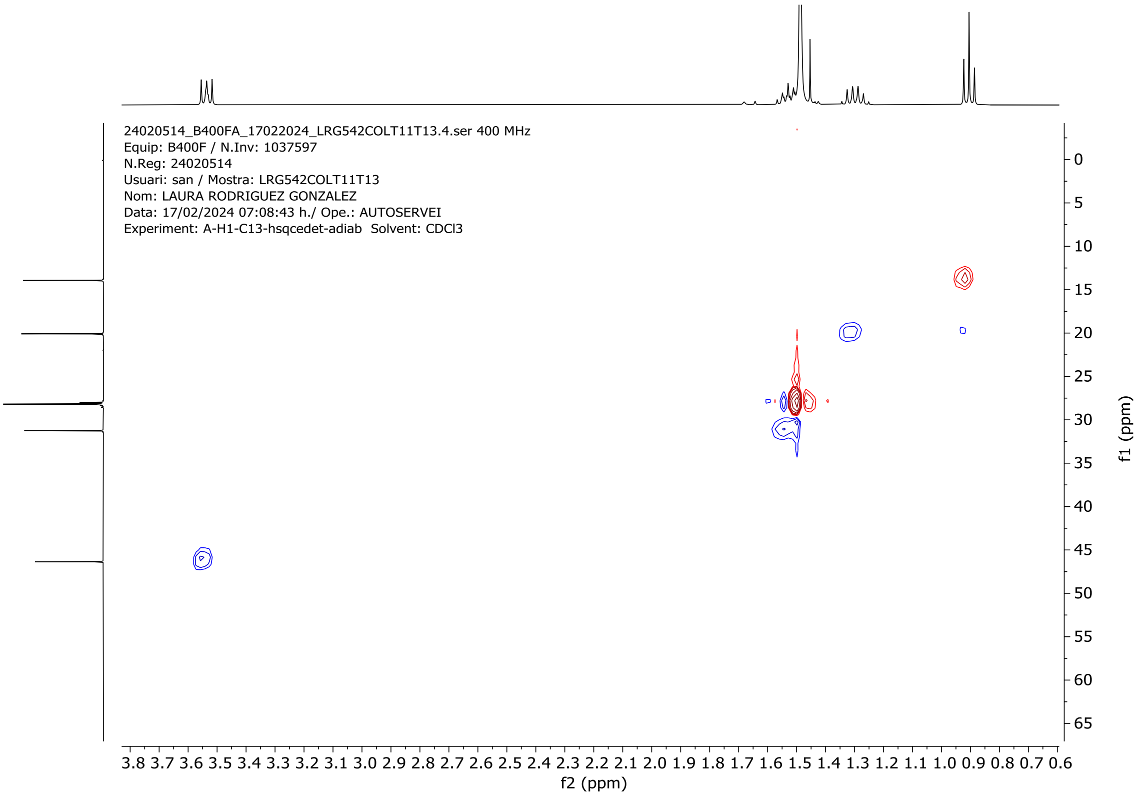

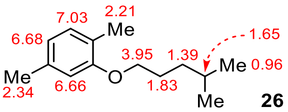

26

auto-27062024-123559.1.fid 1H 400 MHz
Equip: B400Q / N.Inv: 1035091
N.Reg: 24061192
Usuari: san / Mostra: LRG617COLT10T15
Nom: LAURA RODRIGUEZ GONZALEZ
Data: 27/06/2024 12:42:11 h./ Ope.: AUTOSERVEI
Experiment: A_1H-zg30 Solvent: CDCl3 Operator:

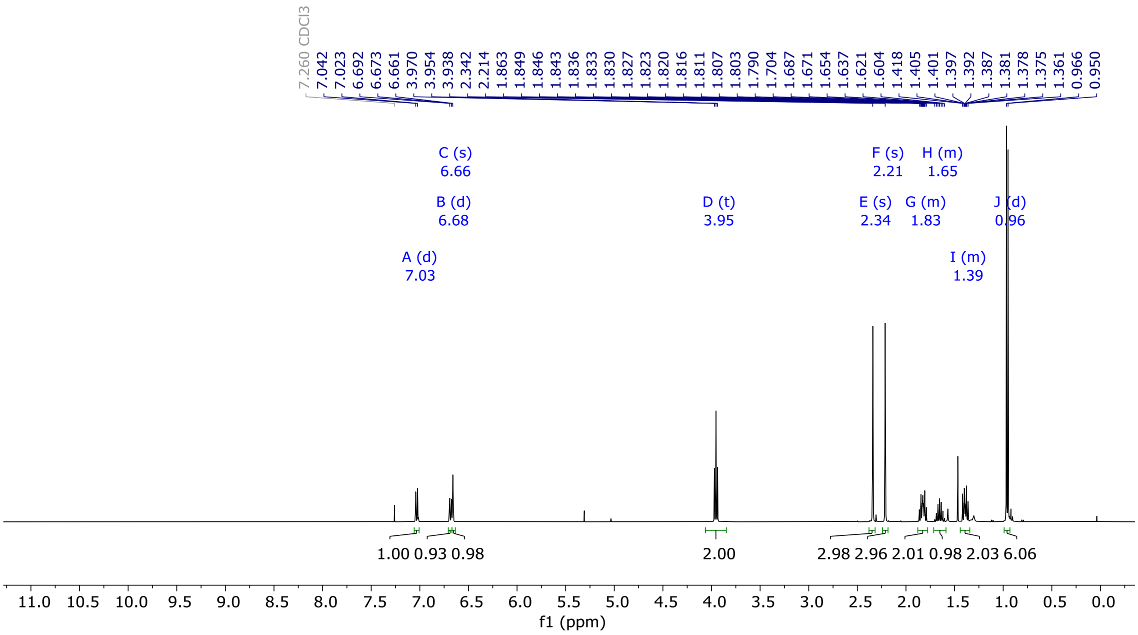

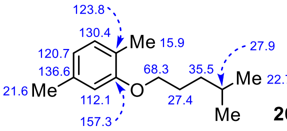

26

auto-27062024-130906.2.fid 13C{1H} 101 MHz
Equip: B400Q / N.Inv: 1035091
N.Reg: 24061194
Usuari: san / Mostra: LRG617CH
Nom: LAURA RODRIGUEZ GONZALEZ
Data: 27/06/2024 23:08:43 h./ Ope.: AUTOSERVEI
Experiment: A_13C-zgpg30 Solvent: CDCl3 Operator:

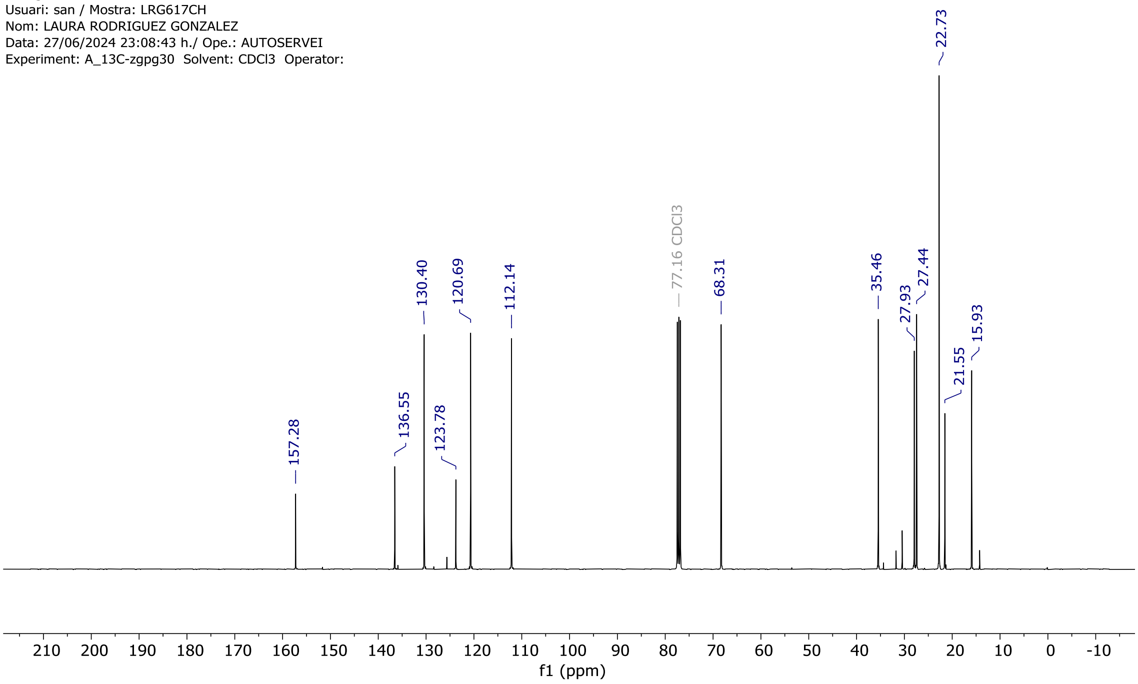

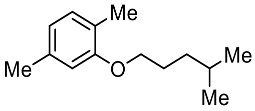

26

2D-COSY

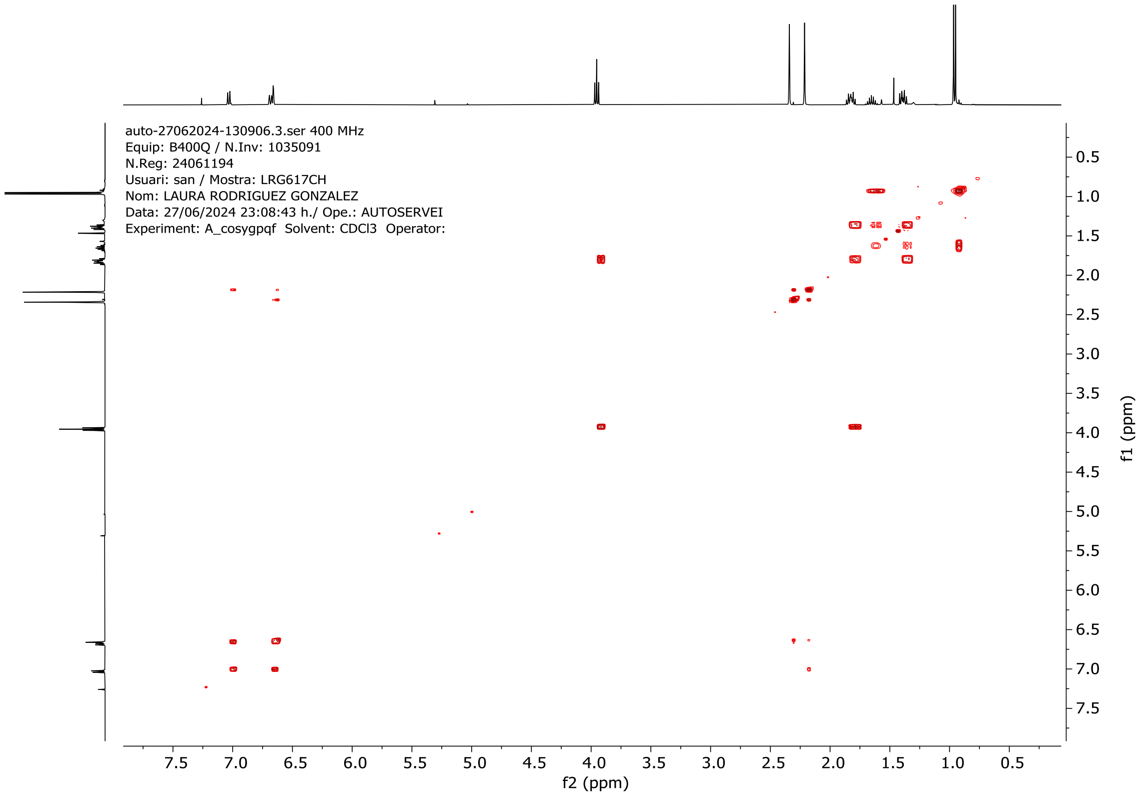

2D-HSQC

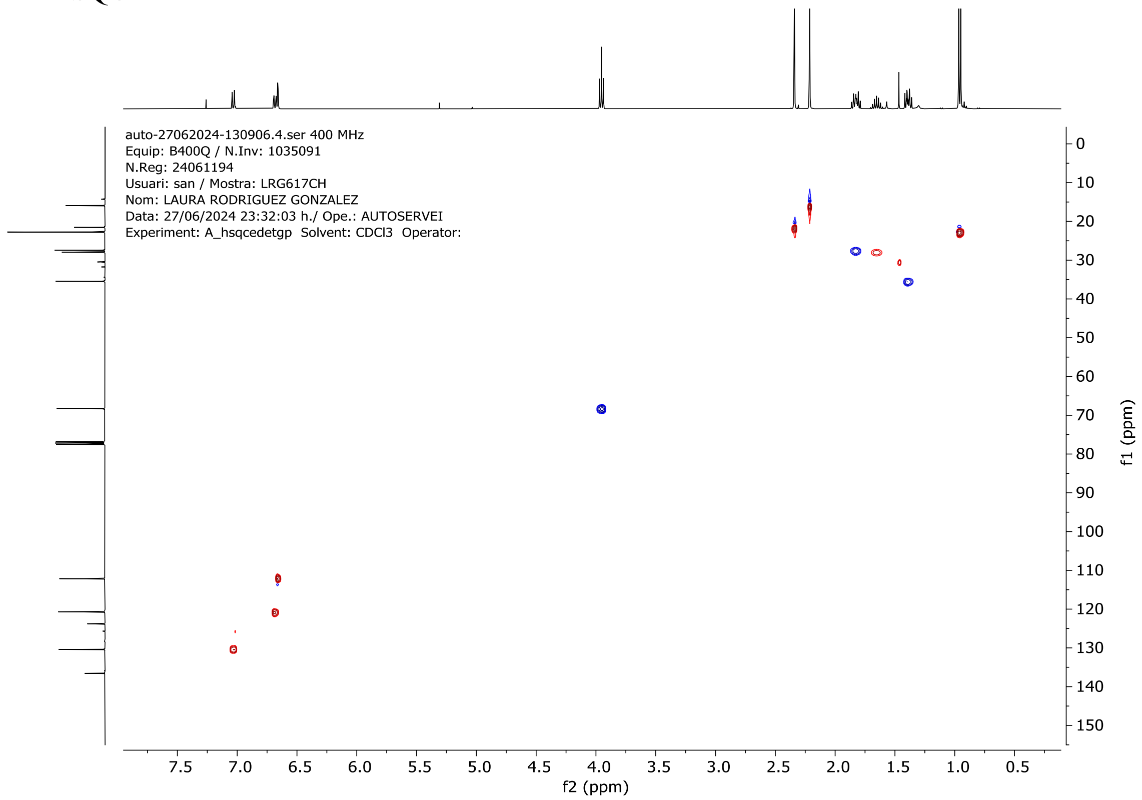

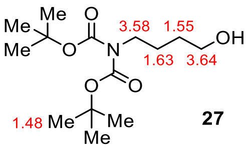
Me
Me
Me
O
O
N
OH
O
O
Me
Me
Me
3.58 1.55
1.63 3.64
1.48

27

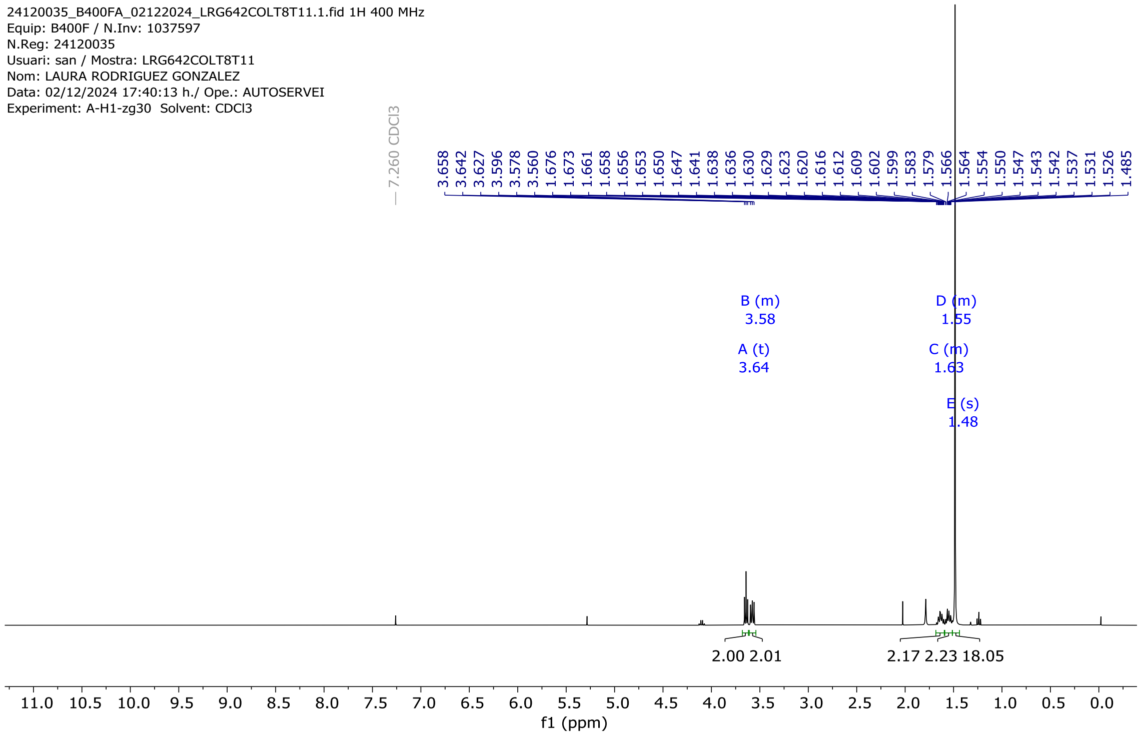
24120035_B400FA_02122024_LRG642COLT8T11.1.fid 1H 400 MHz
Equip: B400F / N.Inv: 1037597
N.Reg: 24120035
Usuari: san / Mostra: LRG642COLT8T11
Nom: LAURA RODRIGUEZ GONZALEZ
Data: 02/12/2024 17:40:13 h./ Ope.: AUTOSERVEI
Experiment: A-H1-zg30 Solvent: CDCl3
7.260 CDCl3
3.658 3.642 3.627 3.596 3.578 3.560 1.676 1.673 1.661 1.658 1.656 1.653 1.650 1.647 1.641 1.638 1.636 1.630 1.629 1.623 1.620 1.616 1.612 1.609 1.602 1.599 1.583 1.579 1.566 1.564 1.554 1.550 1.547 1.543 1.542 1.537 1.531 1.526 1.485
B (m) 3.58
A (t) 3.64
D (m) 1.55
C (m) 1.63
E (s) 1.48
2.00 2.01
2.17 2.23 18.05
11.0 10.5 10.0 9.5 9.0 8.5 8.0 7.5 7.0 6.5 6.0 5.5 5.0 4.5 4.0 3.5 3.0 2.5 2.0 1.5 1.0 0.5 0.0
f1 (ppm)

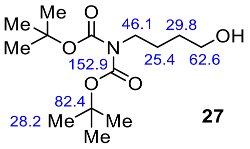
Me
Me
Me
O
O
N
OH
O
O
Me
Me
Me
46.1 29.8
152.9
25.4 62.6
82.4
28.2

27

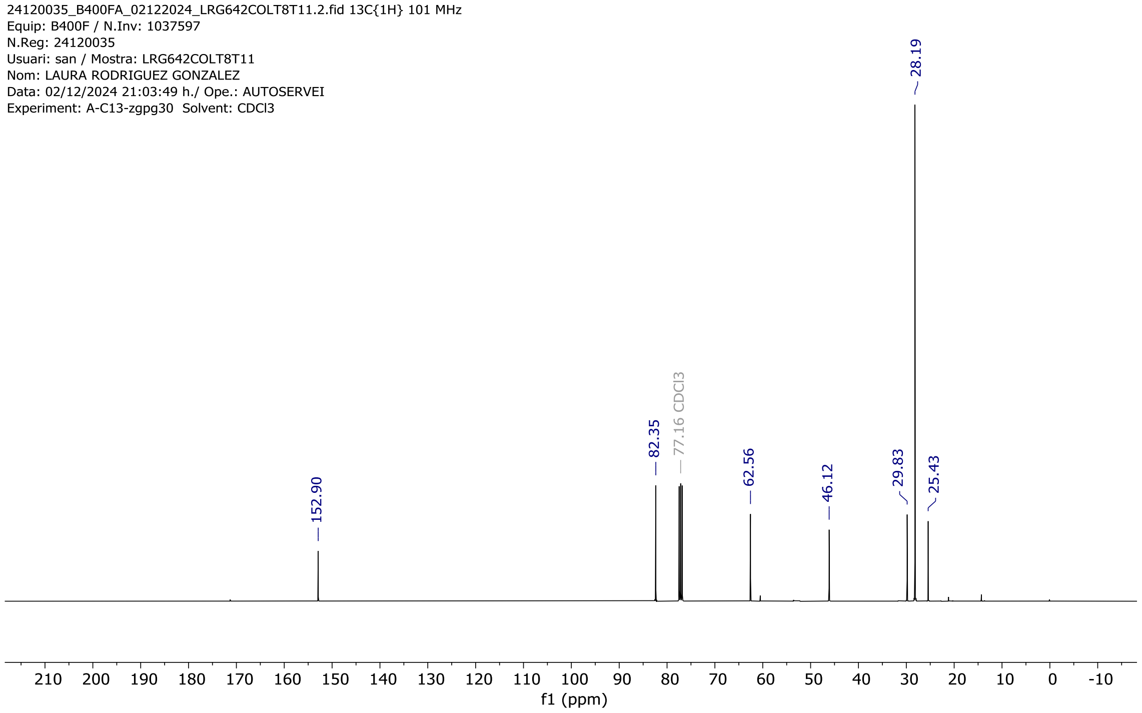
24120035_B400FA_02122024_LRG642COLT8T11.2.fid 13C{1H} 101 MHz
Equip: B400F / N.Inv: 1037597
N.Reg: 24120035
Usuari: san / Mostra: LRG642COLT8T11
Nom: LAURA RODRIGUEZ GONZALEZ
Data: 02/12/2024 21:03:49 h./ Ope.: AUTOSERVEI
Experiment: A-C13-zgpg30 Solvent: CDCl3
28.19
152.90
82.35
77.16 CDCl3
62.56
46.12
29.83
25.43
210 200 190 180 170 160 150 140 130 120 110 100 90 80 70 60 50 40 30 20 10 0 -10
f1 (ppm)

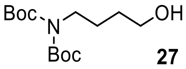

27

2D-COSY

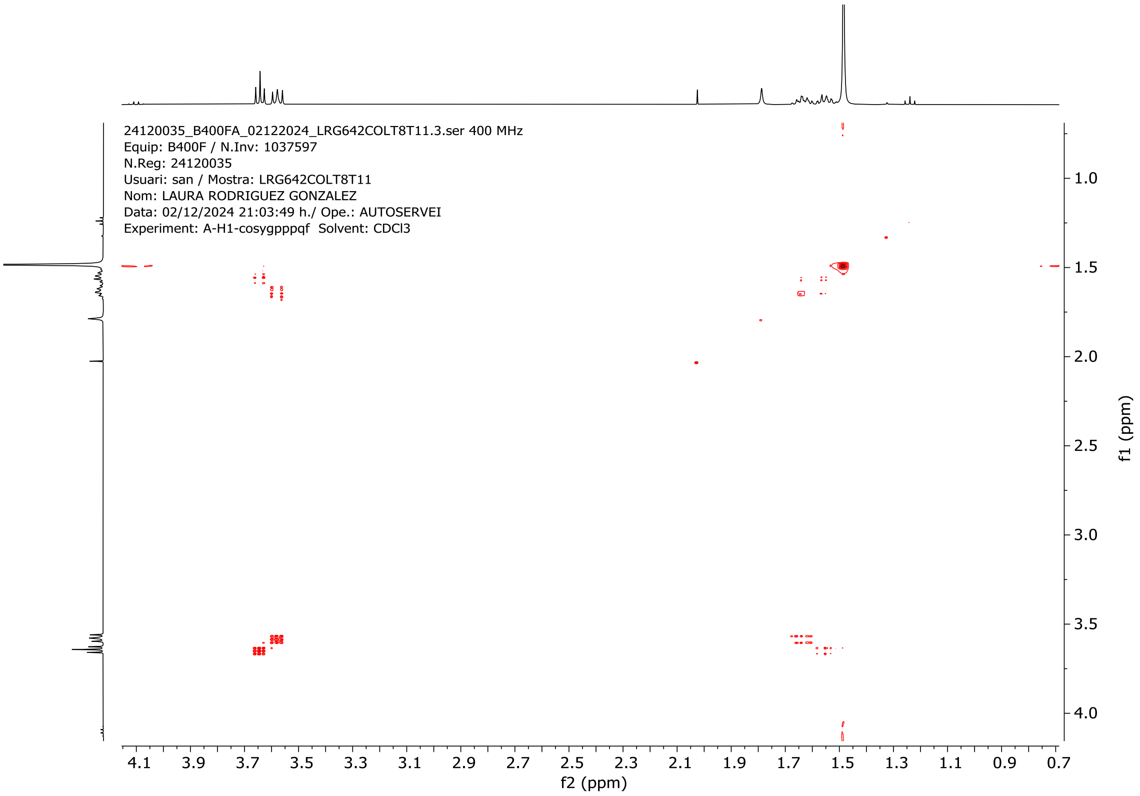

2D-HSQC

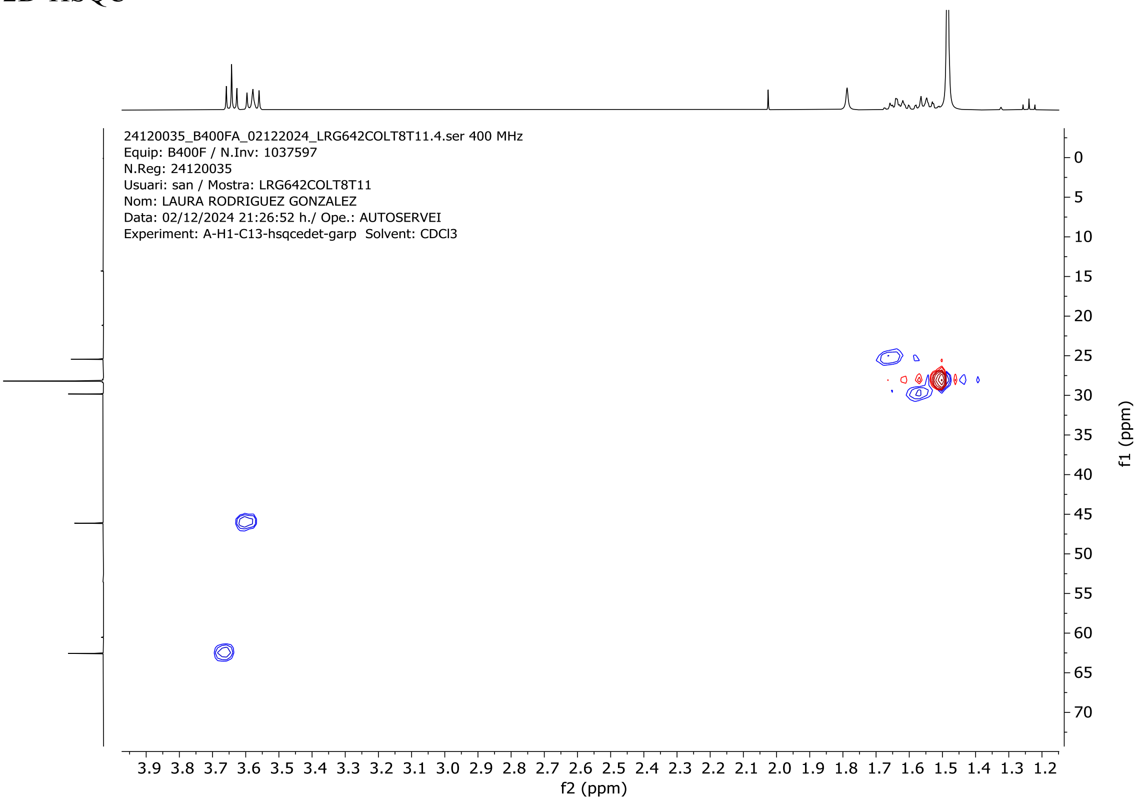

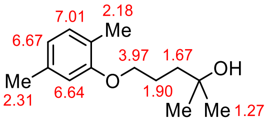

28

24070110_B400FA_03072024_LRG618PREPTOP.1.fid 1H 400 MHz
Equip: B400F / N.Inv: 1037597
N.Reg: 24070110
Usuari: san / Mostra: LRG618PREPTOP
Nom: LAURA RODRIGUEZ GONZALEZ
Data: 03/07/2024 09:36:38 h./ Ope.: AUTOSERVEI
Experiment: A-H1-zg30 Solvent: CDCl3

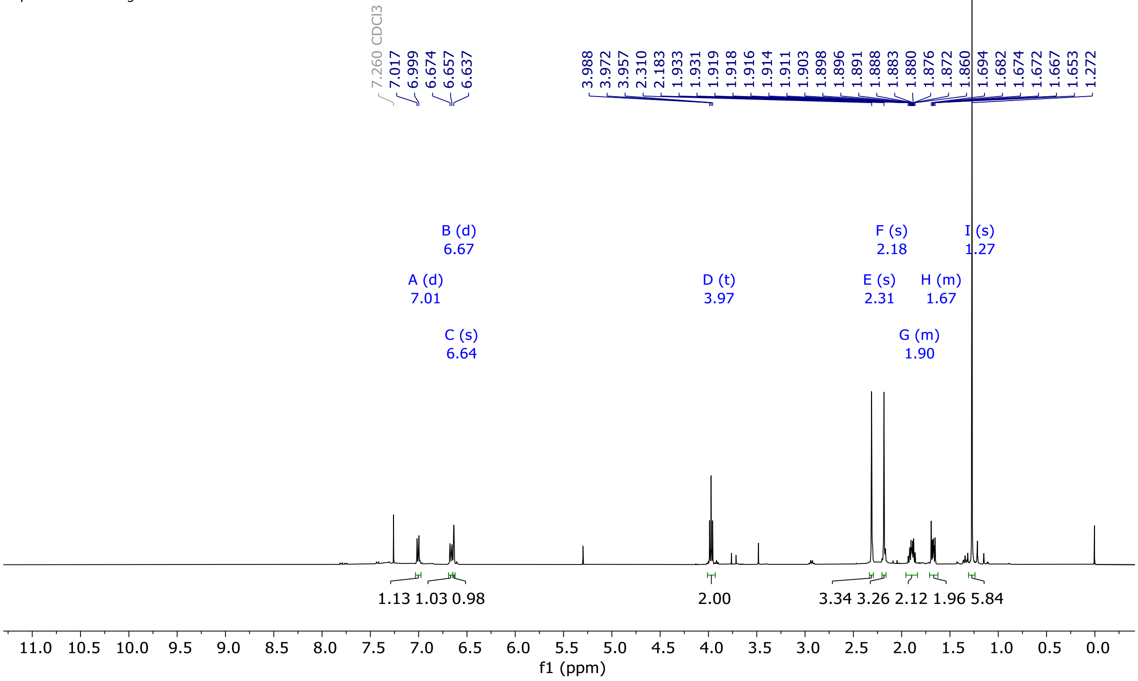

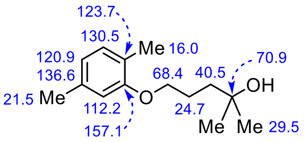

28

24070109_B400FA_03072024_LRG618PREP.2.fid 13C{1H} 101 MHz
Equip: B400F / N.Inv: 1037597
N.Reg: 24070109
Usuari: san / Mostra: LRG618PREP
Nom: LAURA RODRIGUEZ GONZALEZ
Data: 03/07/2024 21:47:20 h./ Ope.: AUTOSERVEI
Experiment: A-C13-zgpg30 Solvent: CDCl3

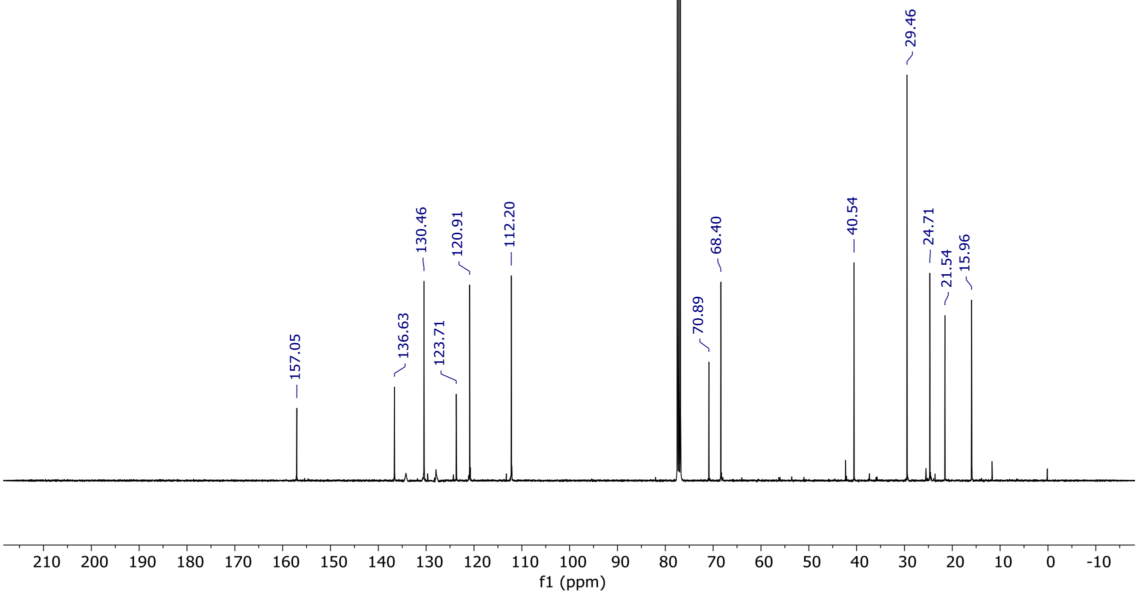

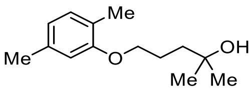

28

2D-COSY

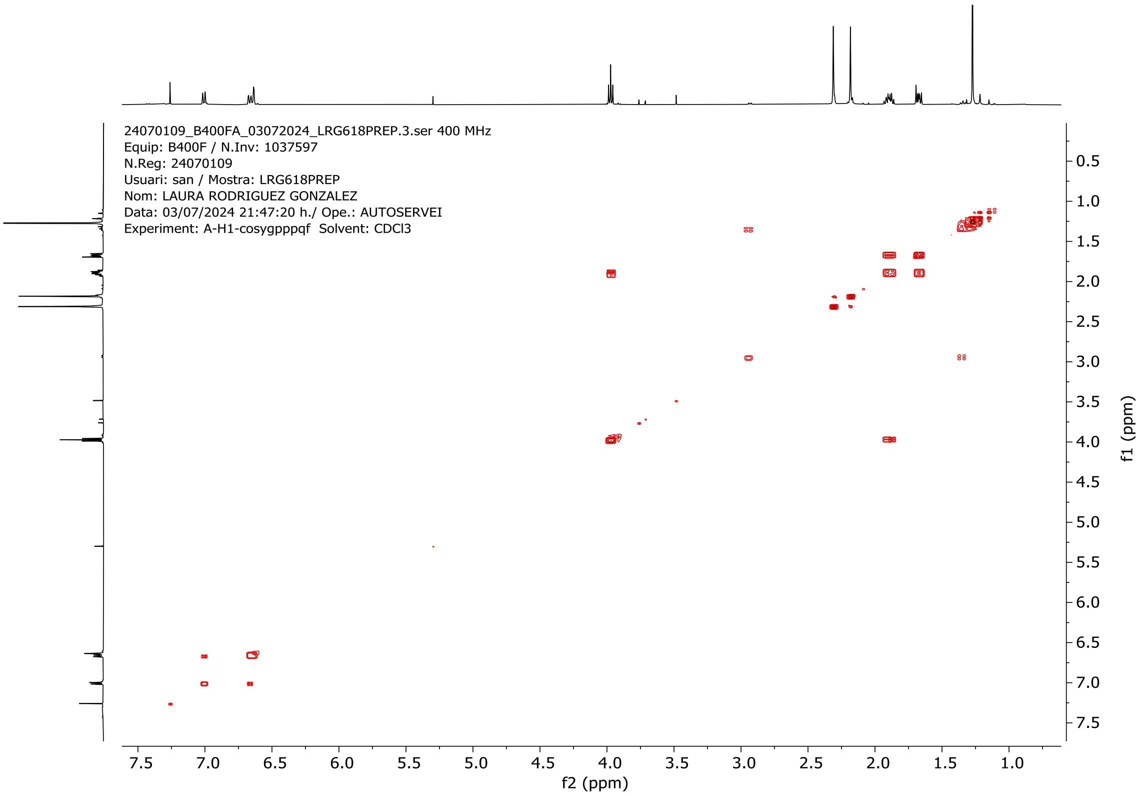

2D-HSQC

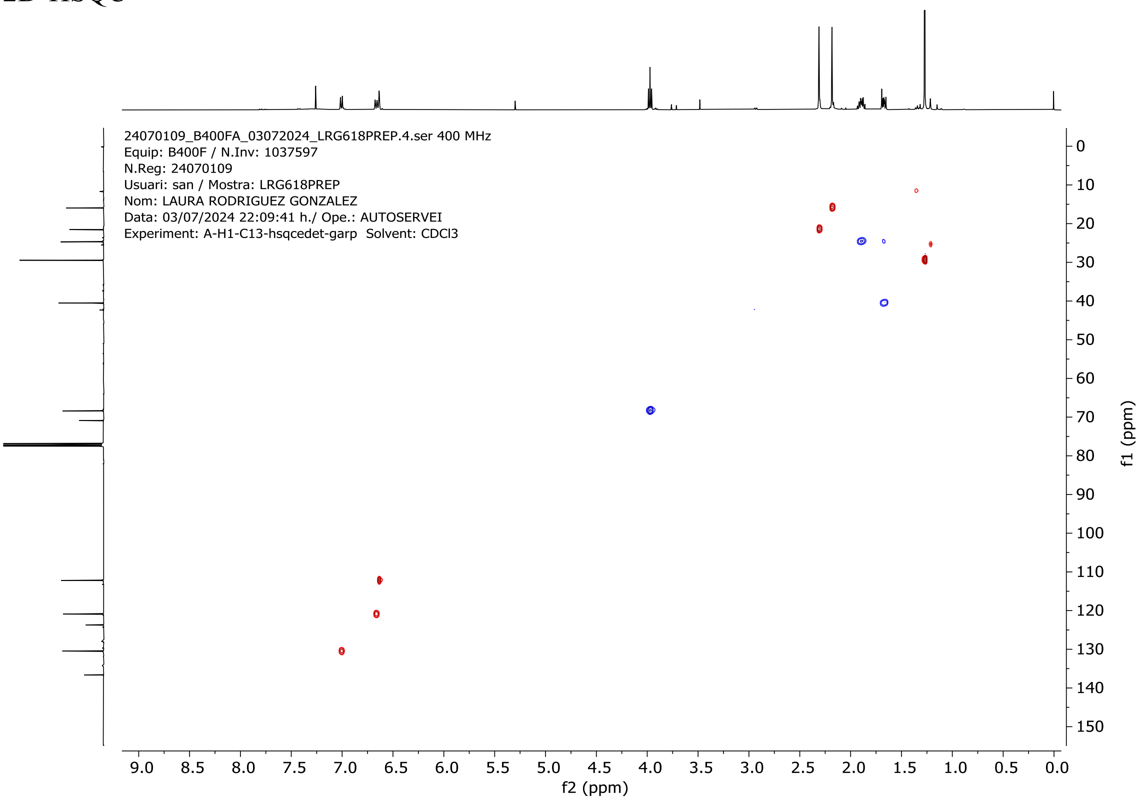

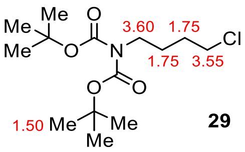

29

LRG677COLT10T11.1.fid 1H 400 MHz
Equip: B400F / N.Inv: 1037597
N.Reg: 24120162
Usuari: san / Mostra: LRG677COLT10T11
Nom: LAURA RODRIGUEZ GONZALEZ
Data: 09/12/2024 16:42:50 h./ Ope.: AUTOSERVEI
Experiment: A-H1-zg30 Solvent: CDCl3

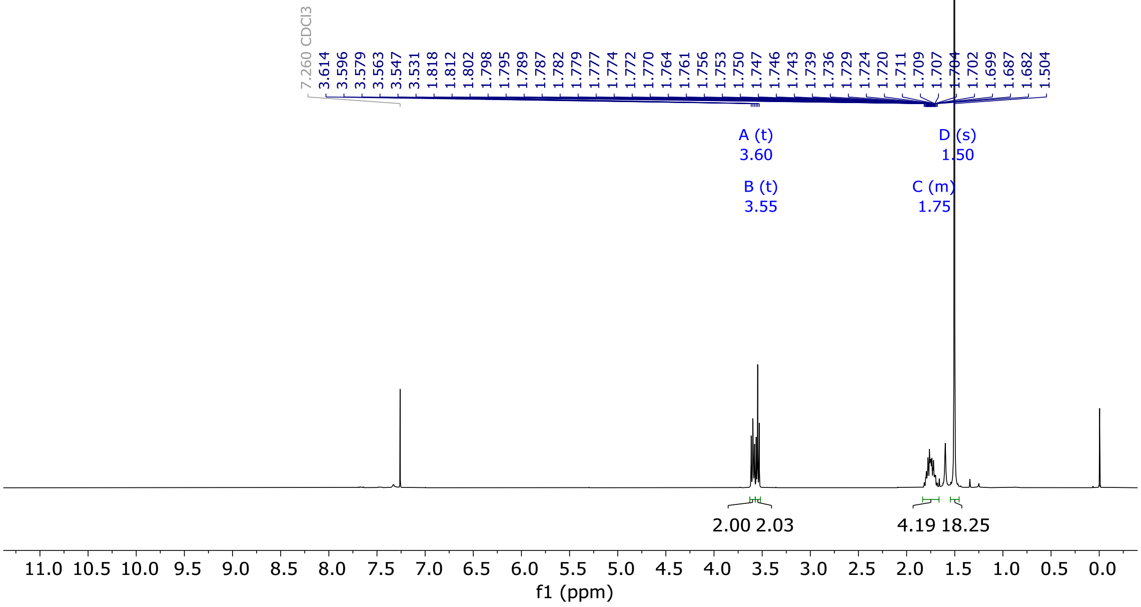

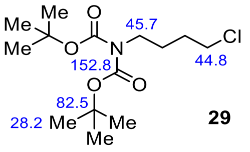

29

LRG677COLT10T11.2.fid 13C{1H} 101 MHz
Equip: B400F / N.Inv: 1037597
N.Reg: 24120162
Usuari: san / Mostra: LRG677COLT10T11
Nom: LAURA RODRIGUEZ GONZALEZ
Data: 09/12/2024 23:52:26 h./ Ope.: AUTOSERVEI
Experiment: A-C13-zgpg30 Solvent: CDCl3

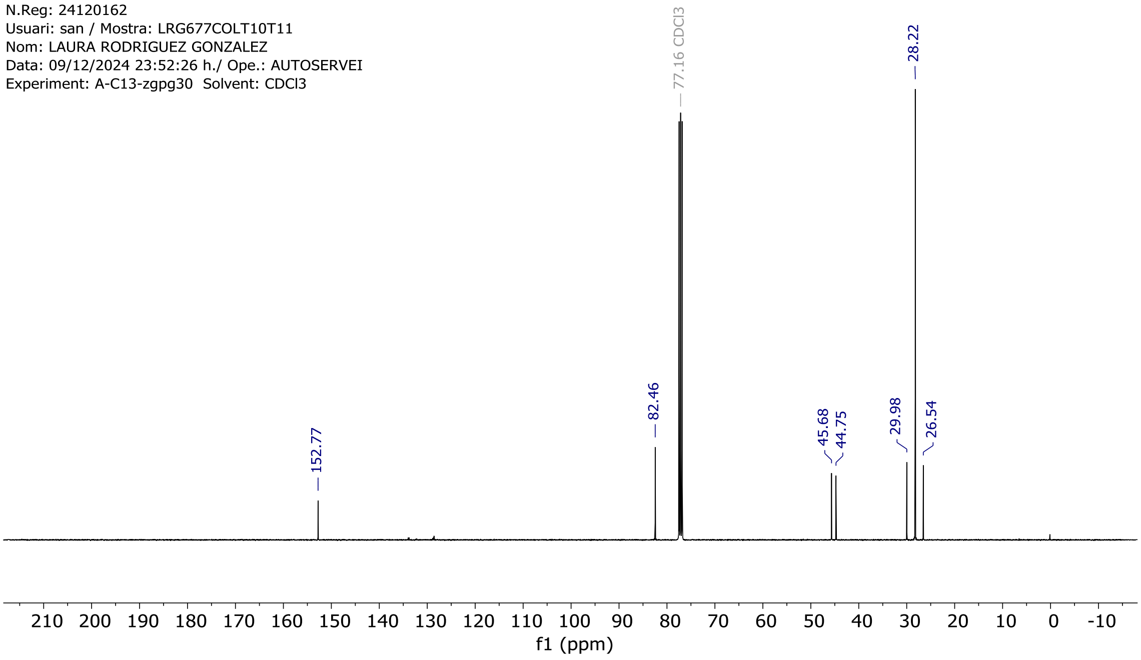

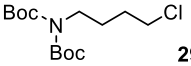

29

2D-COSY

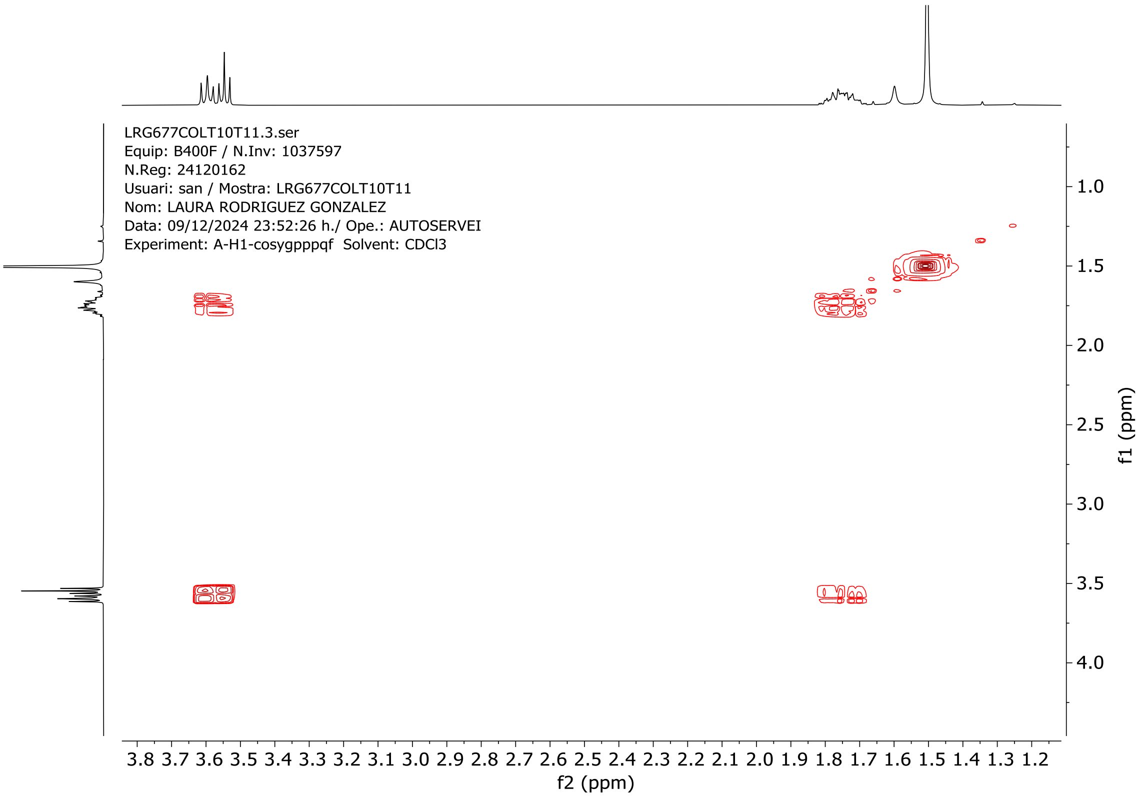

2D-HSQC

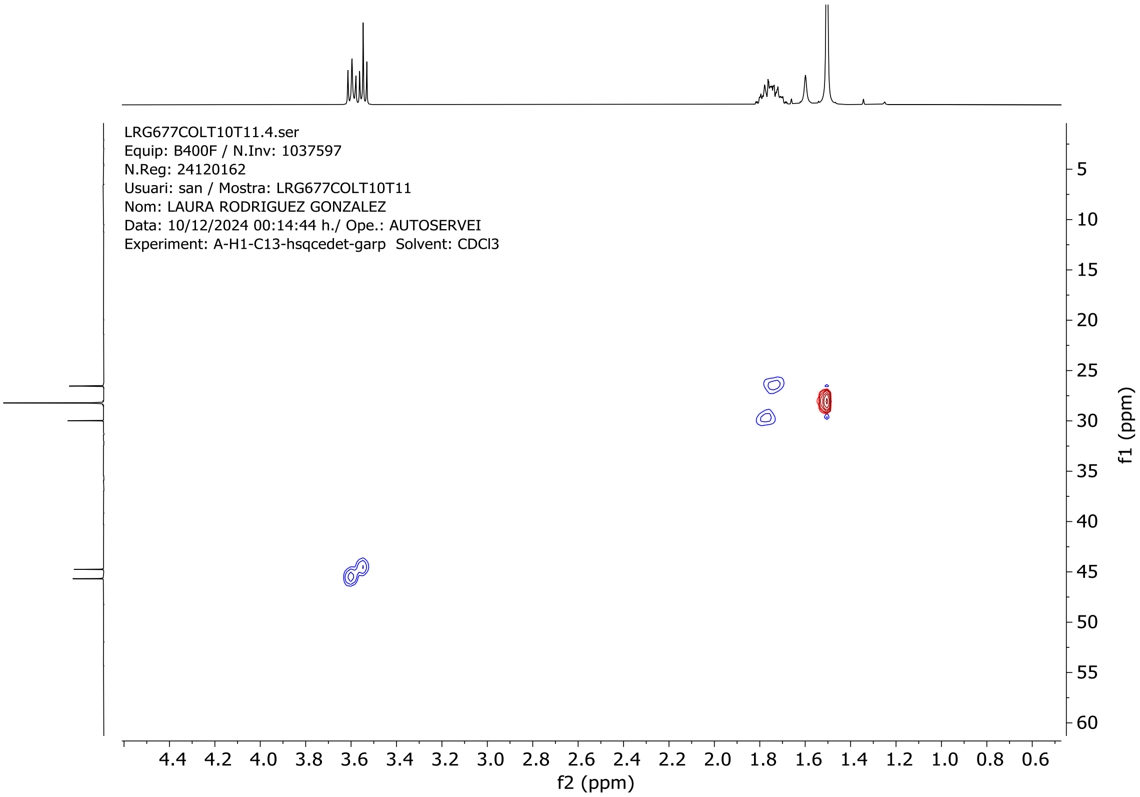

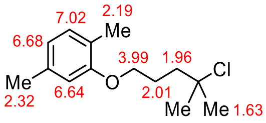

30

24070614_B400FA_15072024_LRG626PREP.1.fid 1H 400 MHz
Equip: B400F / N.Inv: 1037597
N.Reg: 24070614
Usuari: san / Mostra: LRG626PREP
Nom: LAURA RODRIGUEZ GONZALEZ
Data: 15/07/2024 13:07:39 h./ Ope.: AUTOSERVEI
Experiment: A-H1-zg30 Solvent: CDCl3

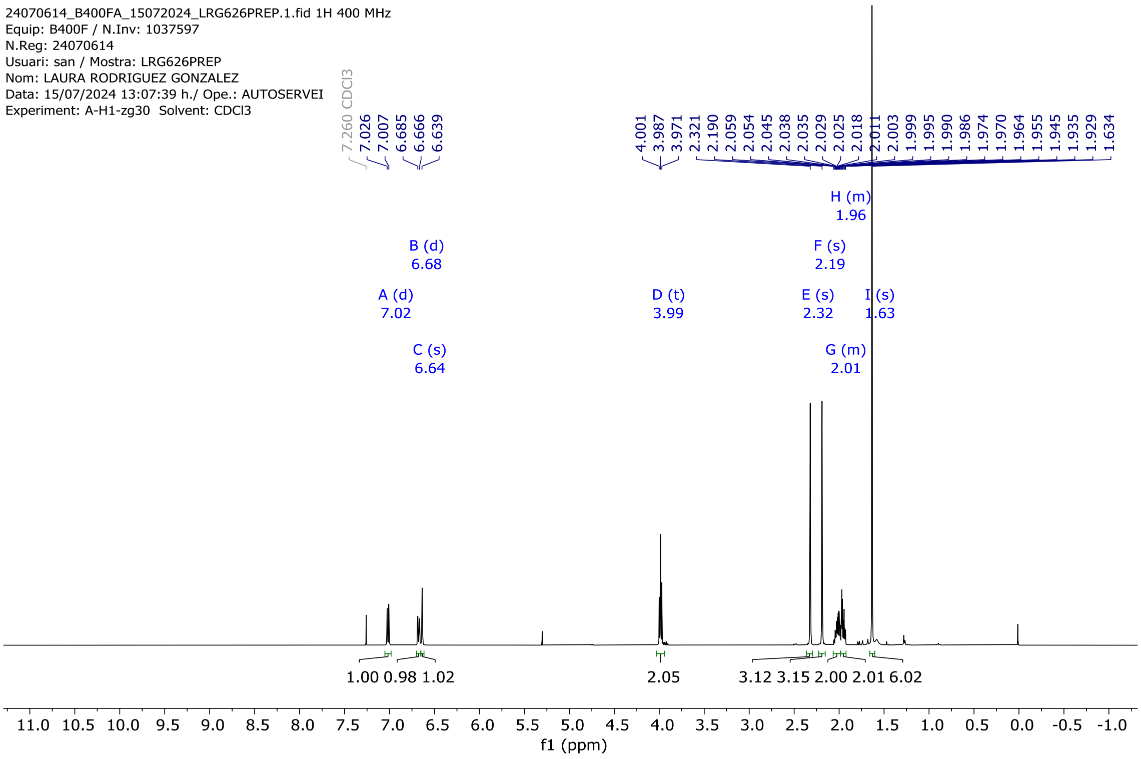

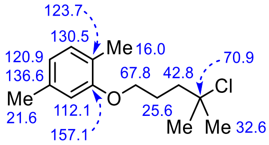

30

24070614_B400FA_15072024_LRG626PREP.2.fid 13C{1H} 101 MHz
Equip: B400F / N.Inv: 1037597
N.Reg: 24070614
Usuari: san / Mostra: LRG626PREP
Nom: LAURA RODRIGUEZ GONZALEZ
Data: 15/07/2024 21:08:48 h./ Ope.: AUTOSERVEI
Experiment: A-C13-zgpg30 Solvent: CDCl3

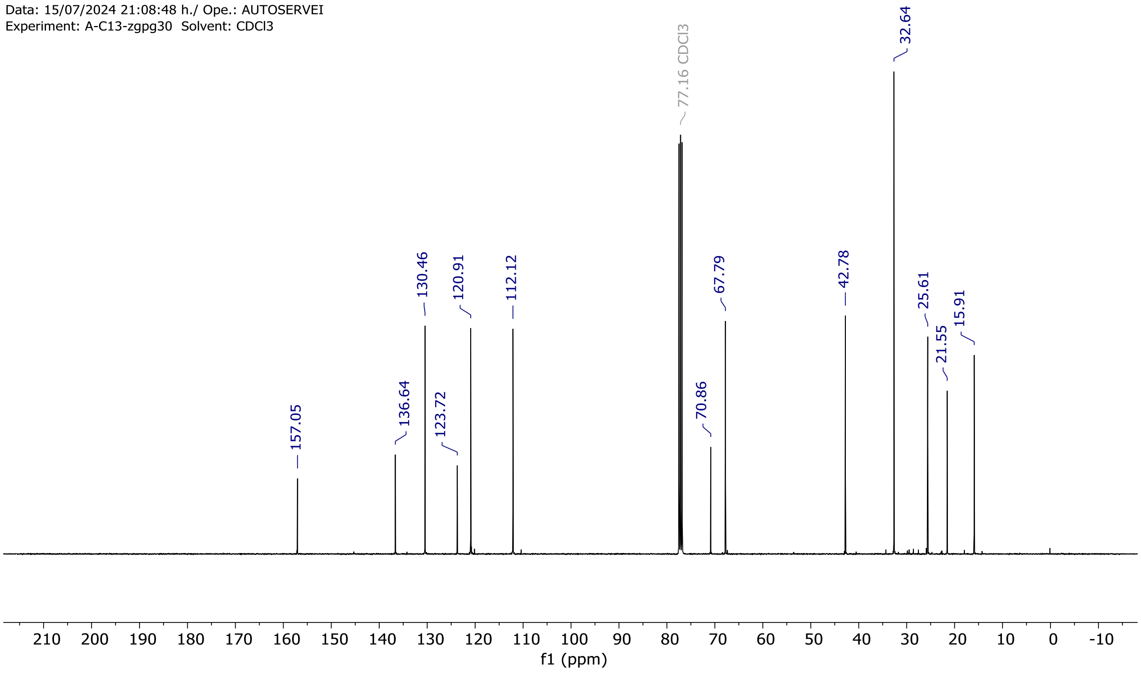

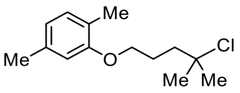

30

2D-COSY

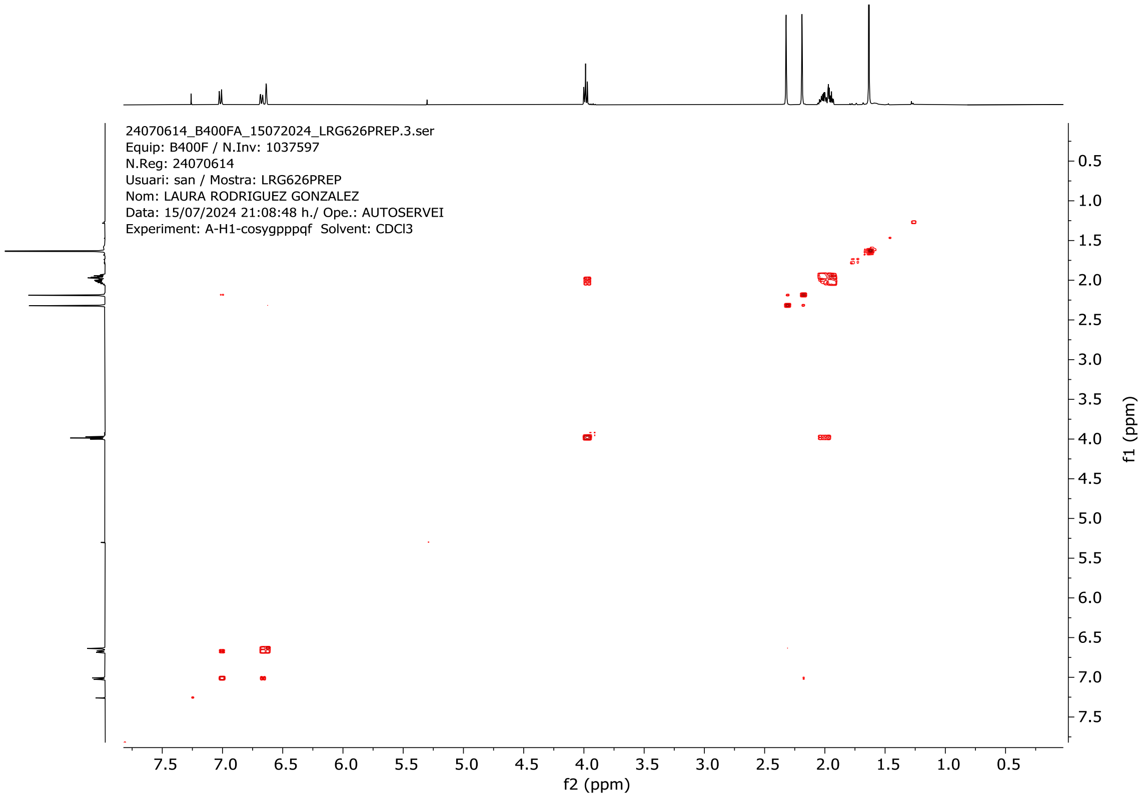

2D-HSQC

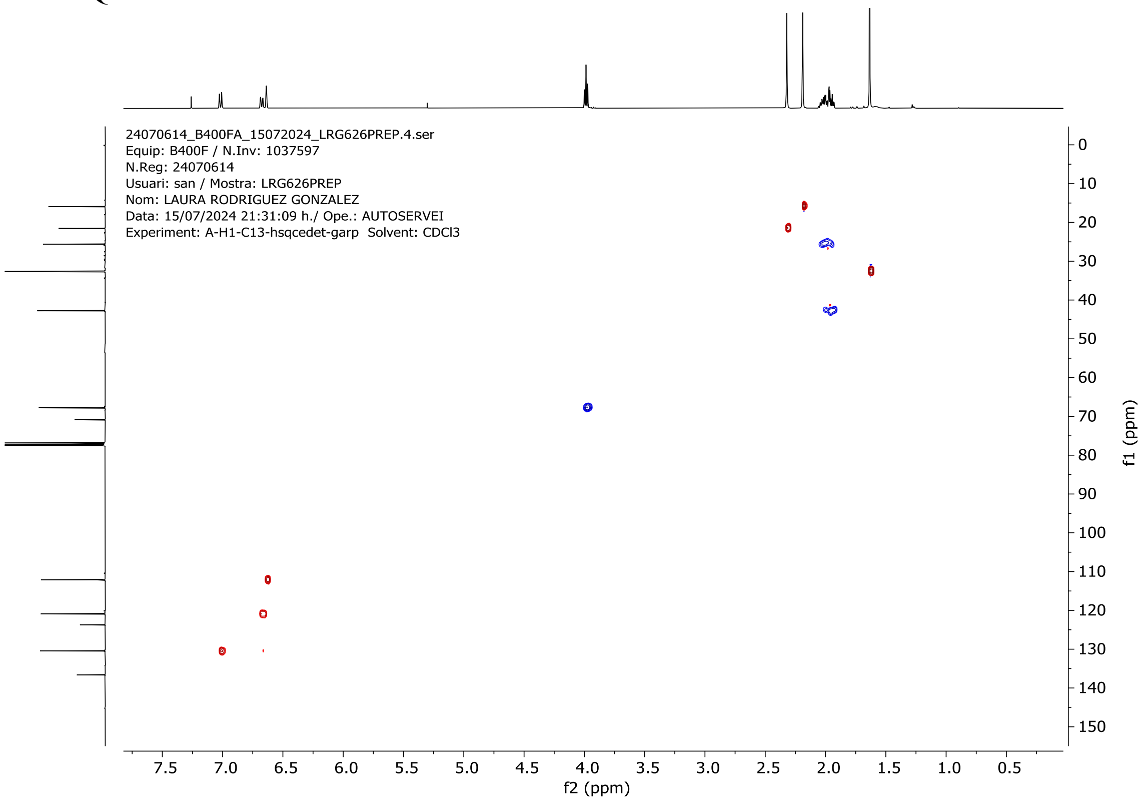

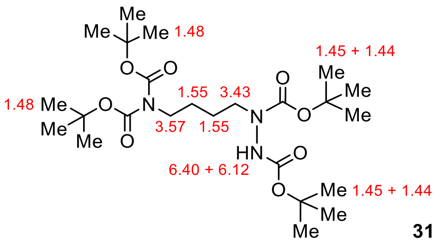

31 The product was isolated as a mixture of rotamers

25030299_B400FA_12032025_BB3013CH.1.fid 1H 400 MHz
Equip: B400F / N.Inv: 1037597
N.Reg: 25030299
Usuari: san / Mostra: BB3013CH
Nom: LAURA RODRIGUEZ GONZALEZ
Data: 12/03/2025 12:38:15 h./ Ope.: AUTOSERVEI
Experiment: A-H1-zg30 Solvent: CDCl3

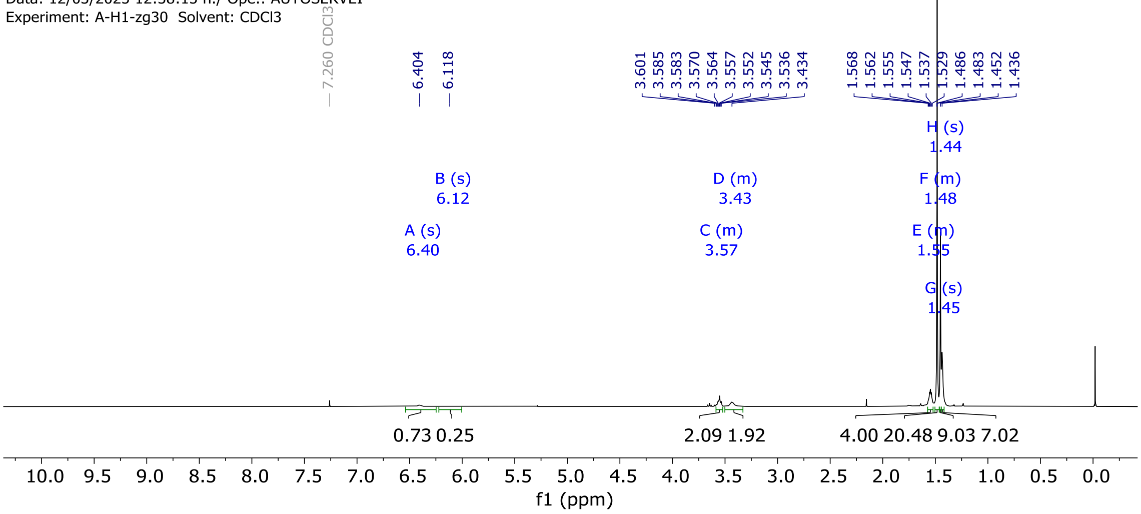

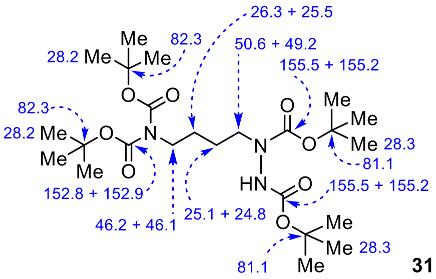

31

25030299_B400FA_12032025_BB3013CH.2.fid 13C{1H} 101 MHz
Equip: B400F / N.Inv: 1037597
N.Reg: 25030299
Usuari: san / Mostra: BB3013CH
Nom: LAURA RODRIGUEZ GONZALEZ
Data: 12/03/2025 21:08:00 h./ Ope.: AUTOSERVEI
Experiment: A-C13-zgpg30 Solvent: CDCl3

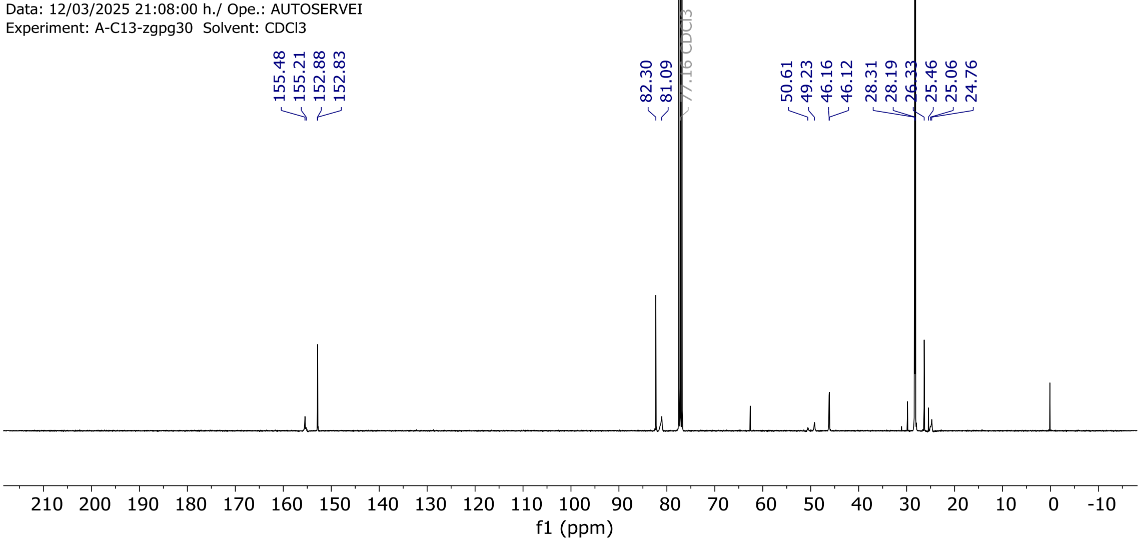

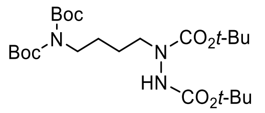

31

2D-COSY

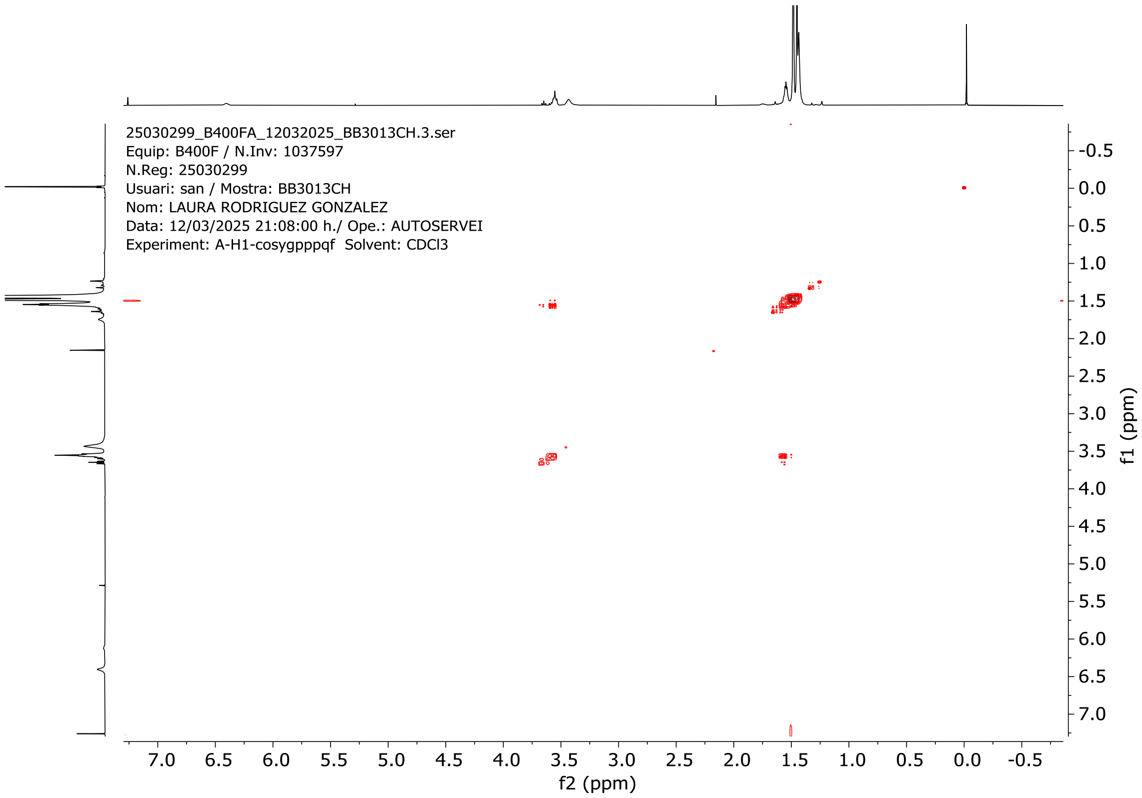

2D-HSQC

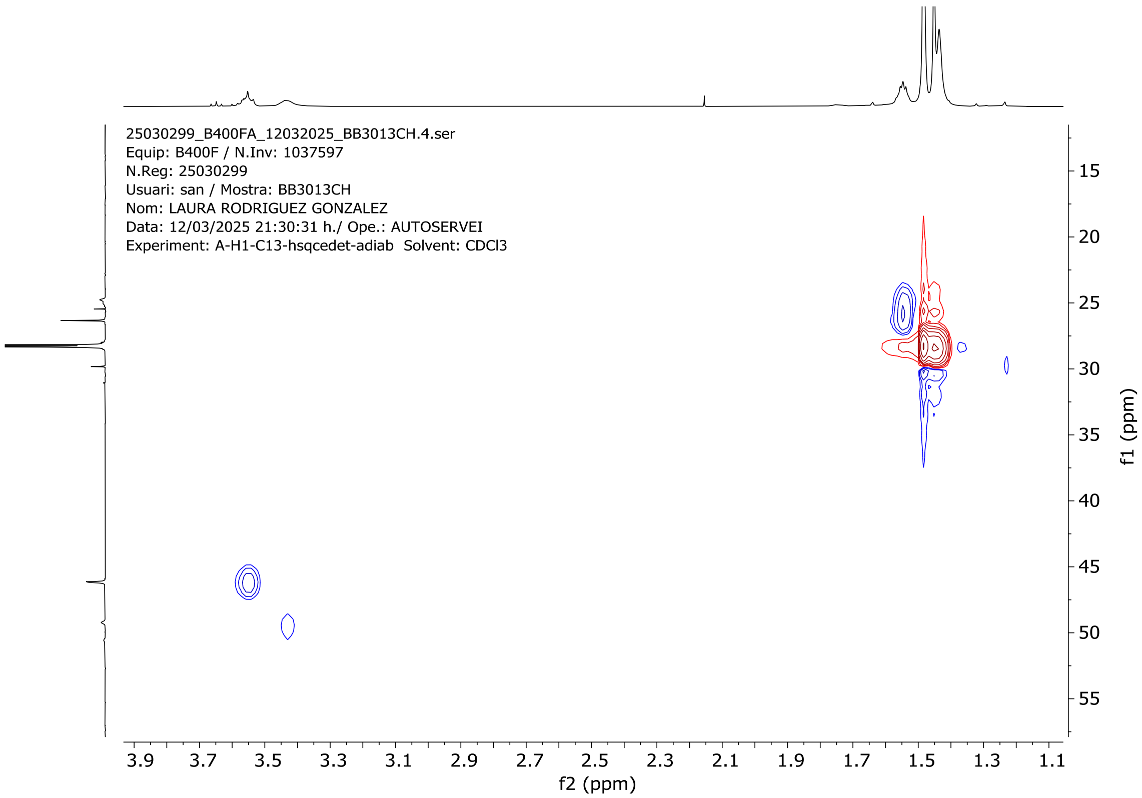

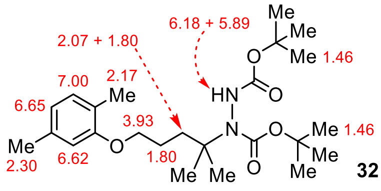

The product was isolated as a mixture of rotamers

24070286_B400FA_05072024_LRG621PREPTOP.1.fid 1H 400 MHz
Equip: B400F / N.Inv: 1037597
N.Reg: 24070286
Usuari: san / Mostra: LRG621PREPTOP
Nom: LAURA RODRIGUEZ GONZALEZ
Data: 05/07/2024 13:28:34 h./ Ope.: AUTOSERVEI
Experiment: A-H1-zg30 Solvent: CDCl3

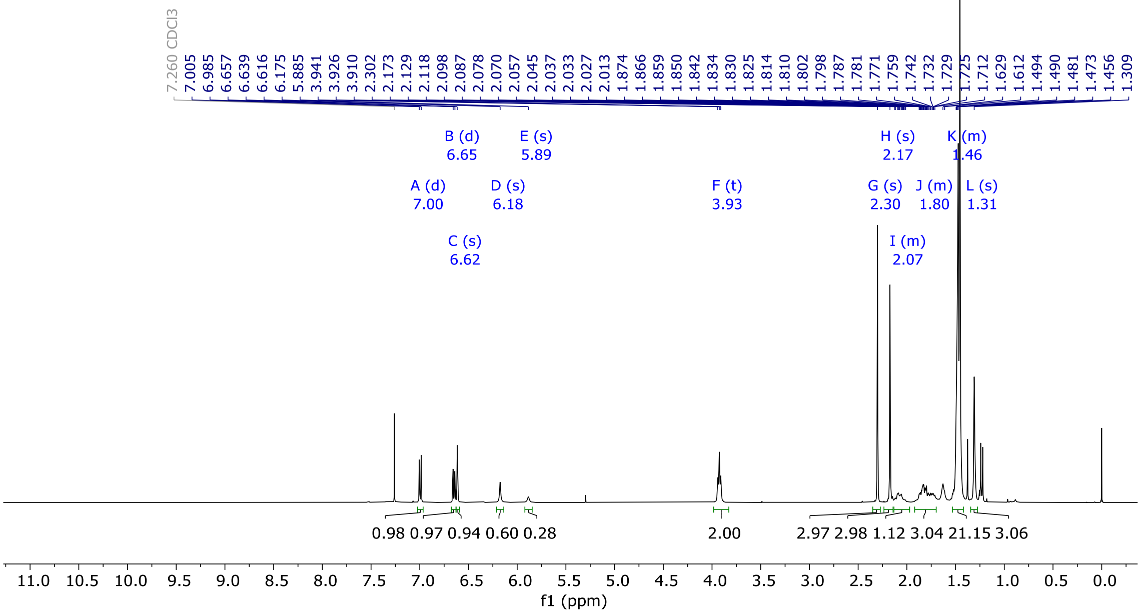

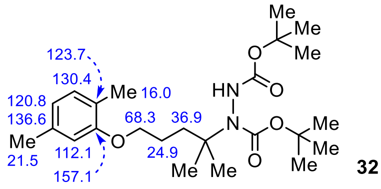

auto-05072024-141116.2.fid 13C{1H} 101 MHz
Equip: B400Q / N.Inv: 1035091
N.Reg: 24070272
Usuari: san / Mostra: LRG621PREPTOPCH
Nom: LAURA RODRIGUEZ GONZALEZ
Data: 06/07/2024 11:19:03 h./ Ope.: AUTOSERVEI
Experiment: A_13C-zgpg30 Solvent: CDCl3 Operator:

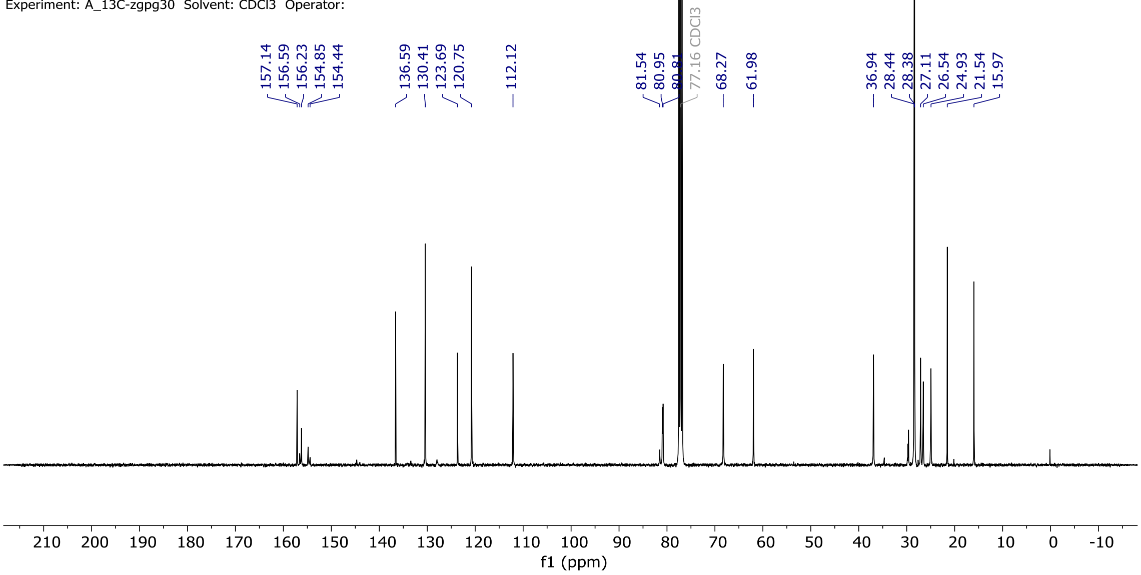

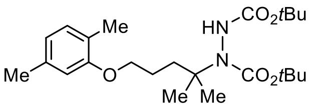

32

2D-COSY

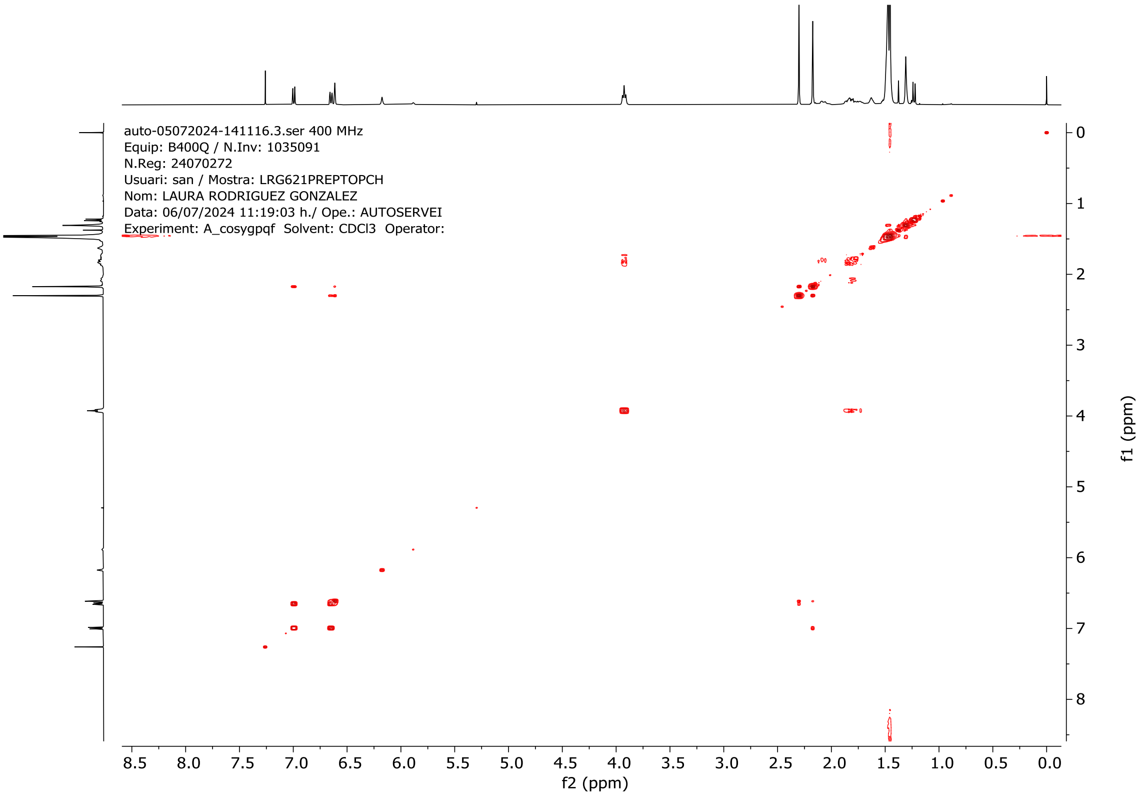

2D-HSQC

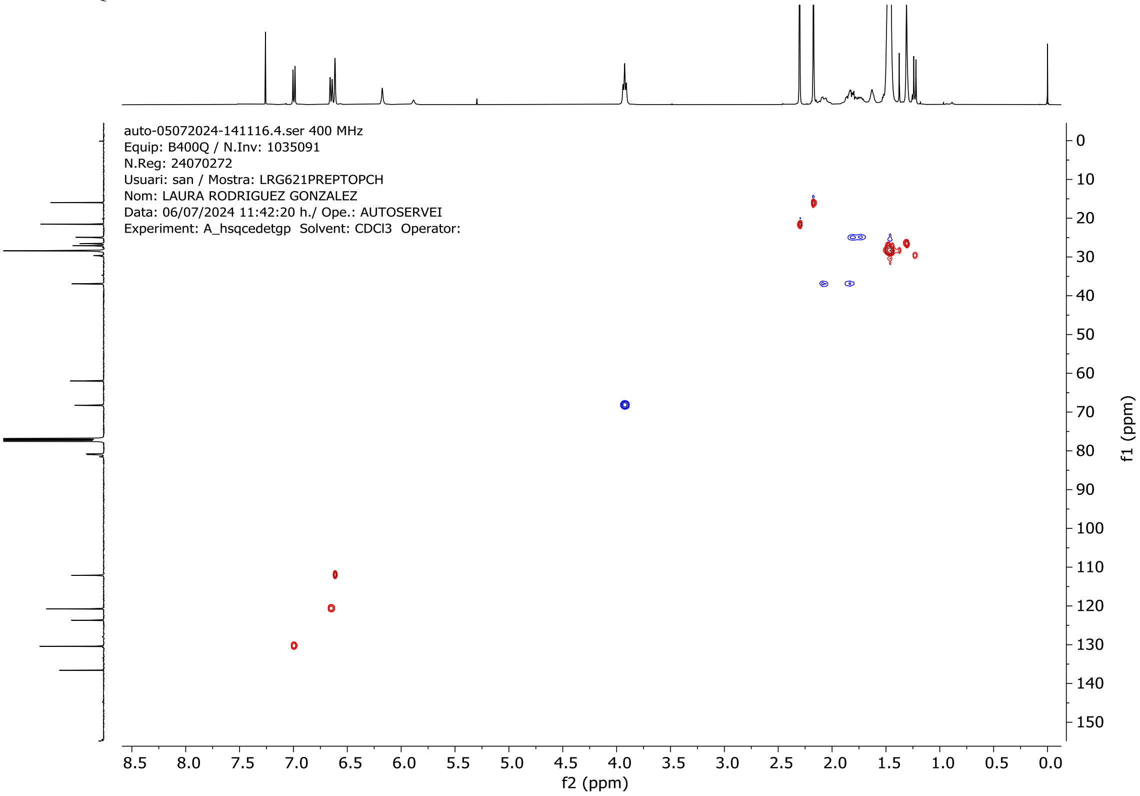

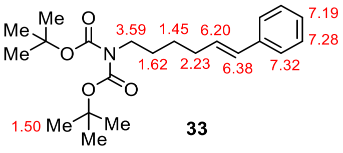

33

4123-2024_B400FA_12042024_LRG580PREPCH.1.fid 1H 400 MHz
Equip: B400F / N.Inv: 1037597
N.Reg: 4123/2024
Usuari: san / Mostra: LRG580PREPCH
Nom: LAURA RODRIGUEZ GONZALEZ
Data: 12/04/2024 19:19:50 h./ Ope.: servei Unitat RMN
Experiment: A-H1-zg30 Solvent: CDCl3

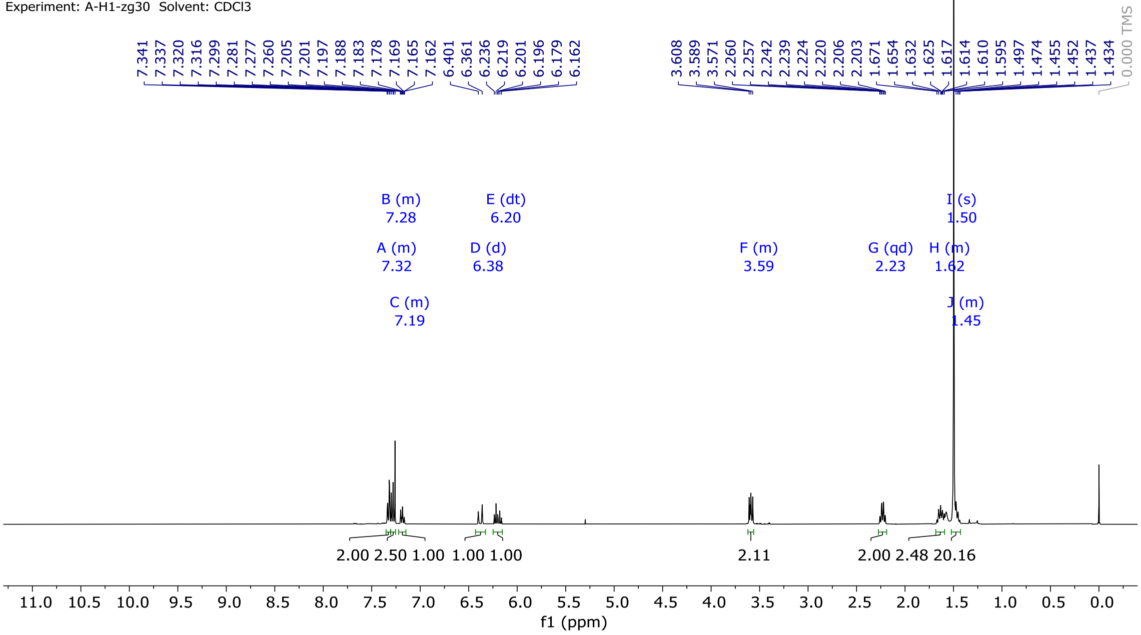

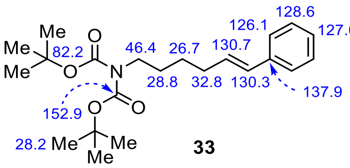

33

4123-2024_B400FA_12042024_LRG580PREPCH.4.fid 13C{1H} 101 MHz
Equip: B400F / N.Inv: 1037597
N.Reg: 4123/2024
Usuari: san / Mostra: LRG580PREPCH
Nom: LAURA RODRIGUEZ GONZALEZ
Data: 12/04/2024 20:12:14 h./ Ope.: servei Unitat RMN
Experiment: A-C13-zgpg30 Solvent: CDCl3

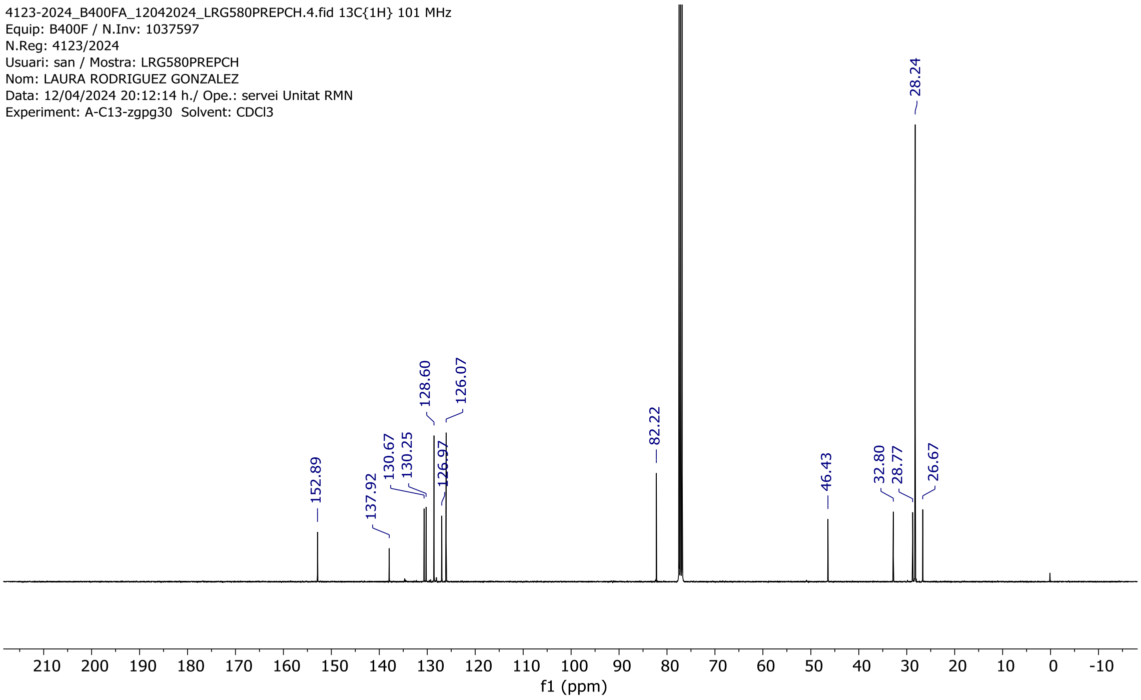

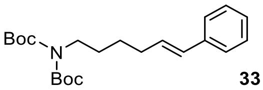

33

2D-COSY

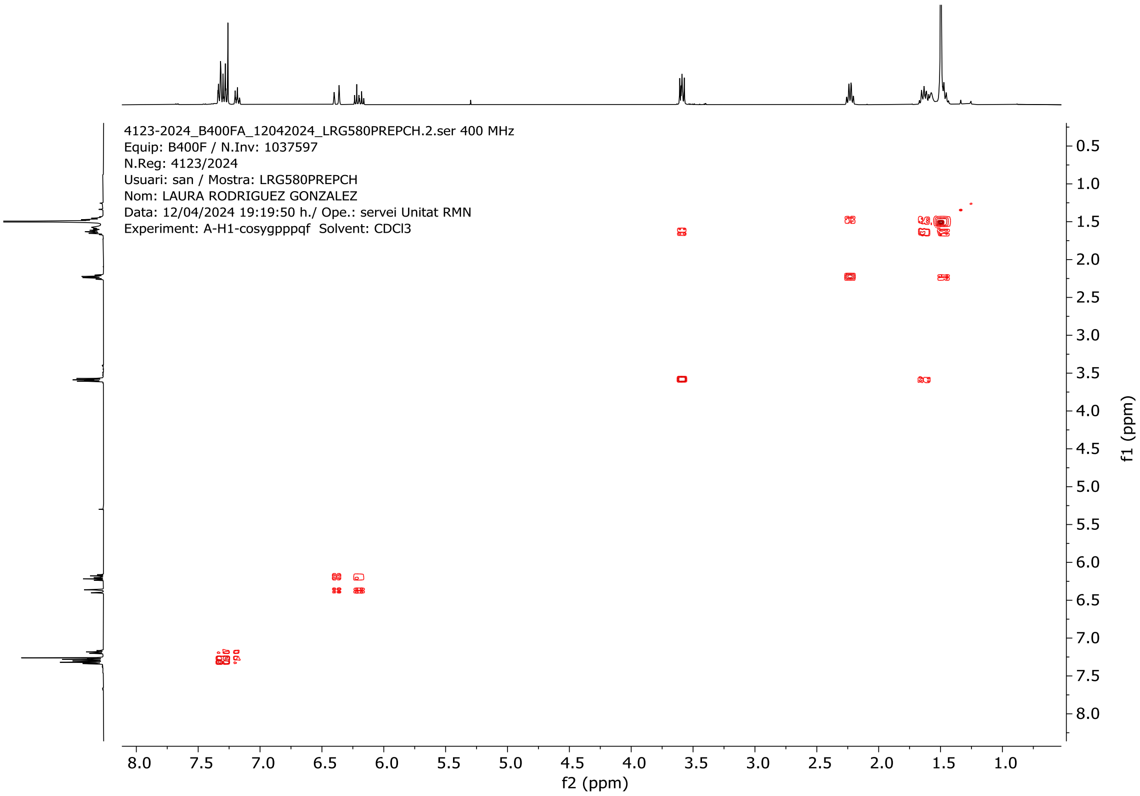

2D-HSQC

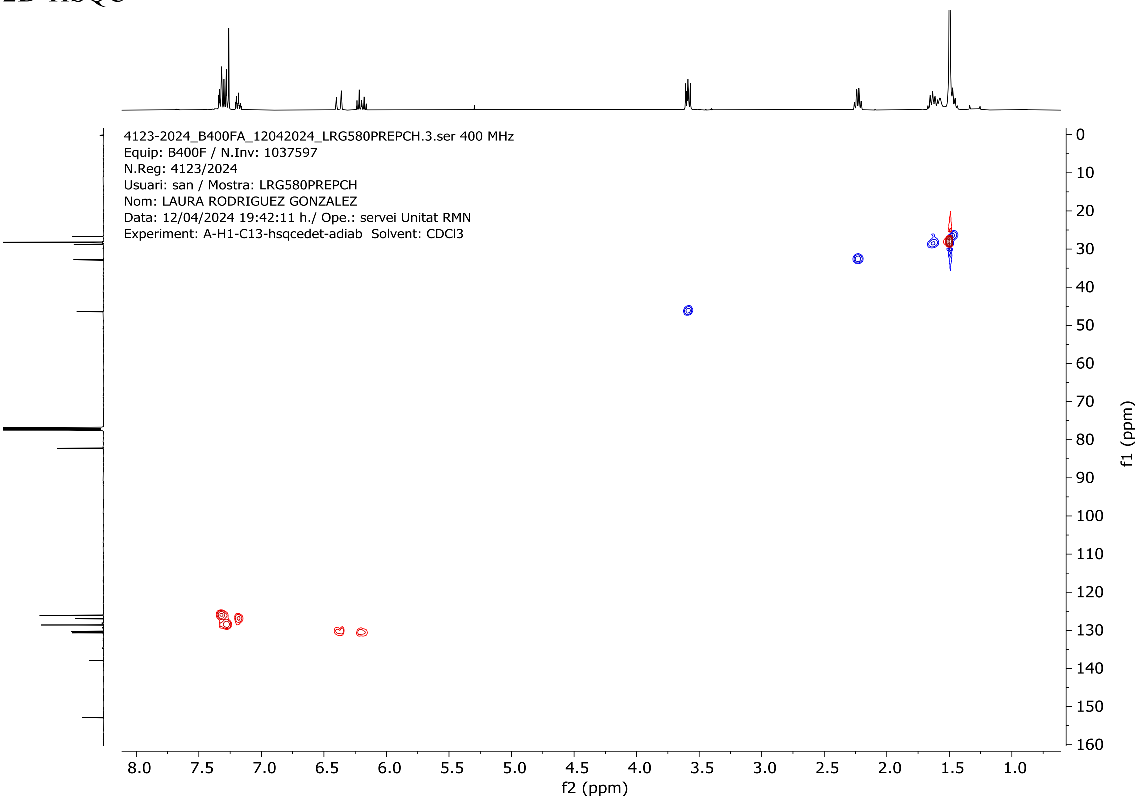

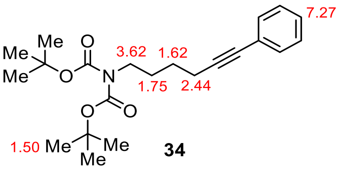

34

24040430_B400FA_11042024_LRG578CH.1.fid 1H 400 MHz
Equip: B400F / N.Inv: 1037597
N.Reg: 24040430
Usuari: san / Mostra: LRG578CH
Nom: LAURA RODRIGUEZ GONZALEZ
Data: 10/04/2024 15:00:13 h./ Ope.: AUTOSERVEI
Experiment: A-H1-zg30 Solvent: CDCl3

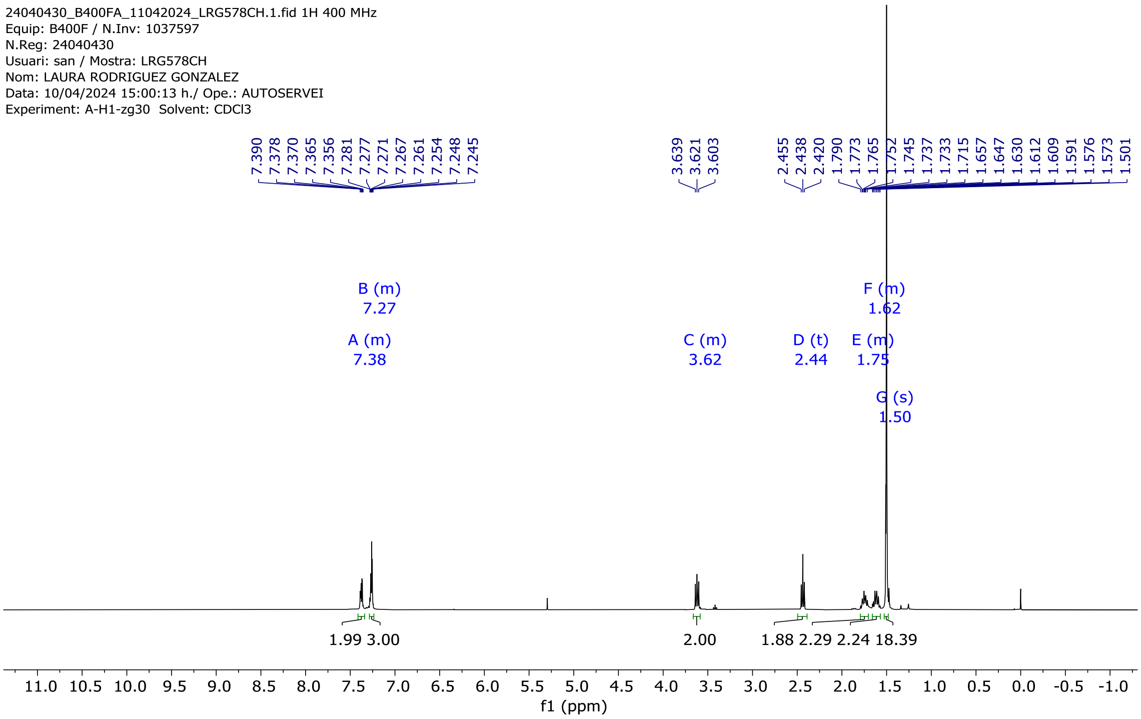

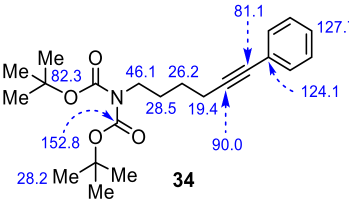

34

24040430_B400FA_11042024_LRG578CH.2.fid 13C{1H} 101 MHz
Equip: B400F / N.Inv: 1037597
N.Reg: 24040430
Usuari: san / Mostra: LRG578CH
Nom: LAURA RODRIGUEZ GONZALEZ
Data: 11/04/2024 03:06:13 h./ Ope.: AUTOSERVEI
Experiment: A-C13-zgpg30 Solvent: CDCl3

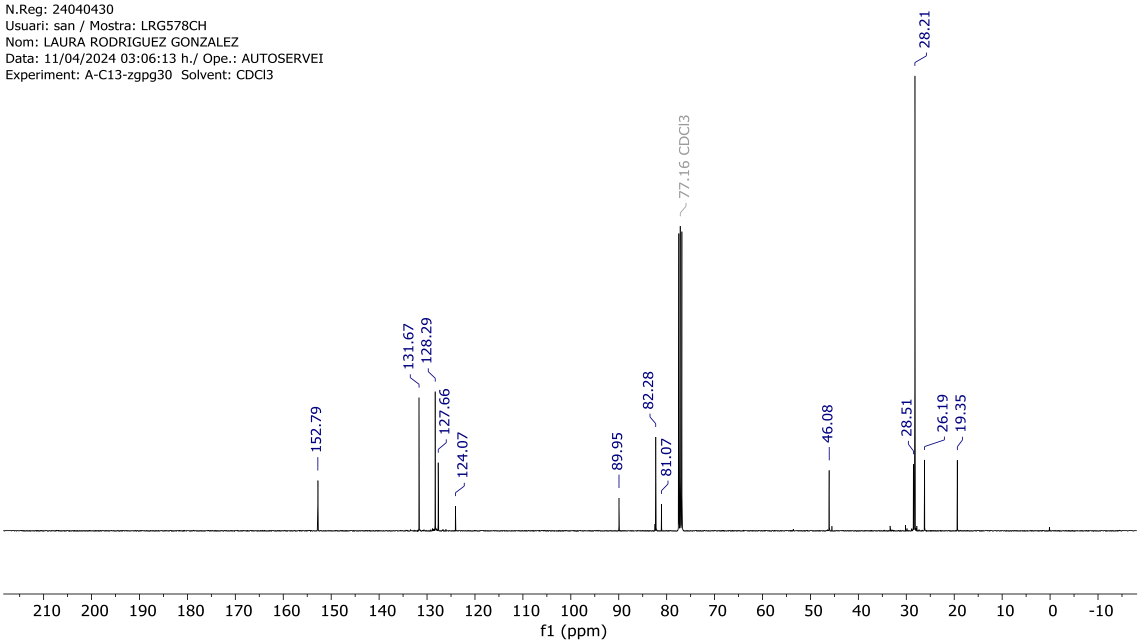

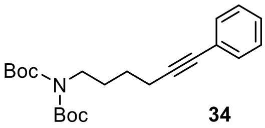

34

2D-COSY

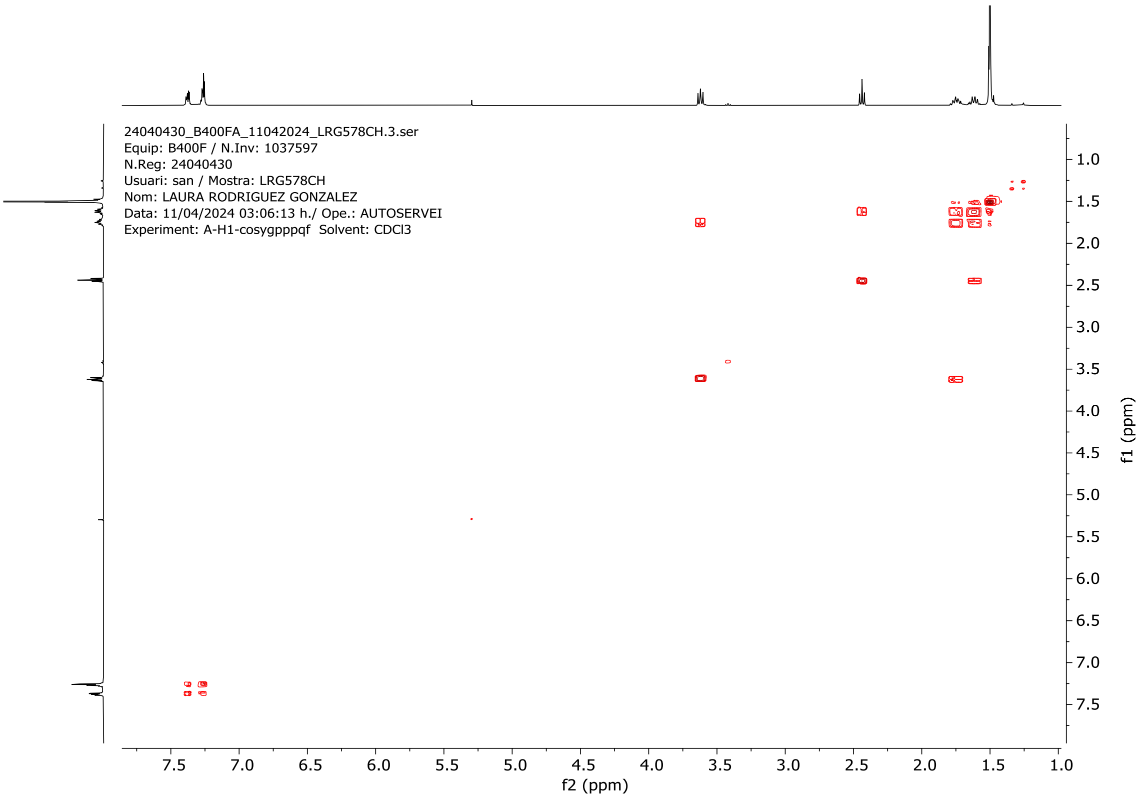

2D-HSQC

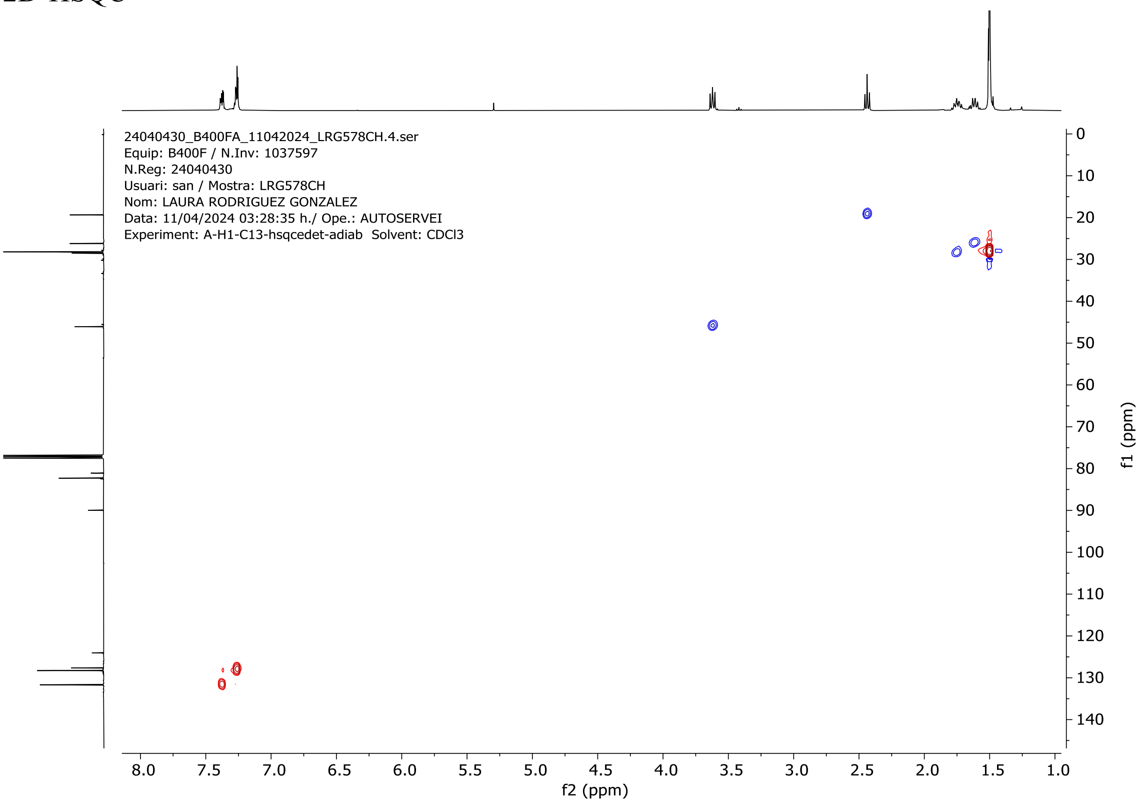

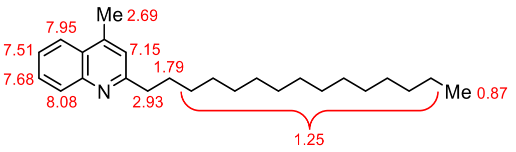

35

24050285_B400FA_08052024_LRG595PREP.1.fid 1H 400 MHz
Equip: B400F / N.Inv: 1037597
N.Reg: 24050285
Usuari: san / Mostra: LRG595PREP
Nom: LAURA RODRIGUEZ GONZALEZ
Data: 08/05/2024 17:10:21 h./ Ope.: AUTOSERVEI
Experiment: A-H1-zg30 Solvent: CDCl3

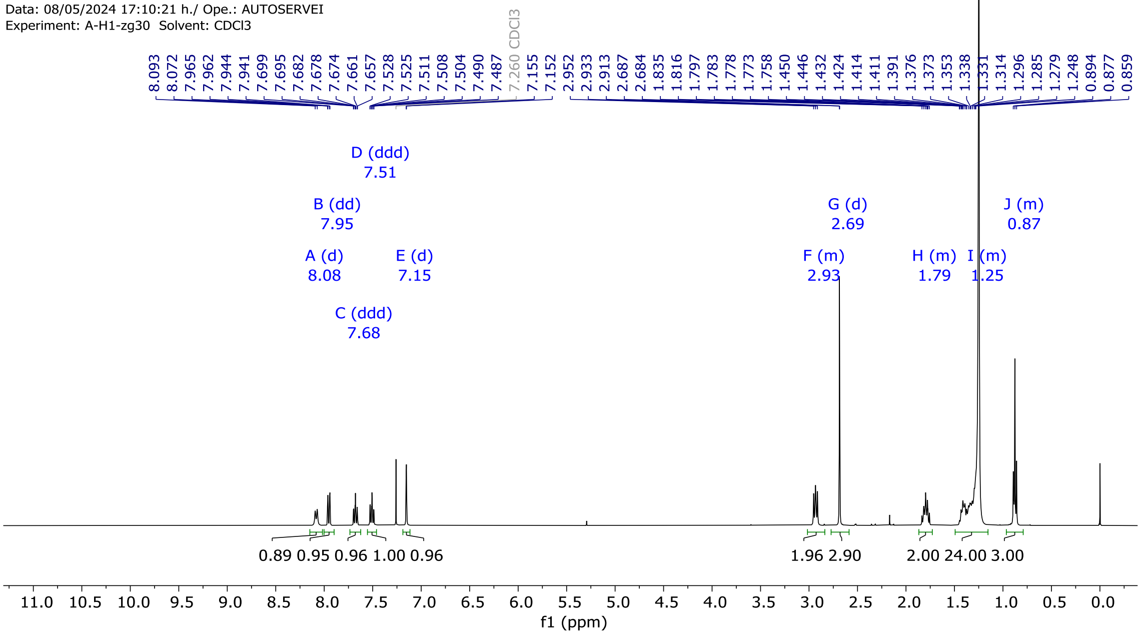

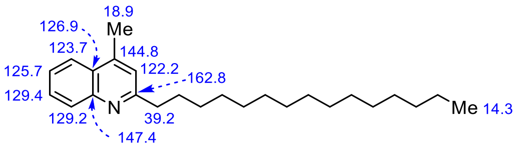

35

24050285_B400FA_09052024_LRG595PREP.2.fid 13C{1H} 101 MHz
Equip: B400F / N.Inv: 1037597
N.Reg: 24050285
Usuari: san / Mostra: LRG595PREP
Nom: LAURA RODRIGUEZ GONZALEZ
Data: 09/05/2024 02:31:11 h./ Ope.: AUTOSERVEI
Experiment: A-C13-zgpg30 Solvent: CDCl3

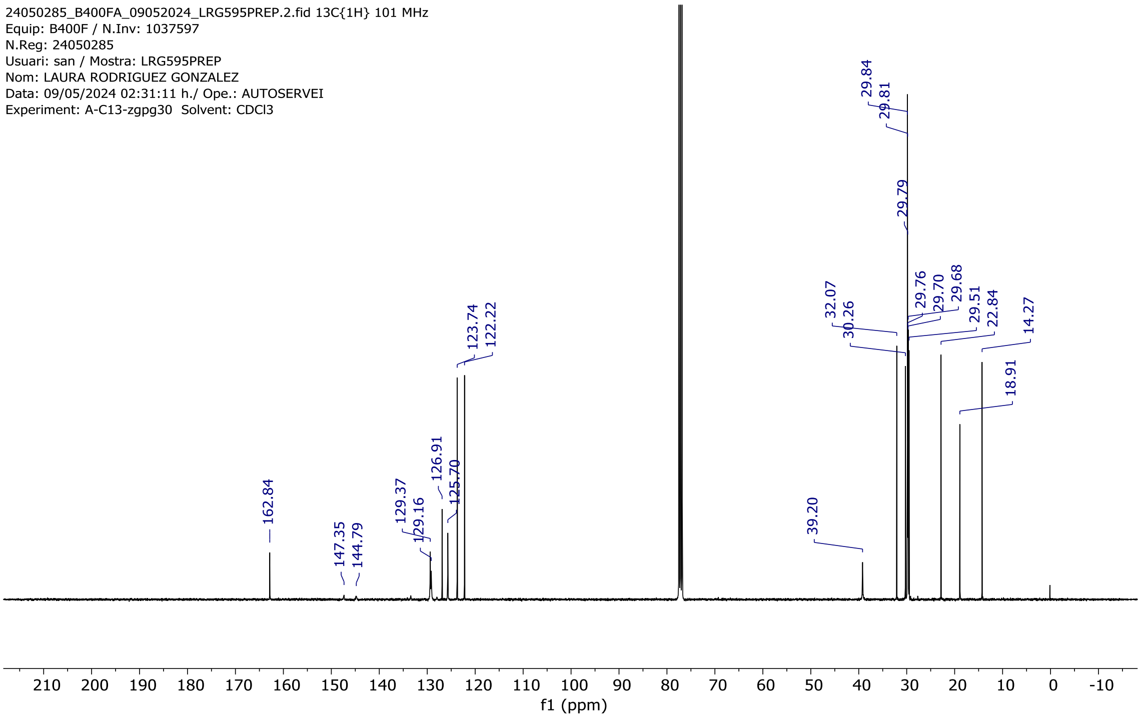

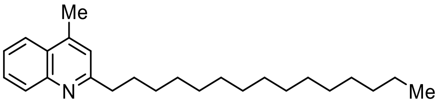

35

2D-COSY

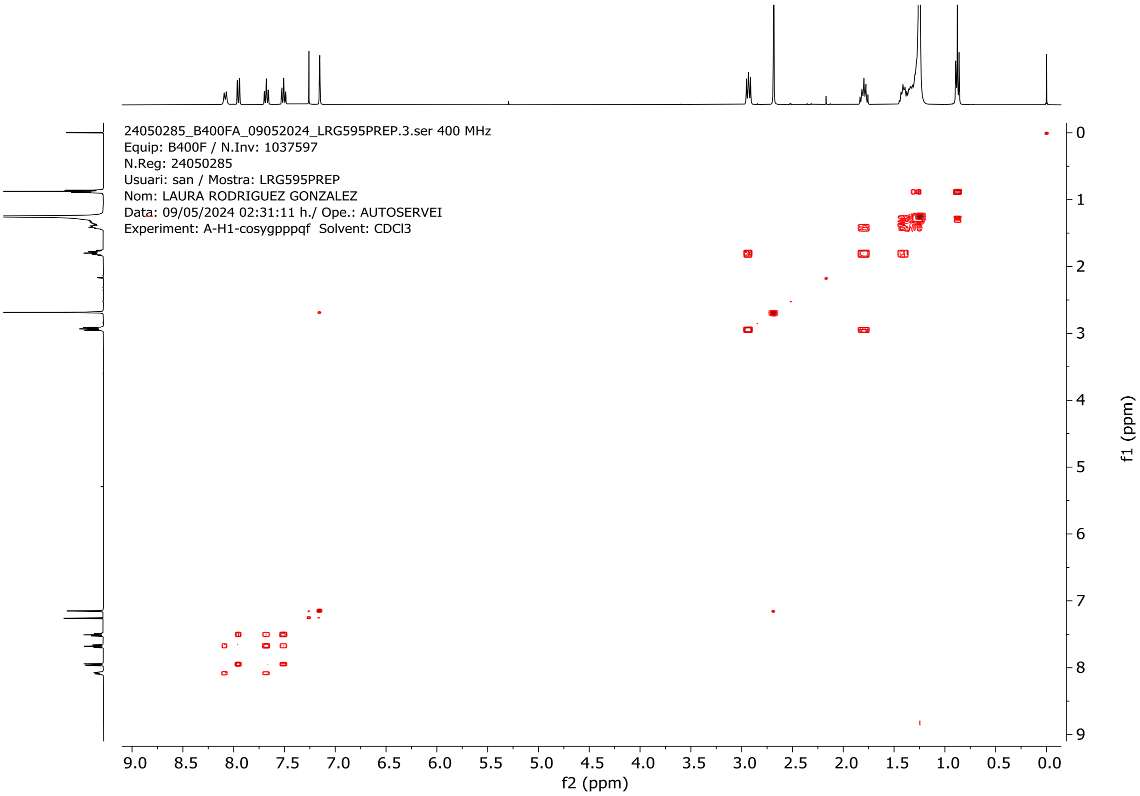

2D-HSQC

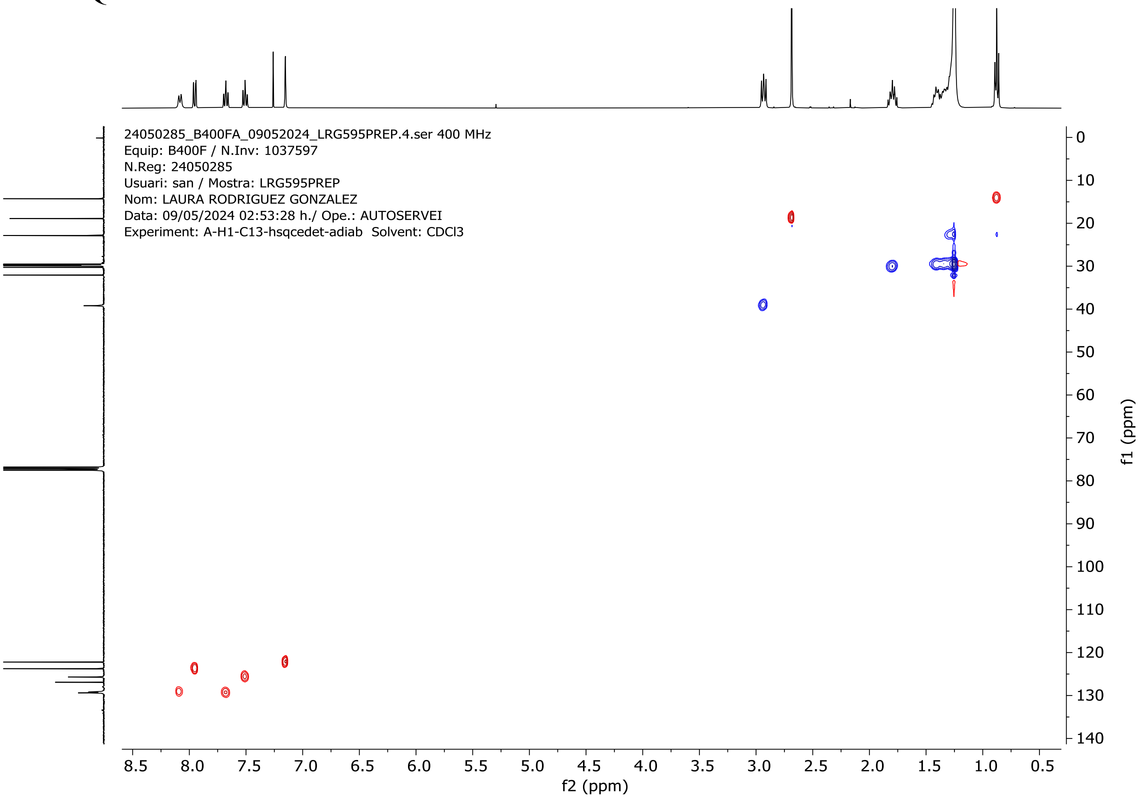

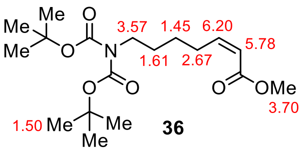
3.57 1.45 6.20
5.78
1.61 2.67
3.70
1.50
OMe

36

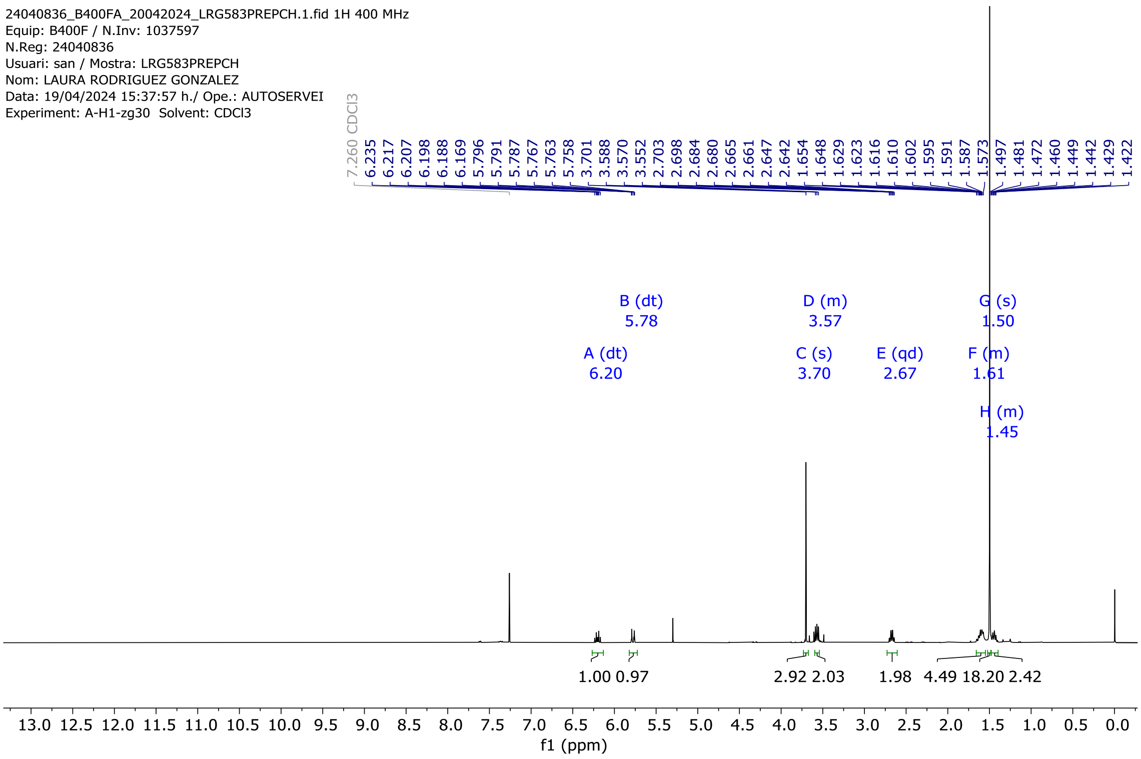
24040836_B400FA_20042024_LRG583PREPCH.1.fid 1H 400 MHz
Equip: B400F / N.Inv: 1037597
N.Reg: 24040836
Usuari: san / Mostra: LRG583PREPCH
Nom: LAURA RODRIGUEZ GONZALEZ
Data: 19/04/2024 15:37:57 h./ Ope.: AUTOSERVEI
Experiment: A-H1-zg30 Solvent: CDCl3
7.260 CDCl3
A (dt) 6.20
B (dt) 5.78
C (s) 3.70
D (m) 3.57
E (qd) 2.67
F (m) 1.61
G (s) 1.50
H (m) 1.45
1.00 0.97 2.92 2.03 1.98 4.49 18.20 2.42
f1 (ppm)

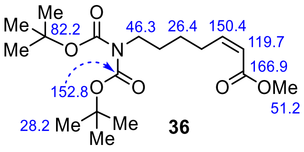
82.2 46.3 26.4 150.4
119.7
166.9
152.8
OMe
51.2
28.2

36

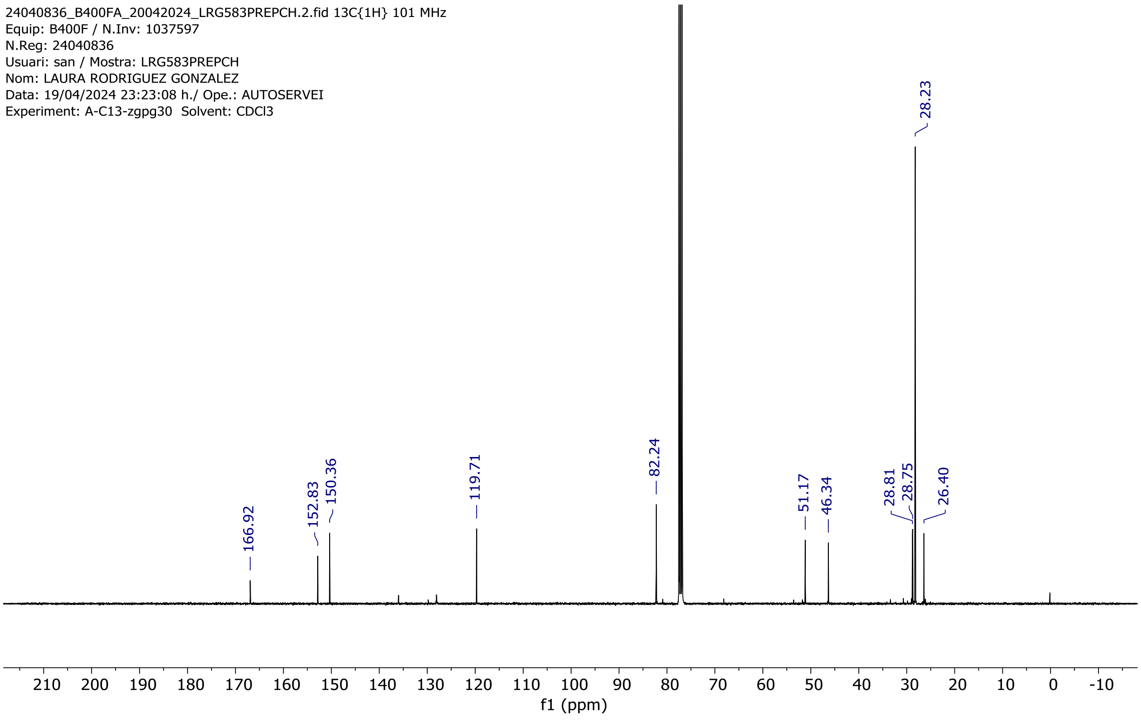
24040836_B400FA_20042024_LRG583PREPCH.2.fid 13C{1H} 101 MHz
Equip: B400F / N.Inv: 1037597
N.Reg: 24040836
Usuari: san / Mostra: LRG583PREPCH
Nom: LAURA RODRIGUEZ GONZALEZ
Data: 19/04/2024 23:23:08 h./ Ope.: AUTOSERVEI
Experiment: A-C13-zgpg30 Solvent: CDCl3
166.92
152.83
150.36
119.71
82.24
51.17
46.34
28.81
28.75
28.23
26.40
f1 (ppm)

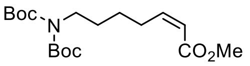

36

2D-COSY

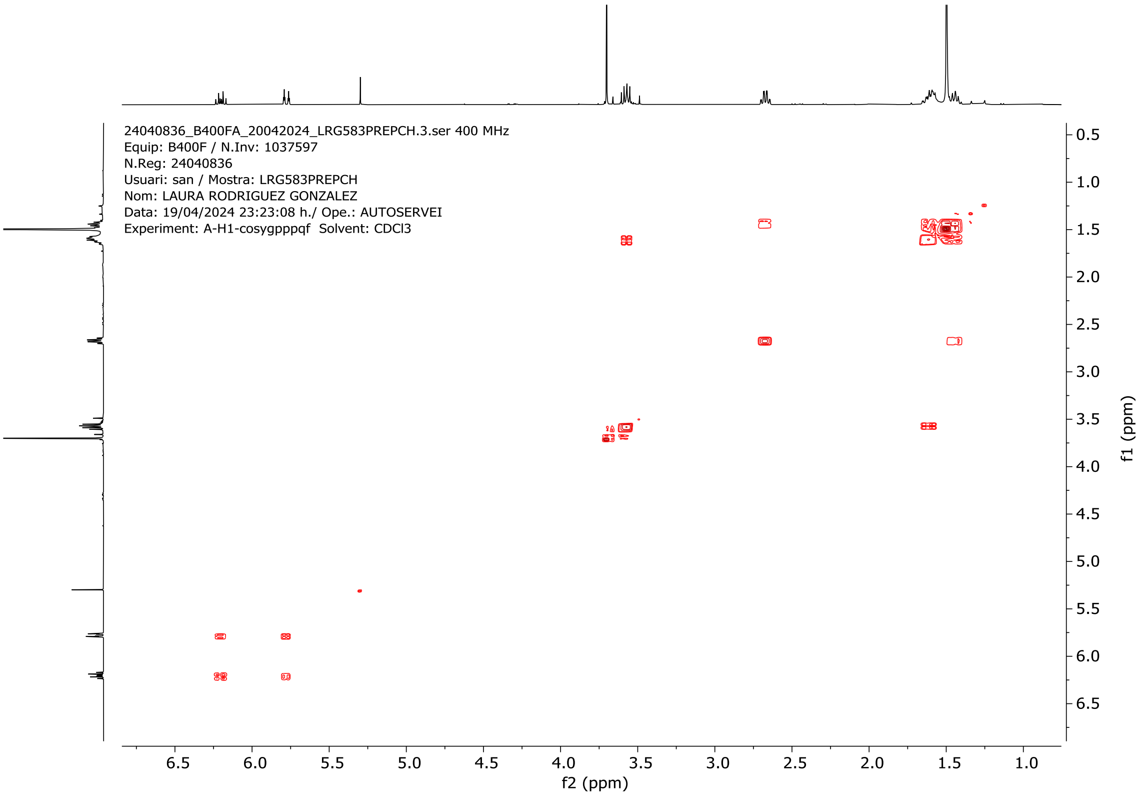

2D-HSQC

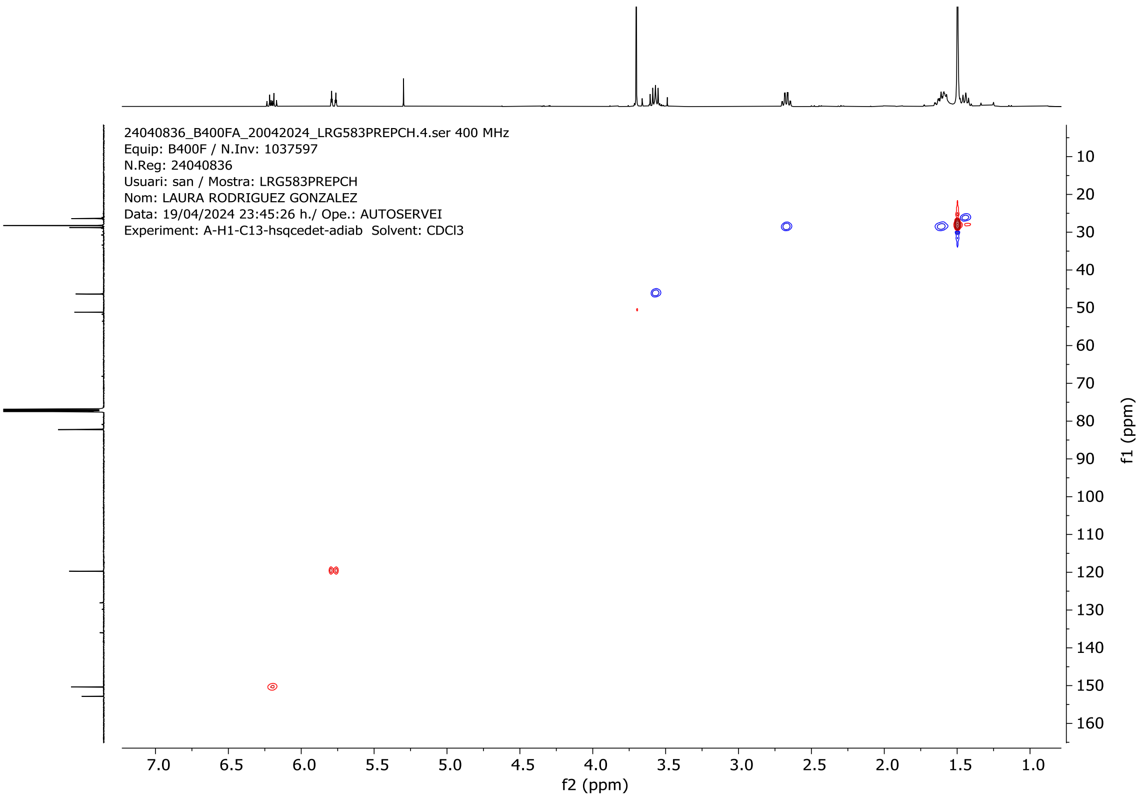

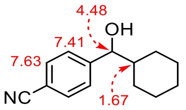

37

24050197_B400FA_07052024_LRGMS485PREP.1.fid 1H 400 MHz
Equip: B400F / N.Inv: 1037597
N.Reg: 24050197
Usuari: san / Mostra: LRGMS485PREP
Nom: LAURA RODRIGUEZ GONZALEZ
Data: 07/05/2024 12:08:07 h./ Ope.: AUTOSERVEI
Experiment: A-H1-zg30 Solvent: CDCl3

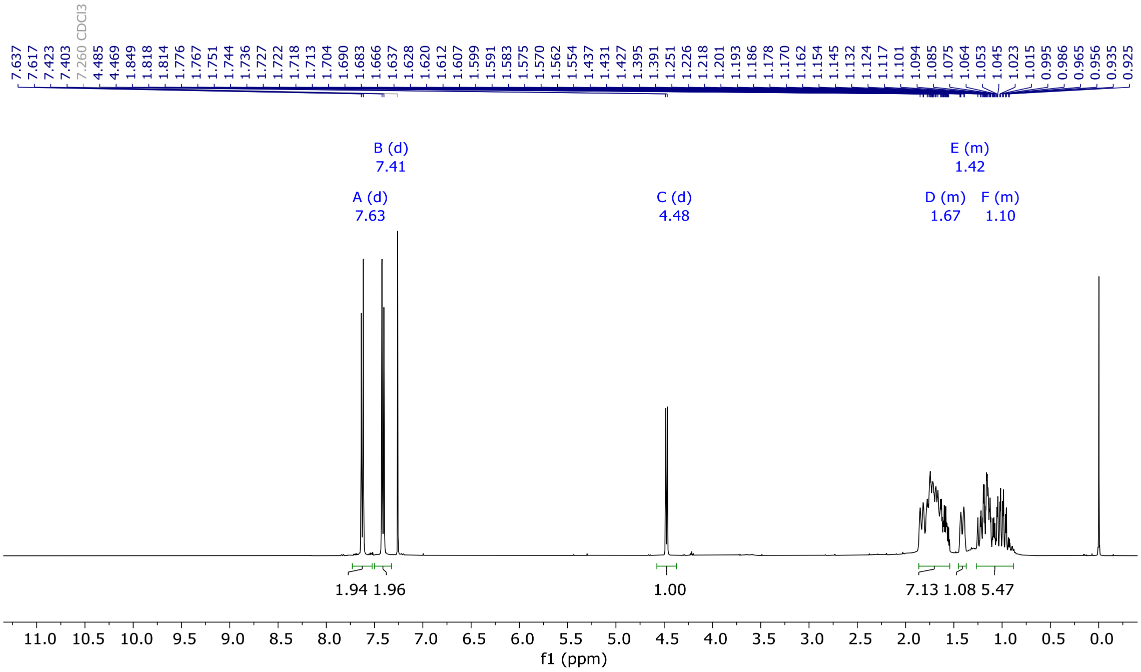

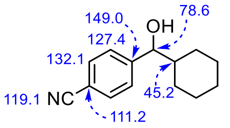

37

24050197_B400FA_07052024_LRGMS485PREP.2.fid 13C{1H} 101 MHz
Equip: B400F / N.Inv: 1037597
N.Reg: 24050197
Usuari: san / Mostra: LRGMS485PREP
Nom: LAURA RODRIGUEZ GONZALEZ
Data: 07/05/2024 22:29:47 h./ Ope.: AUTOSERVEI
Experiment: A-C13-zgpg30 Solvent: CDCl3

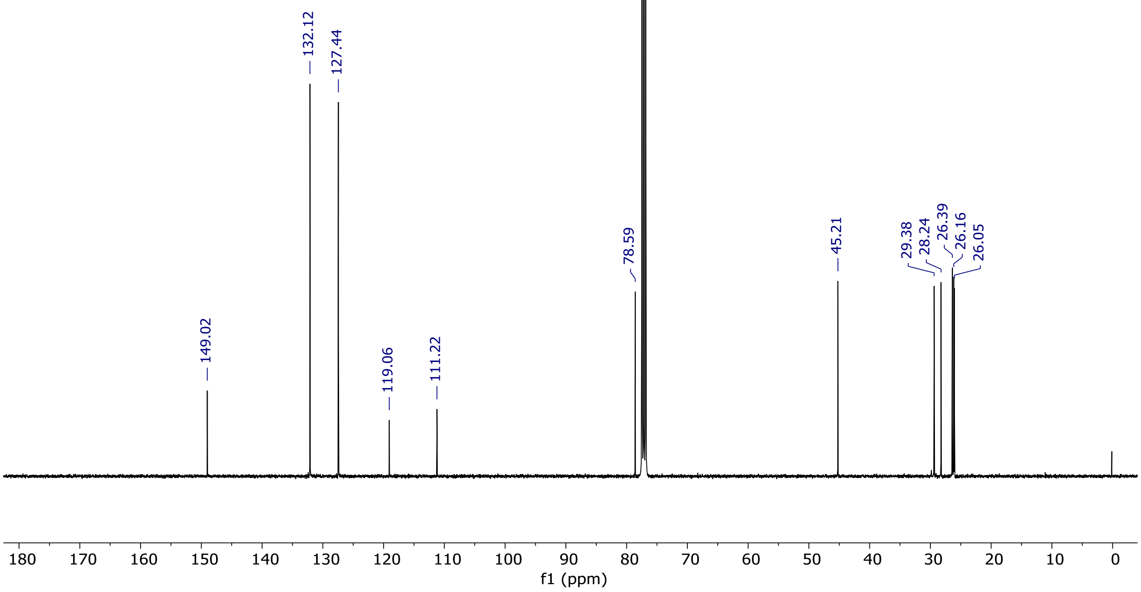

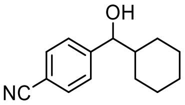

37

2D-COSY

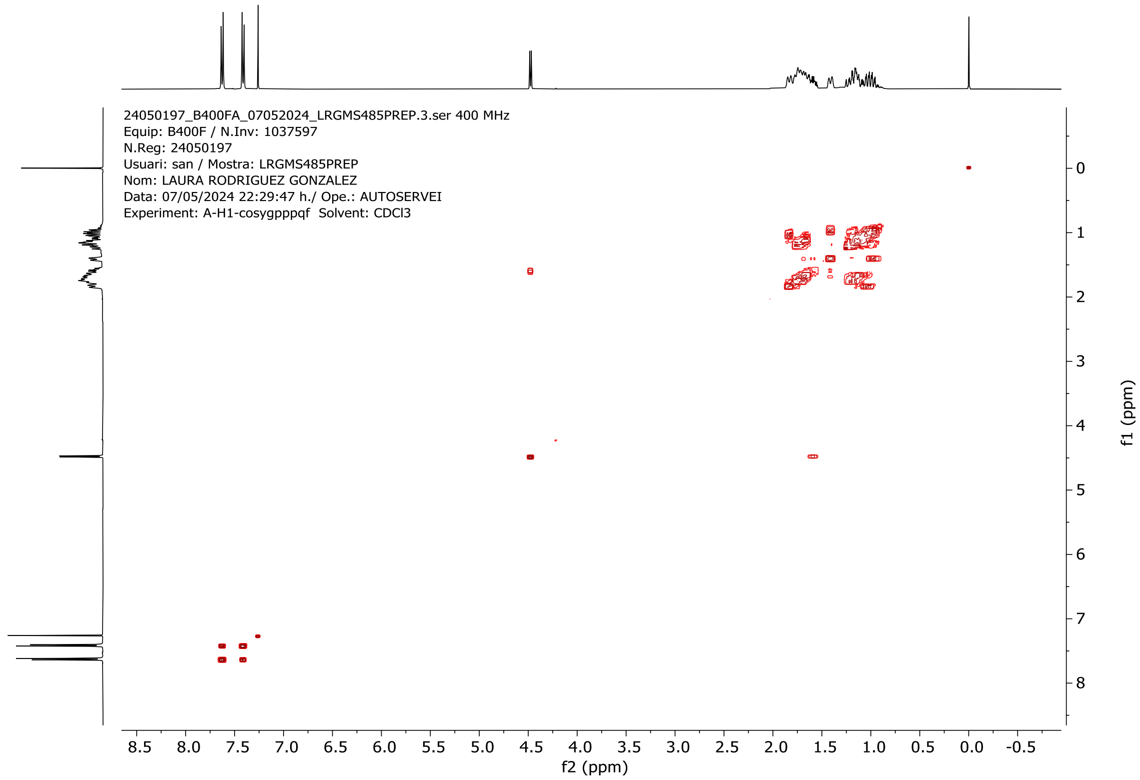

2D-HSQC

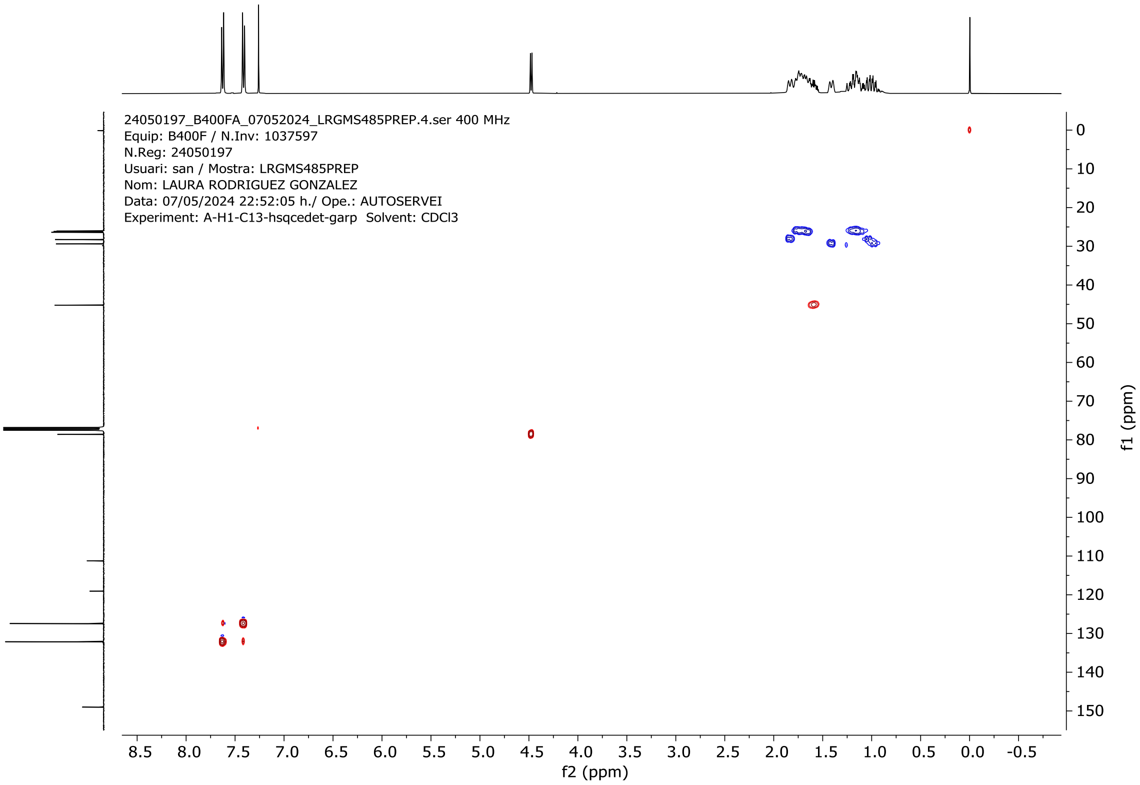

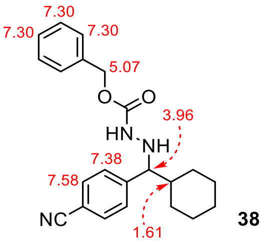

38

24050149_B400FA_06052024_LRG594DRY.1.fid 1H 400 MHz
Equip: B400F / N.Inv: 1037597
N.Reg: 24050149
Usuari: san / Mostra: LRG594DRY
Nom: LAURA RODRIGUEZ GONZALEZ
Data: 06/05/2024 14:46:38 h./ Ope.: AUTOSERVEI
Experiment: A-H1-zg30 Solvent: CDCl3

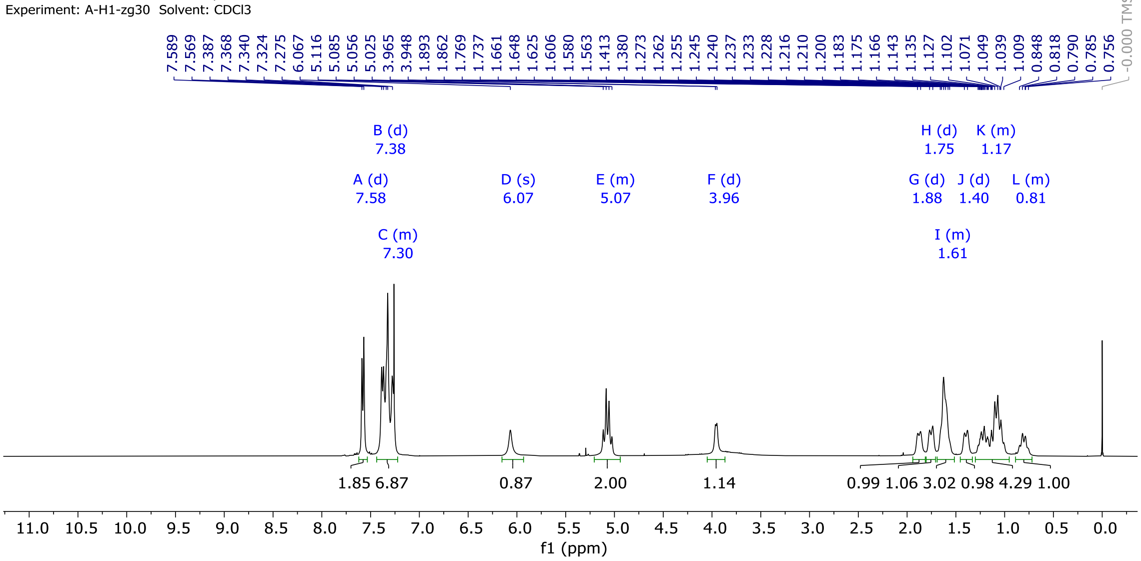

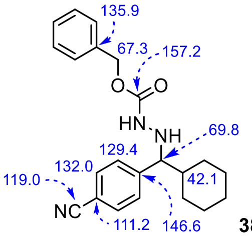

38

24050134_B400FA_06052024_LRG594COLT22T25.2.fid 13C{1H} 101 MHz
Equip: B400F / N.Inv: 1037597
N.Reg: 24050134
Usuari: san / Mostra: LRG594COLT22T25
Nom: LAURA RODRIGUEZ GONZALEZ
Data: 06/05/2024 22:32:41 h./ Ope.: AUTOSERVEI
Experiment: A-C13-zgpg30 Solvent: CDCl3

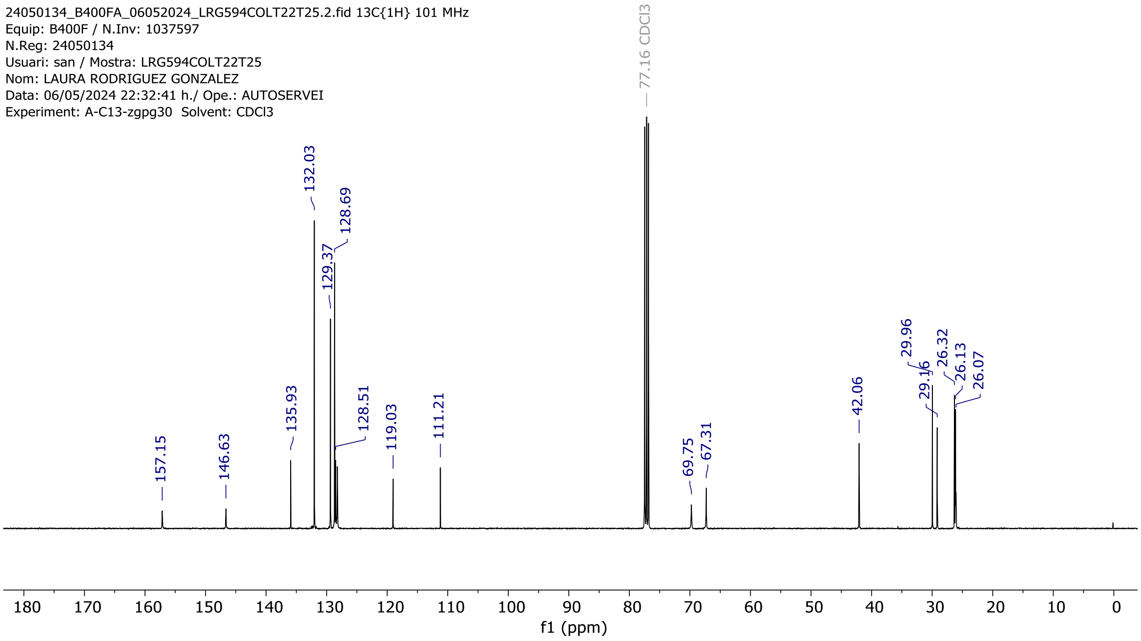

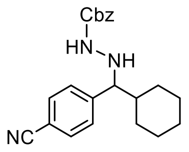

38

2D-COSY

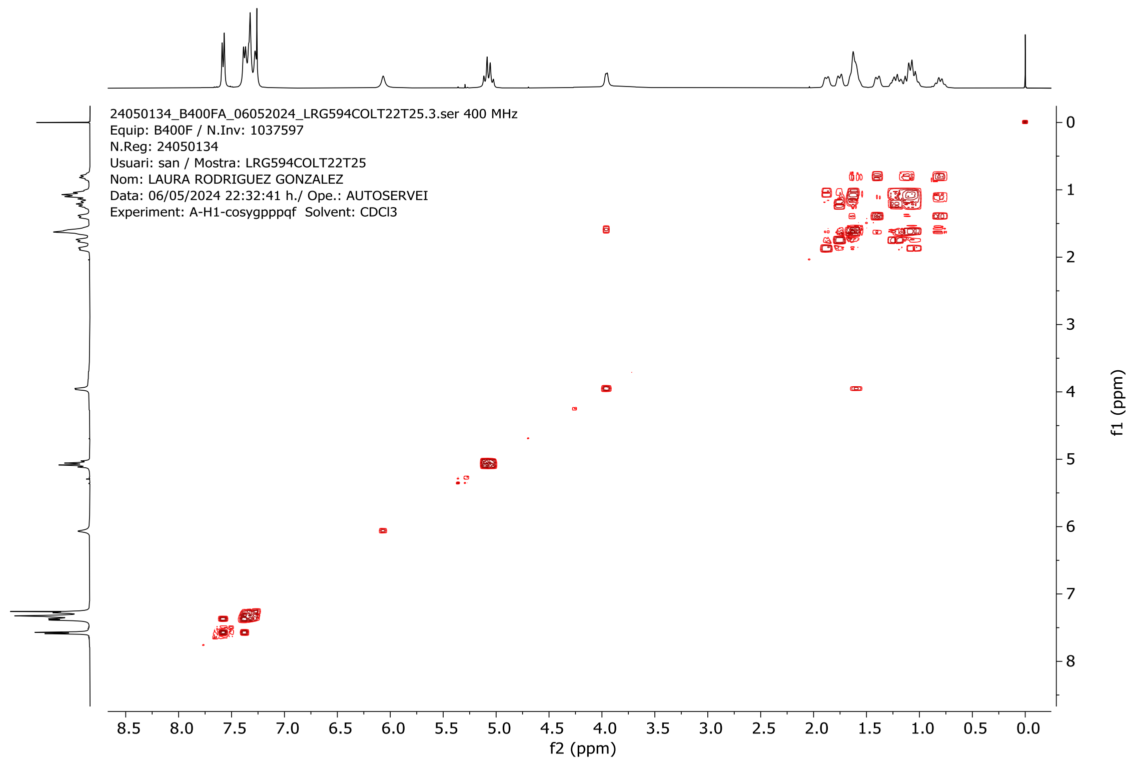

2D-HSQC

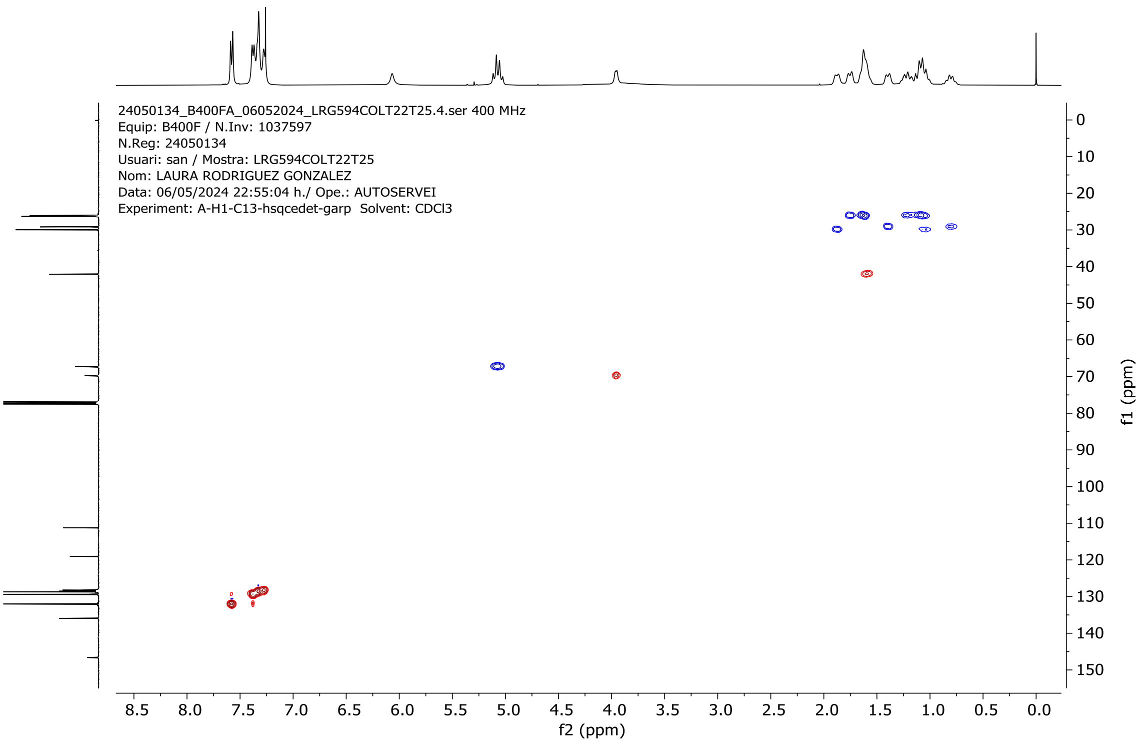

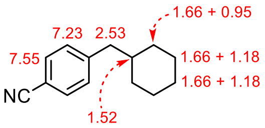

39

24050019_B400FA_02052024_LRG591PREP.1.fid 1H 400 MHz
Equip: B400F / N.Inv: 1037597
N.Reg: 24050019
Usuari: san / Mostra: LRG591PREP
Nom: LAURA RODRIGUEZ GONZALEZ
Data: 02/05/2024 13:45:58 h./ Ope.: AUTOSERVEI
Experiment: A-H1-zg30 Solvent: CDCl3

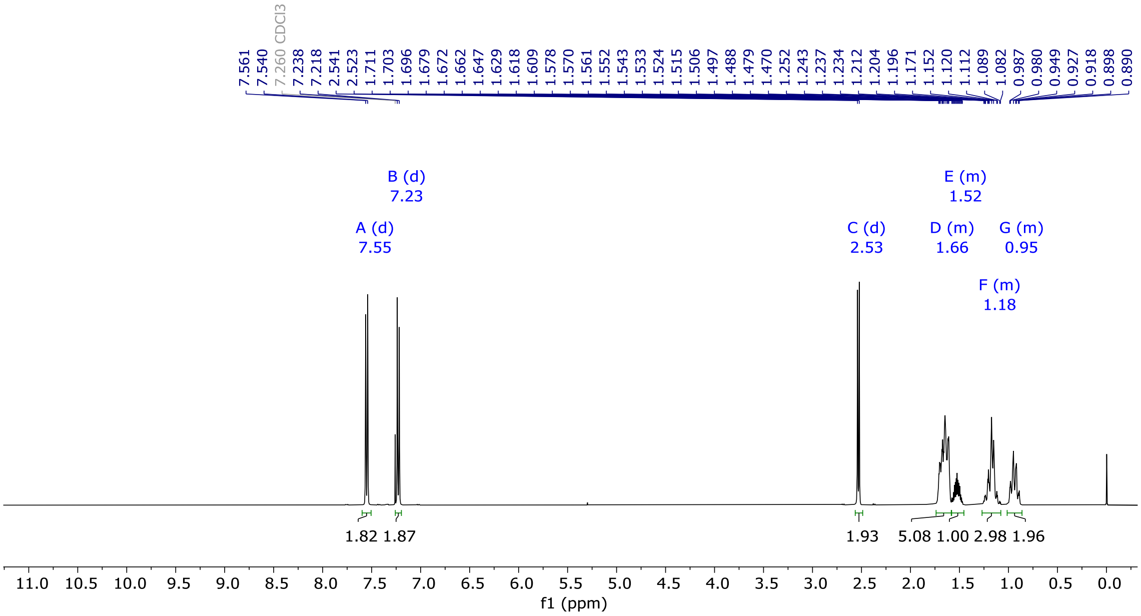

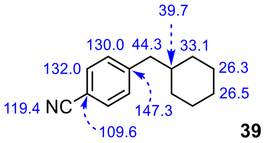

39

24050019_B400FA_03052024_LRG591PREP.2.fid 13C{1H} 101 MHz
Equip: B400F / N.Inv: 1037597
N.Reg: 24050019
Usuari: san / Mostra: LRG591PREP
Nom: LAURA RODRIGUEZ GONZALEZ
Data: 03/05/2024 03:52:20 h./ Ope.: AUTOSERVEI
Experiment: A-C13-zgpg30 Solvent: CDCl3

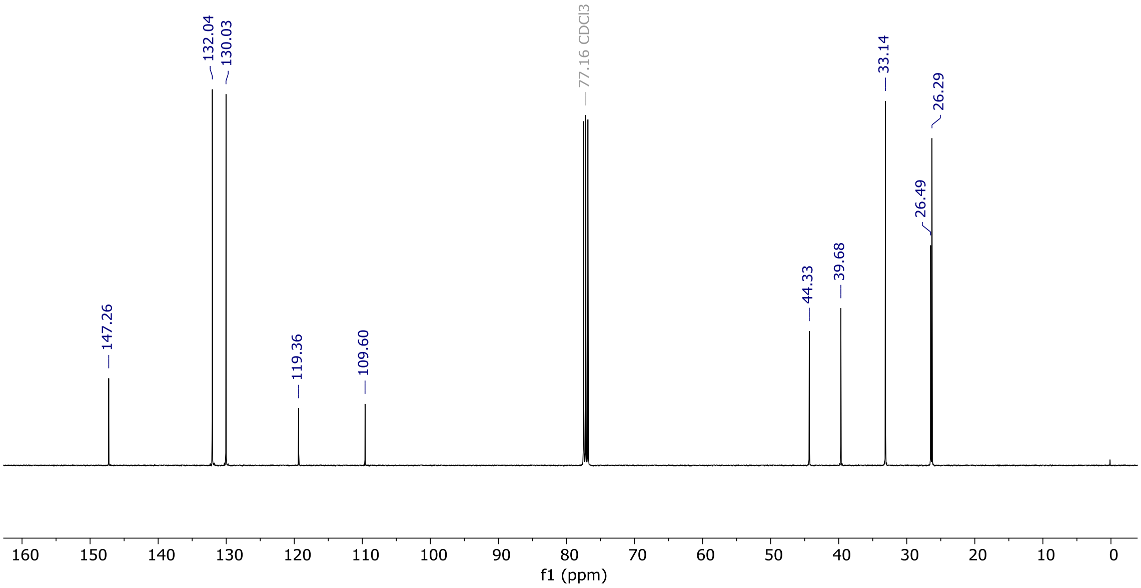

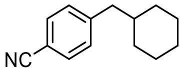

39

2D-COSY

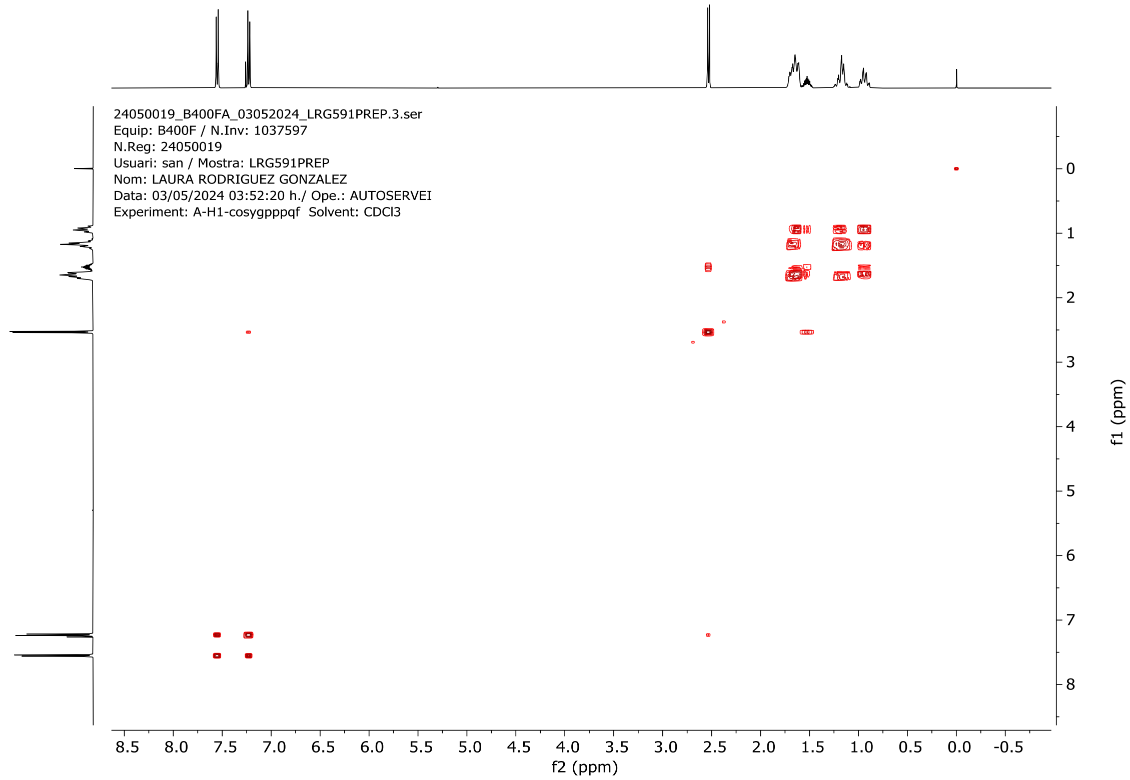

2D-HSQC

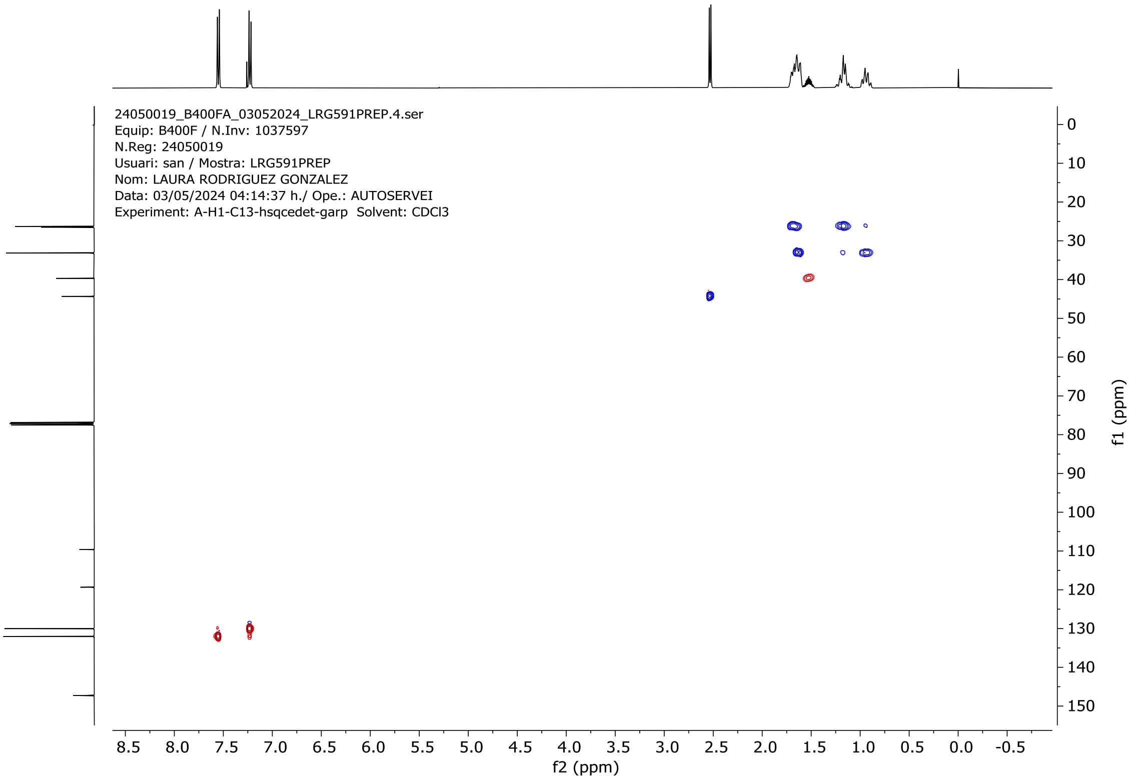

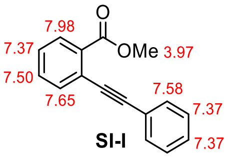

24010559_B400FA_26012024_LRG526COLT17T22.1.fid 1H 400 MHz
Equip: B400F / N.Inv: 1037597
N.Reg: 24010559
Usuari: san / Mostra: LRG526COLT17T22
Nom: LAURA RODRIGUEZ GONZALEZ
Data: 25/01/2024 15:14:54 h./ Ope.: AUTOSERVEI
Experiment: A-H1-zg30 Solvent: CDCl3

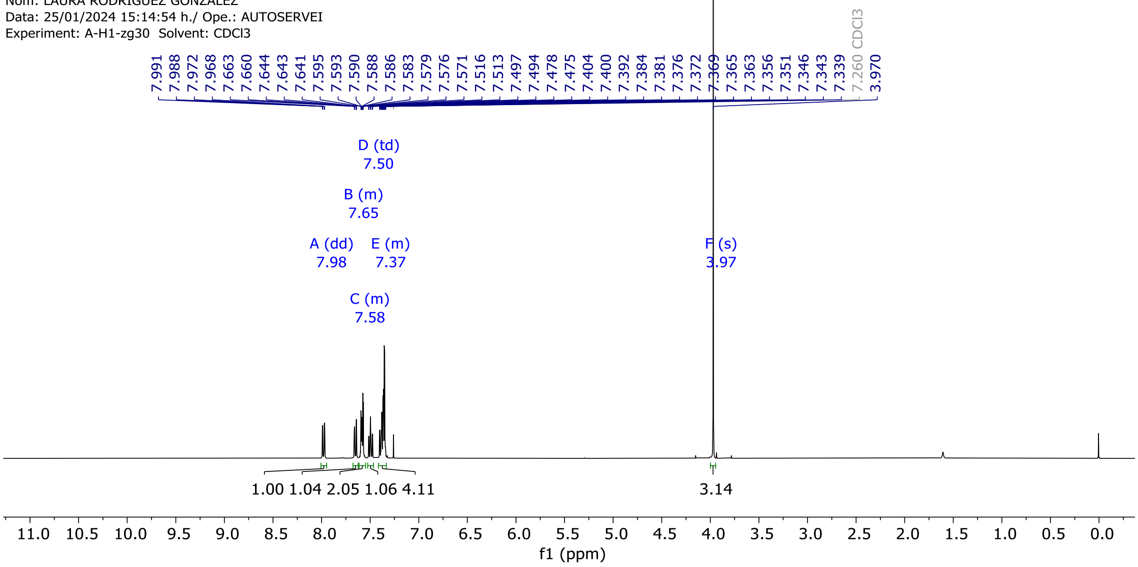

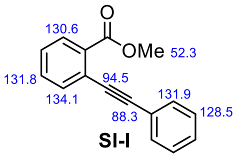

24010559_B400FA_26012024_LRG526COLT17T22.2.fid 13C{1H} 101 MHz
Equip: B400F / N.Inv: 1037597
N.Reg: 24010559
Usuari: san / Mostra: LRG526COLT17T22
Nom: LAURA RODRIGUEZ GONZALEZ
Data: 26/01/2024 01:05:00 h./ Ope.: AUTOSERVEI
Experiment: A-C13-zgpg30 Solvent: CDCl3

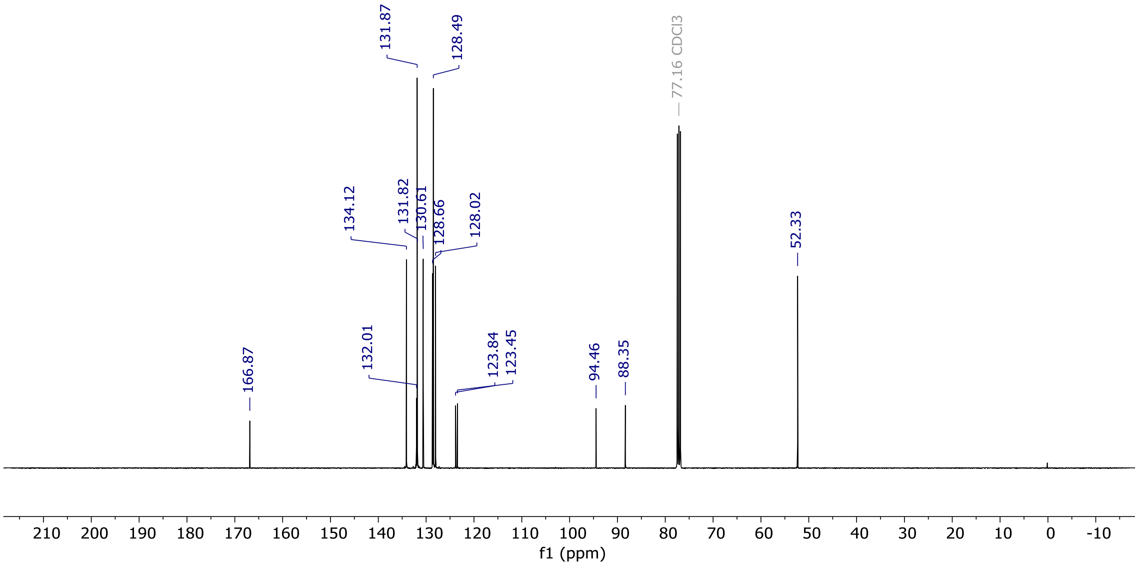

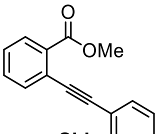

SI-I

2D-COSY

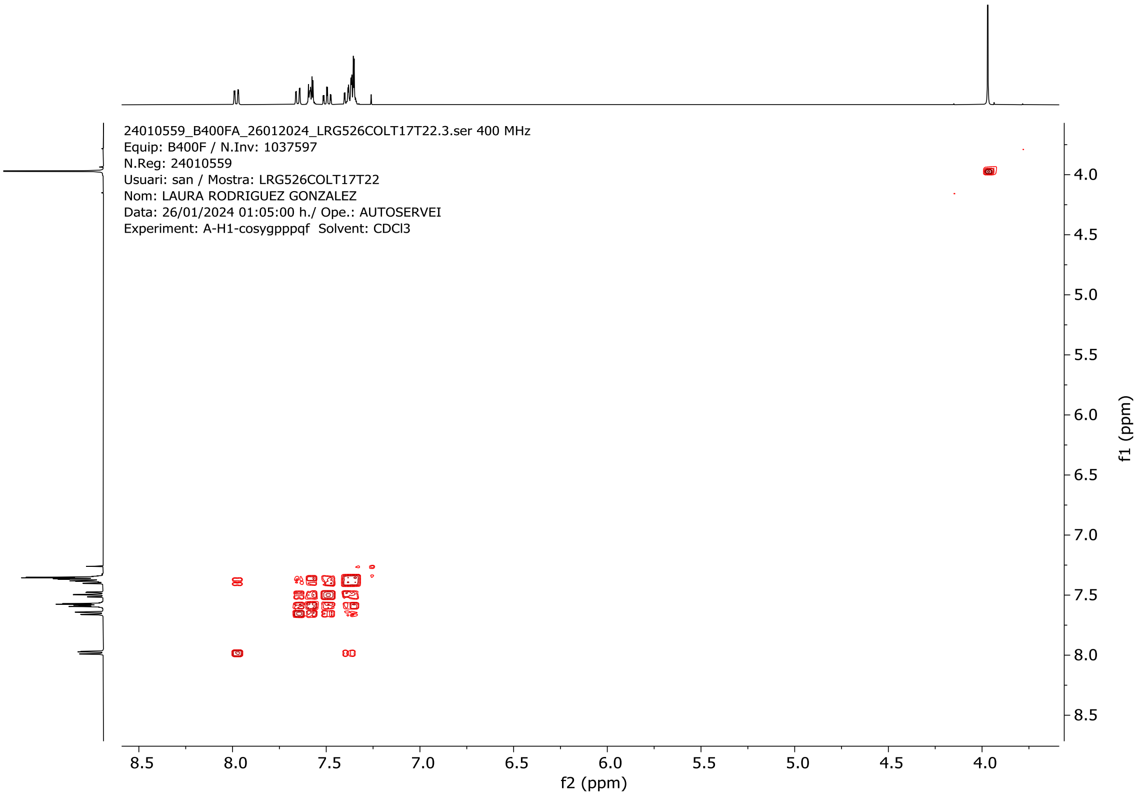

2D-HSQC

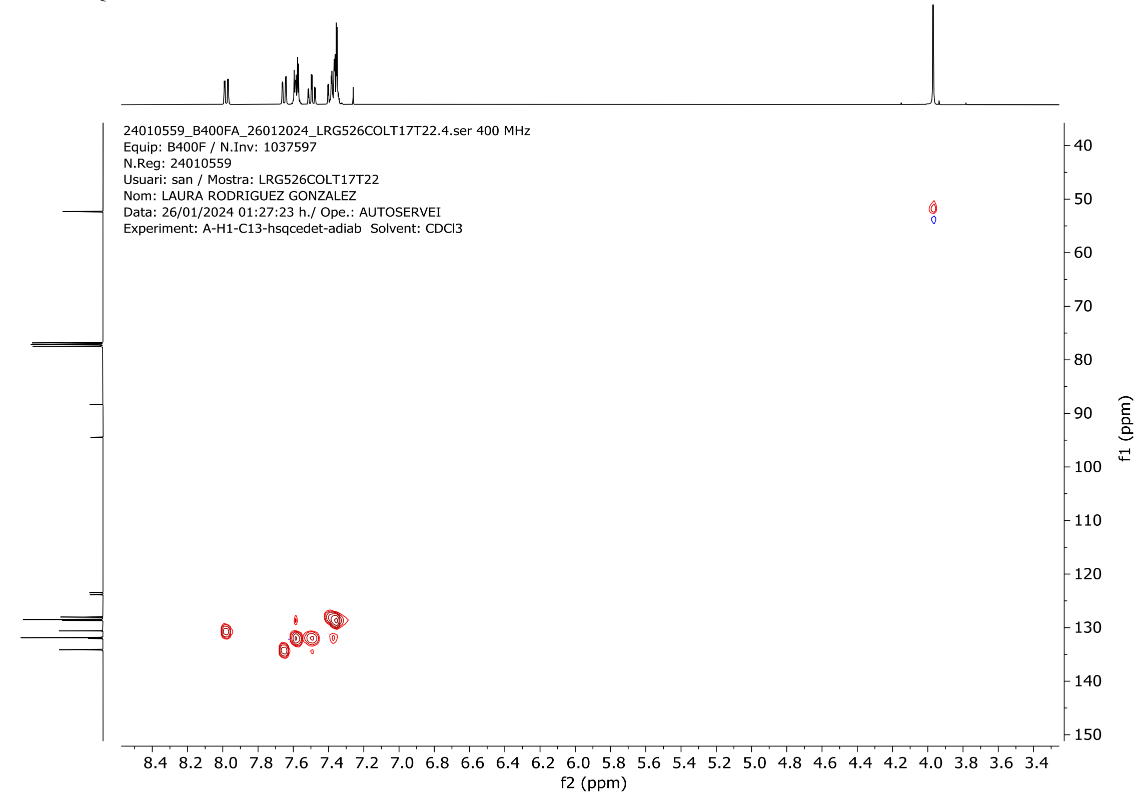

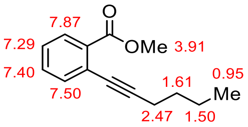

SI-II

24020175_B400FA_07022024_ASV002COLT11T13.1.fid 1H 400 MHz
Equip: B400F / N.Inv: 1037597
N.Reg: 24020175
Usuari: san / Mostra: ASV002COLT11T13
Nom: LAURA RODRIGUEZ GONZALEZ
Data: 07/02/2024 16:53:29 h./ Ope.: AUTOSERVEI
Experiment: A-H1-zg30 Solvent: CDCl3

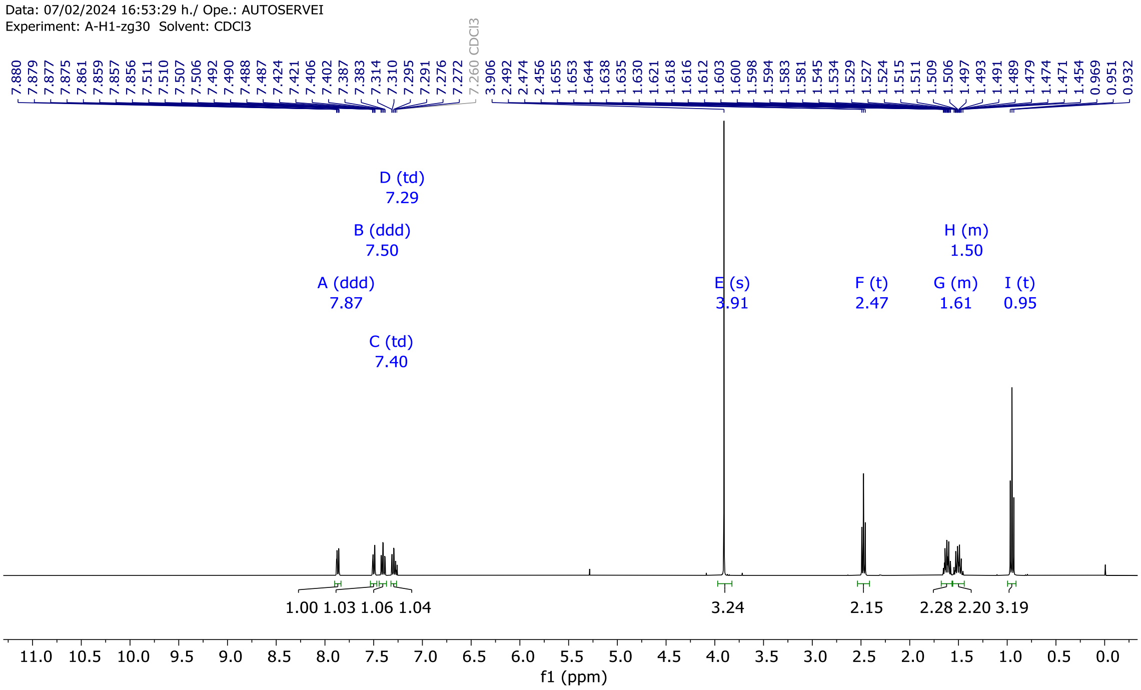

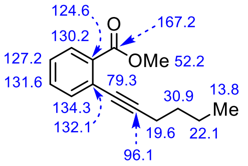

SI-II

ASV002CH.2.fid 13C{1H} 101 MHz
Equip: B400F / N.Inv: 1037597
N.Reg: 24020155
Usuari: san / Mostra: LRG536COLT5T15
Nom: LAURA RODRIGUEZ GONZALEZ
Data: 08/02/2024 00:24:58 h./ Ope.: AUTOSERVEI
Experiment: A-C13-zgpg30 Solvent: CDCl3

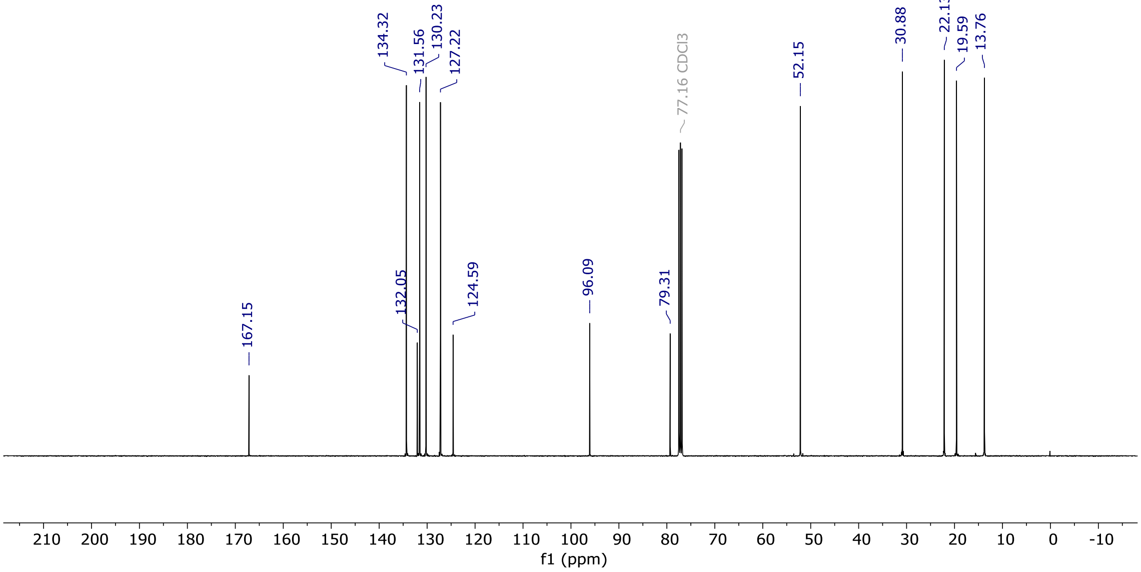

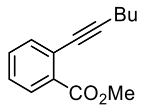

SI-II

2D-COSY

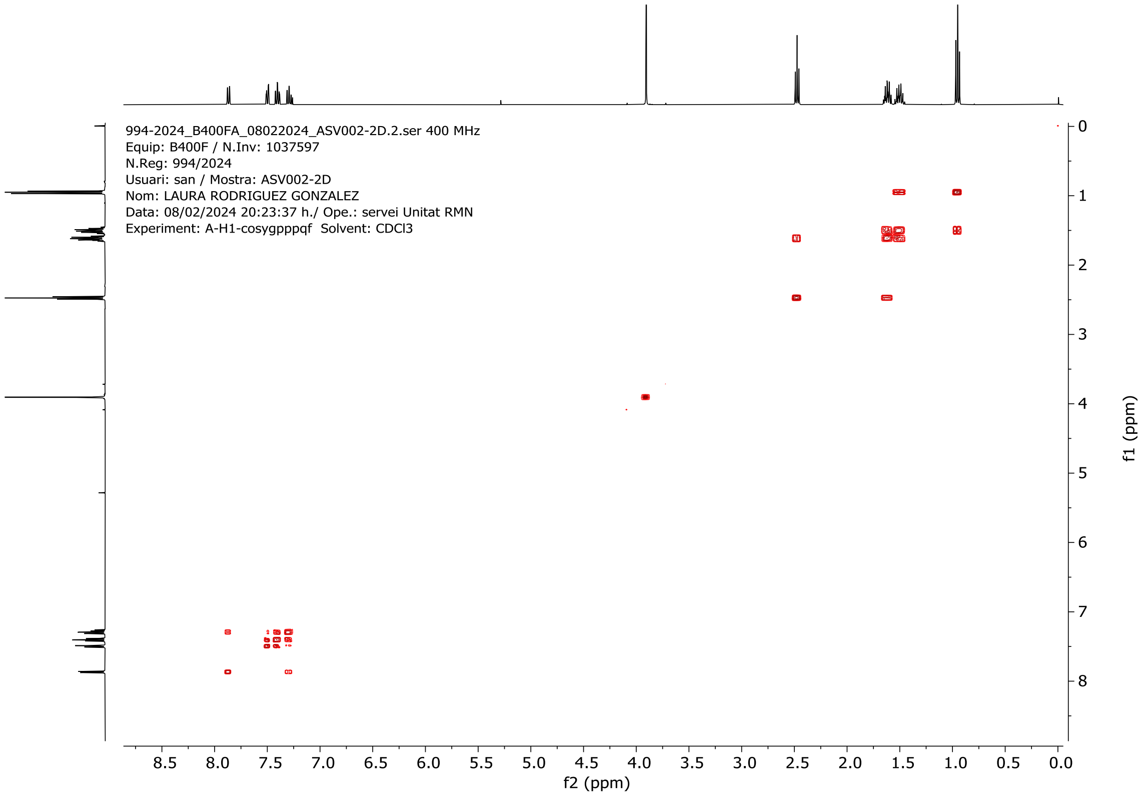

2D-HSQC

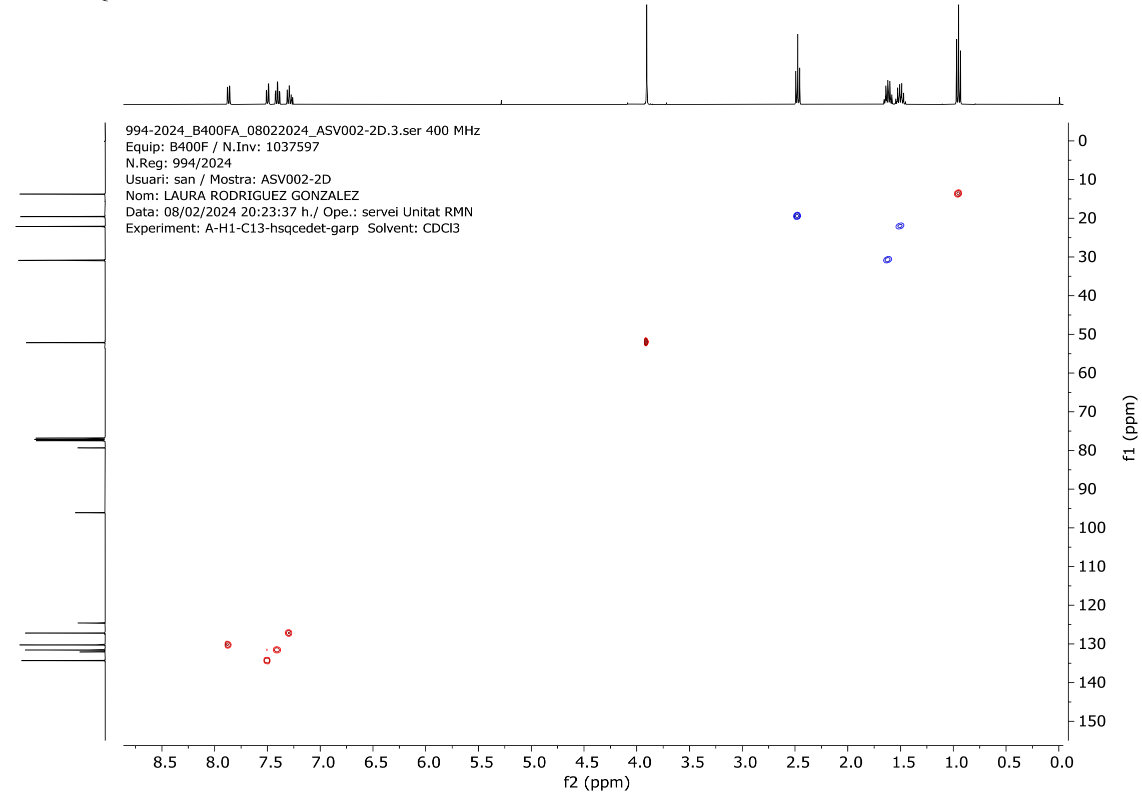

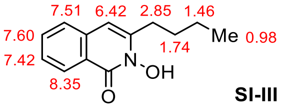

SI-III

24020279_B400FA_11022024_ASV004.1.fid 1H 400 MHz
Equip: B400F / N.Inv: 1037597
N.Reg: 24020279
Usuari: san / Mostra: ASV004
Nom: LAURA RODRIGUEZ GONZALEZ
Data: 09/02/2024 16:43:07 h./ Ope.: AUTOSERVEI
Experiment: A-H1-zg30 Solvent: CDCl3

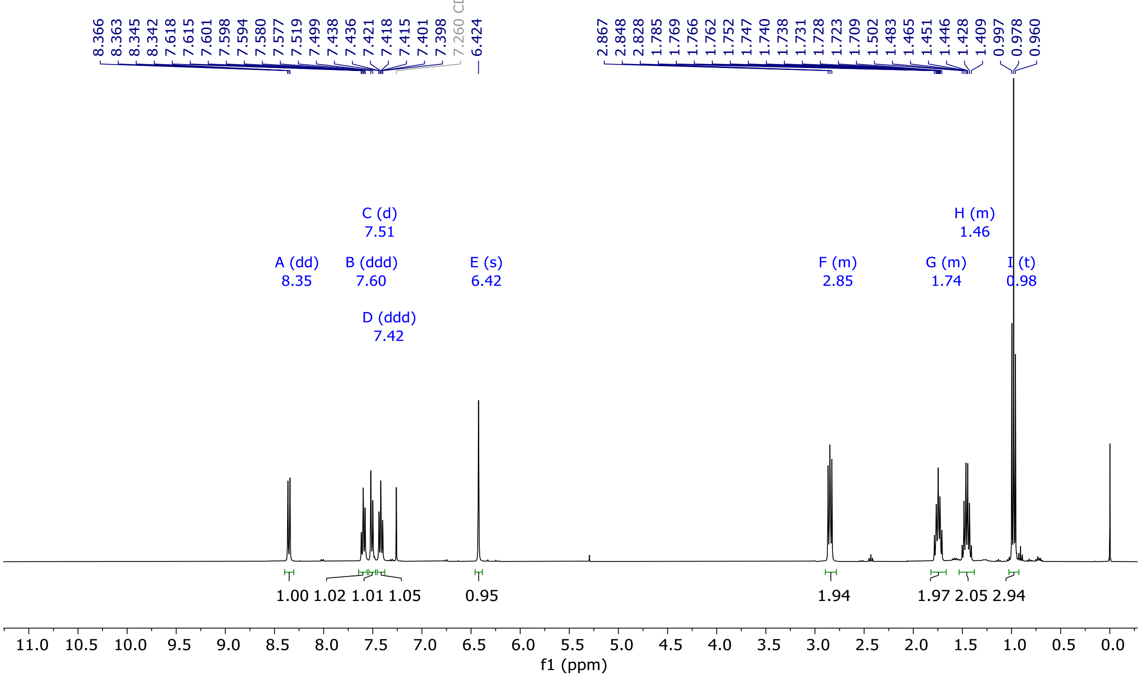

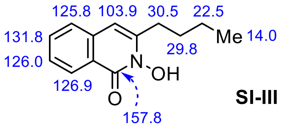

SI-III

24020279_B400FA_11022024_ASV004.2.fid 13C{1H} 101 MHz
Equip: B400F / N.Inv: 1037597
N.Reg: 24020279
Usuari: san / Mostra: ASV004
Nom: LAURA RODRIGUEZ GONZALEZ
Data: 11/02/2024 21:38:54 h./ Ope.: AUTOSERVEI
Experiment: A-C13-zgpg30 Solvent: CDCl3

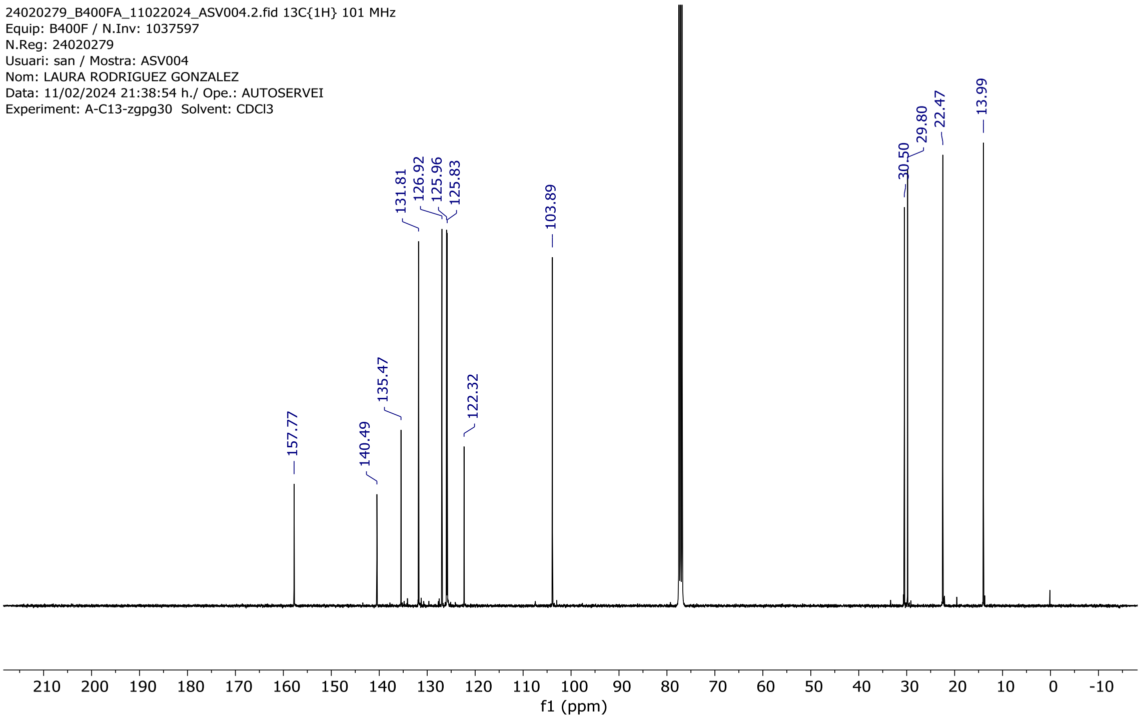

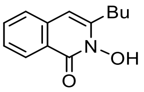

SI-III

2D-COSY

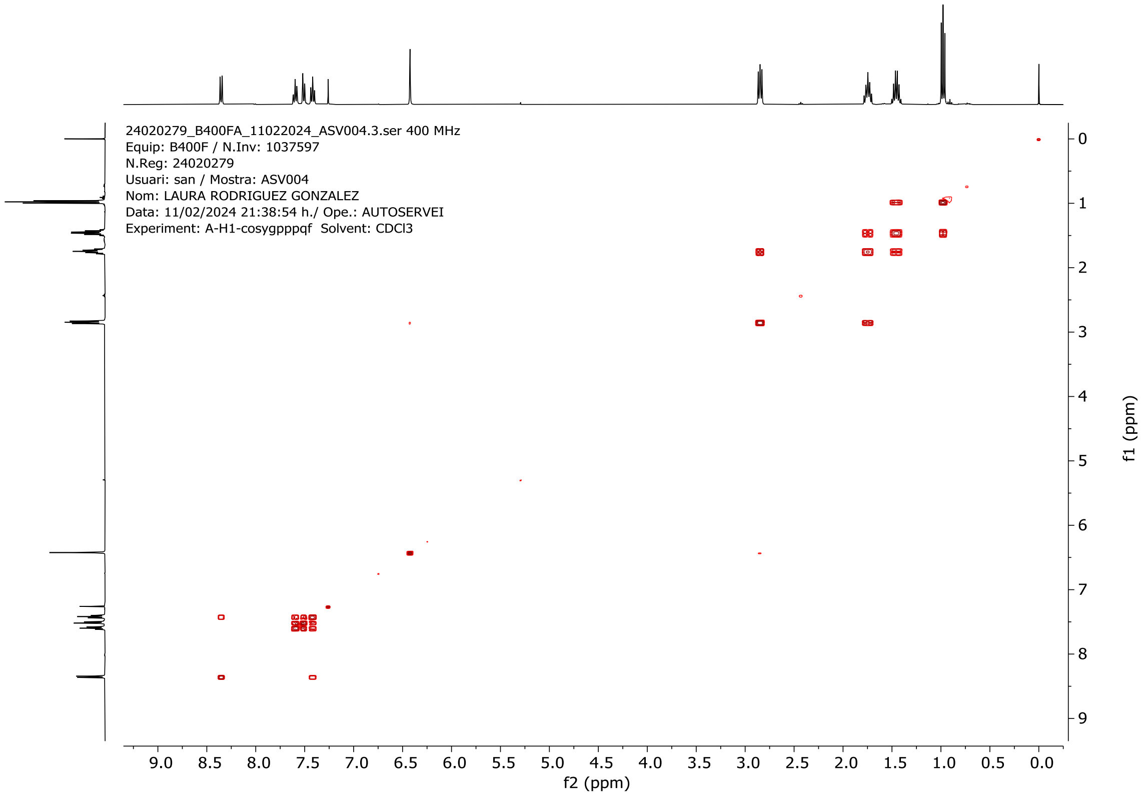

2D-HSQC

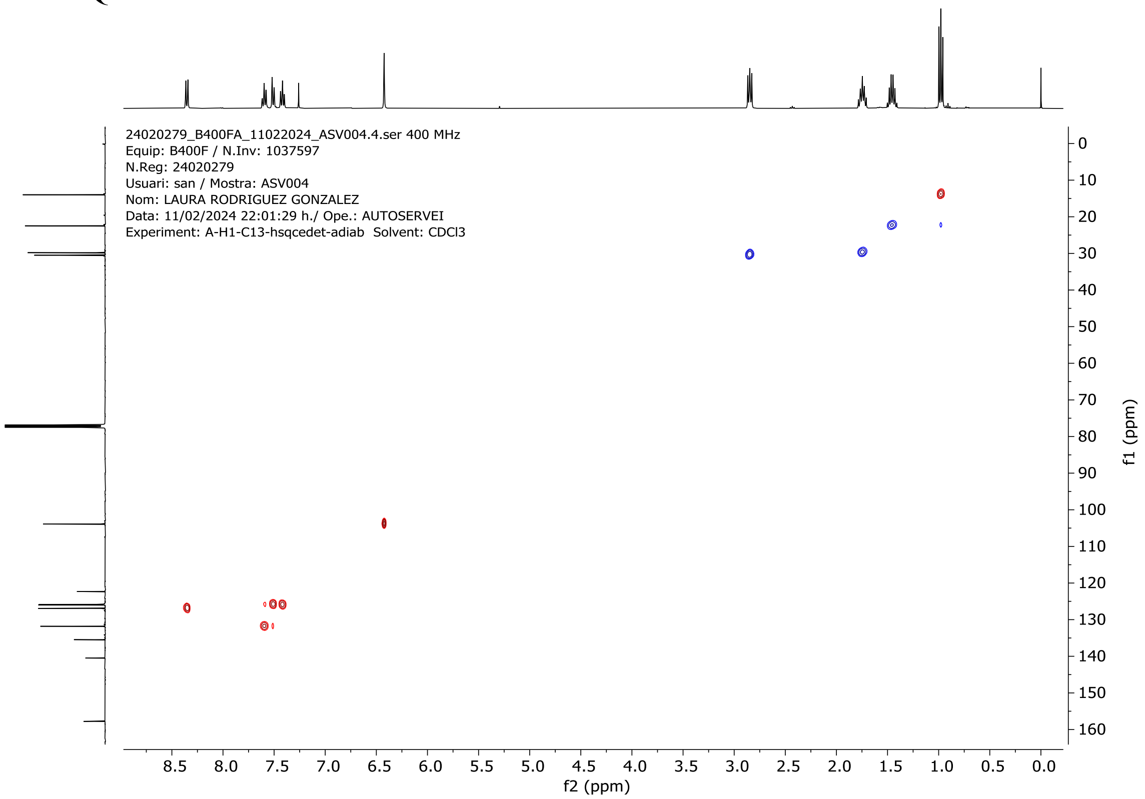

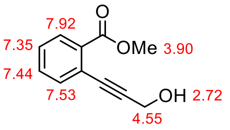

SI-IV

24010684_B400FA_30012024_LRG529COLT25T29.1.fid 1H 400 MHz
Equip: B400F / N.Inv: 1037597
N.Reg: 24010684
Usuari: san / Mostra: LRG529COLT25T29
Nom: LAURA RODRIGUEZ GONZALEZ
Data: 30/01/2024 13:13:15 h./ Ope.: AUTOSERVEI
Experiment: A-H1-zg30 Solvent: CDCl3

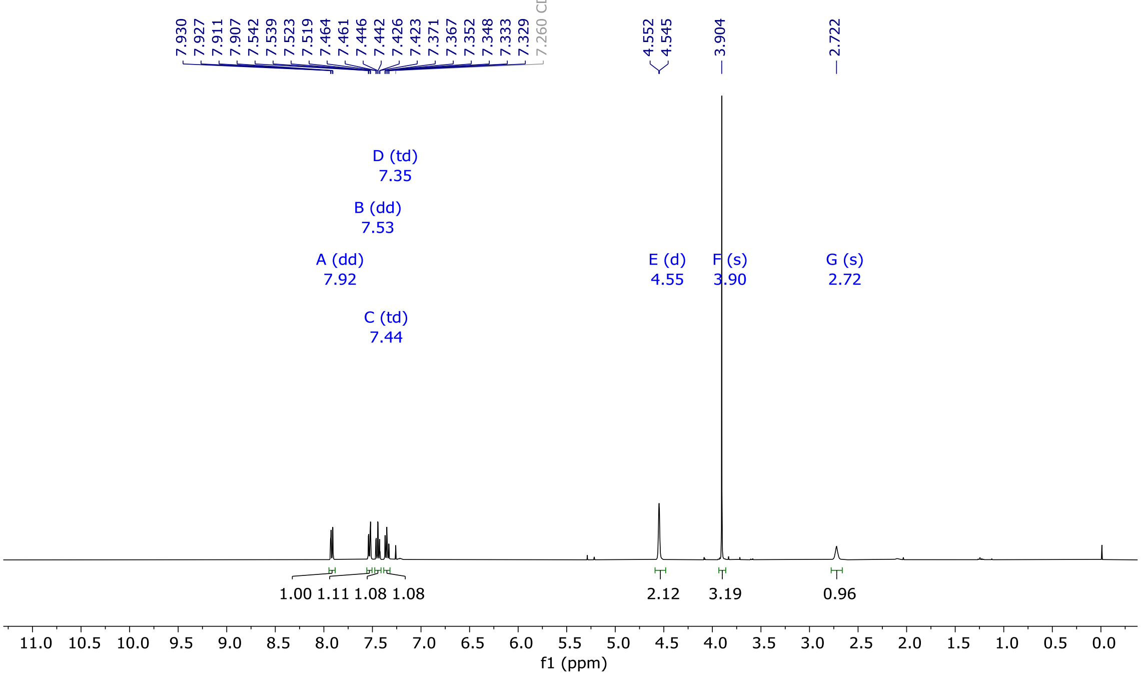

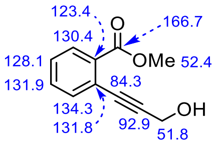

SI-IV

24010684_B400FA_30012024_LRG529COLT25T29.2.fid 13C{1H} 101 MHz
Equip: B400F / N.Inv: 1037597
N.Reg: 24010684
Usuari: san / Mostra: LRG529COLT25T29
Nom: LAURA RODRIGUEZ GONZALEZ
Data: 30/01/2024 21:07:24 h./ Ope.: AUTOSERVEI
Experiment: A-C13-zgpg30 Solvent: CDCl3

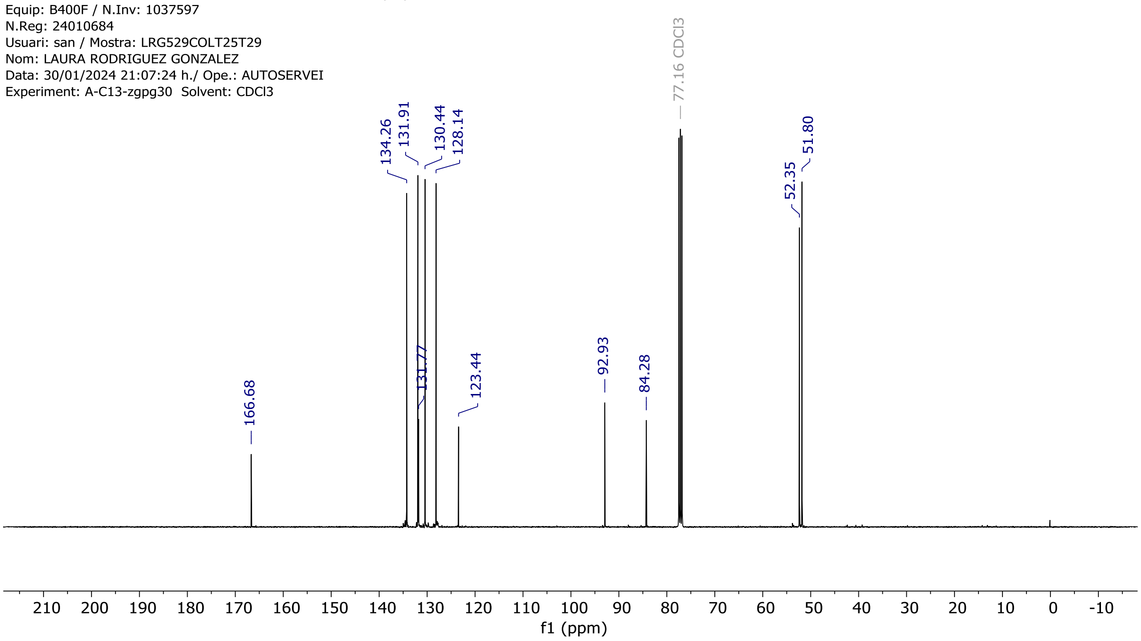

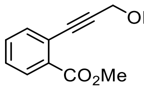

SI-IV

2D-COSY

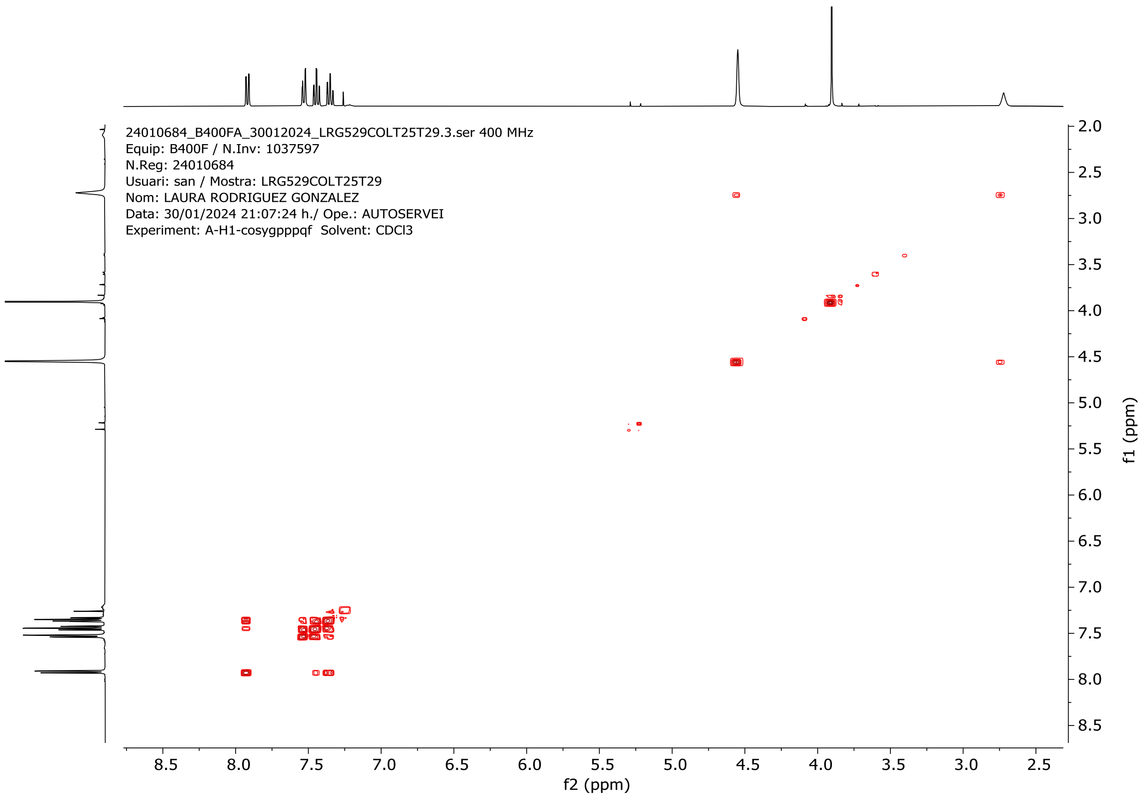

2D-HSQC

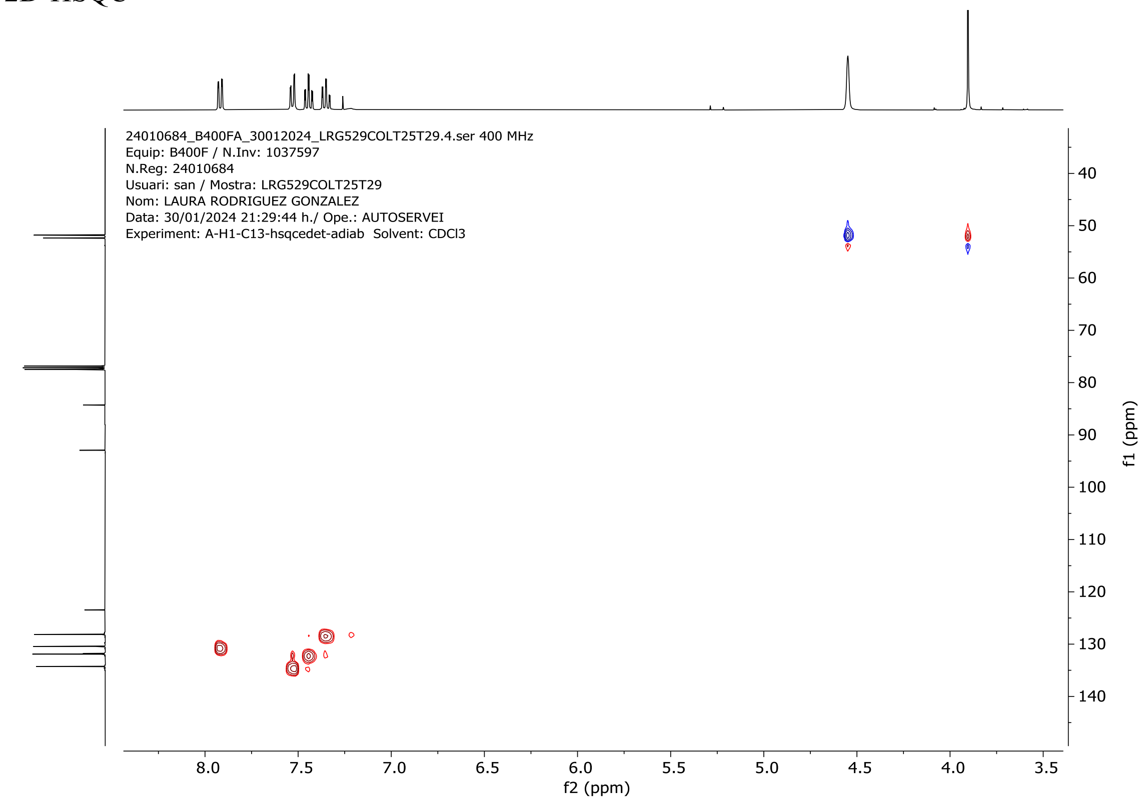

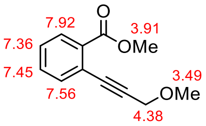

SI-V

24020193_B400FA_08022024_LRG537COLT12T15.1.fid 1H 400 MHz
Equip: B400F / N.Inv: 1037597
N.Reg: 24020193
Usuari: san / Mostra: LRG537COLT12T15
Nom: LAURA RODRIGUEZ GONZALEZ
Data: 08/02/2024 15:20:26 h./ Ope.: AUTOSERVEI
Experiment: A-H1-zg30 Solvent: CDCl3

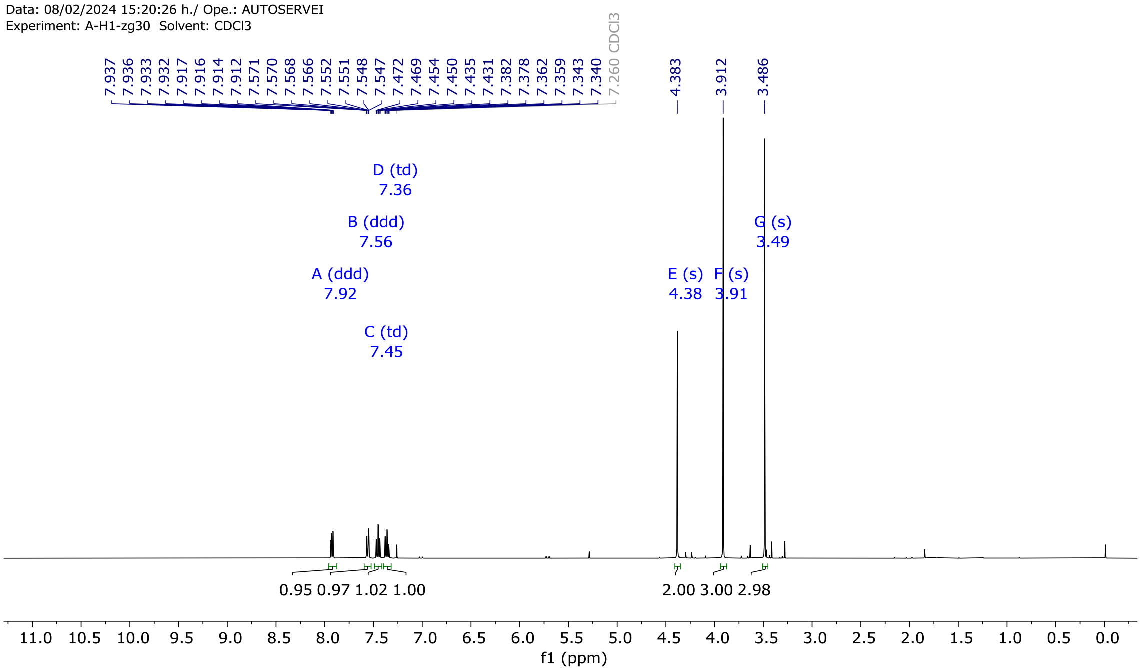

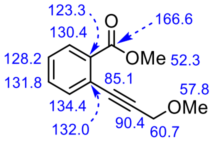

SI-V

24020193_B400FA_09022024_LRG537COLT12T15.2.fid 13C{1H} 101 MHz
Equip: B400F / N.Inv: 1037597
N.Reg: 24020193
Usuari: san / Mostra: LRG537COLT12T15
Nom: LAURA RODRIGUEZ GONZALEZ
Data: 09/02/2024 05:06:06 h./ Ope.: AUTOSERVEI
Experiment: A-C13-zgpg30 Solvent: CDCl3

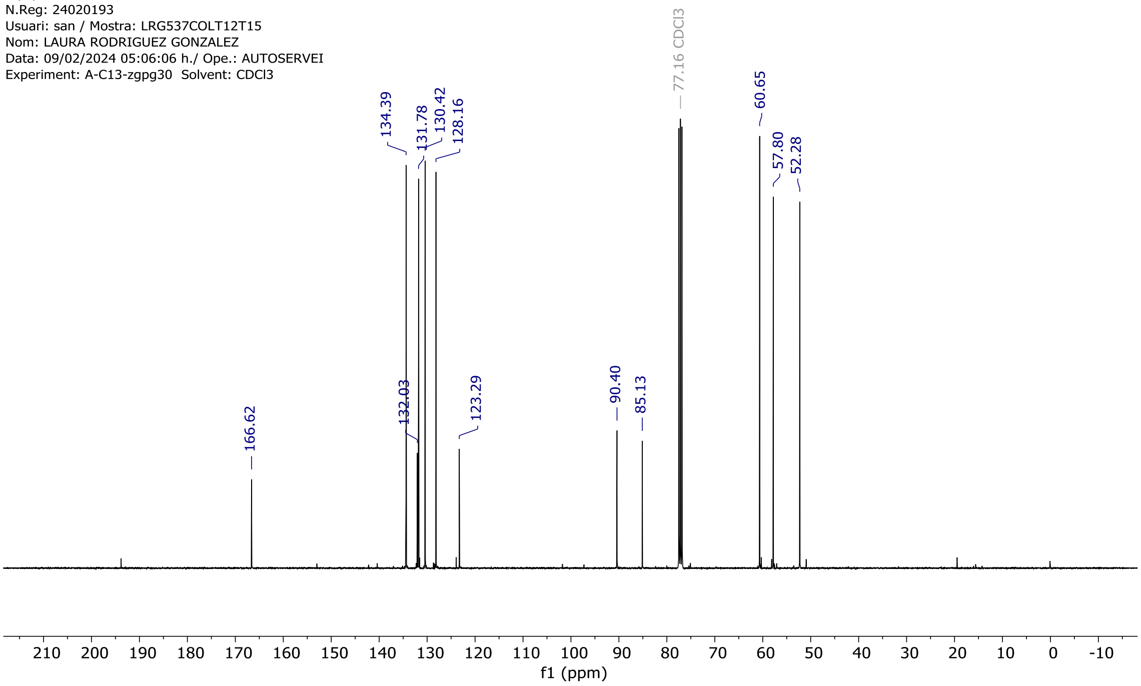

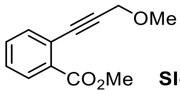

SI-V

2D-COSY

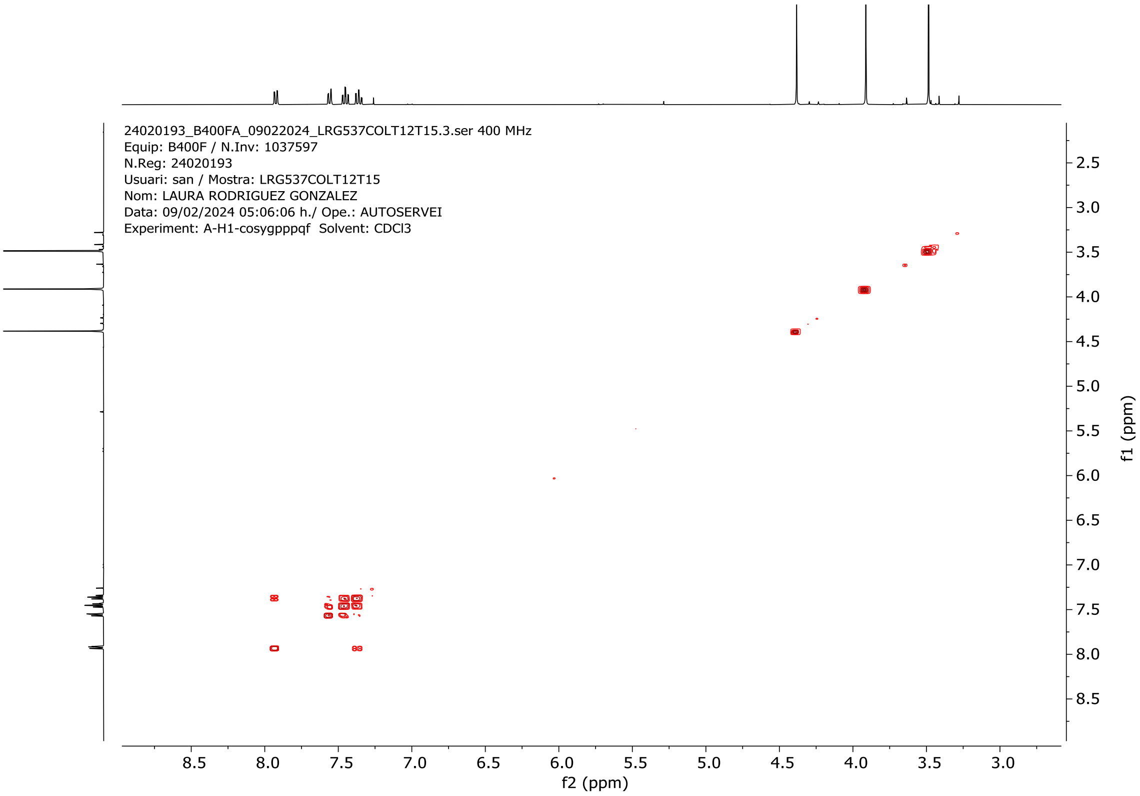

2D-HSQC

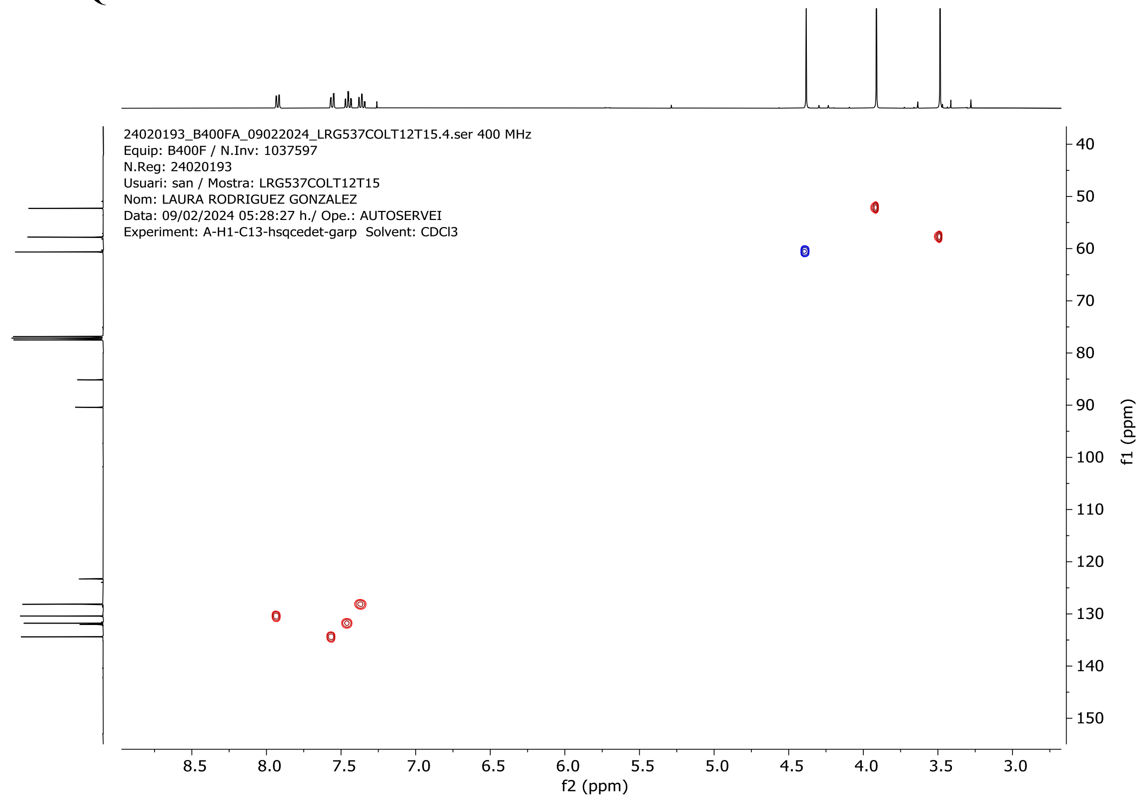

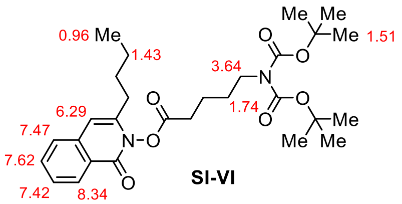

SI-VI

24020465_B400FA_15022024_ASV007DRY.1.fid 1H 400 MHz
Equip: B400F / N.Inv: 1037597
N.Reg: 24020465
Usuari: san / Mostra: ASV007DRY
Nom: LAURA RODRIGUEZ GONZALEZ
Data: 15/02/2024 15:27:09 h./ Ope.: AUTOSERVEI
Experiment: A-H1-zg30 Solvent: CDCl3

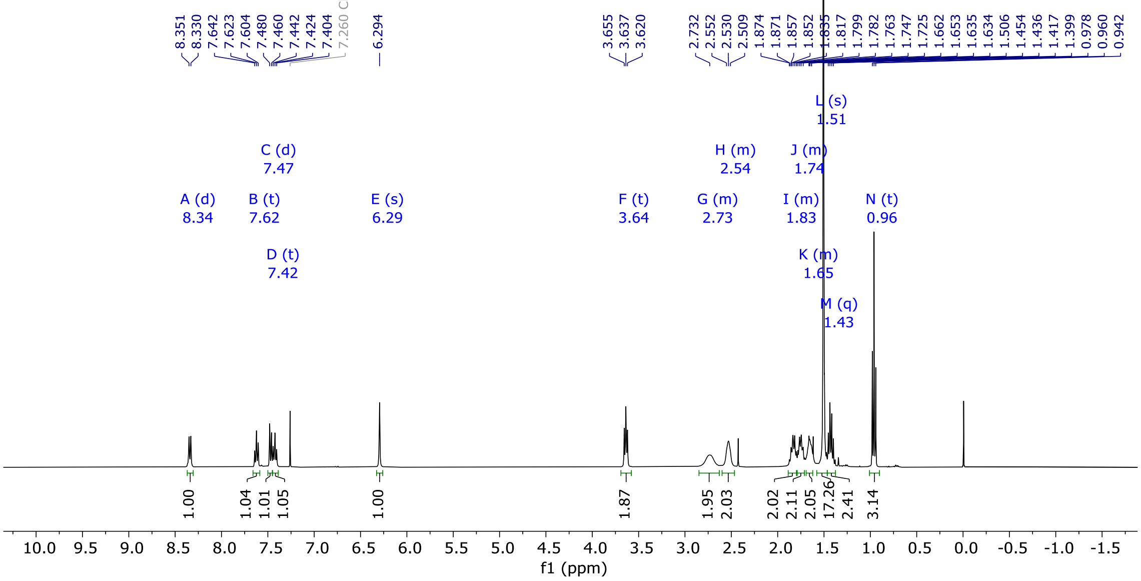

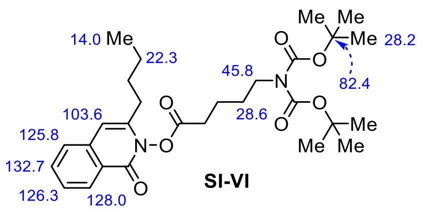

SI-VI

24020410_B400FA_14022024_ASV007CH.2.fid 13C{1H} 101 MHz
Equip: B400F / N.Inv: 1037597
N.Reg: 24020410
Usuari: san / Mostra: ASV007CH
Nom: LAURA RODRIGUEZ GONZALEZ
Data: 14/02/2024 21:48:47 h./ Ope.: AUTOSERVEI
Experiment: A-C13-zgpg30 Solvent: CDCl3

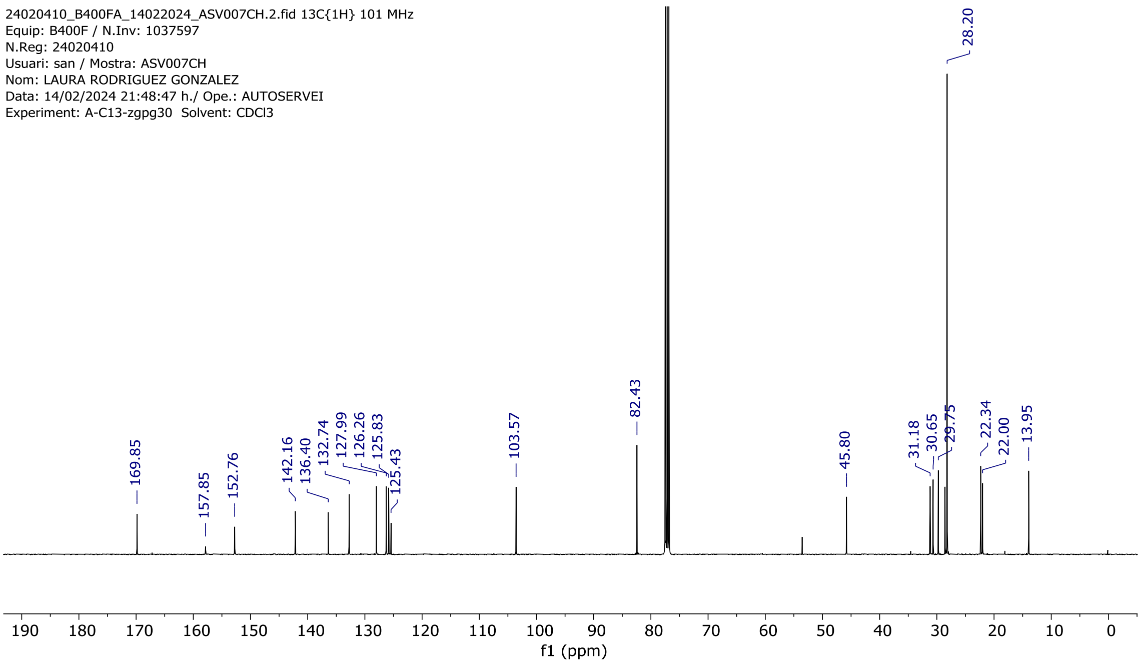

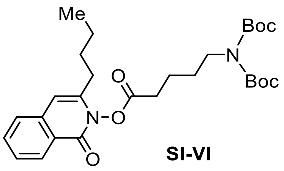

SI-VI

2D-COSY

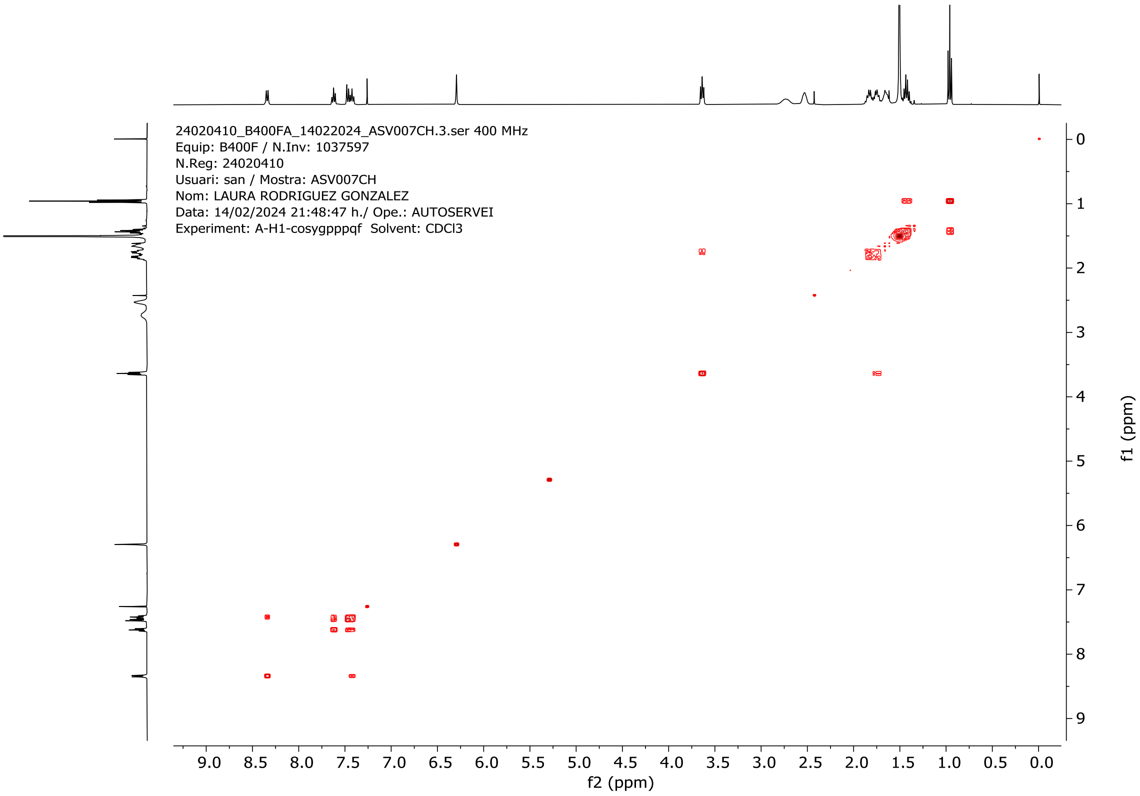

2D-HSQC

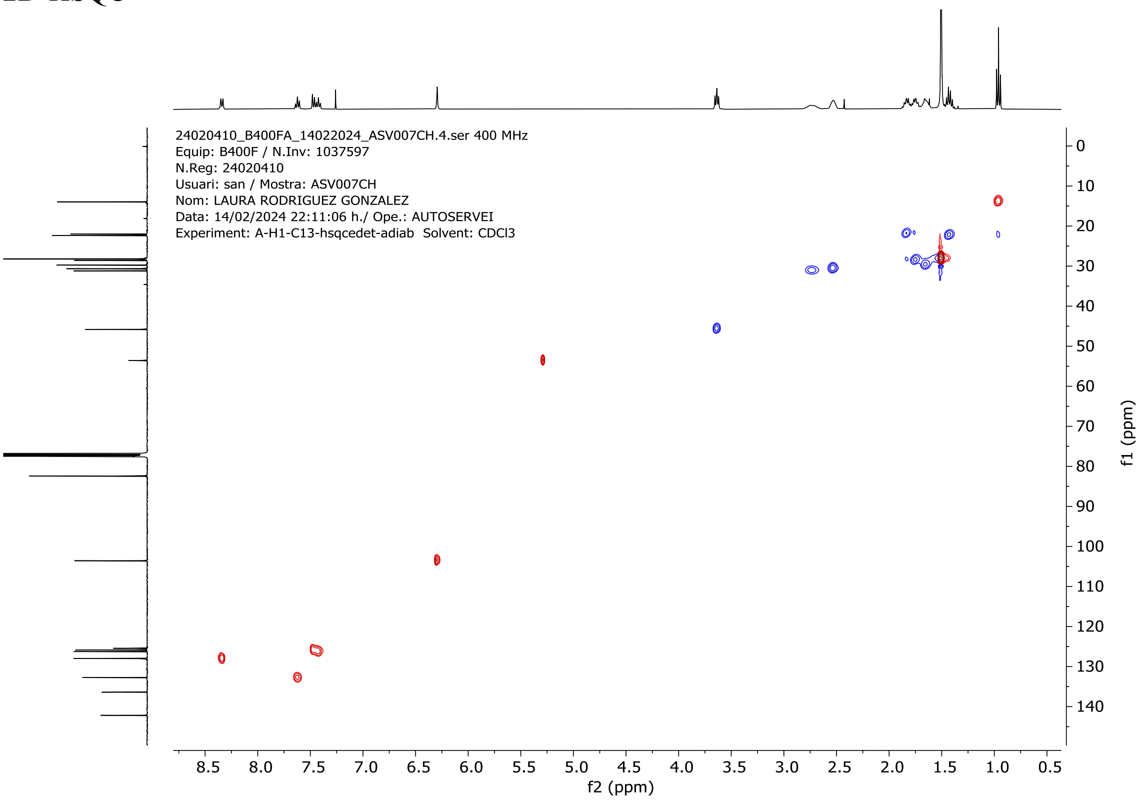

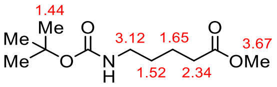

SI-VII

24020244_B400FA_11022024_LRG536COLT17T20CH.1.fid 1H 400 MHz
Equip: B400F / N.Inv: 1037597
N.Reg: 24020244
Usuari: san / Mostra: LRG536COLT17T20CH
Nom: LAURA RODRIGUEZ GONZALEZ
Data: 09/02/2024 11:03:12 h./ Ope.: AUTOSERVEI
Experiment: A-H1-zg30 Solvent: CDCl3

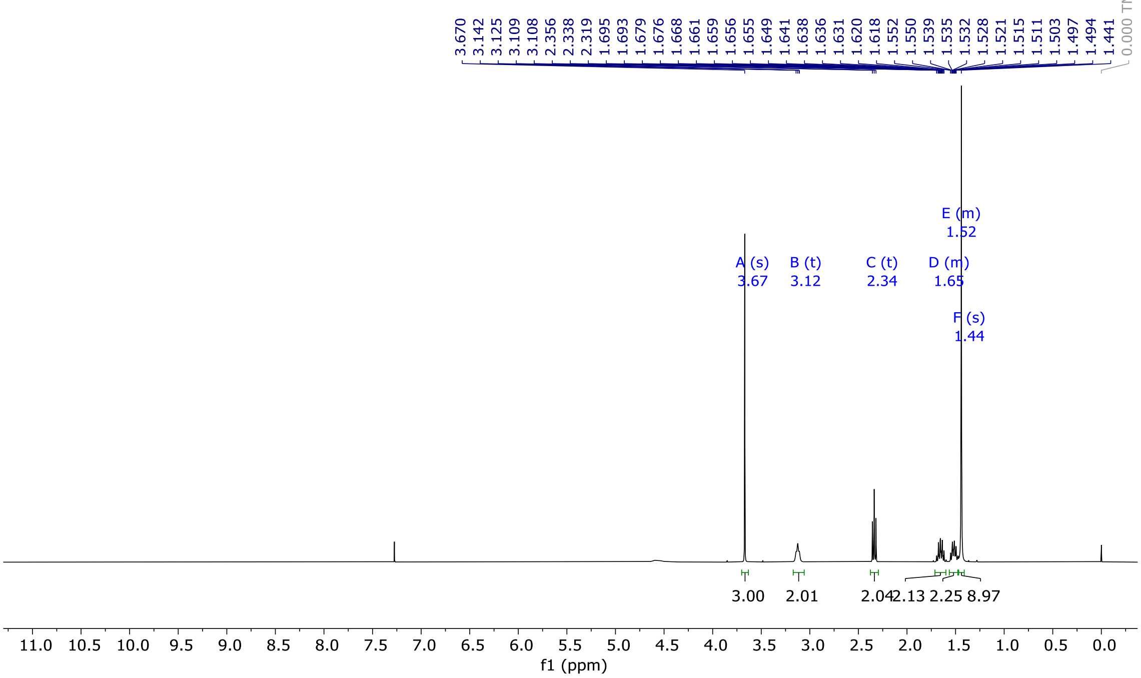

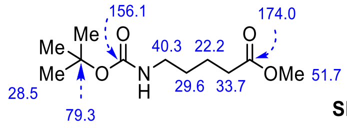

SI-VII

24020244_B400FA_11022024_LRG536COLT17T20CH.2.fid 13C{1H} 101 MHz
Equip: B400F / N.Inv: 1037597
N.Reg: 24020244
Usuari: san / Mostra: LRG536COLT17T20CH
Nom: LAURA RODRIGUEZ GONZALEZ
Data: 11/02/2024 04:08:51 h./ Ope.: AUTOSERVEI
Experiment: A-C13-zgpg30 Solvent: CDCl3

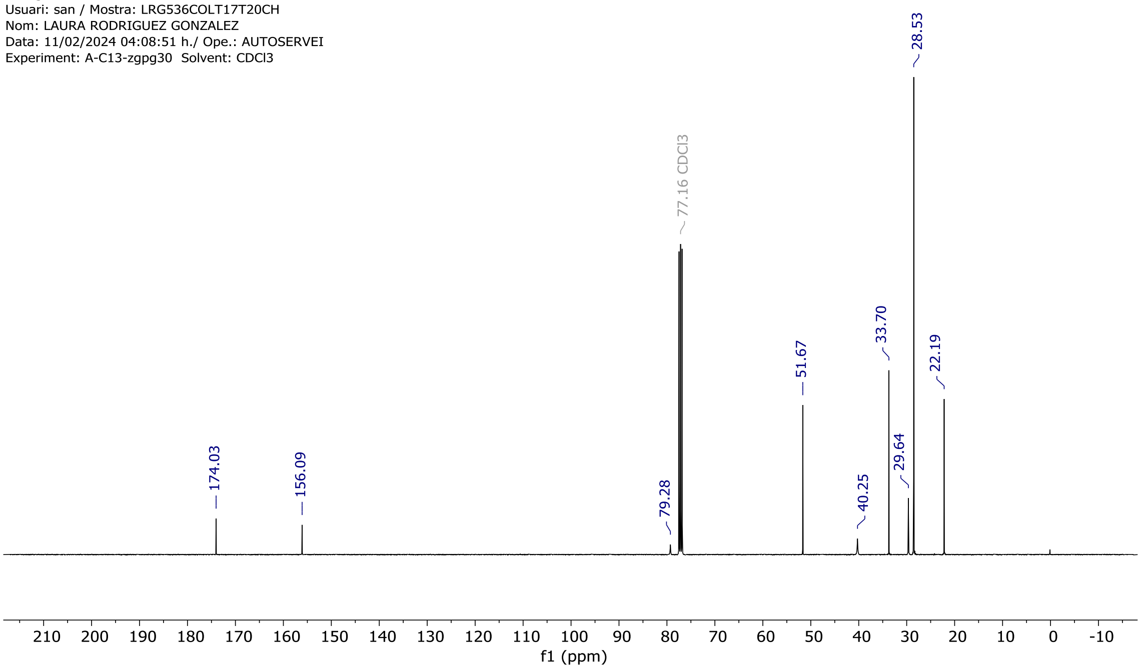

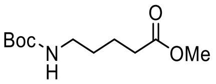

SI-VII

2D-COSY

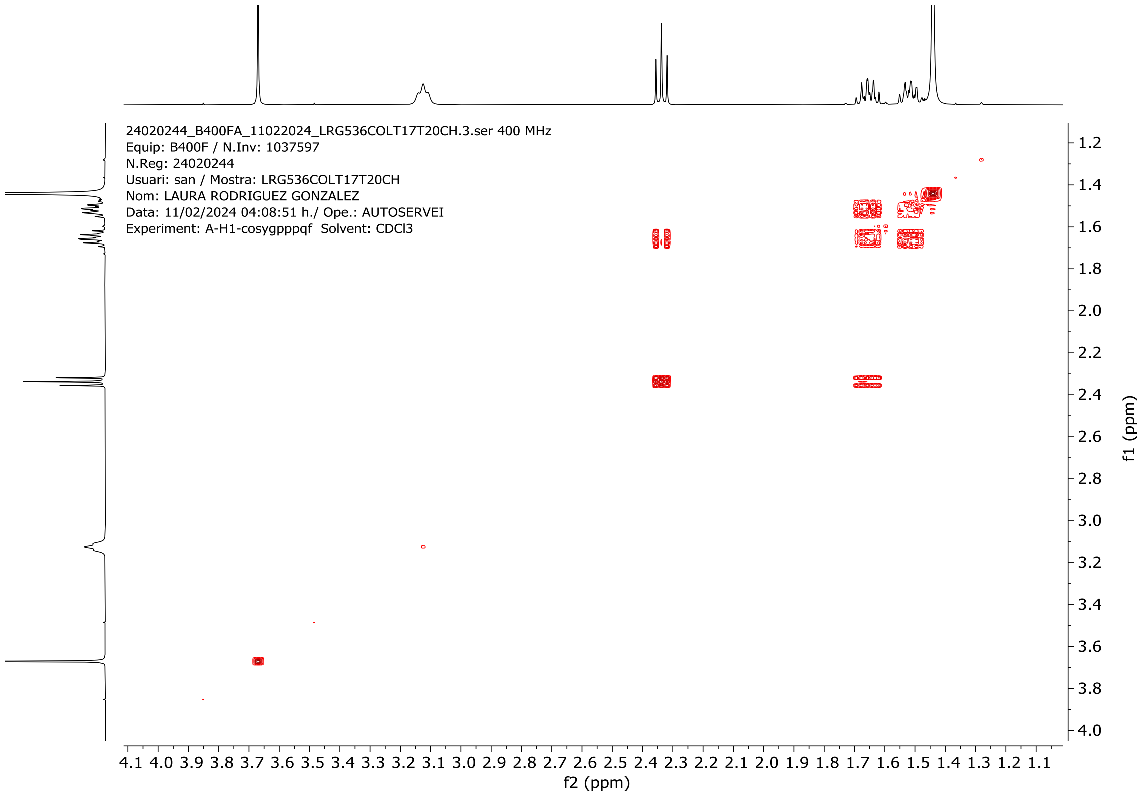

2D-HSQC

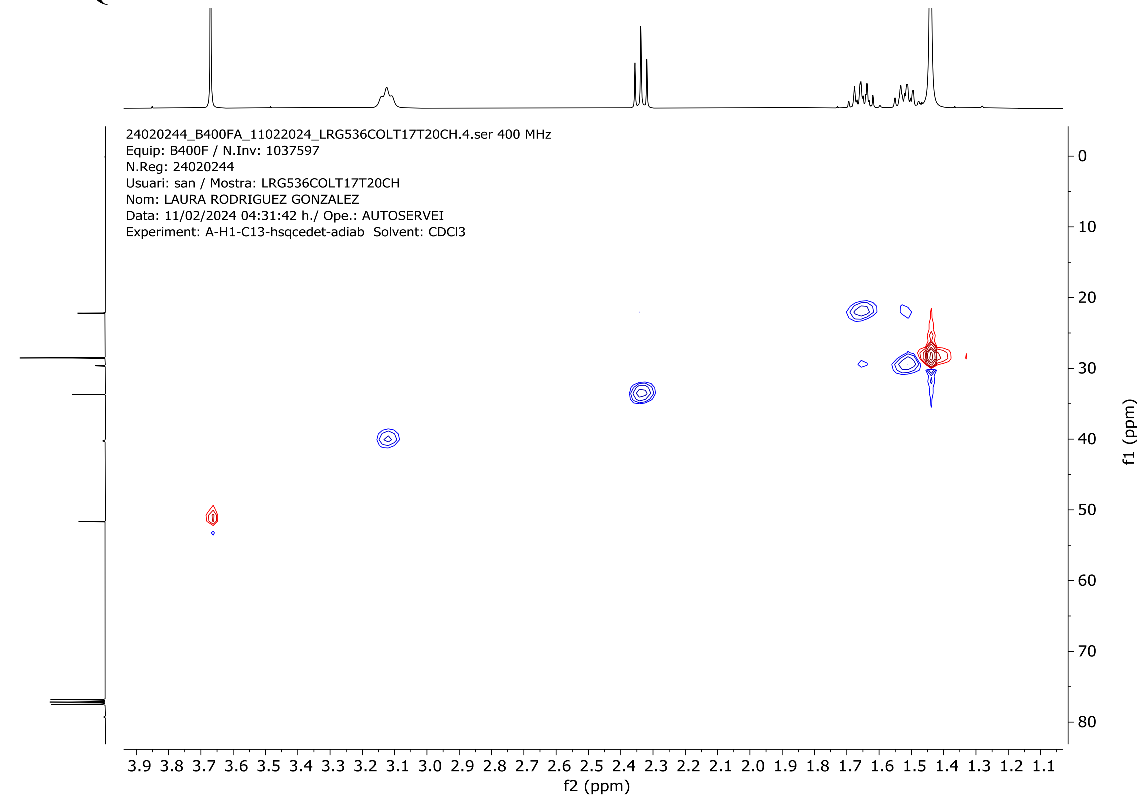

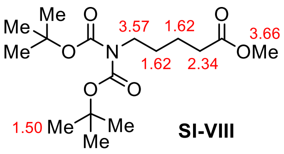

SI-VIII

995-2024_B400FA_09022024_LRG536T5T15CH.1.fid 1H 400 MHz
Equip: B400F / N.Inv: 1037597
N.Reg: 995/2024
Usuari: san / Mostra: LRG536T5T15CH
Nom: LAURA RODRIGUEZ GONZALEZ
Data: 08/02/2024 13:07:06 h./ Ope.: servei Unitat RMN
Experiment: A-H1-zg30 Solvent: CDCl3

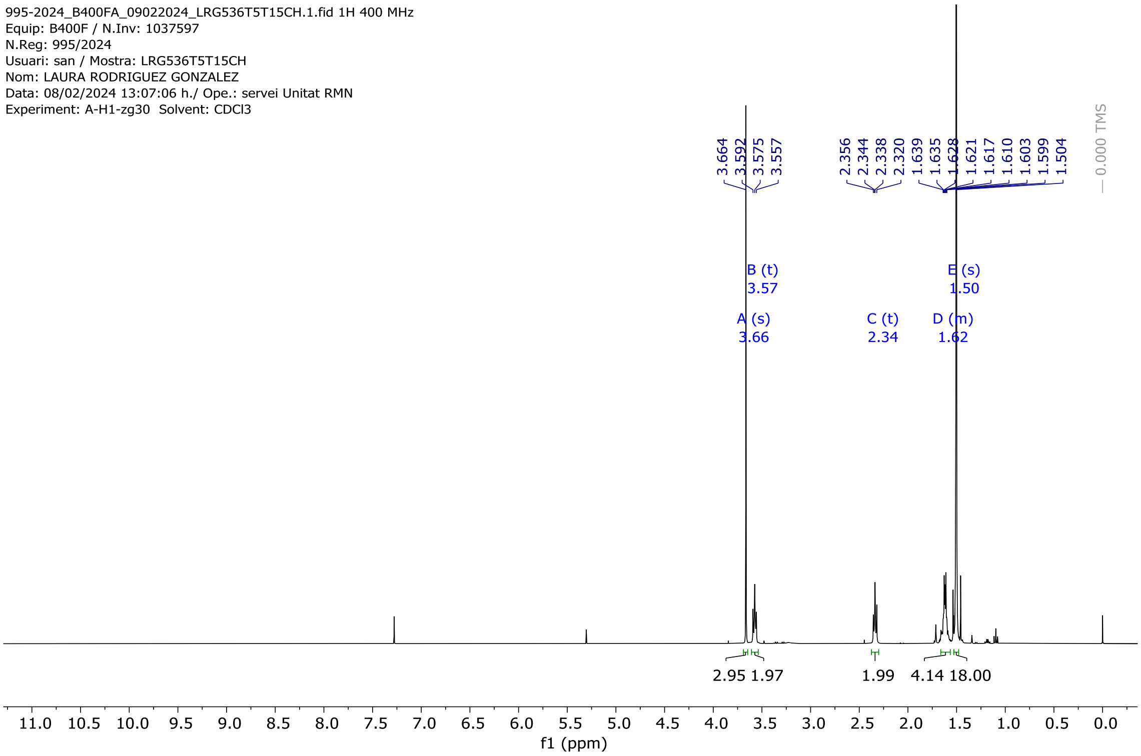

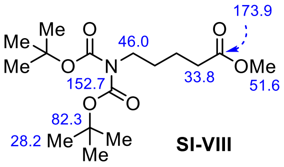

SI-VIII

995-2024_B400FA_09022024_LRG536T5T15CH.4.fid 13C{1H} 101 MHz
Equip: B400F / N.Inv: 1037597
N.Reg: 995/2024
Usuari: san / Mostra: LRG536T5T15CH
Nom: LAURA RODRIGUEZ GONZALEZ
Data: 08/02/2024 21:52:42 h./ Ope.: servei Unitat RMN
Experiment: A-C13-zgpg30 Solvent: CDCl3

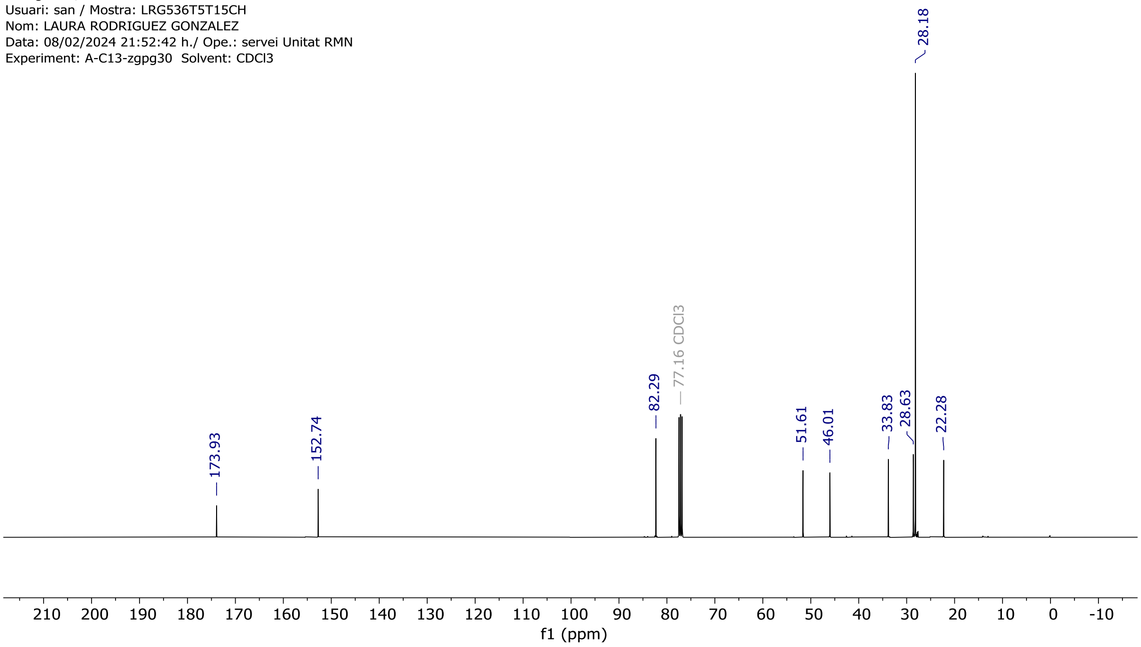

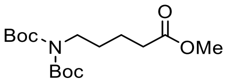

SI-VIII

2D-COSY

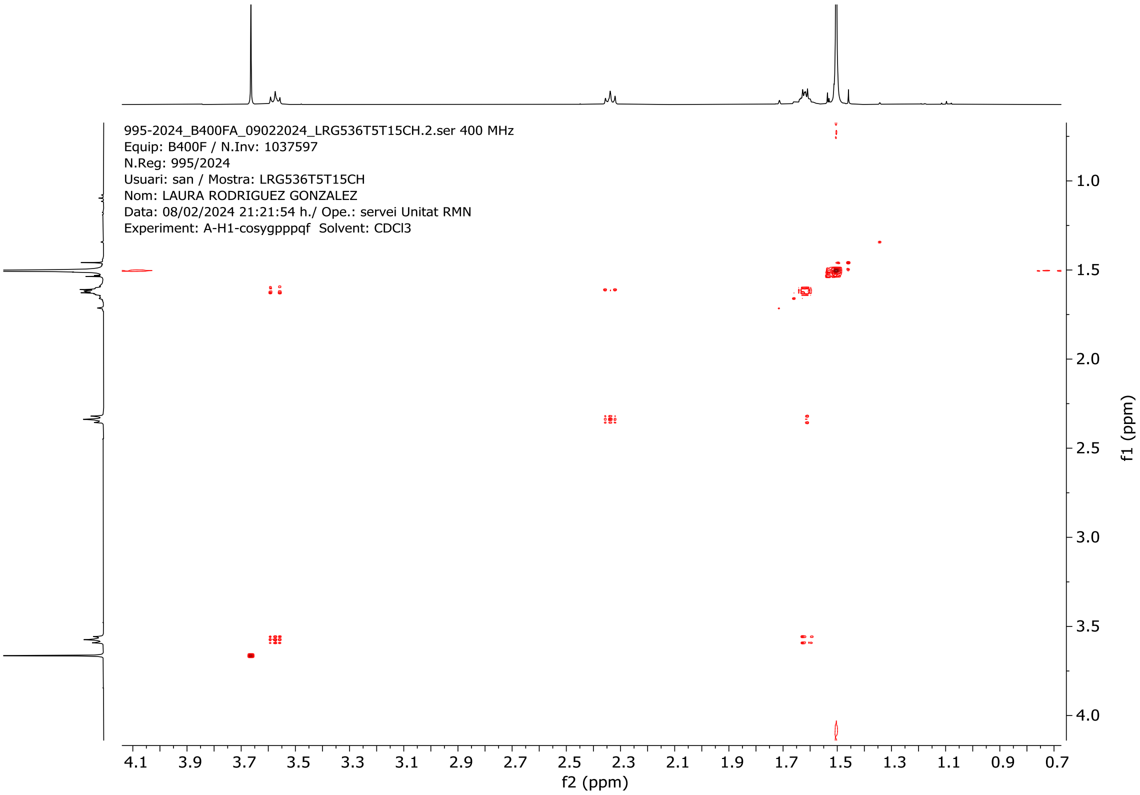

2D-HSQC

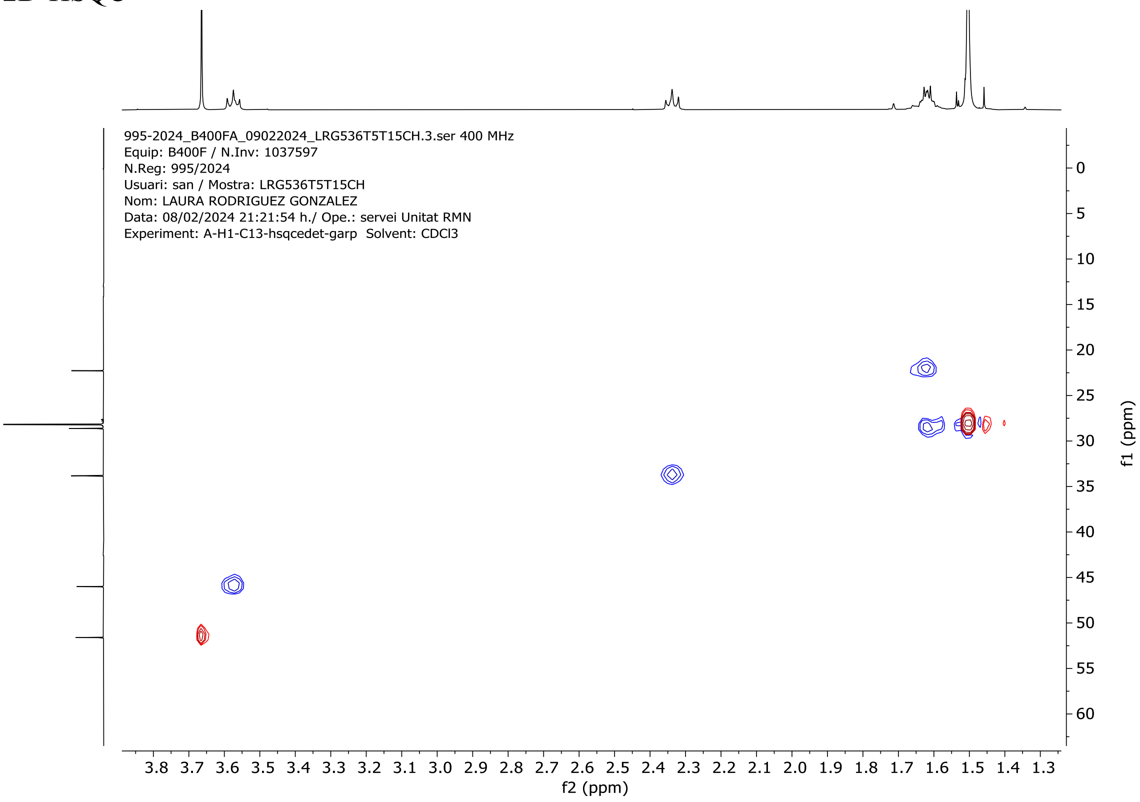

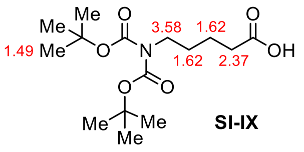

SI-IX

24020280_B400FA_12022024_LRG538CH.1.fid 1H 400 MHz
Equip: B400F / N.Inv: 1037597
N.Reg: 24020280
Usuari: san / Mostra: LRG538CH
Nom: LAURA RODRIGUEZ GONZALEZ
Data: 09/02/2024 16:50:24 h./ Ope.: AUTOSERVEI
Experiment: A-H1-zg30 Solvent: CDCl3

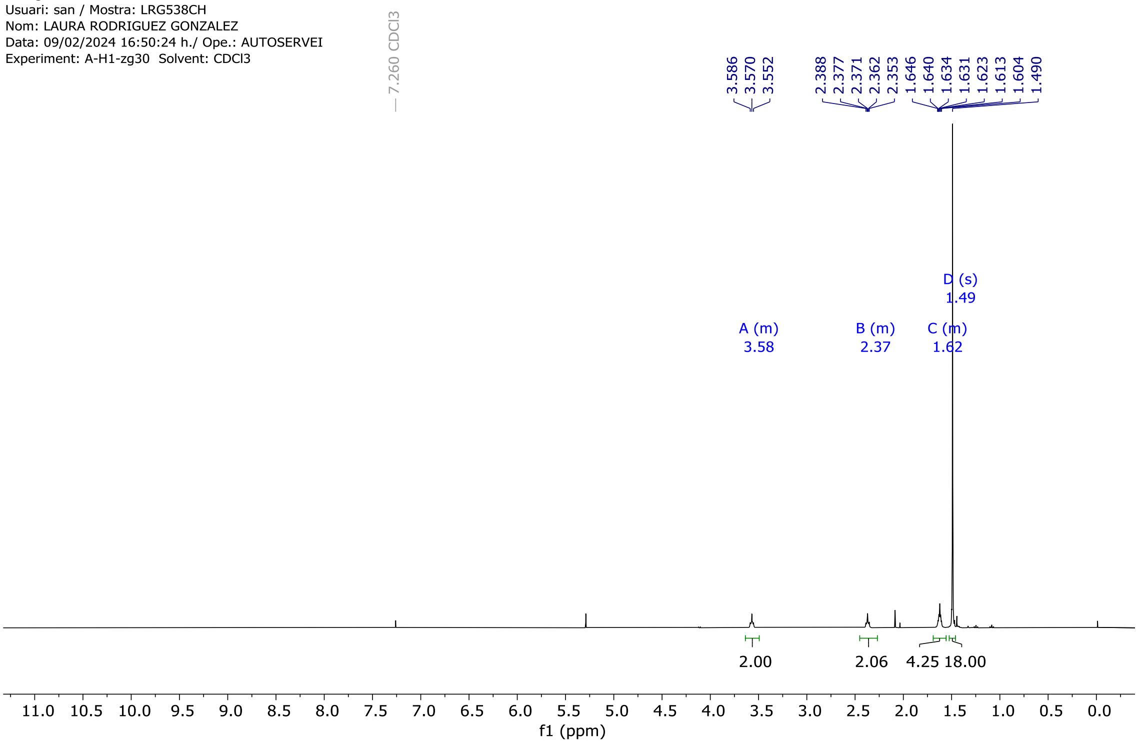

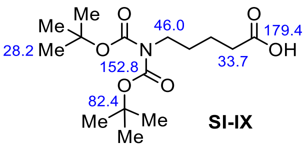

SI-IX

24020280_B400FA_12022024_LRG538CH.2.fid 13C{1H} 101 MHz
Equip: B400F / N.Inv: 1037597
N.Reg: 24020280
Usuari: san / Mostra: LRG538CH
Nom: LAURA RODRIGUEZ GONZALEZ
Data: 12/02/2024 01:46:56 h./ Ope.: AUTOSERVEI
Experiment: A-C13-zgpg30 Solvent: CDCl3

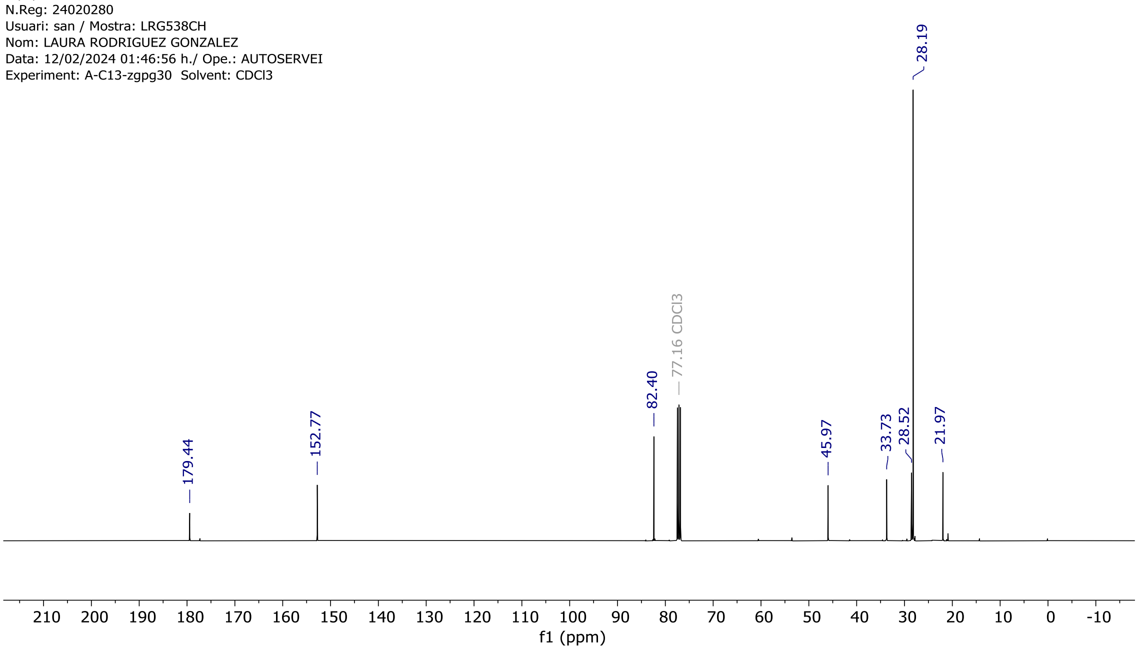

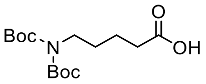

SI-IX

2D-COSY

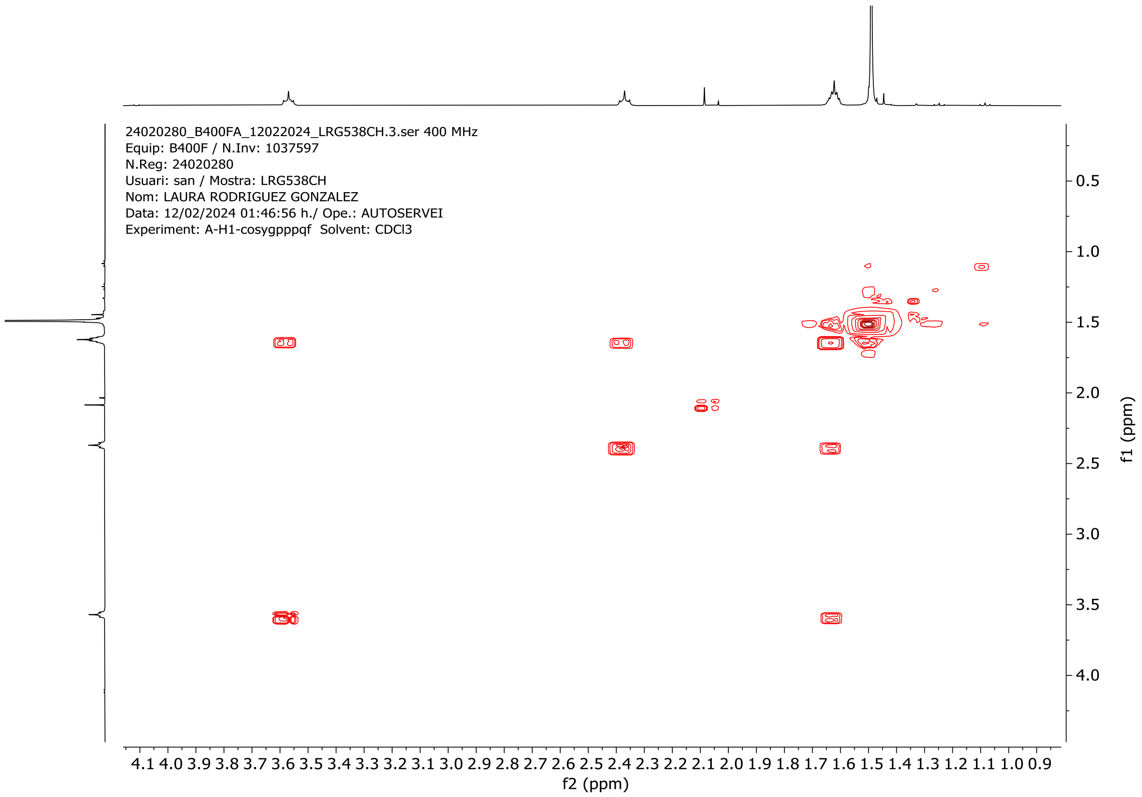

2D-HSQC

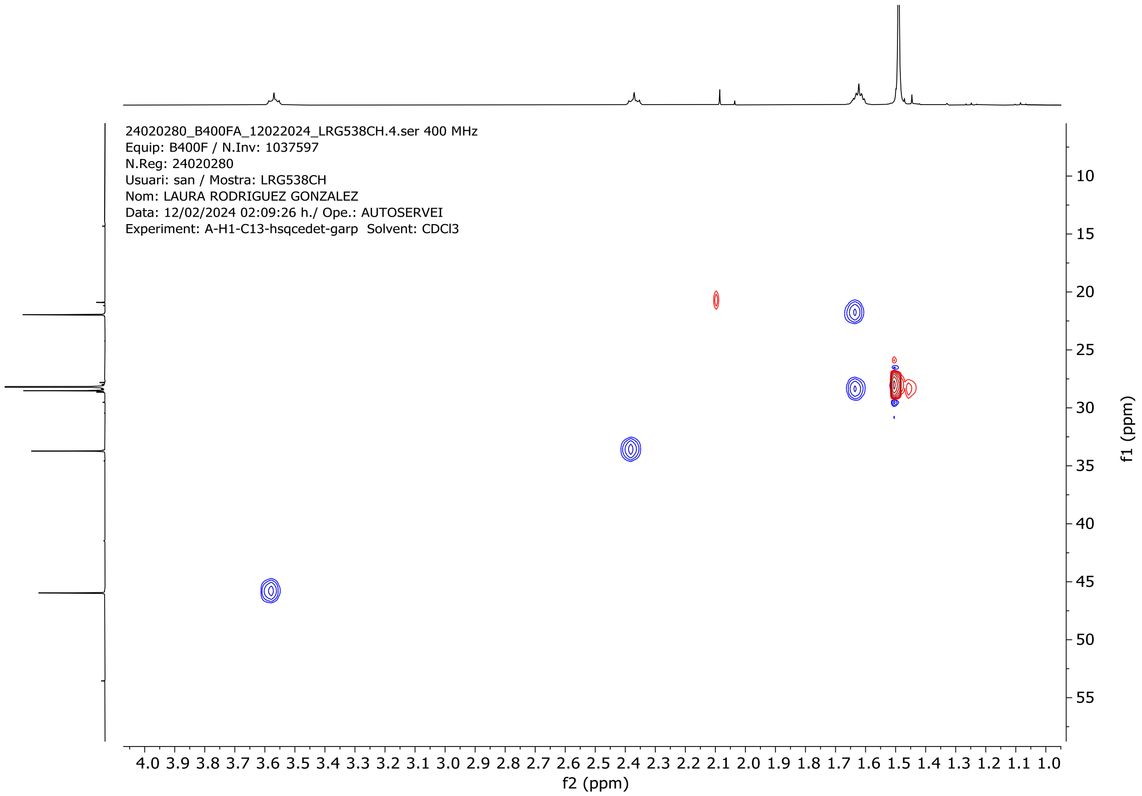

Deuterium labelling experiment of compound 3

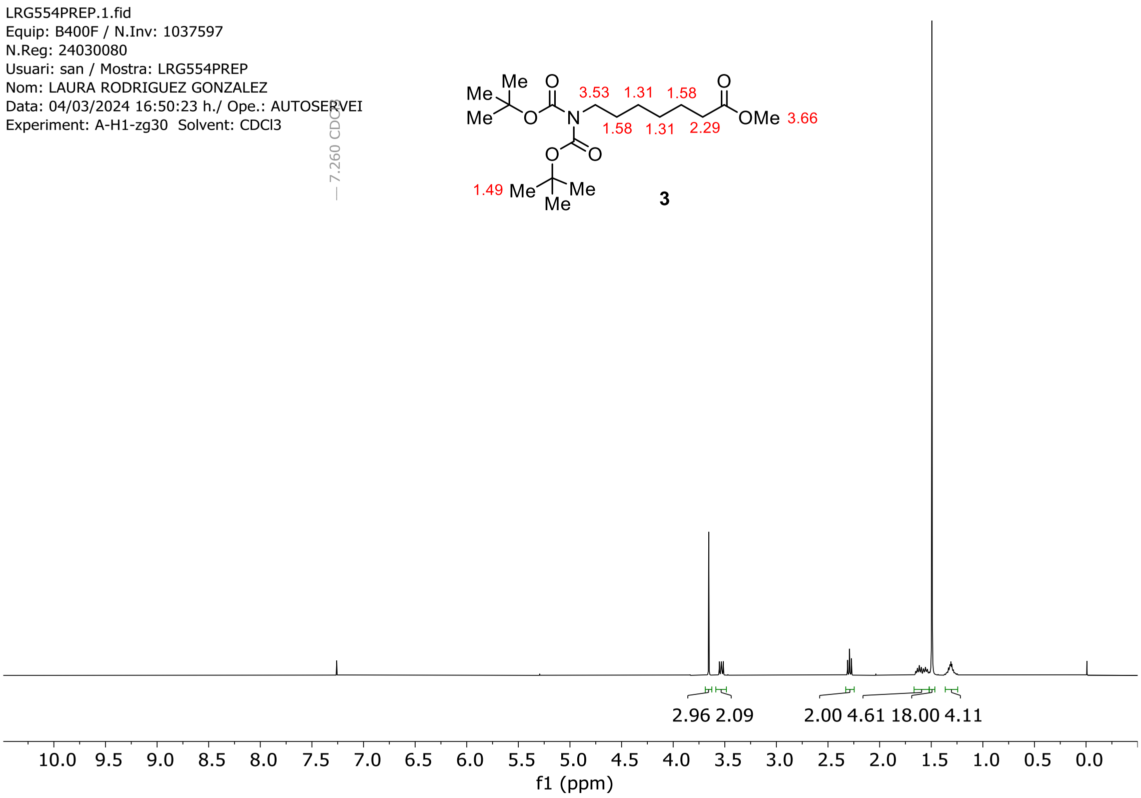

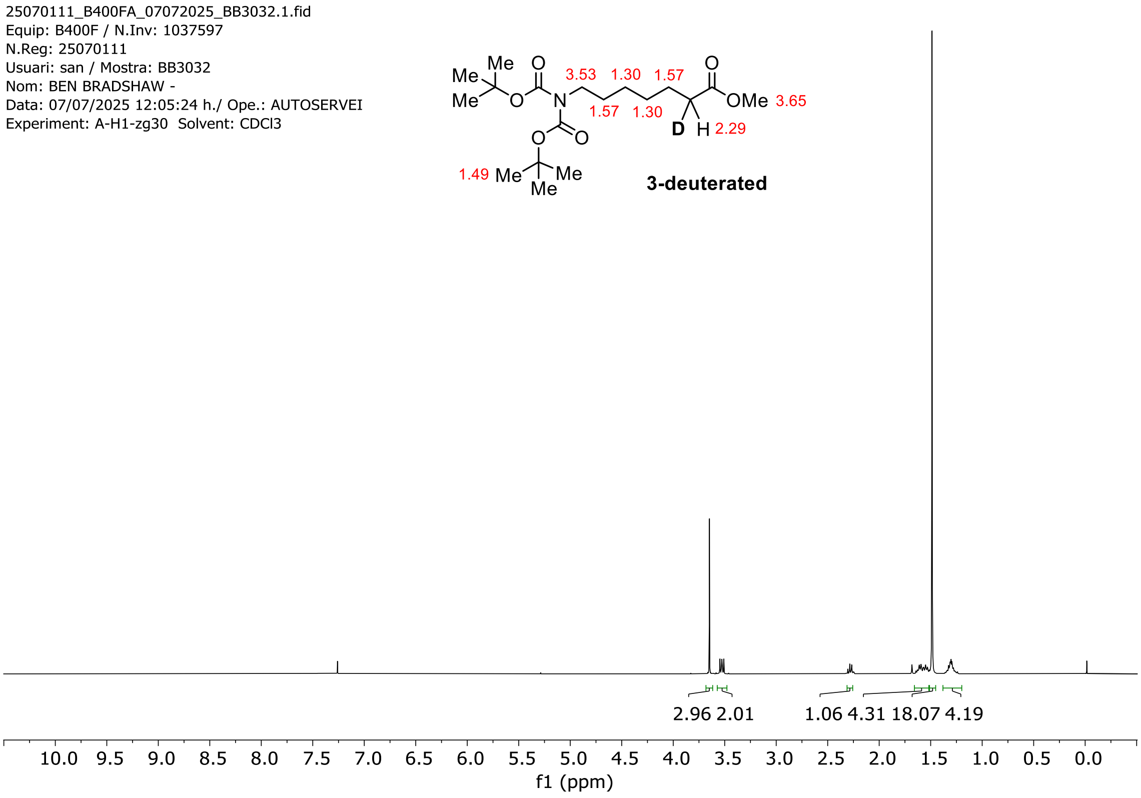

Supplement: SC-016-D5SC04274G-s001 [file SC-016-D5SC04274G-s001.pdf]
